# Affinity Enhancement by Dendritic Side Chains in Synthetic Carbohydrate Receptors**

**DOI:** 10.1002/anie.201409124

**Published:** 2015-01-21

**Authors:** Harry Destecroix, Charles M Renney, Tiddo J Mooibroek, Tom S Carter, Patrick F N Stewart, Matthew P Crump, Anthony P Davis

**Affiliations:** School of Chemistry, University of Bristol, Cantock's CloseBristol, BS8 1TS (UK)

**Keywords:** carbohydrates, dendrimers, molecular recognition, receptors, supramolecular chemistry

## Abstract

Dendritic side chains have been used to modify the binding environment in anthracene-based synthetic carbohydrate receptors. Control of length, charge, and branching enabled the positioning of side-chain carboxylate groups in such a way that they assisted in binding substrates rather than blocking the cavity. Conformational degeneracy in the dendrimers resulted in effective preorganization despite the flexibility of the system. Strong binding was observed to glucosammonium ions in water, with *K*_a_ values up to 7000 m^−1^. Affinities for uncharged substrates (glucose and *N*-acetylglucosamine) were also enhanced, despite competition from solvent and the absence of electrostatic interactions.

The binding of polar molecules in aqueous solution remains a major problem for supramolecular chemistry. Whereas apolar molecules interact poorly with water and are readily bound through the hydrophobic effect, polar species and binding sites are well-hydrated. Binding requires that water be displaced from both partners, and the energetic consequences may be unpredictable. The problem is especially difficult if the targets and/or binding sites bear hydroxy groups, which resemble water molecules yet must be distinguished from solvent. Thus, carbohydrate recognition, an important biological process,[1] is especially challenging.[2,3]

In previous studies we have shown that certain carbohydrates can be bound by amphiphilic cavities, which complement both polar and apolar regions in their targets.[4] For example, both tricycle **1**[4a] and monocycle **2**[4b] bind glucose **3** 
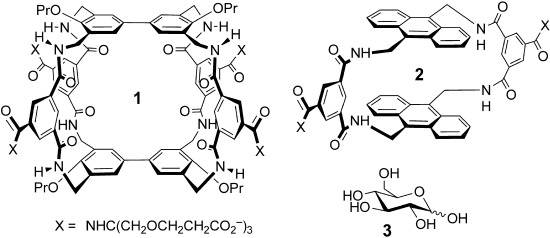
by combining aromatic surfaces (complementary to axial CH groups) with annular amides (complementary to equatorial OH groups). Binding constants are modest at *K*_a_≈60 m^−1^, but selectivities are good (e.g. 20:1 glucose/galactose). Moreover, owing to the high concentrations of glucose in biological fluids, the low affinities do not preclude applications in medical glucose sensing.[4a]

A major aim of this research is to show how particular interactions and supramolecular principles might help to drive and control carbohydrate recognition, in both natural and synthetic systems.[5] The role of polar interactions is especially interesting. Although the contribution from CH–π and hydrophobic effects is readily understood,[6] it is less evident how polar binding groups can be deployed to increase affinities in water. Moreover, studies of this problem are handicapped by design and synthetic issues. In both **1** and **2**, the polar interactions are provided by amide groups, which are intrinsic to the framework, and altering these groups could change the positioning of the hydrophobic surfaces (in some cases with destruction of the cavity). The addition of polar groups to the receptor cores might be feasible at some points, but all such changes would require major synthetic effort.

Faced with this problem, we realized that one position where changes could readily be made, especially in monocyclic **2**, is in the solubilizing groups X. At first sight, such modifications should make little difference to the binding properties. However, if X is dendritic[7] and the size and bulk of the dendrimer is adjusted upwards, terminal groups are located close to the opening of **2**, as required to make contact with the polar groups in bound substrates (Figure 1). The terminal groups will be connected to the core through a flexible chain, so will not be preorganized for binding. However, as one end group moves away, another can move into range. A level of preorganization is therefore achieved through the symmetry and degeneracy of the dendron structure. Effects should be substantial if electrostatic forces can be invoked, but might also be significant for neutral substrates. Herein we report experiments which show that the solubilizing side chains in synthetic carbohydrate receptors can indeed be used to enhance binding. The effect has been exploited to create some of the highest affinities yet observed for biomimetic carbohydrate recognition in water.

**Figure 1:**
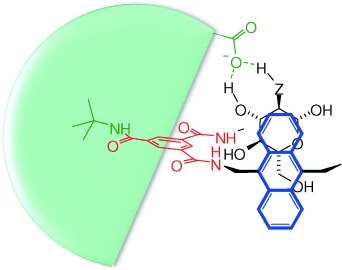
Schematic view of a bisanthracenyl carbohydrate receptor (analogous to 2) binding glucose (Z=O) or glucosammonium (Z=NH_2_^+^) with the aid of hydrogen bonding from a polycarboxylate dendritic side chain (green).

The side chains in **2** are derived from the amine **10** (Scheme 1), available in one step from *tert*-butyl acrylate and tris(hydroxymethyl)aminomethane.[8] As a first test of the concept, we planned to use a second-generation dendrimer constructed with this unit. Accordingly, we prepared the receptor **7** (Scheme 1) by the general route shown in Scheme 2[9] and investigated the binding of this macrocycle to glucose in water by ^1^H NMR spectroscopy. To our initial surprise, the spectra yielded no evidence of complexation. However, further investigation revealed NOE connections between side-chain hydrogen atoms and inward-directed receptor hydrogen atoms (NH, ArH), thus implying that terminal strands from the side chain can thread through the cavity (see Figure S37 in the Supporting Information). Modeling confirmed that such threading was feasible (see Figure S183).

**Scheme 1:**
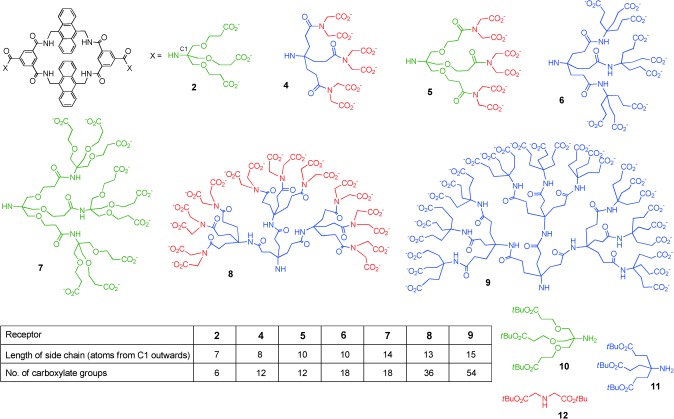
Carbohydrate receptors and side-chain components. Although side chains are shown as fully ionized for simplicity, some carboxylate groups will be protonated at pH 7. For further discussion, see the Supporting Information.

**Scheme 2:**
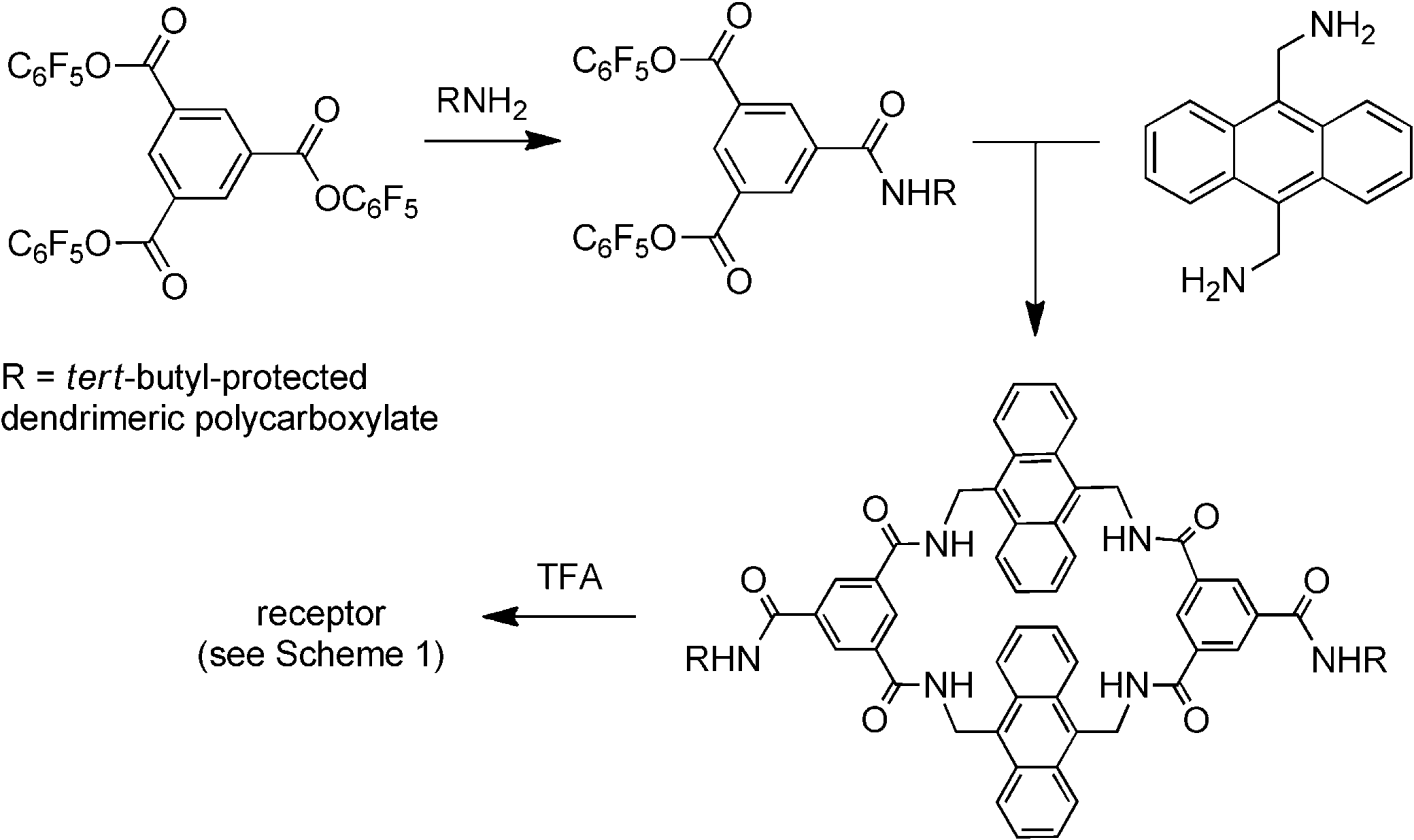
Synthetic route to the receptors in Scheme 1. TFA=trifluoroacetic acid.

Although the effects were negative, the results with **7** confirmed that dendrimeric side chains could indeed influence binding properties. To exploit this principle constructively, we synthesized a range of receptors with controlled side-chain length, charge, and steric bulk (Scheme 1). The side chains were constructed from **10**, which gives units with relatively long branches; the triester **11**,[10] which gives medium-length units; and di-*tert*-butyl iminodiacetate (**12**), which gives short branches. In particular, by restricting the overall length and terminating the dendrimers with **11** or **12**, we expected to prevent the threading which had disabled **7**. Four of the series possessed second-generation (G2) dendrimer side chains (including **7**), whereas two contained G3 side chains (**8** and **9**). Side-chain lengths varied fairly smoothly from 7 to 15 atoms, while the overall charge ranged from −6 to −54.

All receptors dissolved readily in D_2_O to give well-resolved, concentration-independent ^1^H NMR spectra (up to a concentration of about 4 mm). Their binding properties towards carbohydrates were investigated primarily by ^1^H NMR titrations. The substrates included the aminosugars glucosammmonium **13**⋅H^+^ and galactosammonium **14**⋅H^+^, 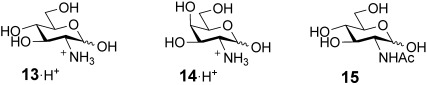
for which electrostatic interactions could contribute to binding. With the exception of **7** (see above), titrations of the receptors with all-equatorial saccharides, such as glucose, caused signal movements similar to those observed earlier for **3**. In particular, substantial downfield shifts were observed for the inward-facing isophthaloyl hydrogen atoms (see, for example, Figure 2). Plotting of the change in shift of these hydrogen atoms against substrate concentration gave plots that fitted well to a 1:1 binding model in most instances and yielded the binding constants *K*_a_ listed in Table 1. In some cases, supporting values were obtained from isothermal titration calorimetry (ITC) and fluorescence titrations (see Table 1).

**Figure 2:**
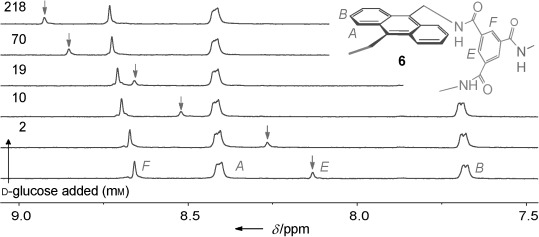
Partial ^1^H NMR spectra, with assignments, from the titration of 6 (0.23 mm) with D-glucose (0–218 mm) in D_2_O at 298 K. The movement of the signal for hydrogen atom E (internally directed isophthalimide CH) is highlighted by arrows.

**Table 1:** Data from the measurement of binding constants to carbohydrates in aqueous solution.[a]

Receptor	2	4	5	6	8	9
Length of side chain[b] (overall charge)	7 (−6)	8 (−12)	10 (−12)	10 (−18)	13 (−36)	15 (−54)
	
Association constant (*K*_a_ [m^−1^]) with:						
D-glucosamine (**13**)[c]	160[d]	1400 (1500[e])	2000 (1700[e])	2400 (2100[e])	7000 (9700[e])	610
D-glucosamine (**13**; 20 mm NaCl)[c]	–[f]	330	420	690	1660	151
D-glucosamine (**13**; 154 mm NaCl)[c]	–[f]	97 (76[g])	135	222< (226[g])	340	53
D-galactosamine (**14**; 154 mm NaCl)[c]	–[f]	–[h]	27	33	98	4
D-glucose (**3**)	56 (55,[g] 58[e])	70 (65,[g] 75[e])	89 (91[e])	90 (81,[h] 87[e])	69 (41[e])	4 (6[e])
methyl β-D-glucoside	96 (101,[g] 121[e])	87 (87[g])	124	115 (120[g])	92	–[f]
*N*-acetyl-D-glucosamine (**15**)	9	19	25	31	33	–[f]
D-galactose	4[d]	6	6	7	3	–[f]
D-mannose	0[i]	0[i]	0[i]	0[i]	0[i]	–[f]
	
Limiting fluorescence change (*F*/*F*_0_)[j] with:						
D-glucose	2.5	3.7	3.4	3.7	2.0	2.2

[a] Association constants *K*_a_ were measured by ^1^H NMR titration in D_2_O at 298 K unless otherwise noted. Calculated errors from curve fitting were typically ≤5 %. Data for **2** are from Ref. [4b] unless otherwise noted. See the Supporting Information for experimental details, spectra, and binding curves, including results with additional substrates.

[b] Number of atoms from C1 outwards (see Scheme 1).

[c] Measurements were made at pH 7. For details of conditions and procedures, see the Supporting Information.

[d] The *K*_a_ value was measured/remeasured as part of the present study.

[e] The *K*_a_ value was measured by fluorescence titration in H_2_O.

[f] The *K*_a_ value was not determined. At these salt concentrations, receptor **2** gives broadened ^1^H NMR spectra, presumably as a result of aggregation.

[g] The *K*_a_ value was measured by ITC in H_2_O.

[h] Poor fit to a 1:1 binding model, thus suggesting multiple stoichiometries.

[i] Approximate value. The relationship between the signal position and the concentration was almost linear.

[j] Emission at 423 nm, excitation at 395 nm.

The data reveal that the side chains in these bisanthracenyl receptors can indeed be tuned to enhance binding properties and adjust selectivity. As expected, the most dramatic effects were observed for glucosammonium **13**⋅H^+^, which is all-equatorial (and is therefore complementary to the cavity) and which is charge-complementary to the side chains. As the dendrimers expanded, affinities for **13**⋅H^+^ increased from 160 m^−1^ (for **2**) to 7000 m^−1^ (for **8**).[11] Modeling confirmed that both side chains in **8** can contribute to binding through strain-free salt bridges (Figure 3).[12] Support was provided by the NOESY spectra of **8** and **8**⋅glucosammonium. Both showed NOE connections between anthracenyl hydrogen atoms and the terminal side-chain CH_2_ groups, a result consistent with the presence of side-chain carboxylate groups near the entrance to the cavity (see Figures S38 and S39). Interestingly, the next step to **9** was counterproductive, despite the increase in negative charge. NOE measurements on free **9** suggested that, as for **7**, terminal side-chain groups are able to penetrate the cavity (see Figure S40). The affinity for glucosammonium was lowered by competing ions, as expected for electrostatic interactions,[13] but *K*_a_ values remained quite high even at physiological salt levels (154 mm NaCl). Galactosammonium **14**⋅H^+^ was bound 3–13 times less strongly than glucosammmonium, thus showing that the all-equatorial preference of the cavity is maintained. The simple nonsaccharidic amine tris(hydroxymethyl)aminomethane (Tris) was also tested as a control substrate for receptor **6**. A ^1^H NMR titration at pH 7 produced just minor changes to the receptor signals, which could not be analyzed to give a binding constant (see Figure S132).

**Figure 3:**
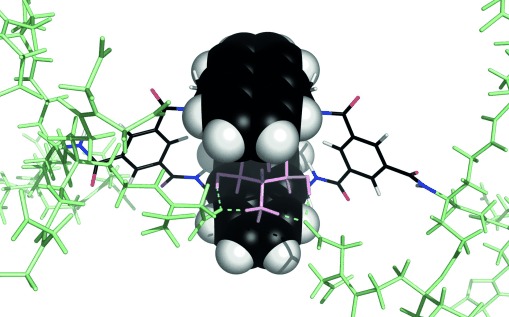
Model of 8 binding glucosammonium 13⋅H^+^, featuring salt-bridge formation by side chains on both sides of the receptor. Anthracene units are shown in the space-filling mode. Substrate atoms are pink, side-chain atoms are pale green, and hydrogen bonds to terminal carboxylate groups are cyan. The structure was minimized without constraints by the use of MacroModel 10.3 (MMFFs force field, aqueous GB/SA solvation).

The studies on uncharged carbohydrates show that effects are not solely dependent on electrostatic interactions. Binding to glucose showed a modest but definite increase from 56 m^−1^ (for **2**) to approximately 90 m^−1^ (for **5** and **6**). Moreover, the affinity for *N*-acetylglucosamine (**15**) increased by a factor of about 4 on moving from **2** to **8**. Modeling indicates that intracomplex O^−^⋅⋅⋅HO and O^−^⋅⋅⋅HN hydrogen bonds are geometrically feasible in most of these systems, but are likely to be more effective for the longer side chains owing to lower strain.[14] The experiments suggest that both interactions, but especially O^−^⋅⋅⋅HN, can be deployed to enhance binding in water despite competing solvation.

Finally, an unexpected outcome of this study was the discovery that the fluorescence response to glucose binding was also affected by the side chains. For prototype **2**, we had previously observed that emission increased by a limiting value of 2.5 upon titration with glucose (based on the 1:1 binding model). The G2 dendrons in **4**, **5**, and **6** raised this value as high as 3.7 (Table 1). Combined with the higher affinities for glucose, the result indicates an approximately twofold increase in sensitivity to changes in the glucose concentration in the range 0–10 mm (see Figure S182). As this is the concentration range of greatest relevance to diabetes, the new systems may have practical significance.

In conclusion, we have shown that dendritic side chains can be used to moderate the binding properties of synthetic carbohydrate receptors as well as serving as solubilizing groups. In particular, the strategy was used to increase affinities for glucosamine to values similar to the highest reported[3g] and among the largest observed for any monosaccharide substrate. Effects were also observed for neutral substrates, despite the absence of electrostatic attraction. The study provides a rare example of the deliberate positioning of polar groups to enhance carbohydrate binding in water. Dendritic side chains will be useful components of future designs, as they provide an element of control that is independent of macrocycle structure while ensuring that water solubility is maintained even for hydrophobic core structures.
