# Supplementary material for: Affinity Enhancement by Dendritic Side Chains in Synthetic Carbohydrate Receptors
Source: Angew Chem Int Ed Engl. 2015 Jan 21;54(7):2057–61. doi: 10.1002/anie.201409124 (PMC4506558; doi:10.1002/anie.201409124)
Supplement: Supplementary file 1 [file anie0054-2057-sd1.pdf]

Angewandte Chemie
Eine Zeitschrift der Gesellschaft Deutscher Chemiker

Supporting Information
© Wiley-VCH 2015
69451 Weinheim, Germany

Affinity Enhancement by Dendritic Side Chains in Synthetic Carbohydrate Receptors**

*Harry Destecroix, Charles M. Renney, Tiddo J. Mooibroek, Tom S. Carter, Patrick F. N. Stewart, Matthew P. Crump, and Anthony P. Davis**

anie_201409124_sm_miscellaneous_information.pdf

CONTENTS

1. *Structures of Receptors and Carbohydrates* S3

2. *Synthesis of Receptors* S4

Synthesis of receptor **4** S5

Synthesis of receptor **5** S12

Synthesis of receptor **6** S20

Synthesis of receptor **7** S25

Synthesis of receptor **8** S31

Synthesis of receptor **9** S38

3. *2D NOESY Spectra* S44

4. *Binding Studies* S51

Table of results S54

Receptor **2** Carbohydrate Binding Studies S55

Receptor **4** Carbohydrate Binding Studies S56

Receptor **5** Carbohydrate Binding Studies S74

Receptor **6** Carbohydrate Binding Studies S89

Receptor **7** Carbohydrate Binding Studies S109

Receptor **8** Carbohydrate Binding Studies S111

Receptor **9** Carbohydrate Binding Studies S127

Comparison of Receptor Sensitivities Towards Glucose S134

5. *Molecular Modeling* S135

References S138

1. Structures of Receptors and Carbohydrates

Receptors

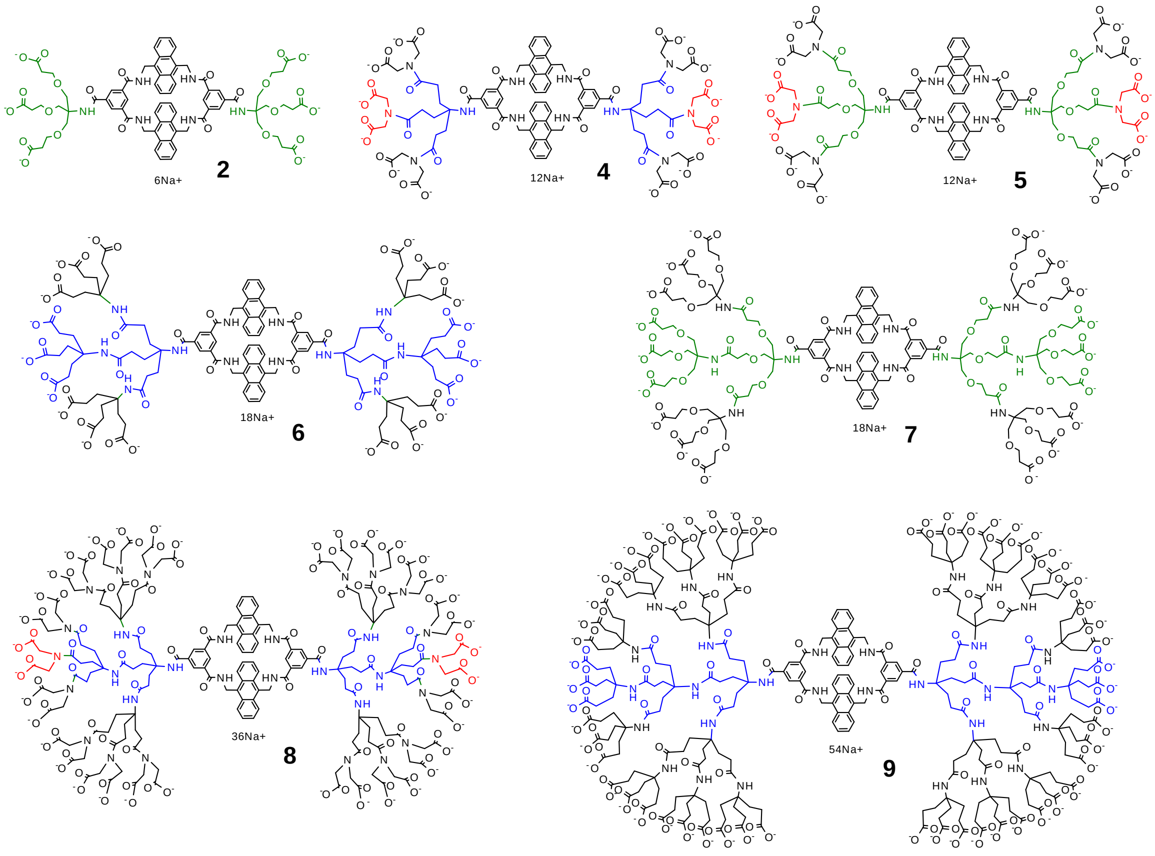

Carbohydrates

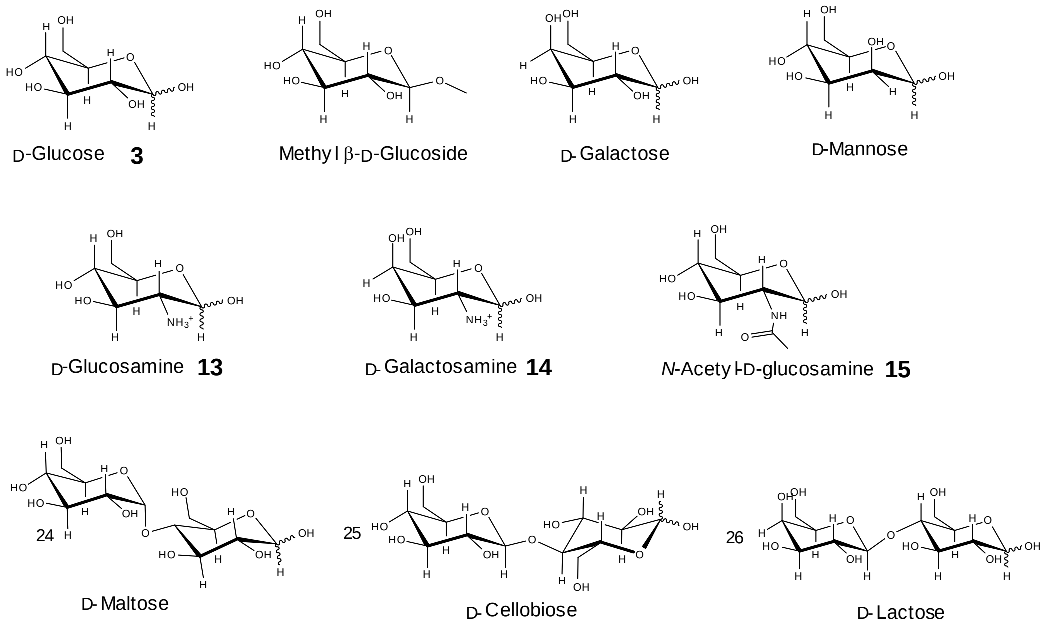

Figure S1. Receptors and carbohydrates used in Binding Studies.

2. Synthesis of Receptors

General methods

^{1}H and ^{13}C NMR spectra were recorded at 400 MHz on a Varian 400-VJ4 spectrometer, at 500 MHz on a Varian System 500-A spectrometer, at 500 MHz on a Varian System 500-B spectrometer, at 600 MHz on a Varian *INOVA* 600 spectrometer, or at 600 MHz on a Varian VNMRS 600 spectrometer equipped with a cryoprobe. Chemical shifts were reported in ppm and referenced to residual solvent peaks. Mass spectra; were recorded on a Bruker Apex 4e 7.0T FT-MS (HR-ESI), a Bruker micrOTOF (HR-ESI), a VG Analytical Quattro (ESI) or on a VG Analytical Autospec (EI). Fluorescent titrations were carried out using a PerkinElmer LS 45 spectrometer. Isothermal Titration Microcalorimetry (ITC) titrations were performed on MicroCal Auto-iTC200. All compounds that were commercially available were purchased from Aldrich, Alfa-Aesar, Sigma, Carbosynth or Frontier Scientific. Precoated silica gel (Merck silica gel 60 F_{254}) Thin Layer Chromatography (TLC) was used for the monitoring of reactions. The spots were made visible using UV light (254, 360 nm) or with potassium permanganate, bromocresol green, ninhydrine or a ethanolic solution of phosphomolybdic acid. These conditions were used for reporting R_f values. Flash column chromatography was performed using silica gel (fisher brand silica 60 Ấ particle size 35-70 micron) as the stationary phase.

Synthesis of Receptor 4

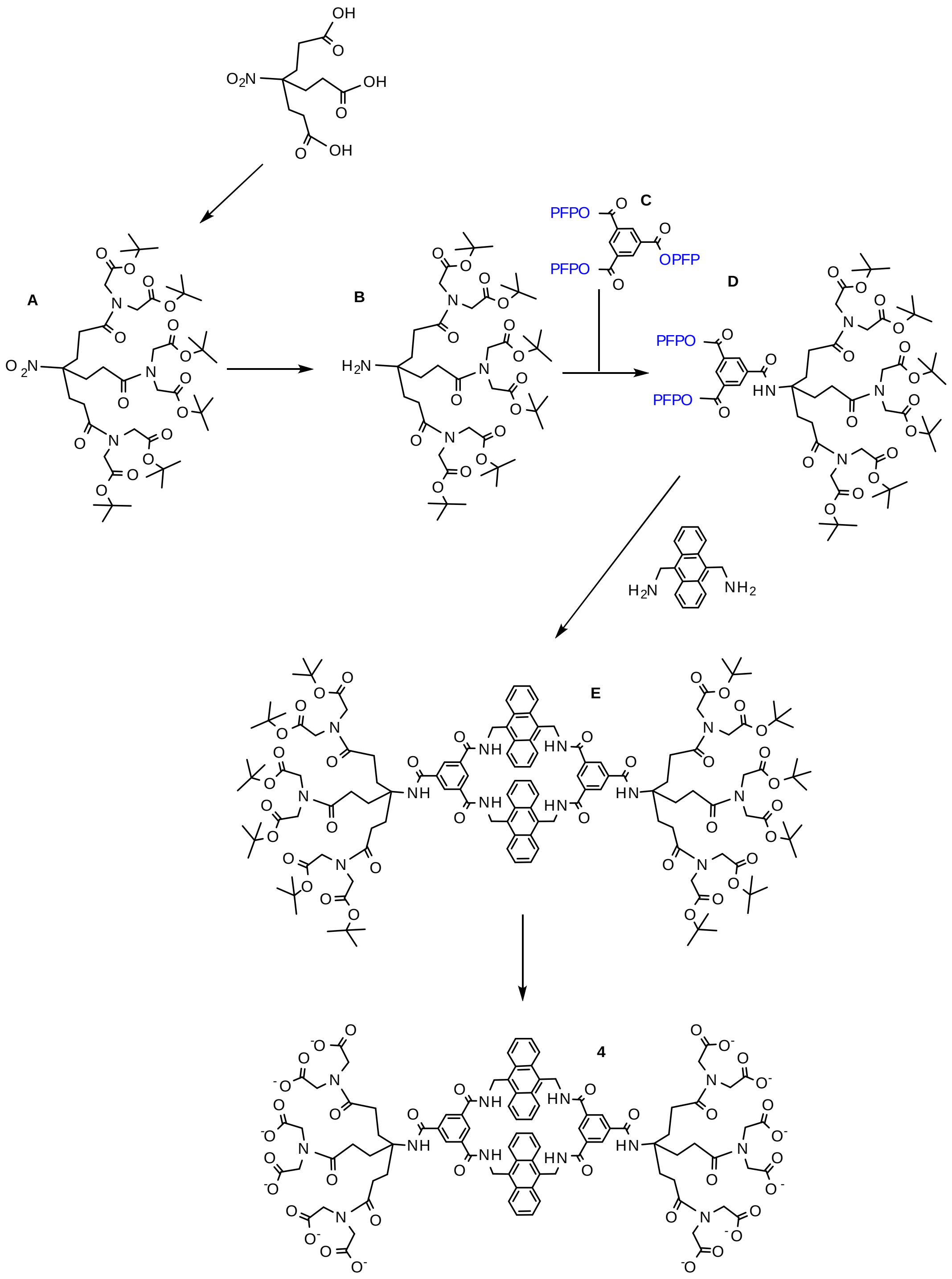

Figure S2. Synthesis of receptor **4**.

Experimental details

Nitrohexa-ester A

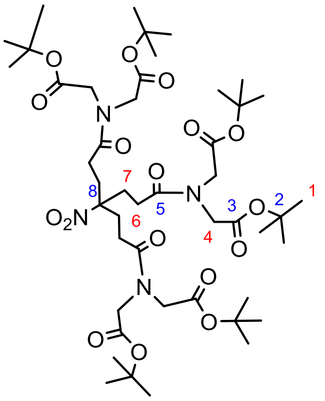

Nitromethanetrispropionic acid[1] (1.80 g, 6.50 mmol), HOBt (3.10 g, 20.2 mmol) and di-tert-butyl iminodiacetate (5.00 g, 20.4 mmol) were dissolved in THF (20 mL, anhydrous) over molecular sieves (4 A) in a flame-dried flask. A solution of DCC (4.50 g, 21.8 mmol) in THF (5 mL, anhydrous) with DIPEA (1 mL) was added and the reaction left stirring for 40 hr at RT under N_2. The solvent and DIPEA were removed under vacuum and the residue purified via flash column chromatography (hexane/EtOAc, 7:3 to 1:1) to yield nitro-hexa-ester **A** (5.0 g, 80 %). R_f = 0.40 (hexane/EtOAc, 3:2). ^{1}H NMR (500 MHz, $CDCl_3$) δ = 1.44 (s, 27H, H1), 1.46 (s, 27H, H1), 2.28 (s, 12H, H7/6), 3.97 (s, 6H, H4), 4.02 (s, 6H, H4). ^{13}C (125 MHz, $CDCl_3$) δ = 28.14 (C1), 28.21 (C1), 31.01 (C4), 49.05 C6/7, 51.00 C6/7, 81.97 (C2), 83.04 (C2), 92.72 (C8), 167.96 (C5/3), 168.33 (C5/3), 171.63 (C3/5). HRMS (ESI): m/z calculated for $C_{46}H_{78}N_4O_{17}Na$ $[M + Na^+]^+$ = 981.5260, found: 981.5214.

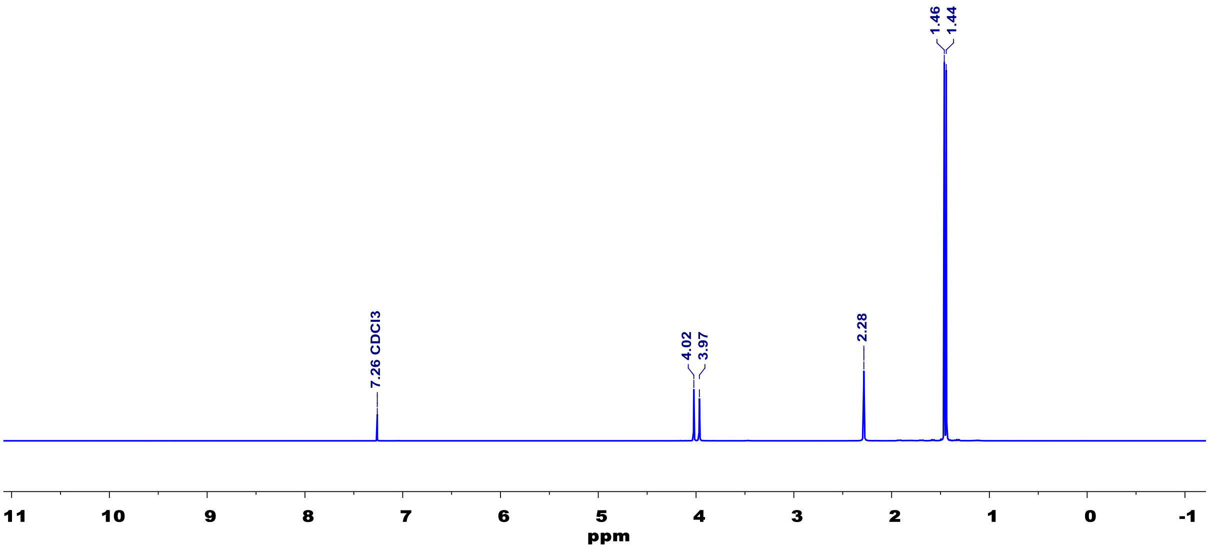

Figure S3. ^{1}H NMR (500 MHz, $CDCl_3$) spectrum of nitro-hexa-ester **A**.

Aminohexa-ester B

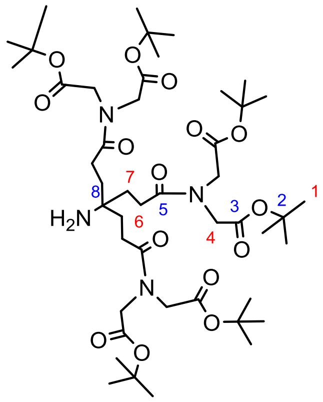

To an autoclave (250 mL) were added nitro-hexa-ester **A** (2.20 g, 2.30 mmol), Raney Ni (5 mL, water slurry) and ethanol (50 mL). The autoclave was then sealed, pressurised with H_2 (50 bar) and left stirring for 48 h at 60 °C. The mixture was then filtered through celite and the solvent removed under reduced pressure to yield amino-hexa-ester **B** (2.10 g, 99%). R_f = 0.30 (DCM/MeOH, 95:5). ^{1}H NMR (500 MHz, $CDCl_3$) δ = 1.44 (s, 27H, H1), 1.47 (s, 27H, H1), 1.81 (s, 6H, H7), 2.42 (s, 6H, H6) 4.02 (s, 6H, H4), 4.05 (s, 6H, H4). ^{13}C (125 MHz, $CDCl_3$) δ = 27.11 (C6), 28.18 (C1), 28.29 (C1), 34.05 (C7), 49.26 (C4), 51.33 (C4), 81.97 (C2), 82.92 (C2), 101.12 (C8), 168.21 (C3), 168.29 (C3), 173.65 (C5). HRMS (ESI): *m/z* calculated for $C_{46}H_{81}N_4O_{15}$ $[M + H]^+$ = 929.5693, found: 929.5679.

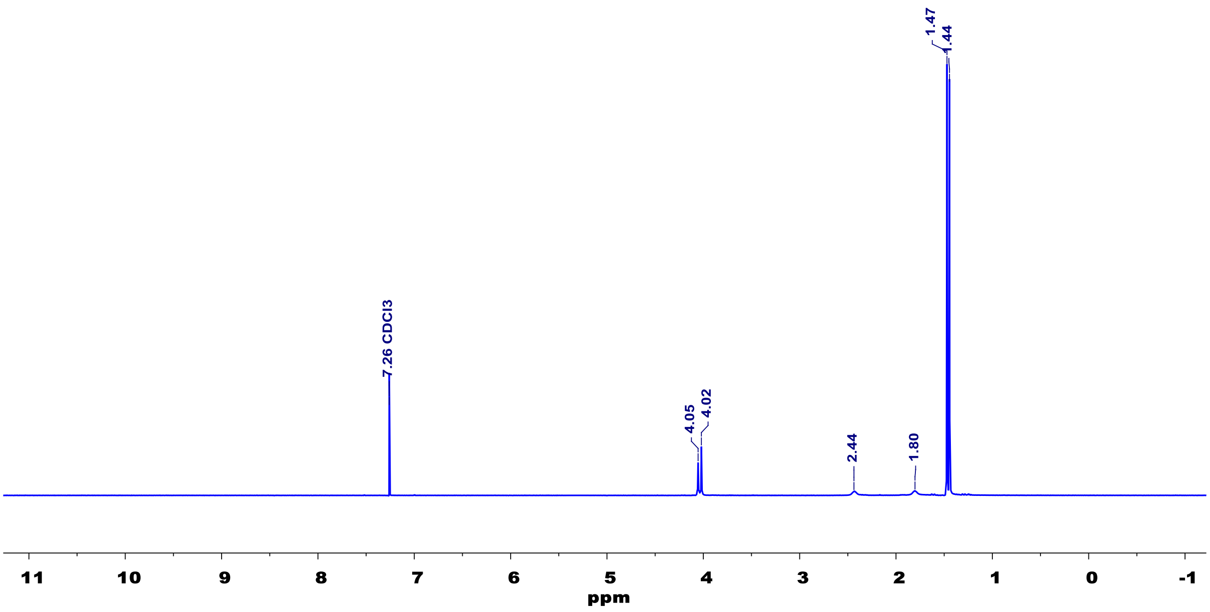

Figure S4. ^{1}H NMR (500 MHz, $CDCl_3$) spectrum of amino-hexa-ester **B**.

Tris-pentafluorophenyl ester C

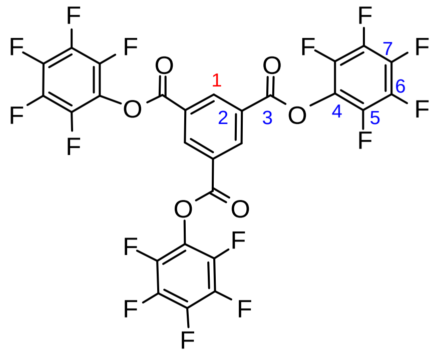

To a solution of pentafluorophenol (9.19 g, 50.0 mmol) and DCC (8.84 g, 42.8 mmol) in THF (100 mL, anhydrous) was added a solution of trimesic acid (3.00 g, 14.28 mmol) in THF (150 mL, anhydrous). The mixture was left stirring under N_2 for 36 hr. The solvent was removed under vacuum and the remaining solid dispersed into DCM (250 mL), filtered and evaporated. The residue was purified via column chromatography (DCM/hexane, 35:65) which yielded the tris-pentafluorophenyl ester **C**[2] as a white solid (9.0 g, 89 %). R_f = 0.40 (DCM/hexane, 65:35). ^{1}H NMR (400 MHz, $CDCl_3$) δ = 9.28 (s, 3H, H1). ^{13}C NMR (100 MHz, $CDCl_3$) ^{19}F NMR (370 MHz, $CDCl_3$) δ = -152.13 (d, JFF = 17.1 Hz, 6F, F5), -156.26 (t, JFF = 21.3 Hz, 3F, F7), -161.32 (t, JFF = 17.1 Hz, 6F, F6). 124.84 (t, J_{CF} = 13.5 Hz, C4), 129.62 (C2), 137.81 (C1), 138.11 (dt, J_{CF} = 250 Hz, 13.7 Hz, C5), 140.24 (dt, J_{CF} = 250 Hz, 13.2 Hz, C7), 141.3.3 (dm, J_{CF} = 254 Hz, C6).

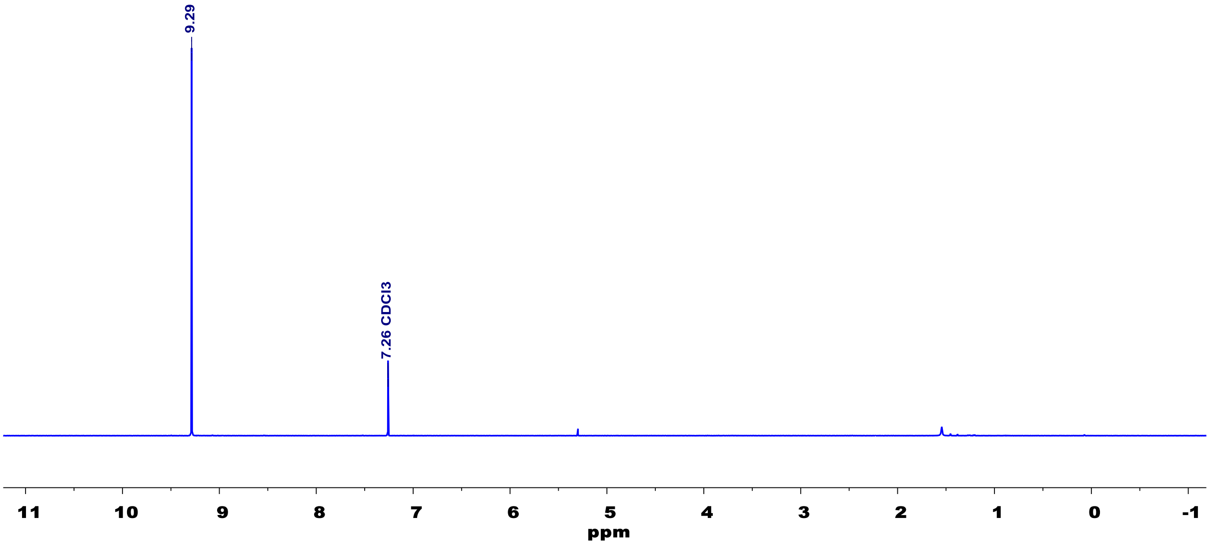

Figure S5. ^{1}H NMR (500 MHz, $CDCl_3$) spectrum of tris-pentafluorophenyl ester **C**.

Bis-pentafluorophenyl ester D

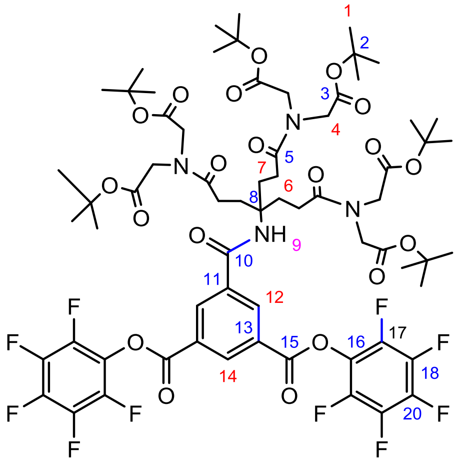

To a stirred suspension of tris-pentafluorophenyl ester **C** (1.00 g, 1.42 mmol) and amino-hexa-ester **B** (0.60 g, 0.65 mmol) in THF (5 mL, anhydrous), was added DIPEA (1 mL). The reaction mixture was heated (40 °C) for 4 h, after which the clear solution was concentrated to dryness with a rotary evaporator. The resulting oil was purified via column chromatography (hexane/EtOAc, 6:4 to 4:6) to yield bis-pentafluorophenyl ester **D** as a white solid (0.94 g, 80.8 %). R_f = 0.50 (hexane/EtOAc, 1:1). ^{1}H NMR (500 MHz, $CDCl_3$) δ = 1.39 (s, 27H, H1), 1.40 (s, 27H, H1), 2.26 (t, J_{HH} = 7.0 Hz, 6H, H7), 2.46 (t, J_{HH} = 7.0 Hz, 6H, H6), 4.02/4.04 (2s, 12H, H4), 9.03 (t, J_{HH} = 1.70 Hz, 1H, H14), 9.09 (d, J_{HH} = 1.70 Hz, 2H, H12), 9.18 (s, 1H, H9). ^{13}C (125 MHz, $CDCl_3$) δ = 27.67 (C6), 28.07 (C1), 28.11 (C1), 31.26 (C7), 49.01 (C4), 51.17 (C4), 58.35 (C8), 81.90 (C2), 82.84 (C2), 128.38 (C13), 134.59 (C14), 135.37 (C12), 137.58 (C11), 161.26 (C15), 163.34 (C10), 168.05 (C3), 168.27 (C3), 173.97 (C5). HRMS (ESI): *m/z* calculated for $C_{67}H_{82}O_{20}N_4F_{10}Na$ $[M + Na]^+$ = 1475.5255, found: 1475.5277.

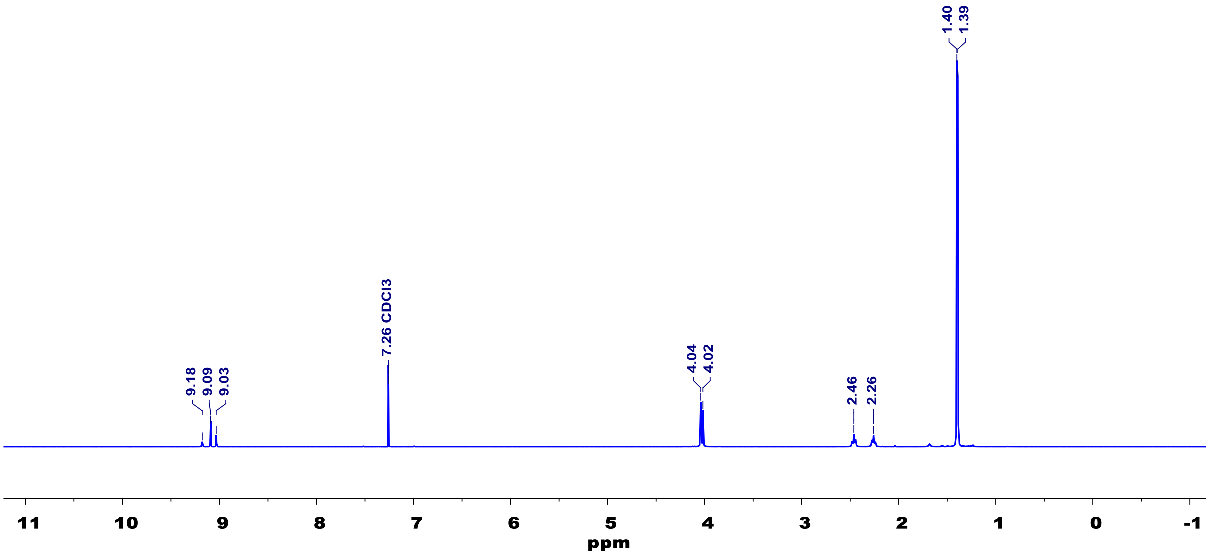

Figure S6. ^{1}H NMR (500 MHz, $CDCl_3$) spectrum of bis-pentafluorophenyl ester **D**.

Protected receptor E

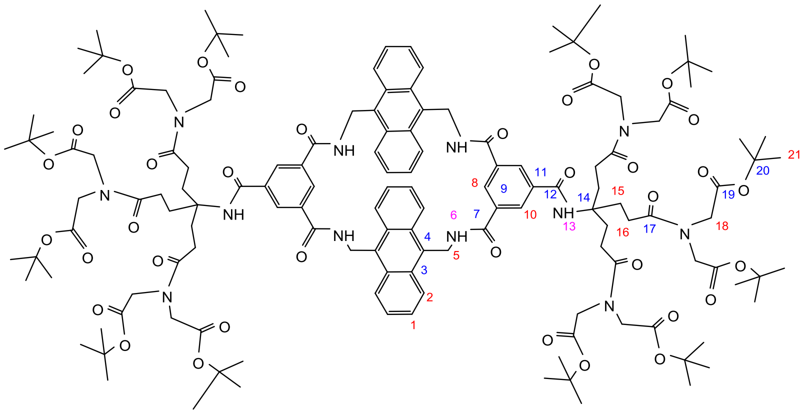

9,10-Bis-(aminomethyl)anthracene[3] (49.9 mg, 0.21 mmol) was dissolved in THF (500 mL, anhydrous) and DIPEA (2 mL, 21.93 mmol). A solution of bis-pentafluorophenyl ester **D** (307 mg, 0.21 mmol) in THF (50 mL, anhydrous) was added over 36 h using an automated syringe pump under N_2 with stirring. After the addition the reaction was left for a further 36 h. The solvent was removed under vacuum and toluene (2 x 300 mL) was added and removed on the rotary evaporator. The residue was then dissolved in acetone/water (5:1 12 mL), filtered (45 µm syringe filter) and subjected to preparative HPLC (acetone/water, 75:25 to 95:5 over 30 min). A major component absorbing at 370 nm and eluting at 12-14 min was collected and evaporated, to yield protected receptor **E** as a white solid (89.4 mg, 32 %). R_f = 0.5 (hexane/EtOAc, 3:7). ^{1}H NMR (500 MHz, $CDCl_3$) δ = 1.42 (s, 56H, H21), 2.29 (t, J_{HH} = 7.0 Hz, 12H, H15), 2.48 (t, J_{HH} = 7.0 Hz, 12H, H16), 4.08/4.14 (2s, 12H, H18), 5.53 (d, J_{HH} = 4.3 Hz, 8H, H10), 6.46 (t, J_{HH} = 4.3 Hz, 4H, H8), 7.42 (s, 2H, H8), 7.47 (dd, J_{HH} = 2.35, 6.45 Hz, 8H, H2), 8.32 (dd, J_{HH} = 2.35, 6.45 Hz, 8H, H1), 8.60 (s, 2H, H13), 8.63 (s, 4H, H10). ^{13}C NMR (125 MHz, $CDCl_3$) δ = 27.62 (C16), 28.00 (C21), 30.98 (C15), 37.15 (C5), 49.03 (C18), 58.16 (C14), 82.35 (C20), 124.62 (C1), 125.23 (C8), 126.39 (C2), 129.92 (C10), 129.96 (C3), 130.01 (C4), 165.83 (C7), 166.76 (C12), 168.13 (C19), 173.83 (C17). HRMS (ESI): *m/z* calculated for $C_{142}H_{192}N_{12}O_{36}Na_2$ $[M + 2 Na]^{2+}$ = 1343.6683, found: 1343.6671.

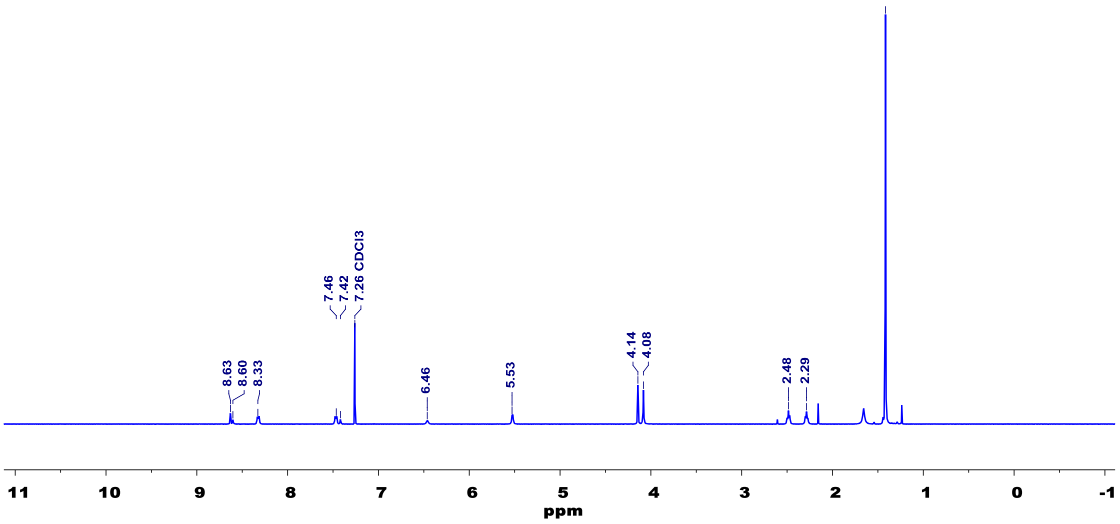

Figure S7. ^{1}H NMR (500 MHz, $CDCl_3$) spectrum of protected receptor **E**.

Receptor 4

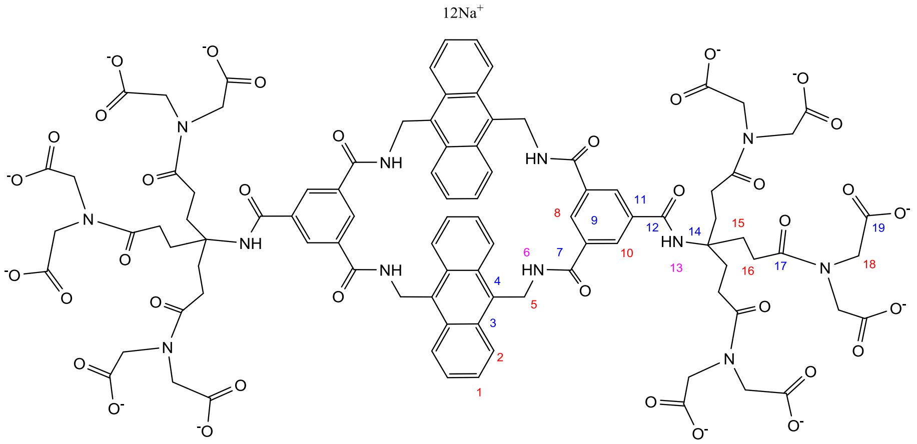

Protected receptor **E** (83.2 mg, 31.7 µmol) and triethylsilane (109 mg, 0.96 mmol) were dissolved in DCM (3 mL) and cooled to 0 °C over ice. TFA (2 mL) was added drop wise over 5 minutes and the reaction left for 6 h at RT under N_2. The solvent was removed under vacuum and the residue dissolved in water/MeOH (6:4, 10 mL) and NaOH (0.1 M) added dropwise until pH = 7. The solution was passed through a 45 µm syringe filter and freeze-dried to yield receptor **4** as a white solid (70 mg, 99%). ^{1}H NMR (500 MHz, D_2O) δ = 2.24 (t, J_{HH} = 6.5 Hz, 12H, H15), 2.50 (t, J_{HH} = 6.5 Hz, 12H, H16), 3.95/4.04 (2s, 24H, H18), 7.57 (dd, J_{HH} = 1.70/7.00 Hz, 8H, H2), 7.90 (s, 2H, H8), 8.33 (dd, J_{HH} = 1.70/7.00 Hz, 8H, H1), 8.5 (s, 4H, H10). HRMS (ESI): *m/z* calculated for $C_{94}H_{94}O_{36}N_{12}$ [M + 10 H]$^{2-}$ = 983.2952, found: 983.2981.

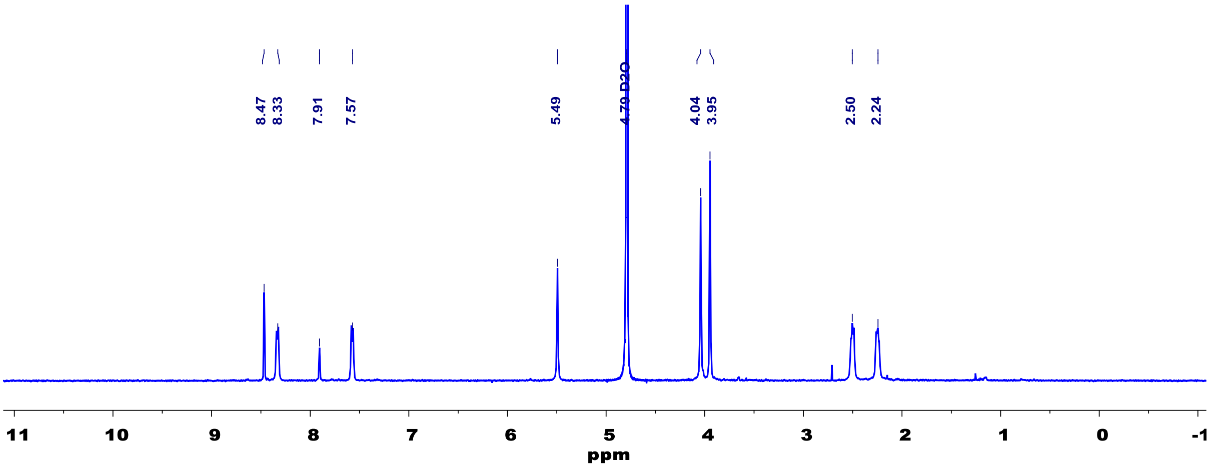

Figure S8. ^{1}H NMR (500 MHz, D_2O) spectrum of receptor **4**.

Synthesis of Receptor 5

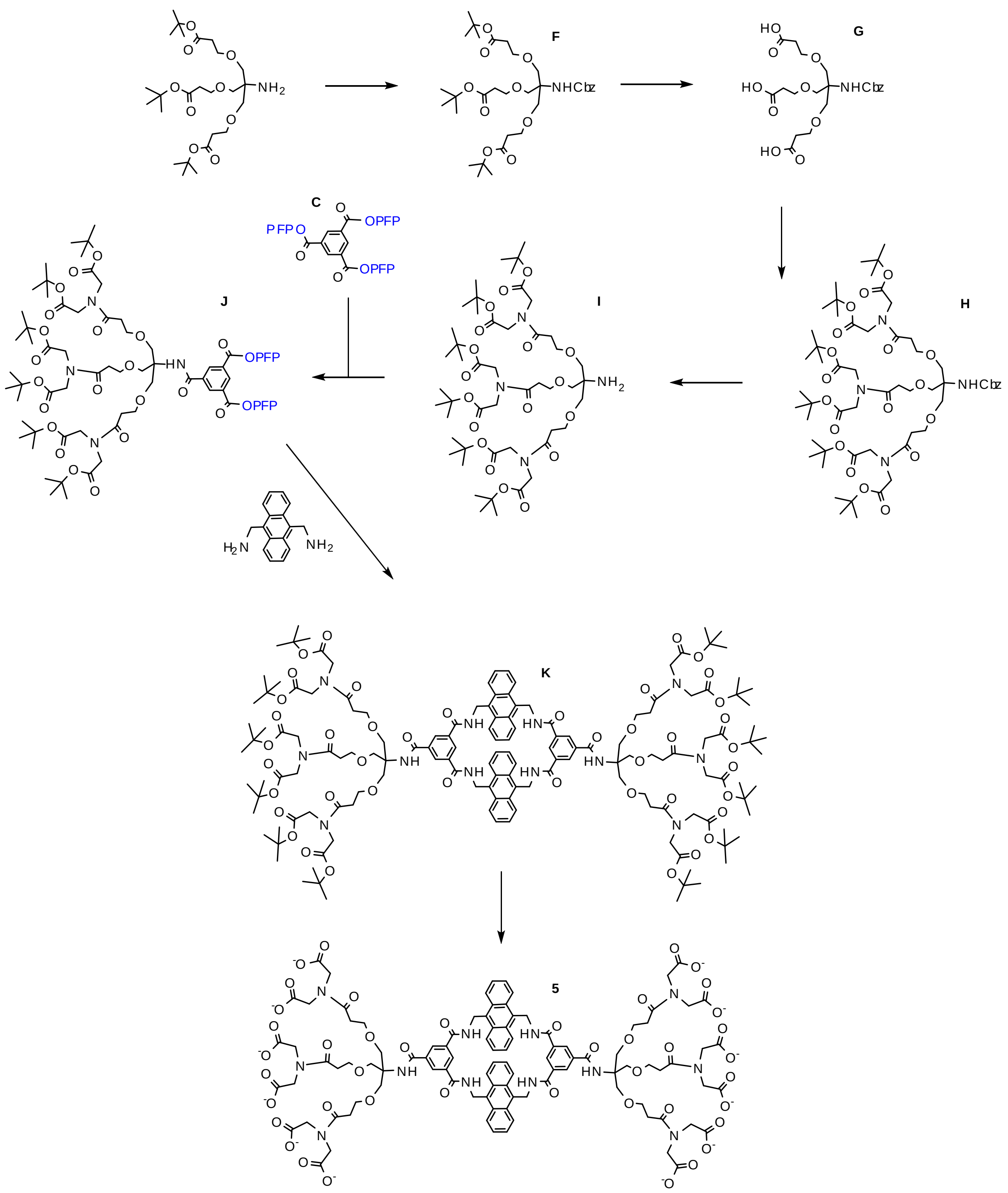

Figure S9. Synthesis of Receptor **5**.

Experimental details

Cbz-triester F

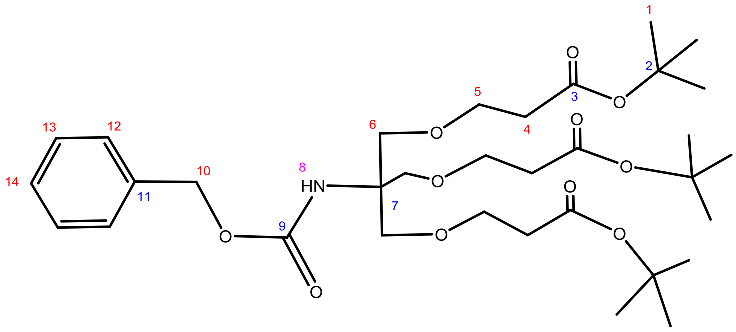

This method is based on that reported by Cardona etal.,[5] To a solution of tris{[2-tert-butoxycarbonyl)ethoxy]methyl}methylamine[4] (2.00 g, 3.95 mmol) in DCM (30 mL) was added a solution of NaHCO (aq, 25% w/v). The mixture was stirred for 5 minutes under N_2, after which benzyl chloroformate (12.4 mL, 28.3 mmol) was added dropwise over 5 minutes. The reaction was stirred under N_2 at RT for 70 h then the mixture was extracted with DCM (3 x 30 mL). The extract was dried over $MgSO_4$, filtered and concentrated under vacuum. The residue was purified by column chromatography (hexane/EtOAc, 7:3) to obtain Cbz-triester **F** as a colourless oil (1.6 g, 64 %). R_f = 0.60 (hexane:EtOAc 7:3). ^{1}H NMR (400 MHz, $CDCl_3$) δ = 1.43 (s, 27H, H1), 2.43 (t, J_{HH} = 6.3 Hz, 6H, H4), 3.63 (t, J_{HH} = 6.3 Hz, 6H, H5), 3.65 (s, 6H, H6), 5.02 (s, 2H, H10), 5.30 (s, 1H, H8), 7.28-7.34 (m, 5H, H12/13/14). [lit.[5] (300 Mhz, $CDCl_3$) δ = 1.43 (s, 27H), 2.44 (t, J_{HH} = 6.4 Hz, 6H), 3.66 (s, 6H), 5.03 (s, 2H), 5.30 (s, 1H), 7.27-7.40 (m, 5H)]. ^{13}C NMR (400 MHz, $CDCl_3$) δ = 28.21 (C1), 36.34 (C4), 58.84 (C7), 66.23 (C10), 67.20 (C5), 69.49 (C6), 80.60 (C2), 128.01/128.10/128.52 C14/13/12, 136.86 (C11), 155.23 (C9), 170.97 C3. MS (ESI) *m/z* calculated for $C_{33}H_{53}NO_{38}Na$ $[M + Na]^+$ = 662.35, found: 662.35.

Cbz-tri-acid G

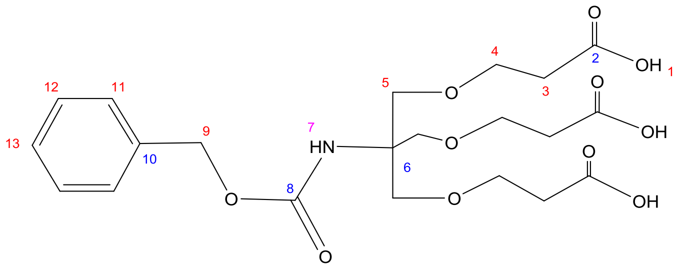

This method is based on that reported by Cardona etal.,[5] Formic acid (30 mL) was added to Cbz-tri-acid **F** (1.60 g, 2.50 mmol) and the mixture left stirring under N_2 for 6 h at RT. The volatiles were then removed under reduced pressure to yield Cbz-tri-acid **G** as a clear oil (1.17g, 100%). ^{1}H NMR (400 MHz, $CDCl_3$) δ = 2.55 (t, J_{HH} = 6.1 Hz, 6H, H3), 3.65 (s, 12H, H4/5), 5.03 (s, 2H, H9), 5.32 (s, 1H, H7), 7.25-7.34 (m, 5H, H11/12/13). [lit.[5] (300 MHz, CD_3COD_3) δ = 2.42 (t, J_{HH} = 6.3 Hz, 6H), 3.67 (s, 6H), 5.01 (s, 2H), 5.75 (s, 1H), 7.27-7.35 (m, 5H)]. ^{13}C NMR (400 MHz, $CDCl_3$) δ = 34.85 (C3), 58.95 (C6), 66.60 C4/9, 69.67 (C5), 128.18/128.21/128.64/136.51 C10/11/12/13, 155.42 (C8), 177.40 (C2), 177.40 (C11), 155.23 (C9), 170.97 (C3). MS (ESI) m/z calculated for $C_{21}H_{29}NO_{11}Na$ $[M + Na]^+$ = 464.16, found: 464.16.

Cbz-hexa-ester H

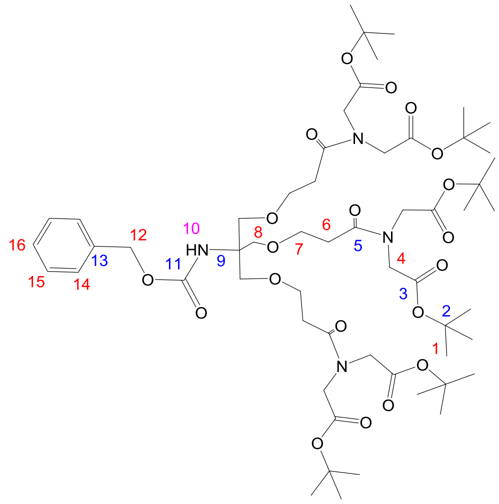

HOBT (57.0 mg, 0.42 mmol), EDCI (290 mg, 1.53 mmol) and Cbz-triacid **G** (200 mg, 0.42 mmol) were dissolved in THF (2 mL, anhydrous) and TEA (0.34 mL, 2.55 mmol) was added. A solution of Di-tert-butyl iminodiacetate (416 mg, 1.7 mmol) in THF (2 mL, anydrous) was added under N_2 at RT with stirring. After 24 h the solvent was removed under vacuum and the residue dissolved in $CHCl_3$ (20 mL), washed with NH_4Cl (sat. aq., 20 mL) and brine (20 mL), dried over $MgSO_4$, filtered and concentrated under vacuum. The residue was purified via column chromatography (hexane/EtOAc, 52:58) to yield Cbz-hexa-ester **H** as a white foam (286 mg, 60%). R_f = 0.4 (hexane/EtOAc, 1:1). ^{1}H NMR (400 MHz, $CDCl_3$) δ = 1.42-46 (2s, 56H, H1), 2.53 (t, J_{HH} = 6.7 Hz, 6H, H6), 3.67 (s, 6H, H8), 3.72 (t, J_{HH} = 6.7 Hz, 6H, H7), 4.02 (s, 12H, H4), 5.02 (s, 2H, H12), 5.55 (s, 1H, H10), 7.26-7.36 (m, 5H, H14-16). ^{13}C NMR (100 MHz, $CDCl_3$) δ = 28.18 (C1), 33.35 (C6), 48.66/51.06 (C4), 59.01 (C9), 65.77 (C12), 67.66 (C7), 69.70 (C8), 81.90/82.72 (C2), 127.96 (C13), 128.11/128.51 C14/15, 137.04 (C16), 154.99 (C11), 168.27/168.49 (C3), 171.69 (C5). HRMS (ESI) *m/z* calculated for $C_{57}H_{92}N_4O_{20}Na$ $[M + Na]^+$ = 1175.6197, found: 1175.6216.

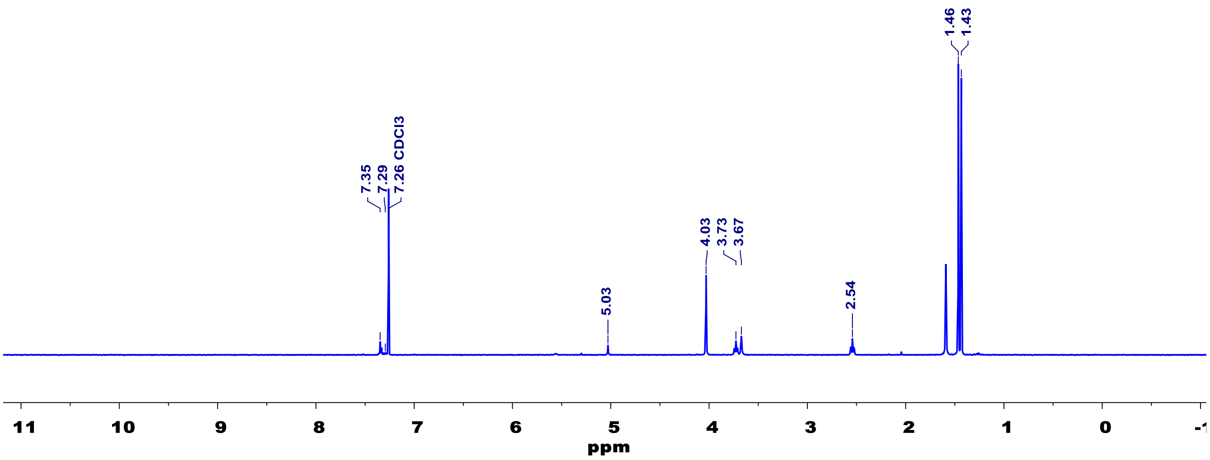

Figure S10. ^{1}H NMR (400 MHz, $CDCl_3$) spectrum of Cbz-hexa-ester **H**.

Amino-hexa-ester I

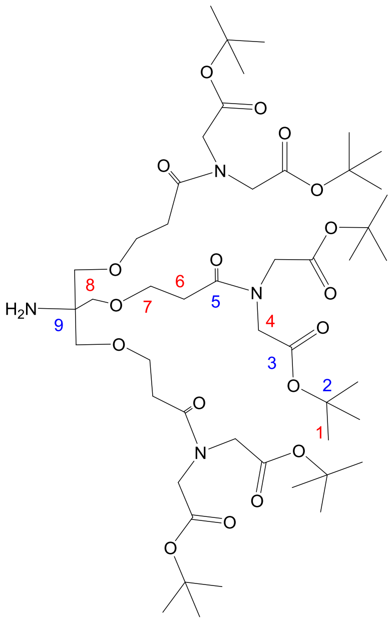

Cbz-hexa-ester **H** (200 mg, 0.17 mmol) was dissolved in EtOH (25 mL, degassed) and added under N_2 to a flask containing activated Pd/C (55 mg, 0.52 mmol). Acetic acid (5 drops) was added and the flask flushed and left under H_2 with stirring for 16 h at RT. Hexane (10 mL) was added, the mixture was filtered through celite and the solvent was removed under vacuum to yield amino-hexa-ester **I** as a clear oil (177 mg, 100%). R_f = 0.40 (DCM/MeOH, 93:7). ^{1}H NMR (500 MHz, $CDCl_3$) δ = 1.45-47 (2s, 56H, H1), 2.56 (t, J_{HH} = 5.3 Hz, 6H, H6), 3.72 (s, 6H, H8), 3.83 (t, J_{HH} = 5.3 Hz, 6H, H7), 4.05-06 (2s, 12H, H4). ^{13}C NMR (125 MHz, $CDCl_3$): δ = 28.20-28.24 (C1), 32.92 (C6), 48.91-51.23 (C4), 60.93 (C9), 61.21 (C8), 67.70 C6/7, 82.29-82.93 (C2), 168.10-168.51 (C3), 172.21 (C5). HRMS (ESI) *m/z* calculated for $C_{49}H_{86}N_4O_{18}Na$ $[M + Na]^+$ =1019.5996, found: 1019.6010.

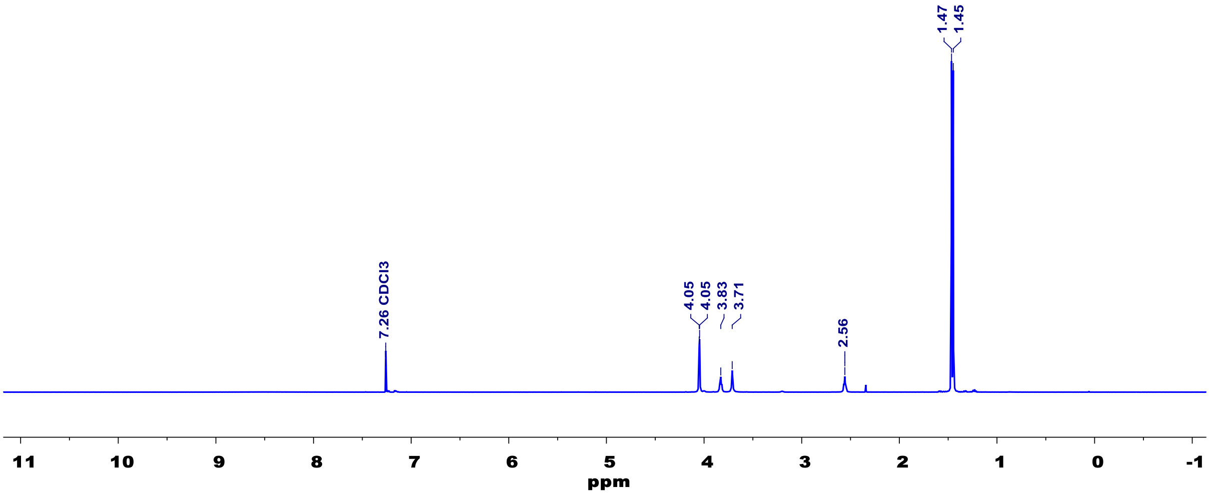

Figure S11. ^{1}H NMR (500 MHz, $CDCl_3$) spectrum of amino-hexa-ester **I**.

Bis-pentafluorophenyl ester J

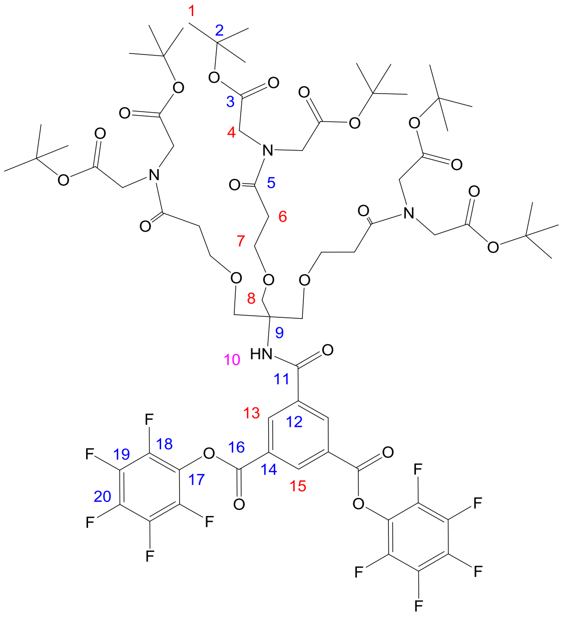

DIPEA (200 µl, 1.15 mmol) was added to a solution of **I** (186 mg, 0.18 mmol) and tris-pentafluorophenyl ester **C** (258 mg, 0.36 mmol) in THF (anhydrous, 2 mL). The reaction was heated (34 °C) and stirred under N_2 for 4 h. The solvent was removed under vacuum and the residue was dissolved in hexane/EtOAc (45:55, 1 mL), filtered and purified by flash chromatography (hexane/EtOAc = 55:45) to yield bis-pentafluorophenyl ester **J** as a colourless oil (166 mg, 60 %). R_f = 0.6 (hexane/EtOAc, 45:55). ^{1}H NMR (400 MHz, $CDCl_3$): δ = 1.38-43 (2s, 56H, H1), 2.54 (t, J_{HH} = 6.43 Hz, 6H, H6), 3.78 (t, J_{HH} = 6.43 Hz, 6H, H7), 3.86 (s, 6H, H8), 3.98-4.01 (2s, 12H, H4), 7.41 (s, 1H, H10), 9.02 (d, J_{HH} = 1.54 Hz, 2H, H13), 9.05 (t, J_{HH} = 1.54 Hz, 1H, H15). ^{13}C NMR (100 MHz, $CDCl_3$) δ = 28.80-28.72 (C1), 32.41 (C6), 48.78-51.20 (C4), 60.58 C9 61.92 (C8), 67.01 (C7), 82.48-82.87 (C2),128.14 (C14), 134.25 (C15), 135.28 (C13), 137.48 (C12), 161.47 (C15), 163.41 (C11), 167.90-168.01 (C3), 172.11 (C5). ^{19}F NMR (470 MHz, $CDCl_3$): δ = -152.14 (d, J_{FF} = 17.3 Hz, 4F, F18), -157.16 (t, J_{FF} = 20.9 Hz, 2F, F20), -161.86 (t, J_{FF} = 19.83 Hz, 4F, F19). HRMS (ESI) *m/z* calculated for $C_{70}H_{88}N_4O_{23}F_{10}Na$ $[M + Na]^+$ = 1565.5577, found: 1565.5643.

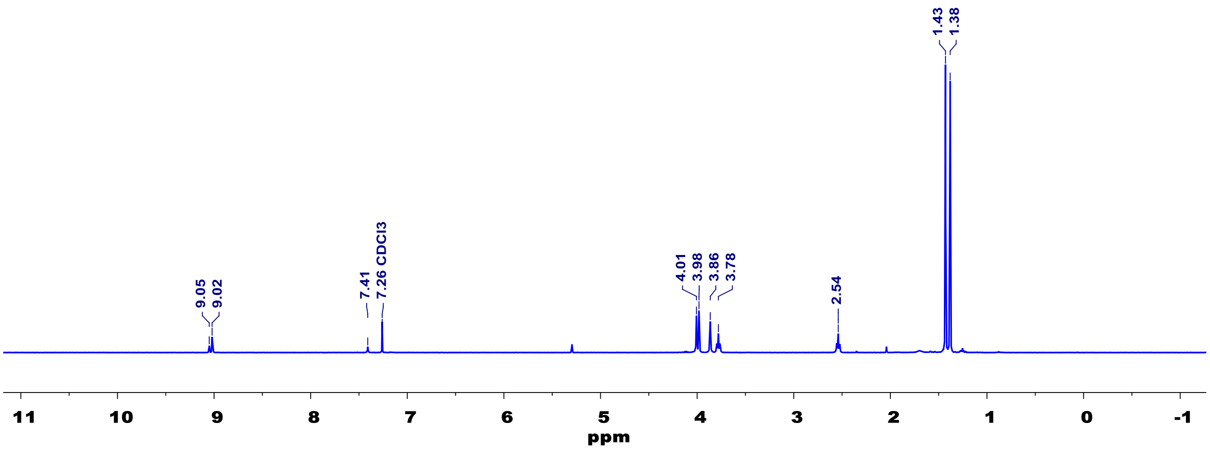

Figure S12. ^{1}H NMR (500 MHz, $CDCl_3$) spectrum of bis-pentafluorophenyl ester **J**.

Protected Receptor K

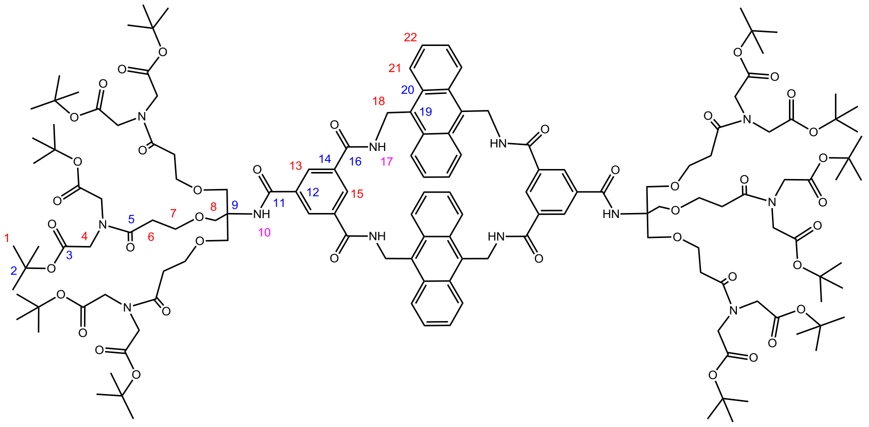

A solution of bis-pentafluorophenyl ester **J** in THF (50 mL, anhydrous) was added dropwise over 36 h (syringe pump) to a solution of 9,10-bis(aminomethyl)anthracene[3] (25.4 mg, 0.11 mmol) and DIPEA (1 mL, 6 mmol) in THF (150 mL, anhydrous) with stirring under N_2. After a further 36 h the solvent was removed under vacuum and the residue dissolved in chloroform (30 mL), washed with NH_4Cl (sat aq, 30 mL), water (30 mL), brine (30 mL), then dried over $MgSO_4$, filtered and evaporated in vacuo. The residue was dissolved in acetone/water (9:1, 6 mL) and passed through a syringe filter (0.45 µm). The solution was injected into a preparative reverse phase HPLC apparatus fitted with a reverse phase column (Waters – Xselect, 250 x 19 mm, 5µm) and eluted with acetone/water (75:25 to 85:15 over 30 min; flow rate 19 mL/min). The component eluting at 16.5 min was collected, concentrated under vacuum and freeze-dried to yield protected receptor **K** (26 mg, 17%) as a pale yellow powder. R_f = 0.5 (hexane/EtOAc, 1:4). ^{1}H NMR (500 MHz, $CDCl_3$/MeOD, 95:5) δ = 1.36-44 (2s, 102H, H1), 2.58 (t, J_{HH} = 6.3 Hz, 12H, H6), 3.74 (t, J_{HH} = 6.3 Hz, 12H, H7), 3.86 (s, 12H, H8), 4.01-08 (2s, 24H, H4), 5.42 (s, 8H, H18), 7.10 (s, 2H, H10), 7.39 (m, 8H, H22), 7.48 (s, 4H, H17), 7.58 (s, 2H, H15), 8.26 (m, 8H, H21) , 8.46 (s, 4H, H13). ^{13}C NMR (125 MHz, $CDCl_3$/MeOD, 95:5) δ = 25.07 (C1), 33.32 (C6), 48.91/51.14 (C4), 60.98 (C9), 67.60 (C7), 69.15 (C8), 82.08/82.84 (C2), 124.78 (C21), 126.06 (C15), 129.63 (C12), 130.01 (C13), 130.19 (C14), 134.35/136.81 (C20/19), 166.16 (C16), 166.91 (C11), 168.29/168.50 (C3), 172.22 (C5). HRMS (ESI) *m/z* calculated for $C_{148}H_{204}N_{12}O_{42}Na_2$ [M + 2Na]$^{2+}$ = 1433.6990, found: 1433.6987.

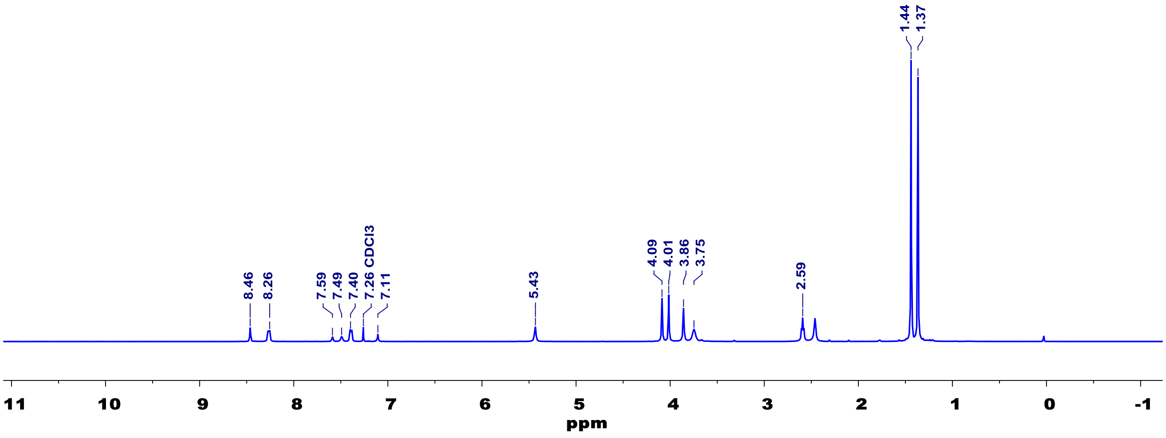

Figure S13. ^{1}H NMR (500 MHz, $CDCl_3$/MeOD, 95:5) spectrum of protected receptor **K**.

Receptor 5

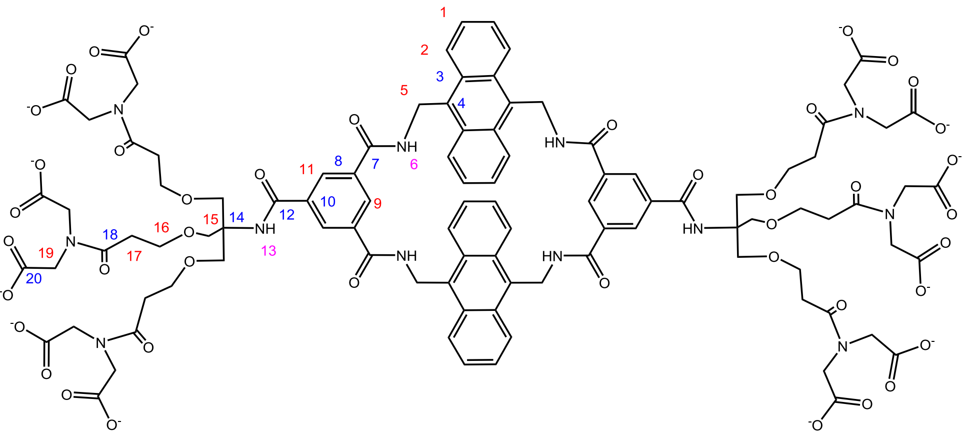

Protected receptor **K** 52 (20 mg, 7.08 µmol) was dissolved in DCM (5 mL) and cooled to 0 °C over ice. TFA (0.5 mL) was added dropwise over 5 min and the solution stirred under N_2 for 16 h at RT. The solvent was removed under vacuum and the residue dissolved in H_2O/MeOH (6:4, 10 mL) and NaOH (0.1 M) added dropwise until pH = 7. The solution was passed through a syringe filter (0.45 µm) and the remaining solution freeze-dried to obtain receptor **5** as a pale yellow powder (17 mg, 99 %). ^{1}H NMR (500 MHz, D_2O) δ = 2.65 (t, J_{HH} = 7.0 Hz, 12H, H17), 3.80 (t, J_{HH} = 7.0 Hz, 12H, H16), 3.81 (s, 12H, H15), 3.87-93 (2s, 24H, H19), 5.39 (s, 8H, H5), 7.46 (s, 8H, H2, 7.87 (s, 2H, H9), 8.38 (s, 4H, H11). 13C NMR (125 MHz, D_2O) δ = 32.83 (C17), 37.42 (C5), 53.08 (C19), 60.47 (C14), 67.45 (C15), 68.87 (C16), 67.45 (C15), 68.57 (C19), 124.48 (C1), 126.36 (C2), 127.47 (C9), 128.41/129.77 C3/4, 130.25 (C11), 131.26/134.54 C8/10, 168.26/169.56 C7/12, 173.52/175.68 C16/20.

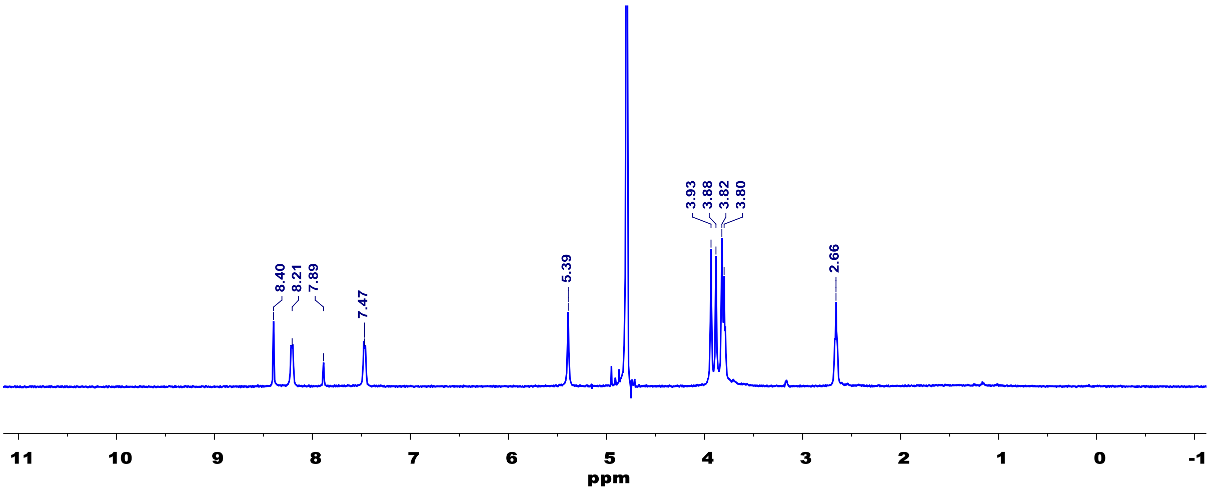

Figure S14. ^{1}H NMR (500 MHz, D_2O) spectrum of receptor **5**.

Synthesis of Receptor 6

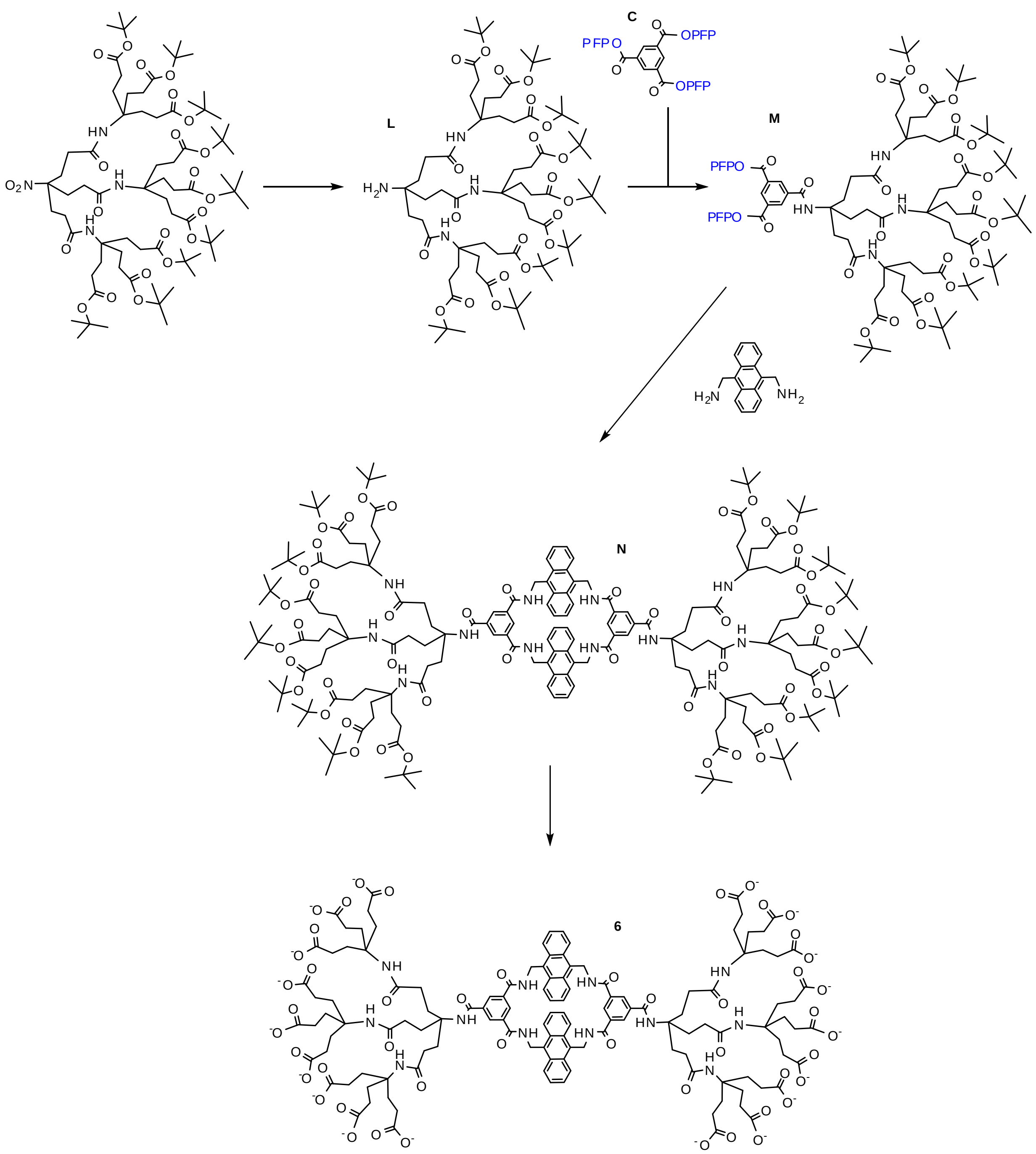

Figure S15. Synthesis of Receptor **6**.

Experimental details

Amino-nona-ester L

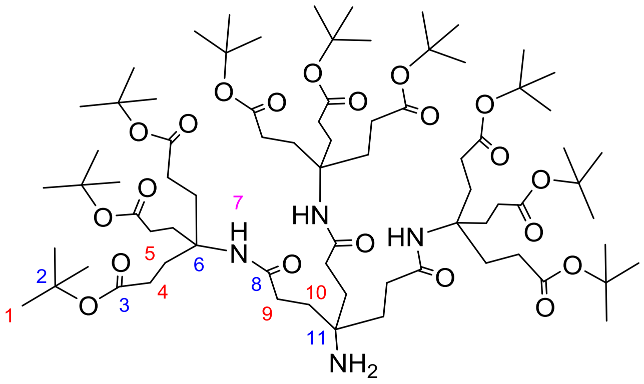

Di-tert-butyl4-[2-(tert-butoxycarbonyl)ethyl]-4-nitroheptanedicarboxylate (3.89 g, 2.65 mmol), Raney Ni (7.5 mL, water slurry) and ethanol (100 mL) were placed in an autoclave (250 mL). The autoclave was sealed, pressurised with H_2 (50 bar) and left stirring for 24 hrs at 60 °C. After cooling to RT the mixture was filtered through celite washing with DCM (50 mL). The solvent was removed under reduced pressure to yield amino-nona-ester **L** (3.46 g, 91%). ^{1}H-NMR (500 MHz, $CDCl_3$) δ = 1.42 (s, 81H, H1), 1.97 (t, 18H, H5), 2.15 (t, 6H, H10), 2.23 (m, 24H, H9/4), 2.38 (s, 2H, H12), 6.22 (s, 3H, H7) [Lit.[6] (400 MHz, $CDCl_3$) δ = 1.43 (s, 81H), 1.61 (m, 18H), 1.95 (m, 18H), 2.17 (m, 6H), 2.21 (m, 18H), 6.04 (s, 3H)]. MS (ESI) *m/z* calculated for $C_{76}H_{135}O_{21}N_4$ $[M + H]^+$ =1439.50, found 1439.96.

Bis-pentafluorophenyl ester M

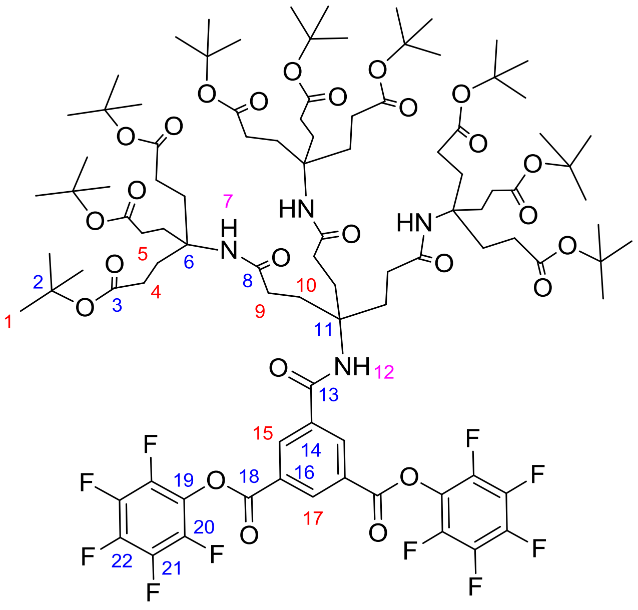

DIPEA (1.81 mL, 10.4 mmol) was added to a stirred suspension of tris-pentafluorophenyl ester **C** (1.64 g, 2.31 mmol) and amino-nona-ester **L** (1.70 g, 1.16 mmol) in a mixture of THF (10 mL, anhydrous, degassed) and DCM (2 mL, anhydrous, degassed). The reaction mixture was heated for two hours at 40 °C, after which the clear solution was concentrated to dryness with a rotary evaporator. The resulting oil was purified via column chromatography (hexane/EtOAc, 9:1 to 2:3) to yield the bis-pentafluorophenyl ester **M** as a white solid (1.68 g, 74.0 %). R_f = 0.34 (hexane/EtOAc, 3:2). ^{1}H-NMR (500 MHz, $CDCl_3$): δ 1.31 (s, 81H, H1), 1.84 (t, 18H, H5), 2.08 (m, 24H,H4/9), 2.23 (t, 6H, H10), 8.96 (t, 1H, H17), 9.15 (d, 2H, H15), 9.47 (s, 1H, H12). ^{13}C NMR (125 Mhz, $CDCl_3$) δ = 28.15 (C1), 29.86/29.91 (C4/5), 31.90 (C9/10), 57.74 (C6), 58.51 (C11), 80.75 (C2), 134.95 (C14), 135.29 (C16), 161.34 (C18), 163.57 (C13), 172.71 (C3), 172.91 (C8). ^{19}F-NMR (500 MHz, $CDCl_3$) δ = -152.42 (d, 4F, F20), -157.75 (t, 2F, F22), -162.30 (t, 4F, F21). HRMS (ESI) *m/z* calculated for $C_{97}H_{136}F_{10}N_4O_{26}Na_2$ $[M + 2Na]^{2+}$ = 1004.4534, found: 1004.4543.

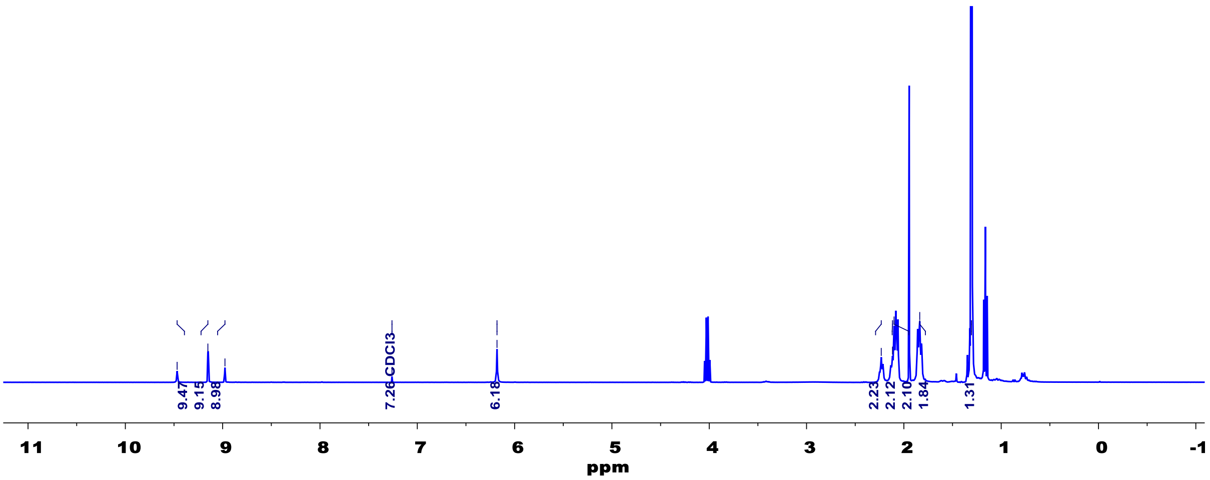

Figure S16. ^{1}H NMR (500 MHz, $CDCl_3$) spectrum of bis-pentafluorophenyl ester **M**.

Protected Receptor N

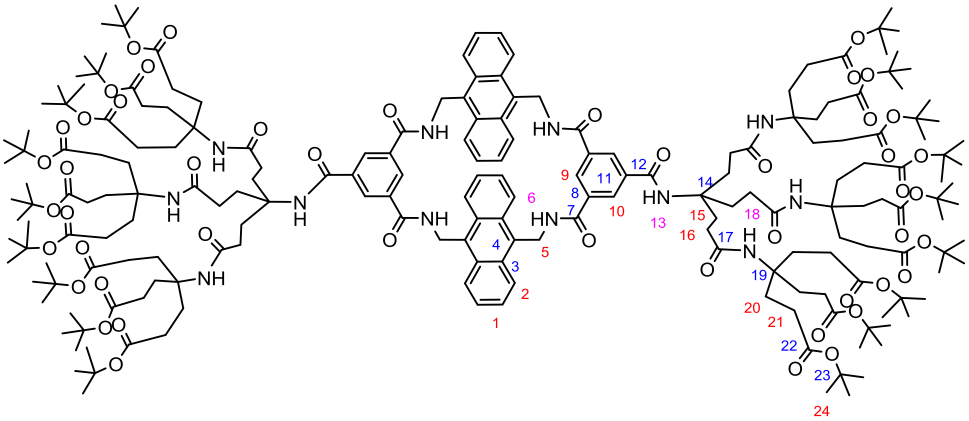

A solution of bis-pentafluorophenyl ester **M** (439 mg, 0.22 mmol) in THF (100 mL, anhydrous) was added dropwise over 36 h (syringe pump) to a solution of 9,10-bis(aminomethyl)anthracene[3] (52.8 mg, 0.22 mmol) and DIPEA (2 mL, 12 mmol) in THF (900 mL, anhydrous) under N_2. After stirring for a further 36 h the solvent was removed under vacuum and the residue dissolved into chloroform (200 mL) and washed with NH_4Cl (sat aq, 200 mL), water (200 mL), brine (200 mL), then dried over $MgSO_4$, filtered and evaporated in vacuo. The residue was dissolved in acetone/water (5:2, 6 mL) and passed through a syringe filter (0.45 µm). The solution was injected into a preparative reverse phase HPLC apparatus fitted with a reverse phase column (Waters – Xselect, 250 x 19 mm, 5µm) and eluted with acetone/water (80:20 to 90:10 over 20 min; flow rate 19 mL/min). The component eluting at 19 min was collected, concentrated under vacuum and freeze-dried to yield protected receptor **N** (58 mg, 14 %) as a pale yellow powder. R_f = 0.70 (hexane/EtOAc, 3:7). ^{1}H NMR (500 MHz, $CDCl_3$) : δ = 1.42 (s, 162H, H24), 2.01 (t, J_{HH} = 7.3 Hz, 36H, H20), 2.22 (t, J_{HH} = 7.0 Hz, 12H, H15), 2.24 (t, J_{HH} = 7.3 Hz, 36H, H21), 2.32 (t, J_{HH} = 7.0 Hz, 12H, H16), 5.53 (d, J_{HH} = 4.9 Hz 8H, H5), 6.18 (s, 6H, H18), 6.60 (t, J_{HH} = 4.9 Hz, 4H, H6), 7.38 (t, J_{HH} = 1.3 Hz, 2H, H9), 7.45 (dd, J_{HH} = 6.9, 3.3 Hz 8H, H1), 8.32 (dd, J_{HH} = 6.9, 3.3 Hz 8H, H2), 8.73 (s, 4H, H10), 8.81 (s, 2H, H13). ^{13}C NMR (125 MHz, $CDCl_3$) δ = 28.22 (C24), 29.99 (C20), 30.03 (C21), 31.92/31.98 C15/16, 37.49 (C5), 57.88 (C19), 58.56 (C14), 80.78 (C23), 124.90 (C2/9), 126.44 (C1), 129.85/130.27 (C3/4), 130.35 (C10), 165.55 (C12), 166.21 (C7), 172.87 (C23), 173.22 (C17). MS (ESI) *m/z* calculated for $C_{202}H_{300}O_{48}N_{12}Na_2$ $[M + 2Na]^{2+}$ = 1855.28, found 1855.14.

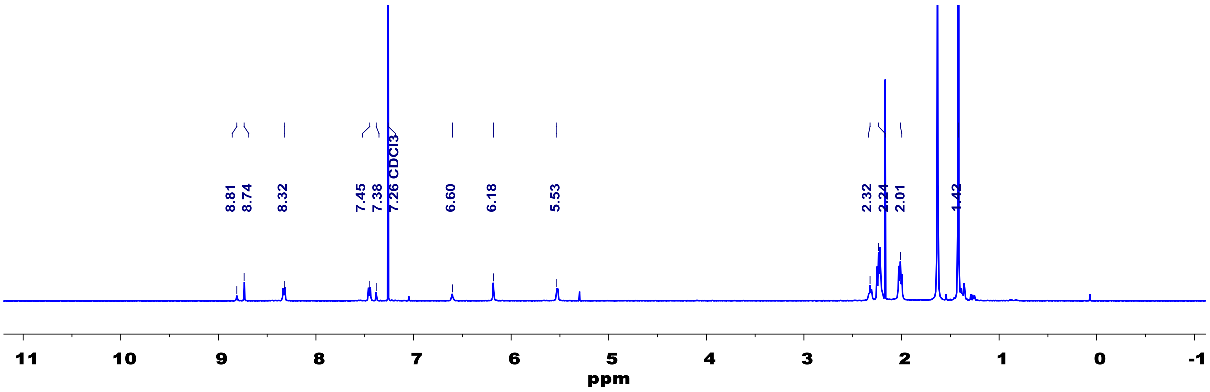

Figure S17. ^{1}H NMR (500 MHz, $CDCl_3$) spectrum of protected receptor **N**.

Receptor 6

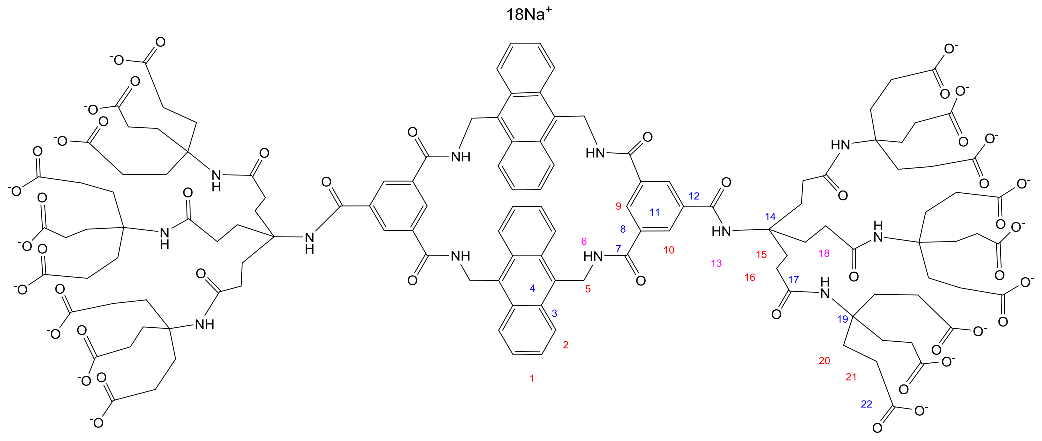

Protected receptor **N** (54.4 mg, 14.8 µmol) was dissolved in DCM (6 mL) and cooled to 0 °C over ice. TFA (2 mL) was added dropwise over 5 min and the solution stirred under N_2 for 16 hrs at RT. The solvent was then removed under vacuum and the residue dissolved in H_2O/MeOH (6:4, 10 mL). NaOH (0.1 M) was added dropwise until pH = 7. The solution was then passed through a syringe filter (0.45 µm) and the remaining solution freeze-dried to obtain receptor **6** as a pale yellow powder (43.7 mg, 97 %). ^{1}H NMR (500 MHz, $CDCl_3$) δ = 1.97 (t, J_{HH} = 7.4 Hz, 36H, H20), 2.20 (m, 48H, H15/21), 2.39 (t, J_{HH} = 7.5 Hz, 12H, H21), 5.48 (s, 8H, H5), 7.56 (dd, J_{HH} = 7.0, 2.6 Hz 8H, H1), 7.99 (s, 4H, H9), 8.29 (dd, J_{HH} = 7.0, 2.6 Hz 8H, H2), 8.53 (s, 4H, H10). ^{13}C NMR (125 MHz, D2O) δ = 30.32 (C20), 30.41 (C15), 30.83 (C16), 31.11 (C21), 37.21 (C5), 58.17 (C19), 58.91 (C14), 124.49 (C2), 127.25 (C1), 127.25 (C9), 128.58 (C4), 129.74 (C3), 130.14 (C10), 133.75 (C8), 135.95 (C11), 168.03/168.22 (C12/7), 175.07 (C17), 182.12 (C22).

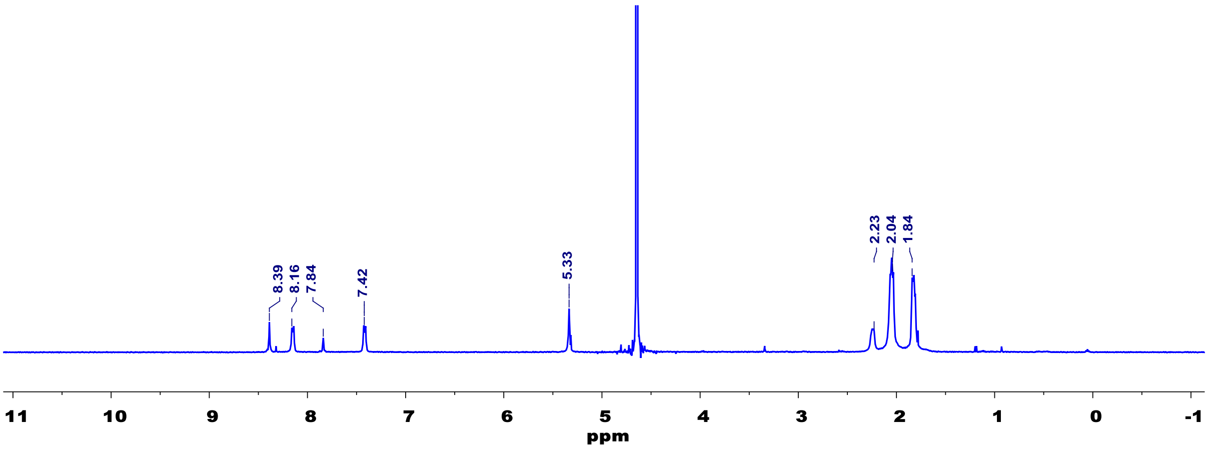

Figure S18. ^{1}H NMR (500 MHz, D_2O) spectrum of receptor **6**.

Synthesis of Receptor 7

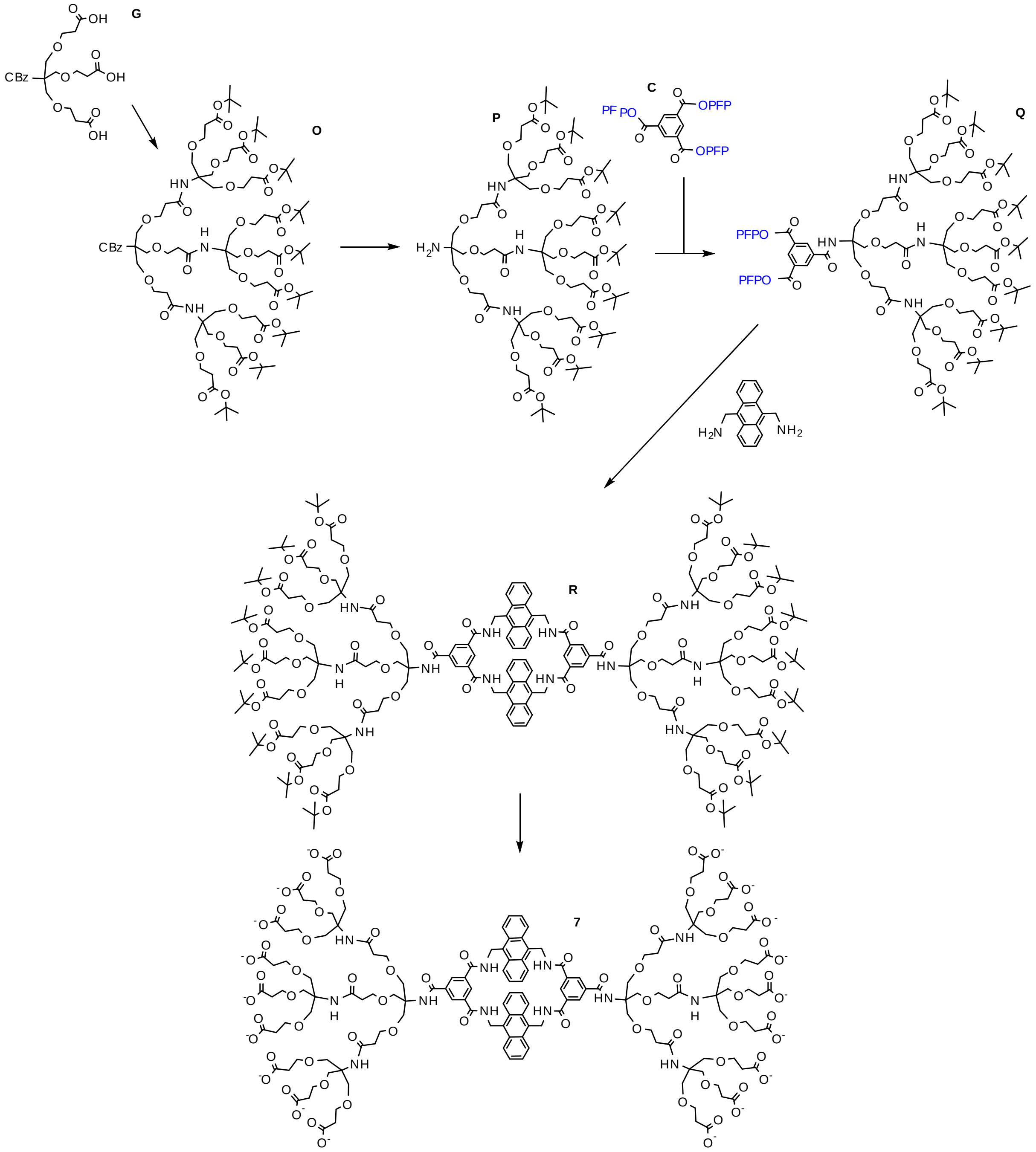

Figure S19. Synthesis of receptor **7**.

Experimental details

Cbz-nona-ester O

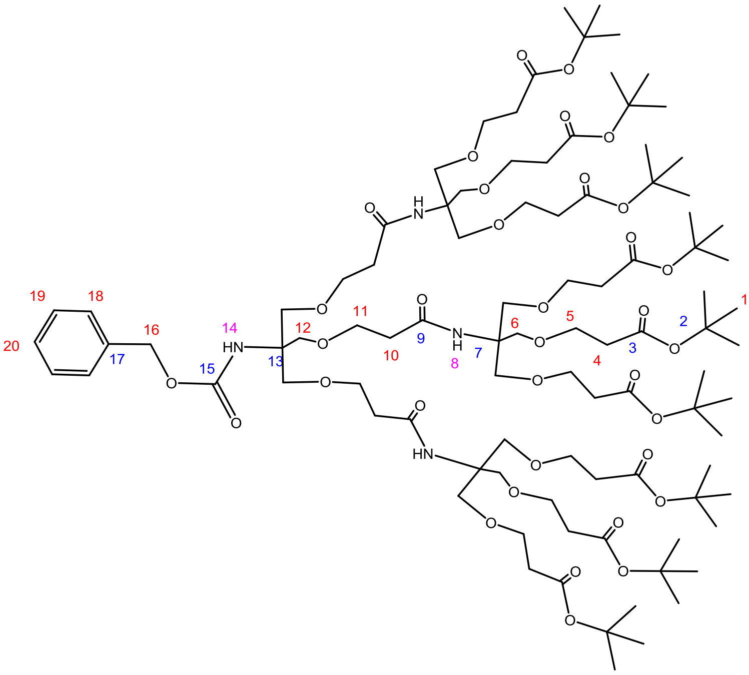

This method is based on that reported by Cardona etal.,[5] HOBt (335 mg, 2.48 mmol), TEA (2 mL, 14.9 mmol) and EDCI (1.71 g, 8.93 mmol) were added to Cbz-tri-acid **G** in THF (15 mL, anhydrous). Tris{[2-tert-butoxycarbonyl)ethoxy]methyl}methylamine[4] in THF (15 mL, anhydrous) was then added and the reaction left stirring under N_2 for 20 h at RT. The solvent was removed under reduced pressure and the residue dissolved in DCM (50 mL), washed with NH_4Cl (sat. aq., 20 mL) and brine (20 mL) before being dried over $MgSO_4$, filtered and concentrated in vacuo. The remaining residue was then purified via column chromatography (hexane/EtOAc 3:7) to yield Cbz-nona-ester **O** as a colourless oil (4.8 g, 50%). R_f = 0.50 (hexane/EtOAc, 3:7). ^{1}H NMR (500 MHz, $CDCl_3$) δ = 1.42 (s, 81H, H1), 2.37 (t, J_{HH} = 6.7 Hz, 6H, H10), 2.41 (t, J_{HH} = 6.3 Hz, 18H, H4), 3.60 (t, J_{HH} = 6.3 Hz, 18H, H5), 3.63 (m, 12H, H12/11), 3.66 (s, 18H, H6), 5.01 (s, 2H, H16), 5.53 (s, 1H, H14), 6.16 (s, 3H, H8) 7.27-7.33 (m, 5H, H18/19/20). [lit.[5] (300 Mhz, $CHCl_3$) δ = 1.44 (s, 81H), 2.40 (t, J_{HH} = 6.9 Hz, 6H), 2.43 (t, J_{HH} = 6.3 Hz, 18H), 3.59 – 3.72 (m, 48H), 5.04 (s, 2H), 5.55 (s, 1H), 6.21 (s, 3H), 7.28 – 7.39 (m, 5H)]. ^{13}C NMR (125 MHz, $CDCl_3$) δ = 28.32 (C1), 36.33 (C4), 37.53 (C10), 59.05 (C13), 59.97 (C7), 66.22 (C16), 67.23 (C5), 67.76 (C11), 69.32 (C6), 69.56 (C12), 80.62 (C2), 128.06/128.20/128.59 (C18/19/20), 137.05 (C17), 155.03 (C15), 171.04 (C15), 171.07 (C3). MS (ESI) m/z calculated for $C_{96}H_{164}N_4O_{35}Na$ $[M + Na]^+$ = 1956.1, found: 1956.20.

Amino-nona-ester P

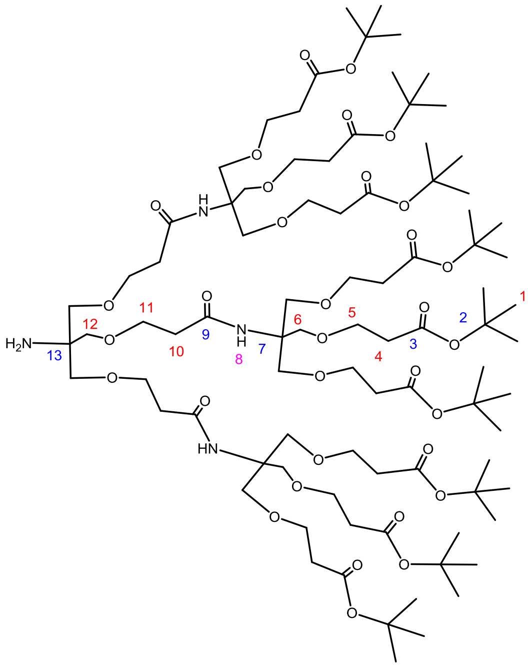

This method is based on that reported by Cardona etal.,[5] Pd/C (220 mg) was added to flask and activated by heating under vacuum for 5 min. After allowing the flask to cool to RT a solution of Cbz-nona-ester **O** (2.00 g, 1.03 mmol) in EtOH (degassed, 100 mL) was added. AcOH (1 mL) was then added and the reaction was stirred at RT under H_2 for 48 h. The mixture was filtered (celite) and evaporated to yield amino-nona-ester **P** as a clear oil (1.85 g, 99%). R_f = 0.30 (DCM/MeOH, 95:5). ^{1}H NMR (400 MHz, $CDCl_3$) δ = 1.42 (s, 81H, H1), 2.42 (t, J_{HH} = 6.7 Hz, 24H, H4/10), 3.61 (t, J_{HH} = 6.7 Hz, 24H, H5/11), 3.67 (s, 24H, H6/12), 5.28 (s, 2H, H8). [lit.[5] (300 Mhz, CD_3COCD_3) δ = 1.33 (s, 81H), 2.27 (t, J_{HH} = 6.0 Hz, 6H), 2.33 (t, J_{HH} = 6.0 Hz, 18H), 3.20 (s, 6H), 3.50 – 3.60 (m, 42H), 6.50 (s, .3H)]. MS (ESI): *m/z* calculated for $C_{88}H_{158}N_4O_{33}$ $[M + H]^+$ = 1799.08, found: 1800.08.

Bis pentafluorophenyl ester Q

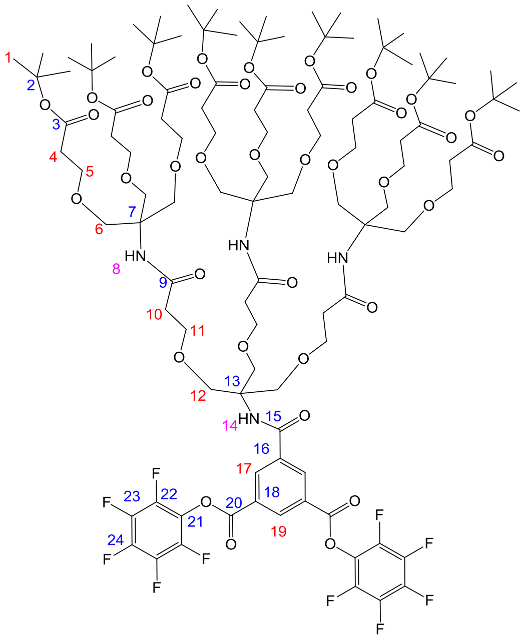

Amino-nona-ester **P** (216 mg, 0.12 mmol) and tris-pentafluorophenyl ester **C** were dissolved in THF (2.5 mL, anhydrous). DIPEA (84 µL, 0.48 mmol) was added and the reaction heated (30 °C) and left stirring under N_2 for 16 h. The solvent and DIPEA were removed under vacuum and the residue purified via column chromatography (hexane/EtOAc, 43:57) obtaining bis-pentafluorophenyl ester **Q** as a clear oil (100 mg, 36%). R_f = 0.3 (hexane:EtOAc, 3:2). ^{1}H NMR (500 MHz, $CDCl_3$) δ = 1.42 (s, 18H, H1), 2.41 (t, J_{HH} = 6.5 Hz, 18H, H4), 2.43 (t, J_{HH} = 6.7 Hz, 6H, H10), 3.57 (t, J_{HH} = 6.5 Hz, 18H, H5), 3.60 (s, 3.60, 6H, H12), 3.71 (t, J_{HH} = 6.7 Hz, 6H, H11), 3.85 (s, 6H, H11), 6.14 (s, 3H, H8), 7.48 (s, 1H, H14), 8.96 (d, J_{HH} = 1.6 Hz, 2 H, H17), 9.06 (t, J_{HH} = 6.5 Hz, 18H, H5). ^{13}C NMR (125 MHz, $CDCl_3$) δ = 28.22, 36.24 (C4), 37.21 (C10), 59.90 (C7), 61.17 (C13), 67.14 (C11/12), 67.78 (C5/6), 69.06 (C5/6), 69.16 (C11/12), 80.55 (C2), 128.23 (C18/16), 134.58 (C17), 135.77 (C19), 138.11 (C18/16), 161.15 (C20), 165.20 (C15), 170.89 (C9), 170.98 (C3). ^{19}F NMR (470 MHz, CDCl3) δ = -159.22 (d, J_{FF} = 18.0 Hz, 4F, F22), -164.26 (t, J_{FF}= 21.6 Hz, 2F, F24), -169.02 (t, J_{FF} = 18.0 Hz, 4F, F23). HRMS (ESI): m/z calculated for $C_{109}H_{160}F_{10}N_4O_{38}Na_2$ $[M + 2 Na]^{2+}$ = 1184.5167, found: 1184.5174.

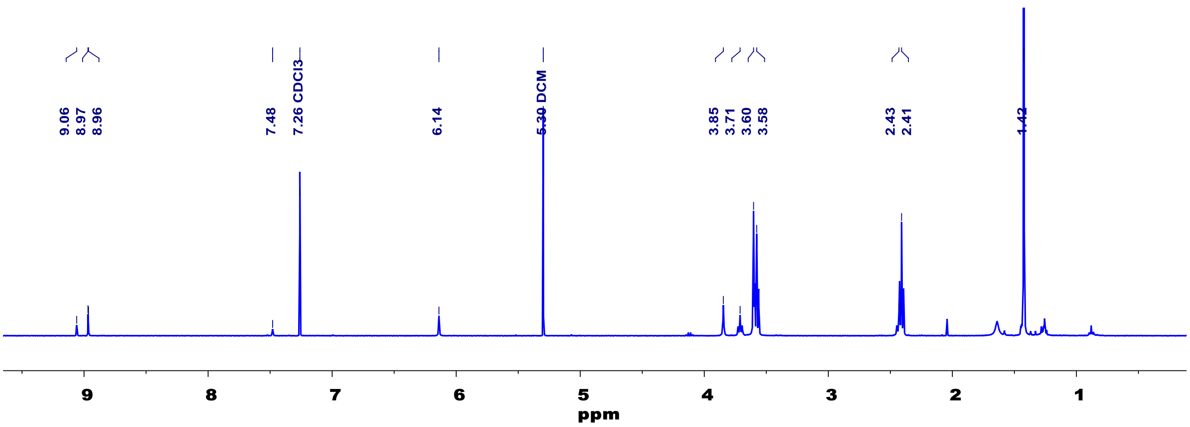

Figure S20. ^{1}H NMR (500 MHz, $CDCl_3$) spectrum of bis-pentafluorophenyl ester **Q**.

Protected receptor R

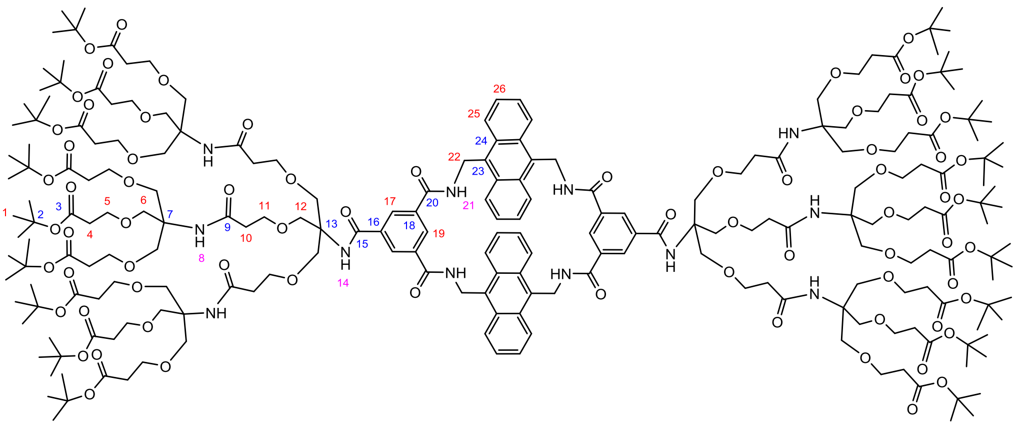

Bis-pentafluorophenyl ester **Q** (83 mg, 3.60 µmol) was dissolved in THF (80 mL, anhydrous) with DIPEA (0.1 mL, 620 µmol). A solution of 9,10-bis(aminomethyl)anthracene[3] (8.44 mg, 3.60 µmol) in THF (50 mL, anhydrous) was added over 36 h using an automated syringe pump under N_2 with stirring. After a further 36 h the solvent was removed under vacuum. The residue was dissolved in chloroform (40 mL) and washed with NH_4Cl (sat aq, 40 mL), water (40 mL), brine (240 mL), then dried over $MgSO_4$, filtered and evaporated in *vacuo*. The residue was dissolved in acetone/water (9:1, 4 mL) and passed through a syringe filter (0.45 µm). The solution was injected into a preparative reverse phase HPLC apparatus fitted with a reverse phase column (Waters – Xselect, 250 x 19 mm, 5µm) and eluted with acetone/water (85:15 to 95:5 over 35 min; flow rate 17 mL/min). The component eluting at 14 min was collected and concentrated under vacuum to yield protected receptor **R** (113 mg, 15 %) as a white solid. R_f = 0.7 (hexane:EtOAc, 3:7). ^{1}H NMR (500 MHz, $CDCl_3$/MeOD, 93:7) δ = 1.38 (s, 162H, H1), 2.38 (s, 36, H4), 2.48 (t, J_{HH} = 6.5 Hz, 12H, H10), 3.56 (s, 36H, H5), 3.60 (s, 36H, H6), 3.70 (t, J_{HH} = 6.5 Hz, 12H, H11), 3.83 (s, 36H, H12), 5.44 (s, 8H, H22), 7.36 (s, 8H, H25), 7.81 (s, 2H, H19), 8.21 (s, 8H, H26), 8.54 (s, 4H, H17). MS (ESI): *m/z* calculated for $C_{226}H_{348}N_{12}O_{72}Na_2$ [M + 2 Na]$^{2+}$ = 2214.18, found: 2215.12.

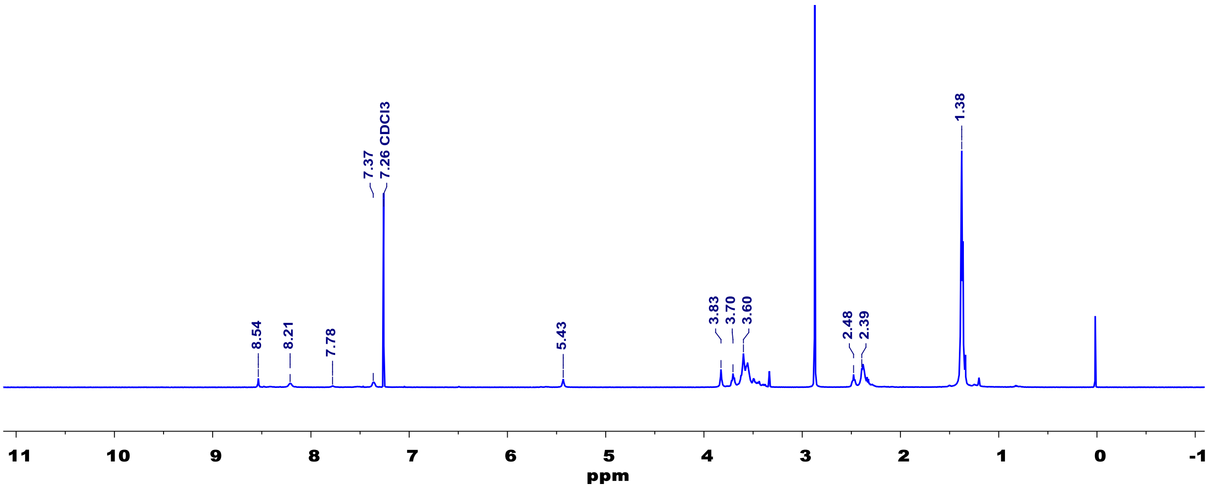

Figure S21. ^{1}H NMR (500 MHz, $CDCl_3$/MeOD, 93:7) spectrum of protected receptor **R**.

Receptor 7

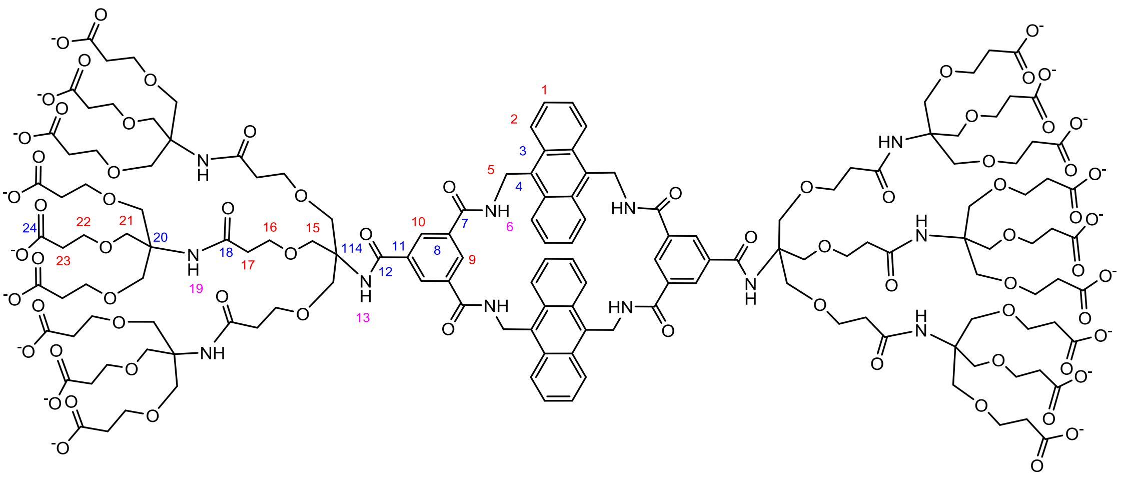

Protected receptor **R** (8.0 mg, 1.82 µmol) was dissolved in DCM (5 mL) and cooled to 0 °C over ice. TFA (0.5 mL) was added dropwise over 5 min and the solution stirred under N_2 for 2 h at RT. The solvent was then removed under vacuum and the residue dissolved in H_2O/MeOH (6:4, 10 mL) and NaOH (0.1 M) added dropwise until pH = 7. The solution was then passed through a syringe filter (0.45 µm) and the remaining solution freeze-dried to obtain receptor **7** as a pale yellow powder (6.8 mg, 99 %). ^{1}H NMR (500 MHz, D_2O) δ = 2.32 (s, 36H, H23), 2.62 (s, 36H, H17), 3.49 (s, H36, H22), 3.52 (s, H36, H21), 3.85 (s, 12H, H16), 3.95 (s, 12H, H15), 5.54 (s, 8H, H5), 7.53 (s, 8H, H2), 8.25 (s, 8H, H2), 8.56 (s, 2H, H9), 8.62 (s, 4H, H10).

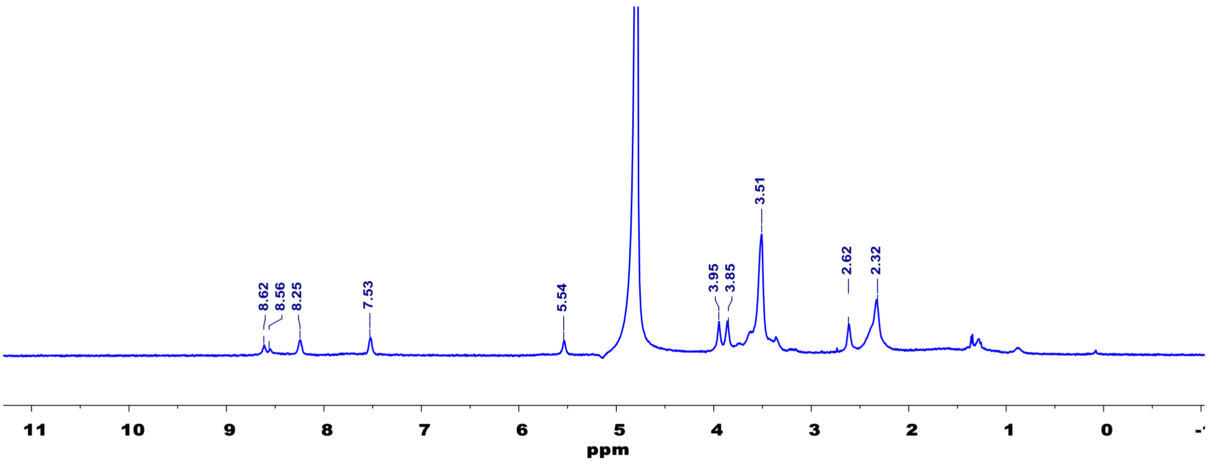

Figure S22. ^{1}H NMR (500 MHz, D_2O) spectrum of receptor **7**.

Synthesis of Receptor 8

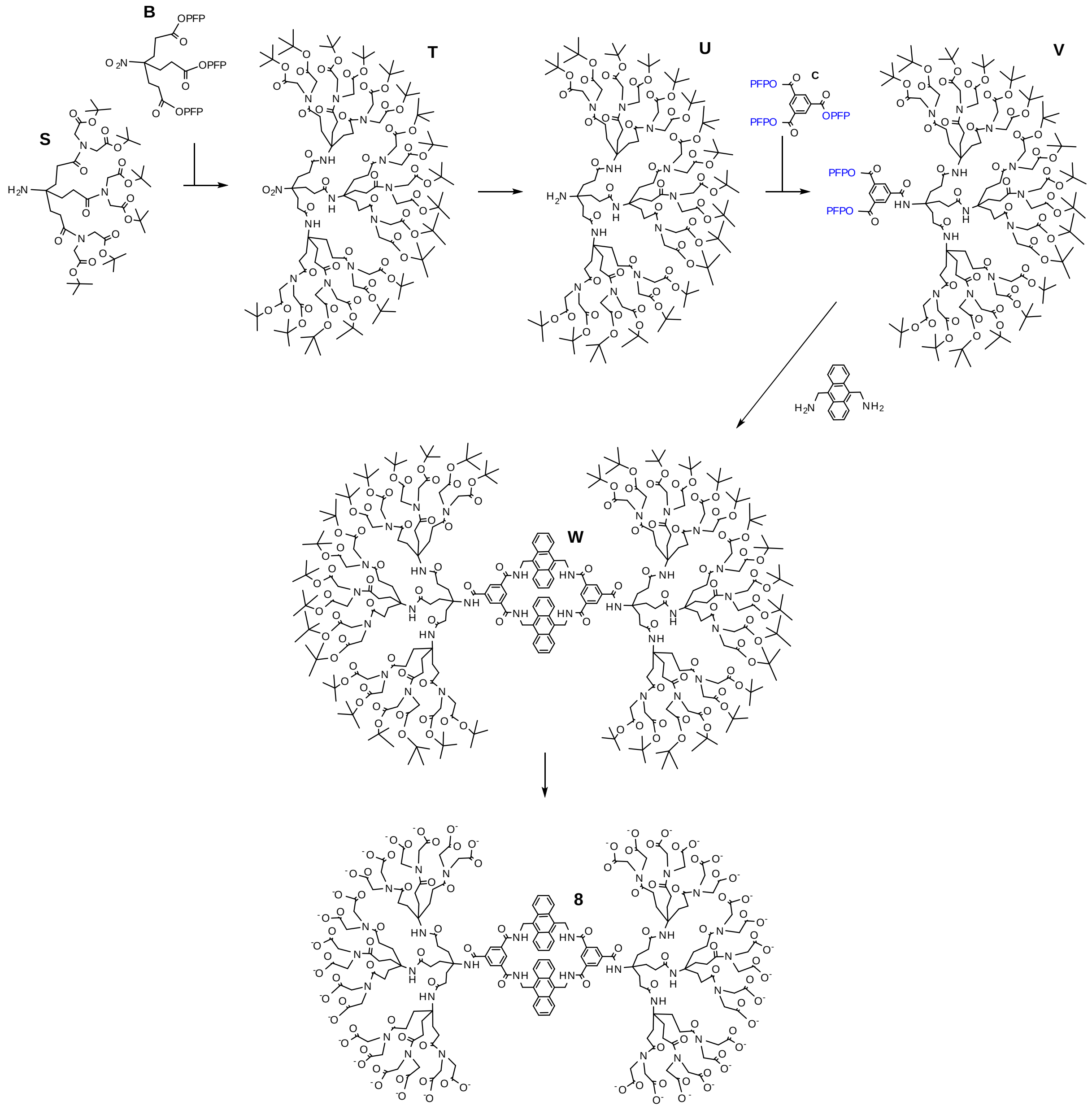

Figure S23. Synthesis of receptor **8**.

Experimental details

Nitromethanetris(pentafluorophenylpropionate) ester S

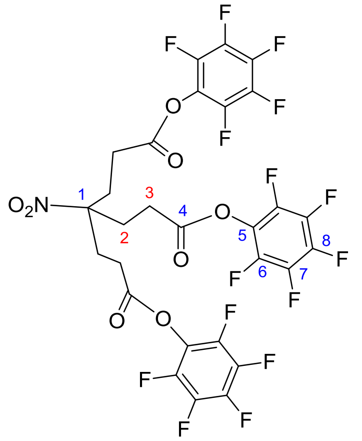

A solution of DCC (7.15 g, 34.6 mmol) in THF (10 mL, anhydrous) was added dropwise over 5 min to a solution of nitromethanetrispropionic acid[1] (3.00, 10.8 mmol) and pentafluorophenol (5.78 g, 31.4 mmol) in THF (30 mL, anhydrous). The reaction left stirring under N_2 for 16 hr at RT. The solvent was removed under reduced pressure and the residue purified via column chromatography (DCM/hexane, 7:3) yielding nitromethanetris(pentafluorophenylpropionate) **S** as a white solid (6.2 g, 76 %). R_f = 0.5 (DCM/hexane, 7:3). ^1H NMR (500 MHz, $CDCl_3$) δ = 2.49 (t, J_{HH} = 8.2 Hz, 6H, H2), 2.81 (t, J_{HH} = 8.2 Hz, 6H, H3). ^{13}F NMR (370 MHz, $CDCl_3$) δ = -152.7 (dd, J_{FF} = 22.6, 5.0 Hz, 2F, 6F), -157.1 (t, J_{FF} = 21.7 Hz, 1F, F8), -161.74-161.92 (m, 2F, F7). ^{13}C NMR (125 MHz. $CDCl_3$) δ = 28.03 (C3), 30.15 (C2), 91.17 (C1), 136.8 (C6), 138.7 (C8), 139.8 (C5), 142.5 (C7), 167.98 (C4). MS (ESI): m/z calculated for $C_{28}H_{12}NO_8F_{15}Na$ $[M + Na]^+$ = 798.02, found: 798.02.

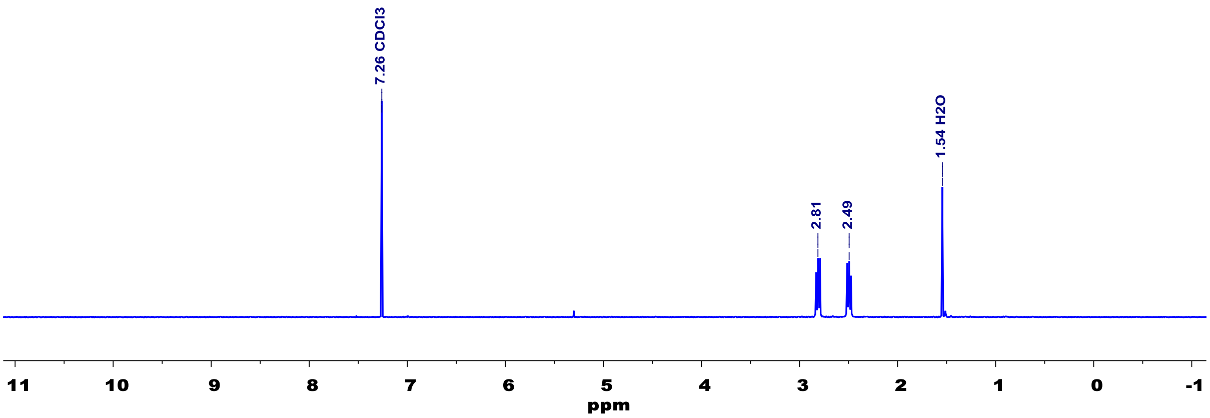

Figure S24. ^1H NMR (500 MHz, $CDCl_3$) spectrum of nitromethanetris(pentafluorophenylpropionate) **S**.

Nitro-octadeca-ester T

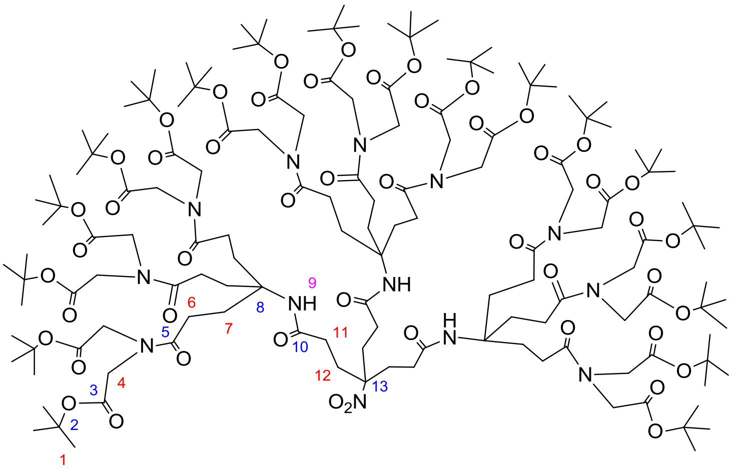

Amino-hexa-ester **B** (1.4 g, 1.50 mmol) and nitromethanetris(pentafluorophenylpropionate) **S** (0.29 g, 0.4 mmol) were dissolved in THF (5 mL, anhydrous) over molecular sieves (4Å). DIPEA (0.5 mL, 2.80 mmol) was added and the reaction heated (40 °C) under N_2 with stirring for 48 h. The solvent was removed under vacuum and toluene (3 x 10 mL) added and evaporated. The residue was purified via flash chromatography (hexane/EtOAc, 2:3 to 0:100) to yield nitro-octadeca-ester **T** as a white foam (0.5 g, 46 %). R_f = 0.30 (hexane/EtOAc, 2:3). ^{1}H NMR (500 MHz, $CDCl_3$/MeOD, 1:1) δ = 1.10, 1.12 (2s, 162H, H1), 1.65 (t, J_{HH} = 7.00 Hz, 18H, H7), 1.82 (m, 12H, H11/12), 1.95 (t, J_{HH} = 7.00 Hz, 18H, H6), 3.64, 3.74 (2s, 36H, H4), 6.97 (s, 3H, H9). ^{13}C NMR (125 MHz, $CDCl_3$/MeOD, 1:1) δ = 26.38 (C6), 27.21/27.24 (C1), 29.58 (C7), 30.34 (C12), 30.90 (C11), 48.71/50.74 (C4), 57.05 (C8), 81.40/82.30 (C2), 92.00 (C13), 167.95/168.02 (C3), 171.33 (C10), 173.60 (C5). MS (MALDI): *m/z* calculated for $C_{148}H_{249}N_{13}O_{20}Na$ [M + Na]$^+$ = 3031.72, found: 3031.979.

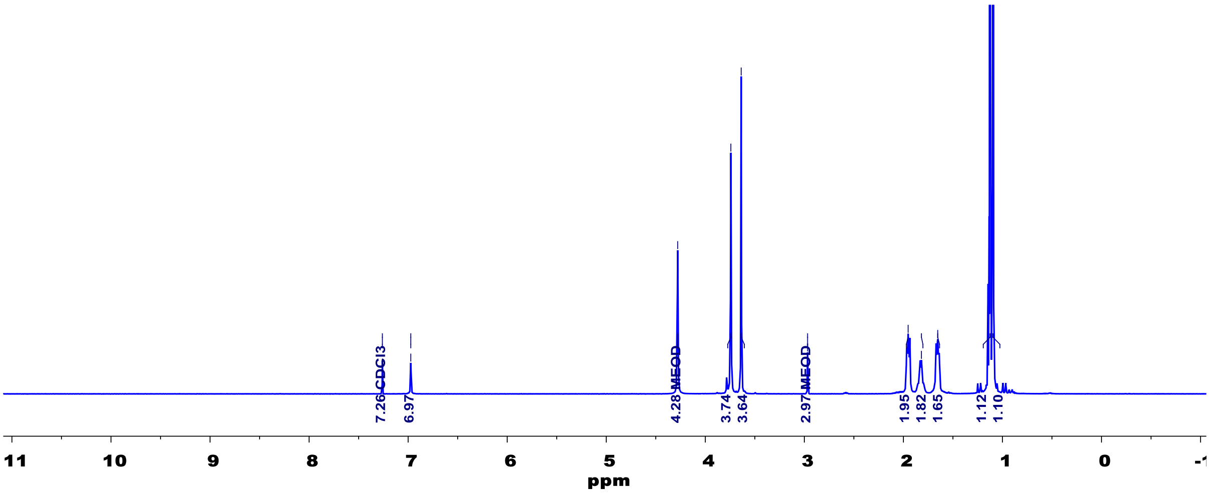

Figure S25. ^{1}H NMR (500 MHz, $CDCl_3$/MeOD, 1:1) spectrum of nitro-octadeca-ester **T**.

Amino-octadeca-ester U

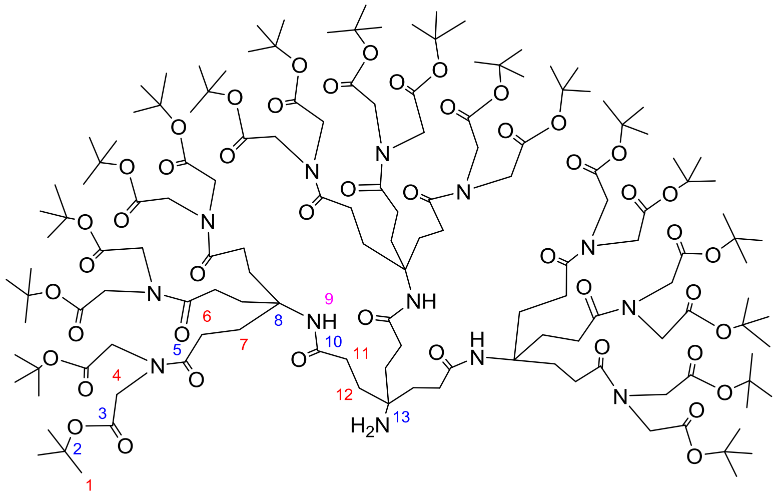

Nitro-octadeca-ester **T** (175 mg, 59.2 µmol), Raney Ni (2 mL, water slurry) and ethanol (30 mL) were added to an autoclave (250 mL). The autoclave was then sealed, pressurised with H_2 (50 bar) and left stirring for 24 h at 50 °C. The mixture was filtered through celite, rinsing with DCM (3 x 20 mL), and the solvent removed under reduced pressure to yield amino-octadeca-ester **U** (170 mg, 98%). R_f = 0.20 (EtOAc). ^{1}H NMR (500 MHz, $CDCl_3$/MeOD, 1:1) δ = 1.17, 1.19 (2s, 171H, H1), 1.62 (t, J_{HH} = 6.00 Hz, 6H, H11), 1.72 (t, J_{HH} = 7.00 Hz, 18H, H7), 2.02 (t, J_{HH} = 7.00 Hz, 18H, H6), 2.08 (t, J_{HH} = 6.00 Hz, 6H, H12), 3.72/3.80 (2s, 36H, H4). ^{13}C NMR (125 MHz, $CDCl_3$/MeOD, 1:1) δ = 26.50 (C6), 27.42 (C1), 29.41 (C7), 29.56 (C12), 30.96 (C11), 48.93/50.91 (C4), 56.18 (C13), 57.56 (C8), 81.72/82.55 (C2), 168.07/168.10 (C3), 172.55 (C10), 173.90 (C5). MS (MALDI): *m/z* calculated for $C_{148}H_{252}N_{13}O_{18}Na$ [M + H]$^+$ = 2979.76, found: 2980.21.

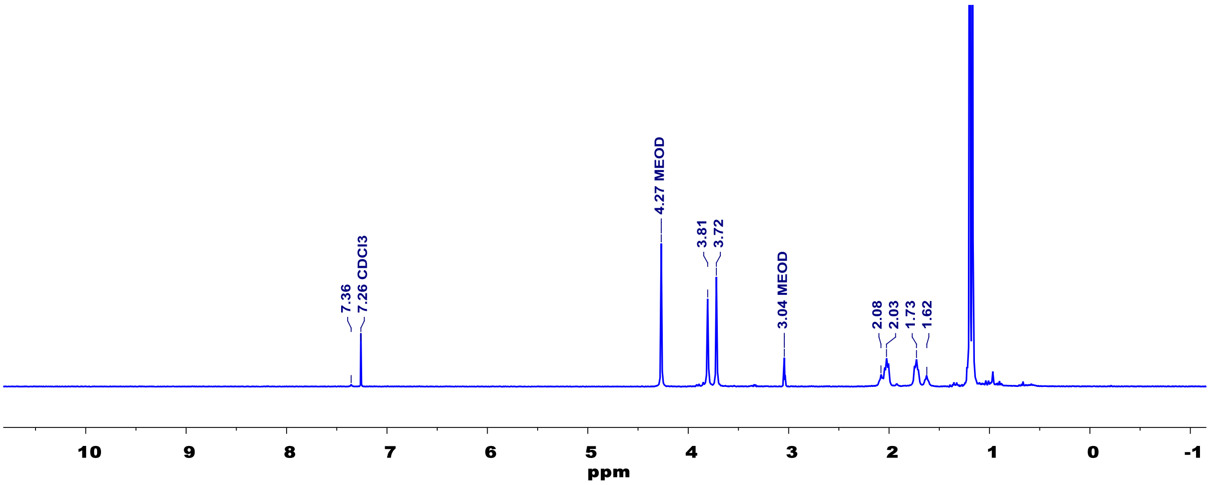

Figure 26. ^{1}H NMR (500 MHz, $CDCl_3$/MeOD, 1:1) spectrum of amino-octadeca-ester **U**.

Bis pentafluorophenyl ester V

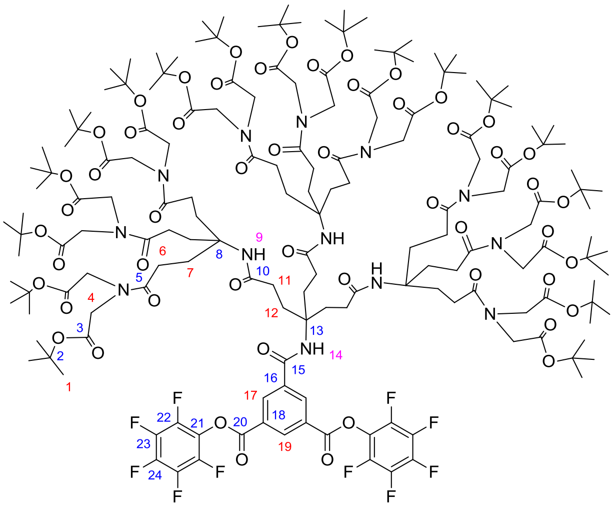

Tris-pentafluorophenyl ester **C** (150 mg, 211 µmol) and amino-octadeca-ester **U** (170 mg, 57.0 µmol) were loaded into a young's tube (4 mL) and dissolved in THF (1 mL, anhydrous) with DIPEA (200 µL). The tube was sealed under N_2, heated (45 °C) and left stirring for 4 h. The solvent was removed under vacuum and toluene (3 x 5 mL) added and removed on the rotary evaporator. The residue was dissolved in acetone/water (80:20, 8 mL), filtered (45 µm, syringe filter) and injected into a preparative reverse phase HPLC apparatus fitted with a reverse phase column (Waters – Xselect, 250 x 19 mm, 5µm) and eluted with acetone/water (80:20 to 100:0 over 20 min; flow rate 19 mL/min). The component eluting at 12 min was collected, concentrated under vacuum and freeze-dried to yield bis-pentafluorophenyl ester **V** (128 mg, 48 %) as a white solid. R_f = 0.65 (hexane/EtOAc, 3:2). ^{1}H NMR (500 MHz, $CDCl_3$) δ = 1.42, 1.43 (2s, 171H, H1), 2.01 (t, J_{HH} = 7.20 Hz, 18H, H7), 2.14 (t, J_{HH} = 7.80 Hz, 6H, H11), 2.24 (t, J_{HH} = 7.80 Hz, 6H, H12), 2.27 (t, J_{HH} = 7.20 Hz, 18H, H6), 3.97/4.00 (2s, 36H, H4), 6.91 (s, 3H, H9), 9.02 (t, J_{HH} = 1.80 Hz, 1H, H19), 9.15 (d, J_{HH} = 1.80 Hz, 2H, H17), 9.40 (s, 1H, H14). ^{13}C NMR (125 MHz, $CDCl_3$) δ = 27.06 (C6), 28.14/28.19 (C1), 30.45 (C7), 31.67/31.70 (C11/12), 48.94/51.21 (C4), 57.70 (C8), 58.35 (C13), 81.66/82.71 (C2), 128.29 (C17), 134.69 (C19), 135.57 (C17), 137.72 (C18), 161.38 (C20), 163.37 (C15), 168.33/168.48 (C3), 173.14 (C10), 173.41 (C5). MS (MALDI): *m/z* calculated for $C_{169}H_{253}F_{10}N_{13}O_{53}Na$ $[M + Na]^+$ = 3525.7, found: 3526.2.

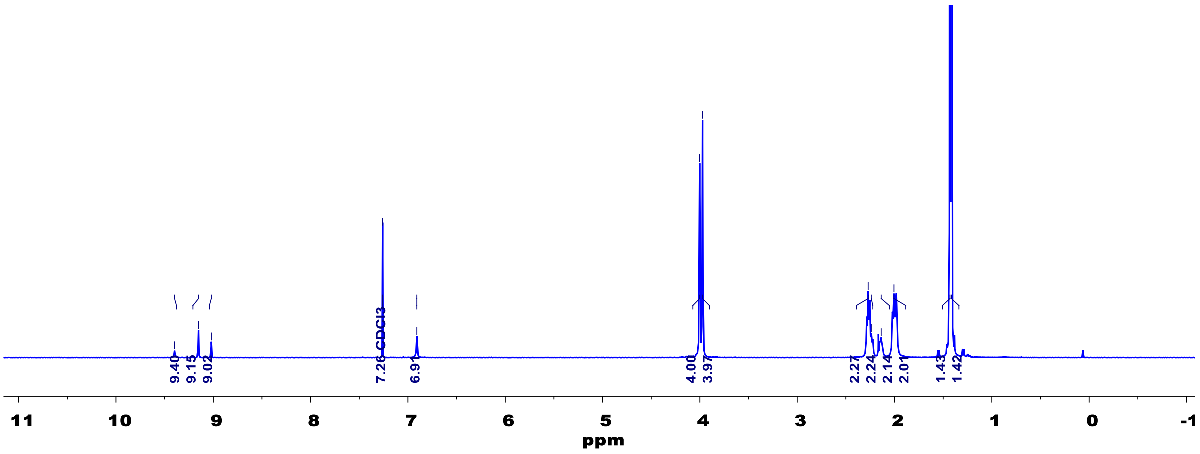

Figure S27. ^{1}H NMR (500 MHz, $CDCl_3$) spectrum of bispentafluorophenyl ester **V**.

Protected Receptor W

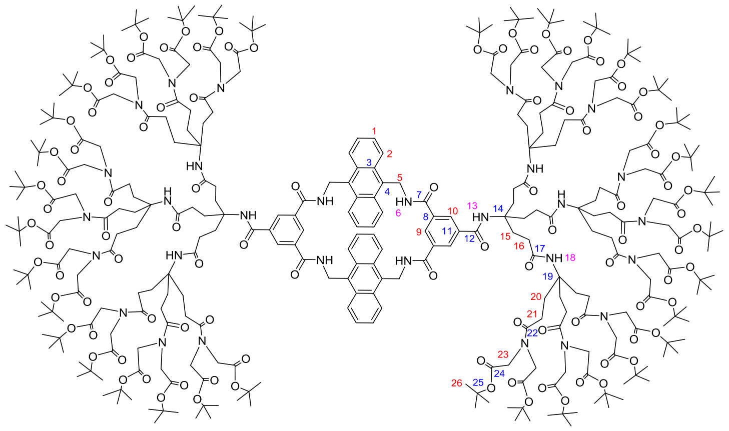

9,10-Bis-(aminomethyl)anthracene[3] (7.53 mg, 31.4 µmol) was dissolved in THF (250 mL, anhydrous) and DIPEA (2 mL, 21.9 mmol). A solution of bis-pentafluorophenyl ester **V** (110 mg, 31.4 µmol) in THF (50 mL, anhydrous) was injected into the solution of amine over 36 h with an automated syringe pump under N_2 with stirring. After the addition the reaction was left for a further 36 h. The solvent was removed under vacuum and toluene (3 x 50 mL) was added and removed on the rotary evaporator. The residue was dissolved in acetone/water (9:1, 8 mL), filtered (45 µm, syringe filter) and injected into a preparative reverse phase HPLC apparatus fitted with a reverse phase column (Waters – Xselect, 250 x 19 mm, 5µm) and eluted with acetone/water (85:15 to 95:5 over 30 min; flow rate 19 mL/min). The component eluting at 13 min was collected, concentrated under vacuum and freeze-dried to yield protected receptor **W** (37.0 mg, 35.0 %) as a white powder. R_f = 0.5 (hexane/EtOAc, 3:7). ^{1}H NMR (500 MHz, $CDCl_3$/MeOD, 1:1) δ = 1.41/1.45 (2s, H324, H26), 2.05 (t, J_{HH} = 7.10 Hz, 36H, H20), 2.17 (m, 12H, H16), 2.26 (m, 12H, H15), 2.32 (t, J_{HH} = 7.10 Hz, 36H, H21), 4.00/4.12 (s, 72H, H23), 5.44 (s, 8H, H5), 7.35 (s, 6H, H18), 7.42 (dd, J_{HH} = 2.60, J_{HH} = 6.70, 8H, H2), 7.54 (s, 4H, H6), 7.64 (s, 2H, H9), 8.26 (s, H2, H13), 8.27 (dd, J_{HH} = 2.60, J_{HH} = 6.70, 8H, H1), 8.65 (s, 4H, H10). ^{13}C NMR (125 MHz, $CDCl_3$/MeOD, 1:1) δ = 27.21 (C21), 28.25 (C26), 30.40 (C20), 31.84 (C15), 32.05 (C16), 37.50 (C5), 49.51/51.65 (C23), 58.14/58.06 (C14/19), 82.31/83.23 (C25), 125.07 (C1), 126.48 (C2), 126.51 (C3), 126.53 (C11), 126.56 (C9), 130.11 (C4), 130.85 (C10), 130.96 (C8), 166.79 (C12), 166.95 (C7), 168.92 (C22), 174.45 (C17), 174.41 (C24). MS (MALDI): *m/z* calculated for $C_{346}H_{534}N_{30}O_{102}Na$ $[M + Na]^+$ = 6764.74, found: 6768.0.

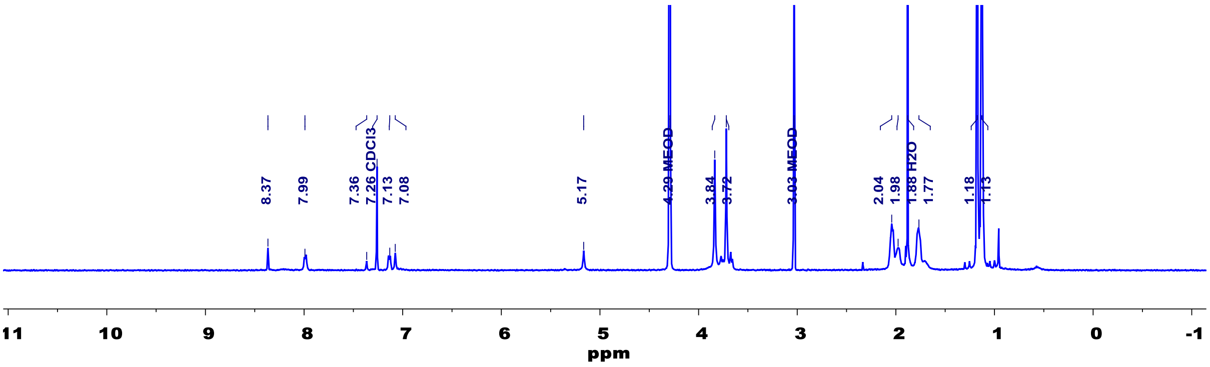

Figure S28. ^{1}H NMR (500 MHz, $CDCl_3$MeOD, 1:1) spectrum of protected receptor **W**.

Receptor 8

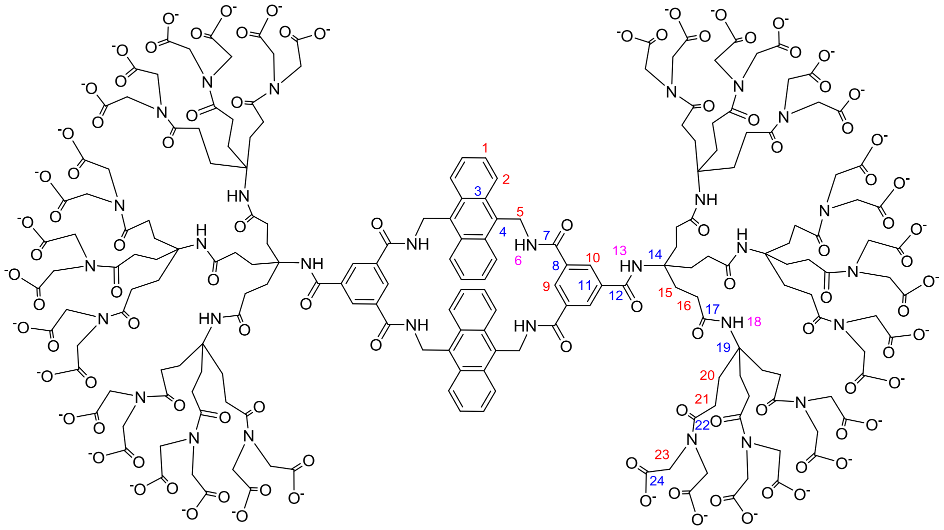

Protected receptor **W** (25 mg, 3.00 µmol) and triethylsilane (36.4 mg, 0.31 mmol) were dissolved in DCM (2 mL) and cooled to 0 °C over ice. TFA (2 mL) was added drop wise over 5 minutes and the reaction left for 6 h at RT under nitrogen. The solvent was removed under vacuum and then dissolved in water/MeOH (6:4, 10 mL) and NaOH (0.1 M) added dropwise until pH = 7. The solution was passed through a 45 µm syringe filter and the water removed on the freeze drier to yield receptor **8** (16.5mg, 80%). ^{1}H NMR (500 MHz, D_2O) δ = 2.03-2.35 (m, 144H, H15/16/20/21), 3.93/4.01 (2s, 144H, H23), 5.44 (s, 8H, H5), 7.53 (s, 8H, H2), 8.05 (s, 2H, H9), 8.25 (s, 8H, H1), 8.68 (s, 4H, H10) ^{13}C NMR (125 MHz, D_2O) δ = 26.90 (C21), 29.27 (C20), 30.59 (C15), 31.11 (C16), 37.79 (C5), 51.27/53.01 (C23), 58.10 (C19), 58.34 (C14), 124.48 (C1), 126.60 (C2), 126.51 (C3), 127.39 (C11), 127.62 (C9), 130.02 (C4), 130.99 (C10), 130.82 (C8), 167.94 (C12), 168.14 (C7), 172.91 (C22), 175.52 (C24), 175.56 (C17).

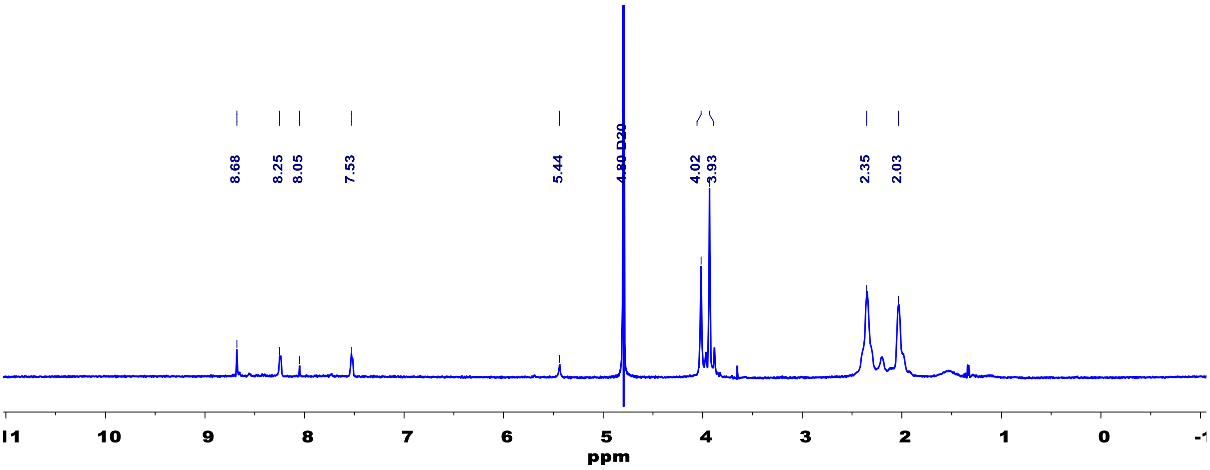

Figure S29. ^{1}H NMR (500 MHz, D_2O) spectrum of receptor **8**.

Synthesis of Receptor 9

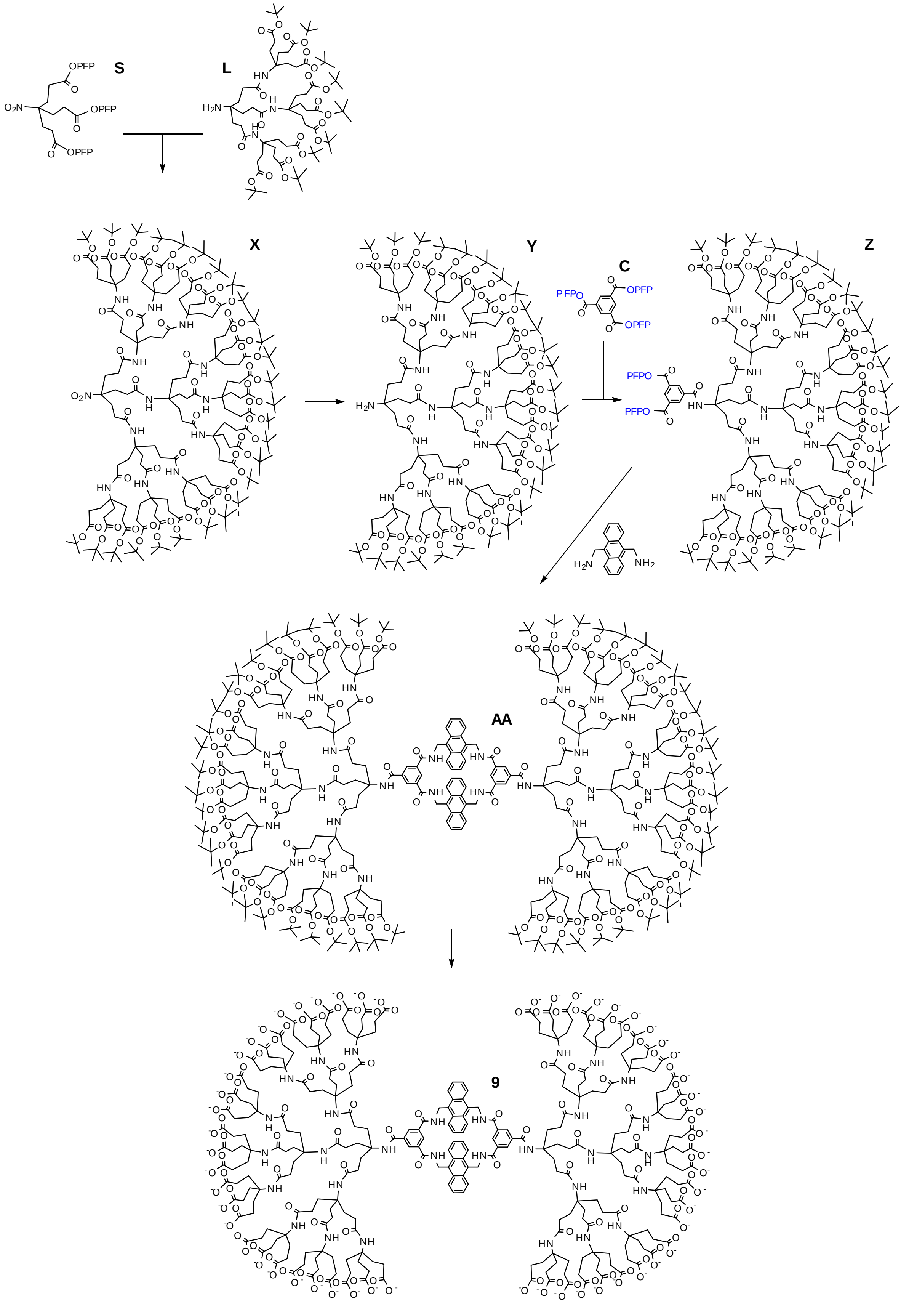

Figure S30. Synthesis of Receptor **9**.

Experimental

Nitro-heptacosa-ester X

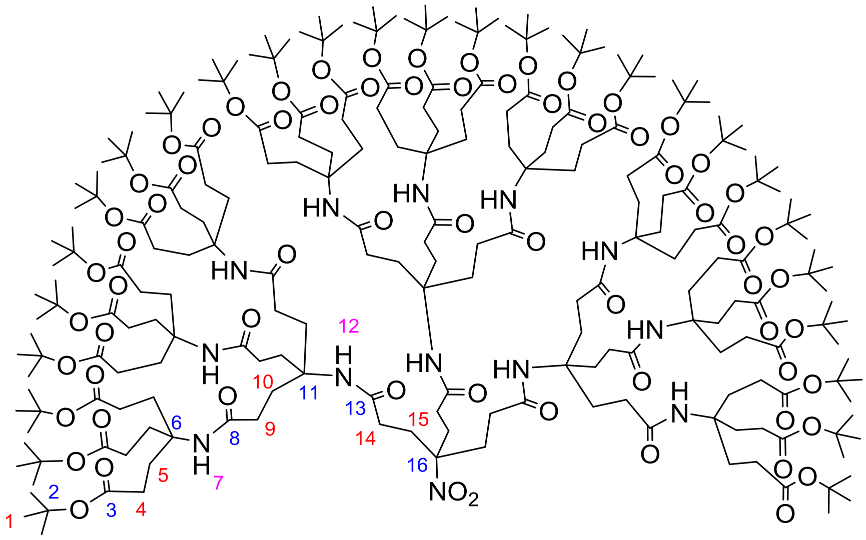

Amino-nona-ester **L** (6.31 g, 4.4 mmol) and nitromethanetris(pentafluorophenylpropionate) **S** (1.00 g, 1.30 mmol) was dissolved in THF (20 mL, anhydrous) under N_2. The reaction was heated to 50 °C and left stirring over molecular sieves (4 Å) for 48 h. The solvent was removed and toluene was added and evaporated three times to remove the DIPEA. The residue was purified via column chromatography (hexane/EtOAc, 7:3 to 1:1 then EtOAc/MeOH, 95:5) to yield nitro-heptacosa-ester **X** as a white solid (3.04 g, 52%). R_f = 0.65 (hexane/EtOAc, 1:1). ^{1}H NMR (500 MHz, $CDCl_3$) δ = 1.42 (s, 243H, H1), 1.94 (m, 78H, H5/10/15), 2.16 (m, 78H, H4/9/14), 6.28 (s, 9H, H7), 7.00 (s, 3H, H12) [lit.[7] (400 MHz, $CDCl_3$) δ = 1.42 (s, 243 H), 1.93 (m, 78H), 2.12 (m, 78H), 6.19 (s, 12H)]. ^{13}C NMR (125 MHz, $CDCl_3$) δ = 28.26 (C1), 29.93 (C4/5/14/15), 31.33 (C10), 31.63 (C9), 57.52 (C6), 58.11 (C11), 80.55 (C2), 93.07 (C16), 172.81 (C13), 172.86 (C3), 172.90 (C8) HRMS (ESI): m/z calculated for $C_{238}H_{411}O_{68}N_{13}Na_3$ [M + 3Na]$^{3+}$ = 1536.2938, found: 1536.2926.

Amino-heptacosa-ester Y

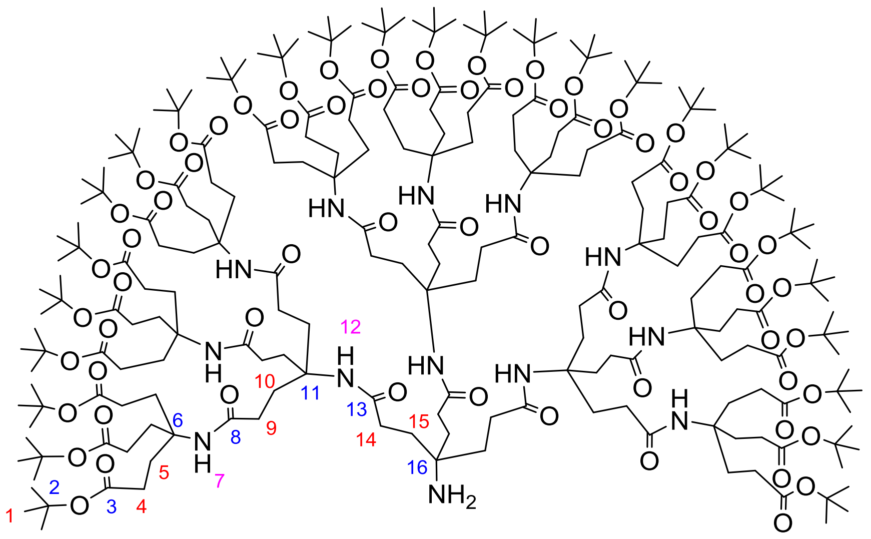

Nitro-heptacosa-ester **X** (2.93 g, 0.64 mmol), Raney Ni (10 mL, water slurry) and ethanol (40 mL) were added to an autoclave (250 mL). The autoclave was sealed, pressurised with H_2 (50 bar) and left stirring for 24 h at 60 °C. The mixture was filtered through celite, washing with DCM (50 mL) and the solvent removed under reduced pressure to yield amino-heptacosa-ester **Y** (2.90 g, 99%). R_f = 0.50 (EtOAc). ^{1}H NMR (500 MHz, $CDCl_3$) δ = 1.40 (s, 243H, H1), 1.92 (m, 78H, H5/10/15), 2.17 (m, 78H, H4/9/14), 6.41 (s, 9H, H7), 7.66 (s, 3H, H12) [lit.[7] (400 MHz, $CDCl_3$) δ = 1.22 (s, 243H), 1.77 (m, 78H), 2.00 (m, 78H), 6.14 (s, 12H)]. HRMS (ESI): *m/z* calculated for $C_{238}H_{414}O_{66}N_{13}Na_3$ $[M + 3Na]^{3+}$ = 1518.9739, found: 1518.9682.

Bis-pentafluorophenyl ester Z

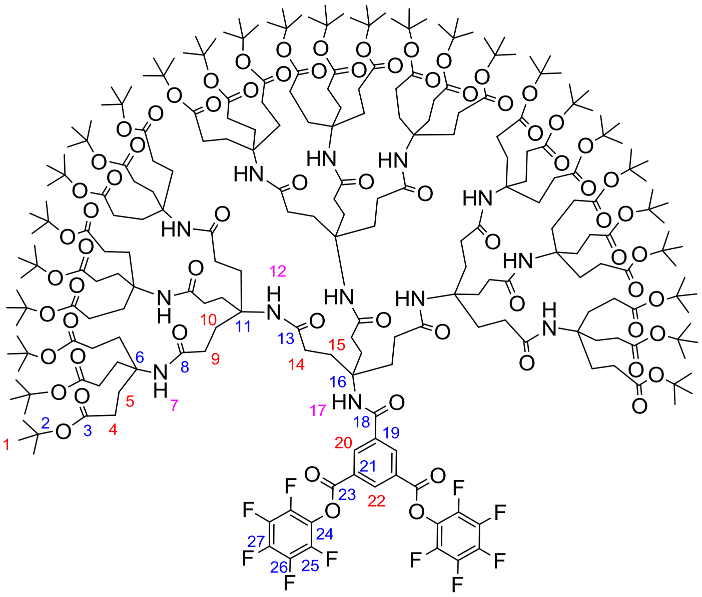

Amino-heptacosa-ester **Y** (0.50 g, 111 µmol) and tris-pentafluorophenyl ester **C** (0.54 g, 333 µmol) were dissolved in THF (1 mL, anhydrous) under N_2 over molecular sieves (4Å). DIPEA (1 mL, 10.4 mmol) was injected and the reaction heated to 40 °C and left stirring for 4 h at RT under N_2. The solvent was removed under vacuum and toluene (60 mL) was added and removed three times on the rotary evaporator to remove the DIPEA. The residue was purified via column chromatography (hexane/EtOAc, 6:4 to 4:6 to 0:1) to yield bis-pentafluorophenyl ester **Z** as a white foam (280 mg, 50 %). R_f = 0.5 (hexane/EtOAc, 1:1). ^{1}H NMR (500 MHz, $CDCl_3$) δ = 1.41 (, 243H, H1), 1.92 (m, 78H, H5/10/15), 2.16 (m, 78H, H4/9/14), 6.27 (s, 9H, H7), 6.88 (s, 3H, H12), 9.06 (s, 1H, H22), 9.14 (s, 2H, H20), 9.49 (s, 1H, H17). ^{13}C NMR (125 MHz, $CDCl_3$) δ = 28.24 (C1), 29.82-29.89 (C4/5/9/10/14/15), 57.99/58.61 (C11/16), 80.54 (C2), 135.41 (C21), 134.82 (C22), 135.01 (C19), 135.61 (C20), 161.24 (C23), 164.78 (C18), 172.75 (C3), 173.52 (C8), 174.06 (C13). ^{19}F NMR (470 MHz, $CDCl_3$) δ = -151.92 (d, J_{FF} = 19.1 Hz, 4F, F25), -157.32 (t, J_{FF} = 22.0 Hz, 2F, F27), -161.89 (t, J_{FF} = 20.2 Hz, 4F, F26). HRMS (ESI): m/z calculated for $C_{259}H_{415}O_{71}N_{13}F_{10}Na_3$ [M + 3Na]$^{3+}$ = 1700.9593, found: 1700.9530.

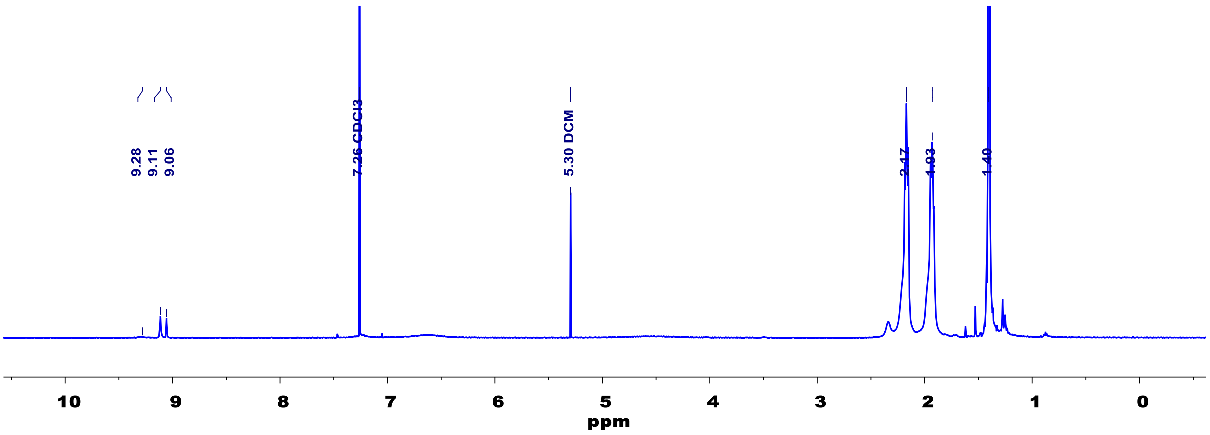

Figure S31. ^{1}H NMR (500 MHz, $CDCl_3$) spectrum of bis-pentafluorophenyl ester **Z**.

Protected receptor AA

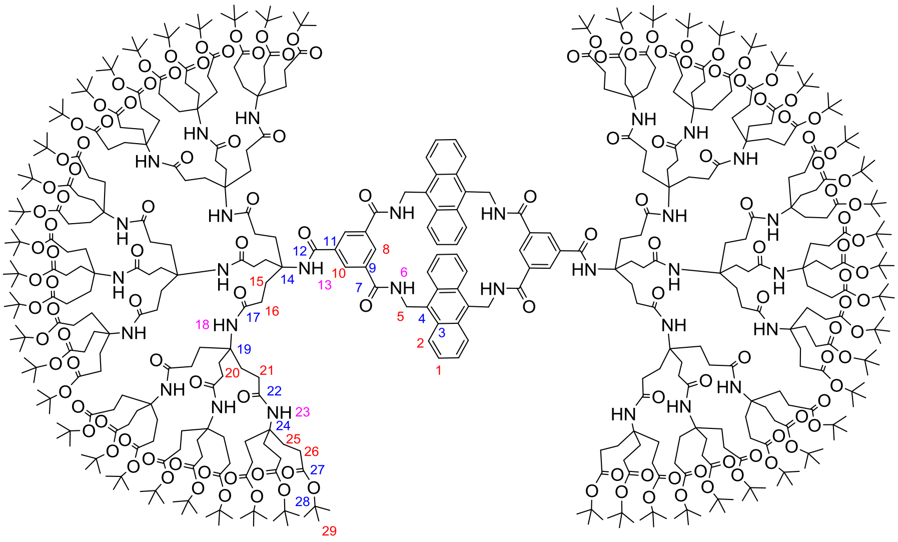

9,10 Bis-(aminomethyl)anthracene (13.1 mg, 55.6 µmol) was dissolved in THF (250 mL, anhydrous) and DIPEA (2 mL, 21.9 mmol). A solution of linker **Z** (280 mg, 55.6 µmol) in THF (50 mL, anhydrous) was injected into the solution of amine over 36 h with an automated syringe pump under N_2 with stirring. After the addition the reaction was left for a further 36 h. The solvent was removed under vacuum and the residue dissolved in DCM (50 mL) and washed with NH_4Cl (sat. aq., 50 mL), water (50 mL), brine (50 mL), then dried over $MgSO_4$, filtered and evaporated in vacuo. The residue was dissolved in acetone/water (85:15, 4 mL) and passed through a syringe filter (0.45 µm). The solution was injected into a preparative reverse phase HPLC apparatus fitted with a reverse phase column (Waters – Xselect, 250 x 19 mm, 5µm) and eluted with acetone/water (85:15 to 100:0 over 30 min; flow rate 19 mL/min). The component eluting at 22 min was collected, concentrated under vacuum and freeze-dried to yield **AA** (130 mg, 48 %) as a white powder. R_f = 0.5 (hexane/EtOAc, 2:3). ^{1}H NMR (500 MHz, $CDCl_3$) δ = 1.38 (s, 243H, H30), 1.96 (s, 108H, H25), 2.05 (m, 54H, H20/15), 2.19 (m, H26/21/16), 5.55 (s, 8H, H5), 6.58 (s, 18H, H23), 7.05 (s, 6H, H18), 7.34 (s, 2H, H13), 7.42 (m, 8H, H2), 7.48 (s, 2H, H8), 8.39 (m, 8H, H1), 8.67 (s, 4H, H10). ^{13}C NMR (125 MHz, $CDCl_3$): δ = 28.25 (C29), 29.84-29.91 (C25/26), 31.51 (C15/16/20/21), 37.51 (C5), 57.91/58.66 (C14/19/24), 80.54 (C28), 125.16 (C1), 126.24 (C2), 130.02 (C10), 172.81 (C27). MS (ESI): *m/z* calculated for $C_{526}H_{858}N_{30}O_{138}Na_3$ $[M + 3Na]^{3+}$ = 3291.02, found: 3292.90.

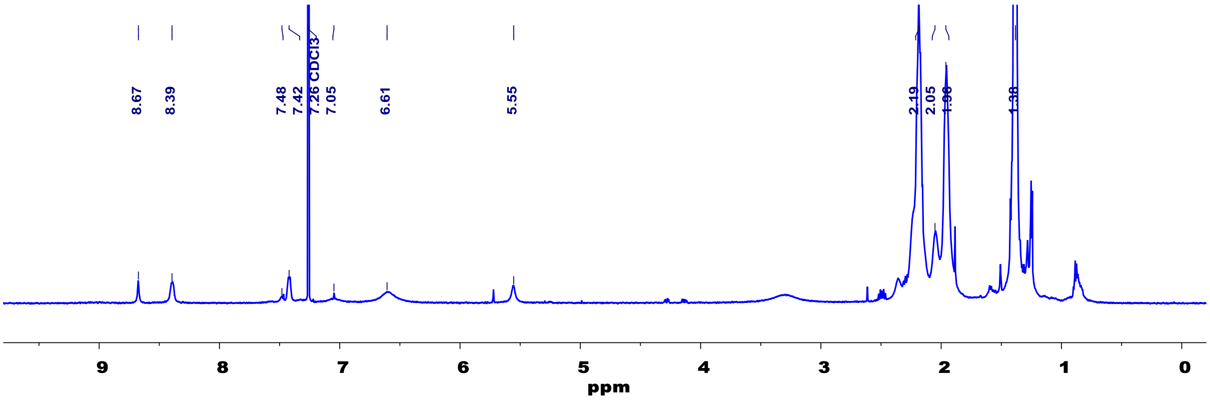

Figure S32. ^{1}H NMR (500 MHz, $CDCl_3$) spectrum of protected receptor **AA**.

Receptor 9

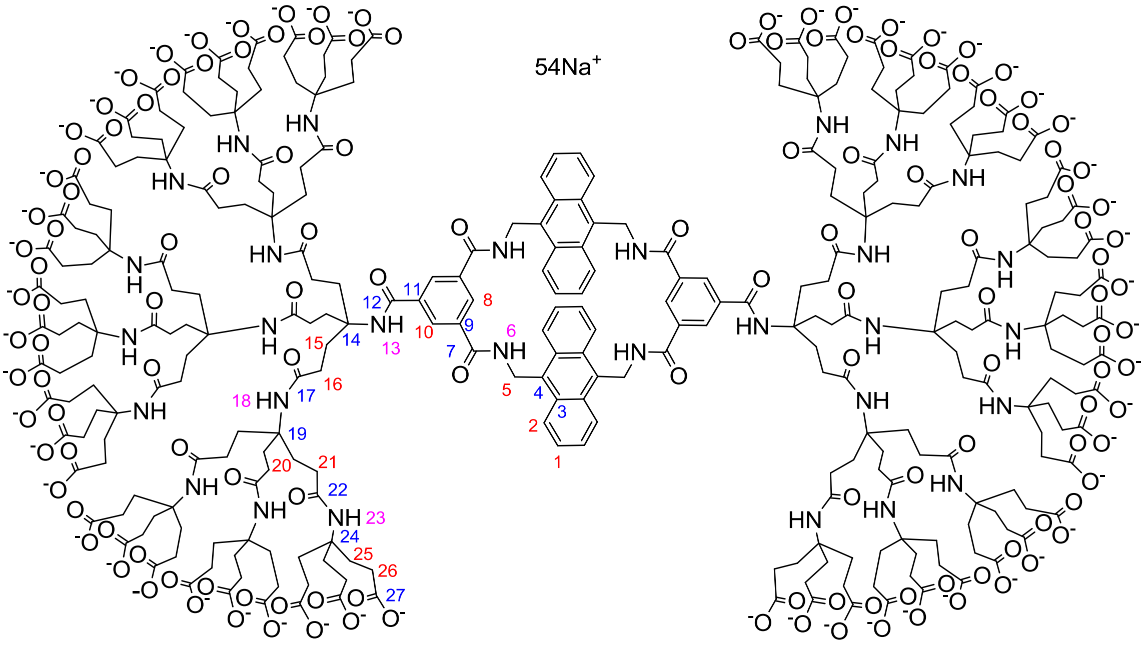

Protected receptor **AA** (68 mg, 18.6 µmol) was dissolved in DCM (6 mL) and cooled to 0 °C over ice. TFA (2 mL) was added drop wise over 5 minutes and the reaction left for 16 h at RT under N_2. The TFA was then removed under vacuum and the residue was dissolved in H_2O:MeOH (6:4, 10 mL). NaOH (0.1 M) was added until pH = 7 and the solution freeze dried to yield receptor **9** (56 mg, 99%). ^{1}H NMR (500 MHz, D_2O) δ = 1.93 (s, 108H, H25), 2.01 (s, 54H, H20/15), 2.20-2.23 (m, 162H, H26/21/16), 5.42 (s, 8H, H5), 7.52 (s, 8H, H2), 8.20 (m, 8H, H1), 8.39 (s, 2H, H8), 8.69 (s, 4H, H10). ^{13}C NMR (125 MHz, D_2O) δ = 30.28-30.85 C15/16/20/21/25/26, 37.64 (C5), 57.91/58.66 C14/19/24, 124.34 (C1), 130.65 (C10).

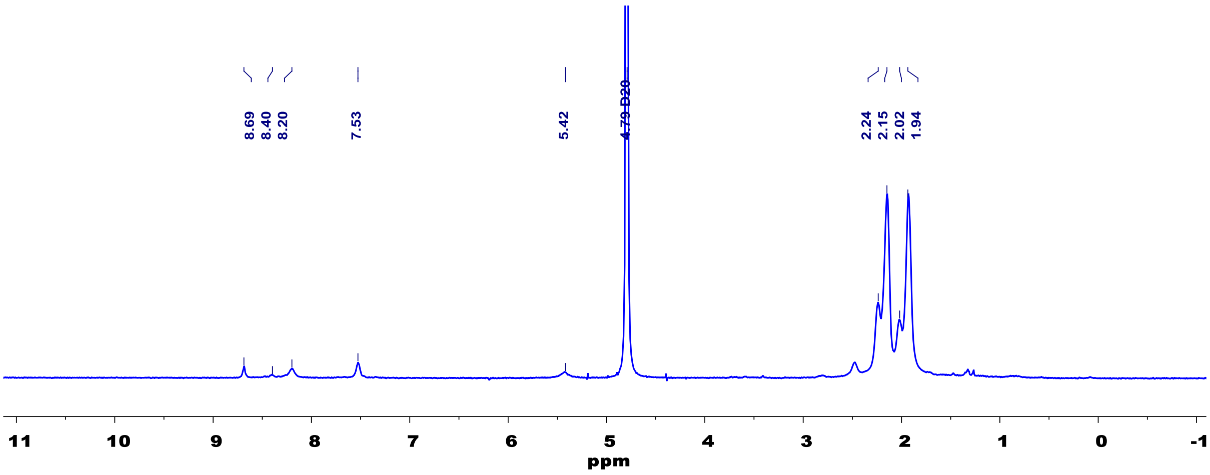

Figure S33. ^{1}H NMR (500 MHz, D_2O) spectrum of receptor **9**.

3. 2D NOESY Spectra

Receptor 4

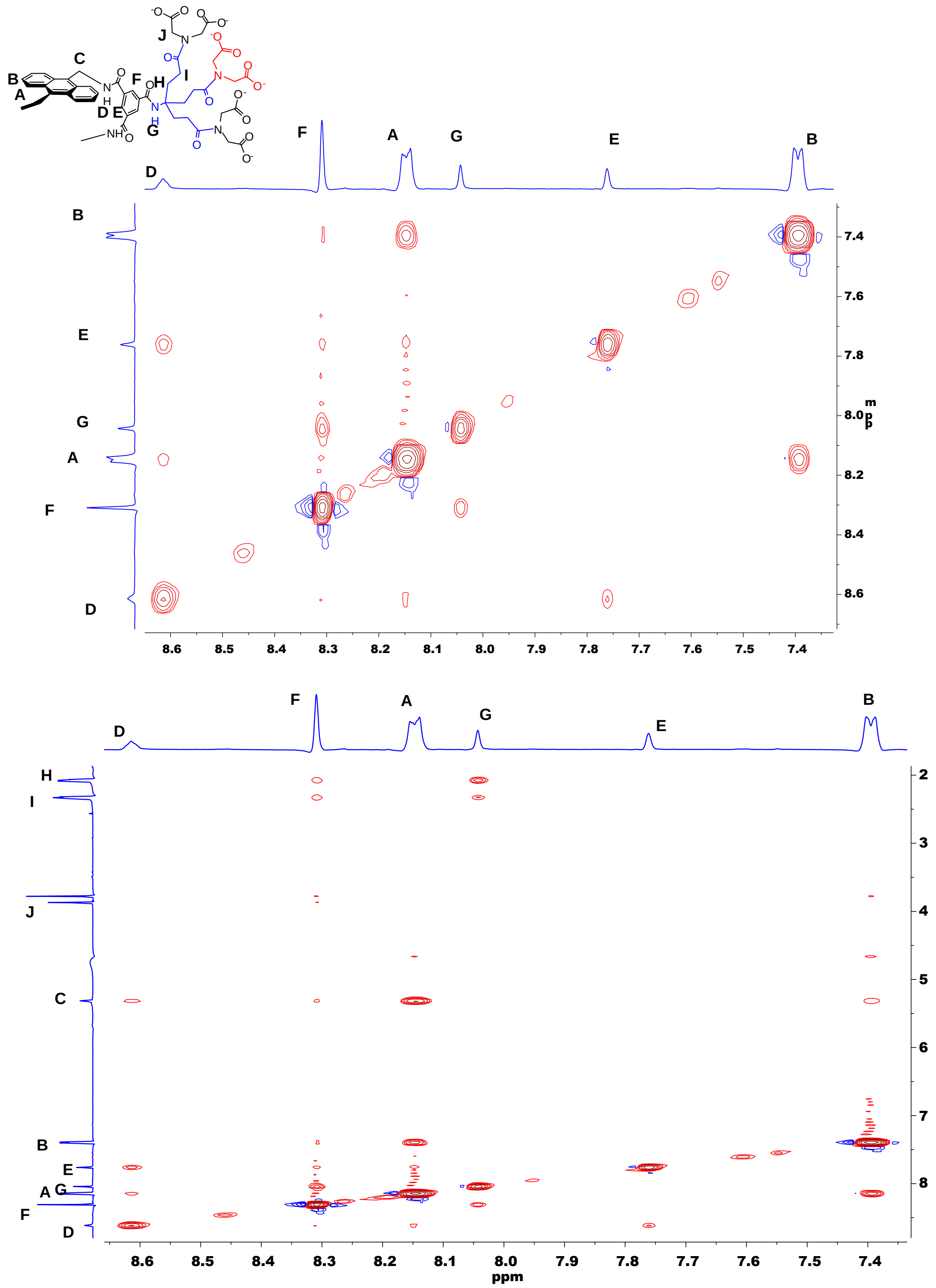

Figure S34. NOESY spectrum (600 MHz, mixing time: 250 ms) of receptor **4** (1.01 mM) in D_2O/H_2O (v/v = 1/9) at 298 K.

Receptor 5

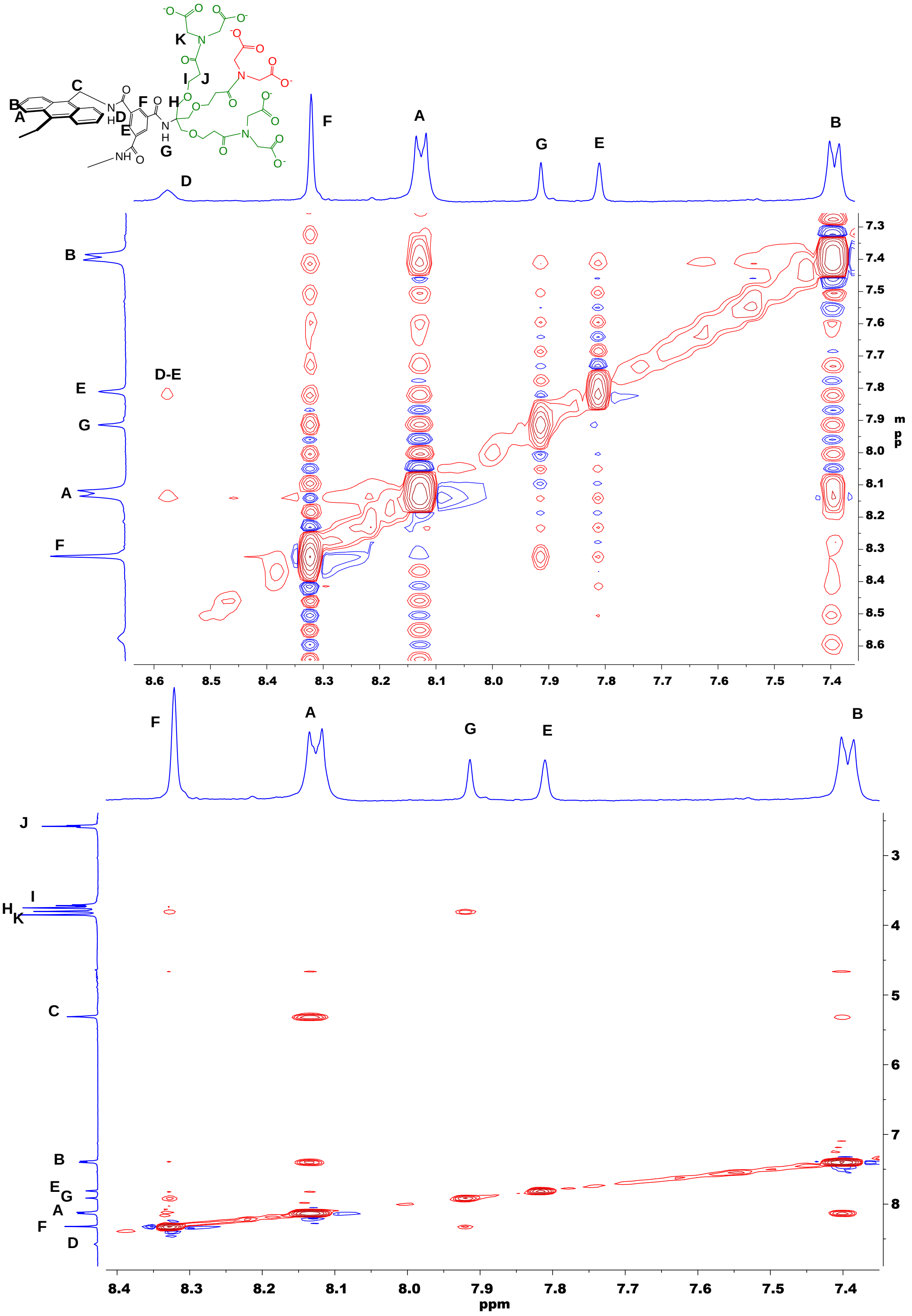

Figure S35. NOESY spectrum (600 MHz, mixing time: 250 ms) of receptor **5** (0.82 mM) in D_2O/H_2O (v/v = 1/9) at 298 K.

Receptor 6

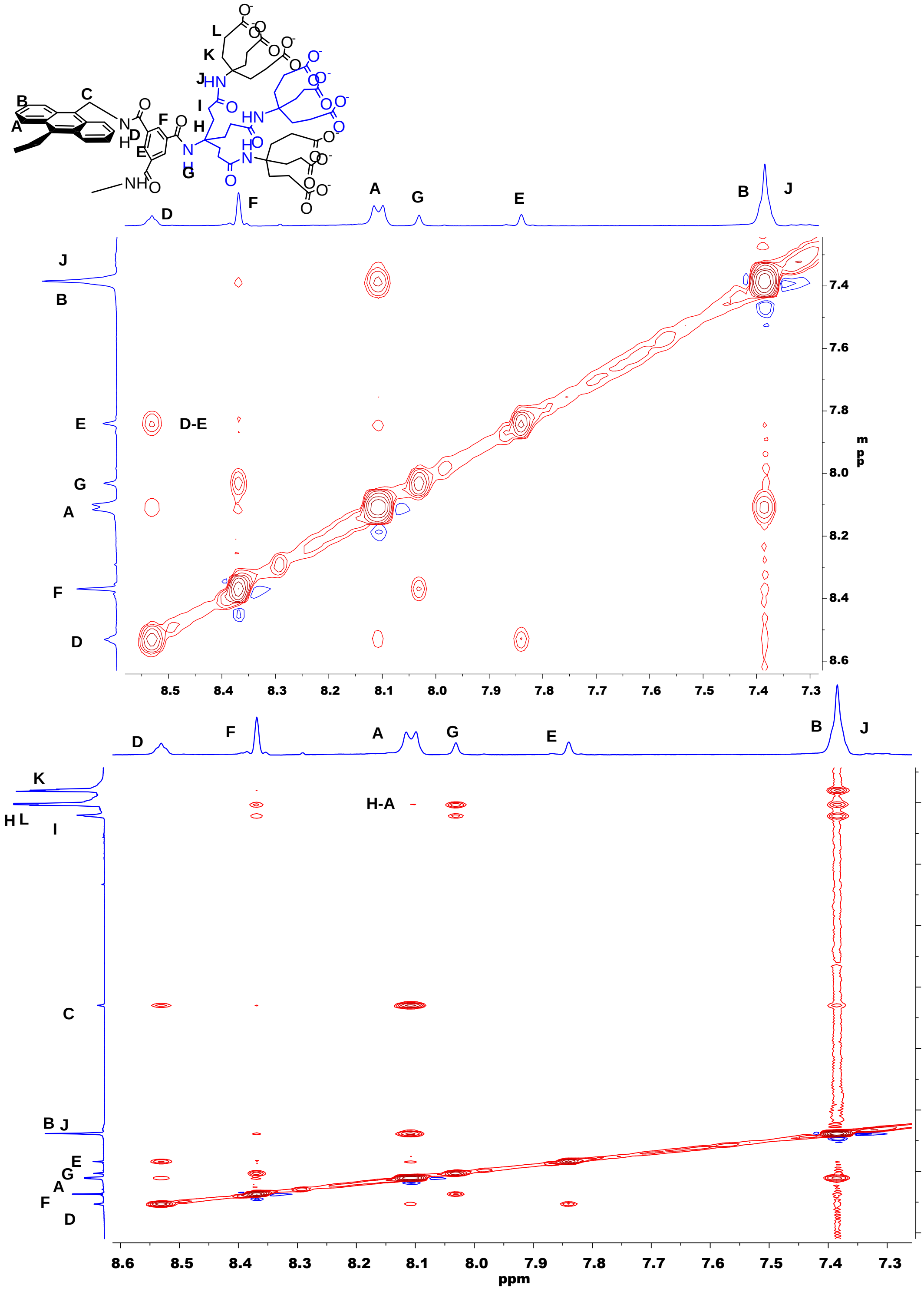

Figure S36. NOESY spectrum (600 MHz, mixing time: 250 ms) of receptor **6** (1.11 mM) in D_2O/H_2O (v/v = 1/9) at 298 K.

Receptor 7

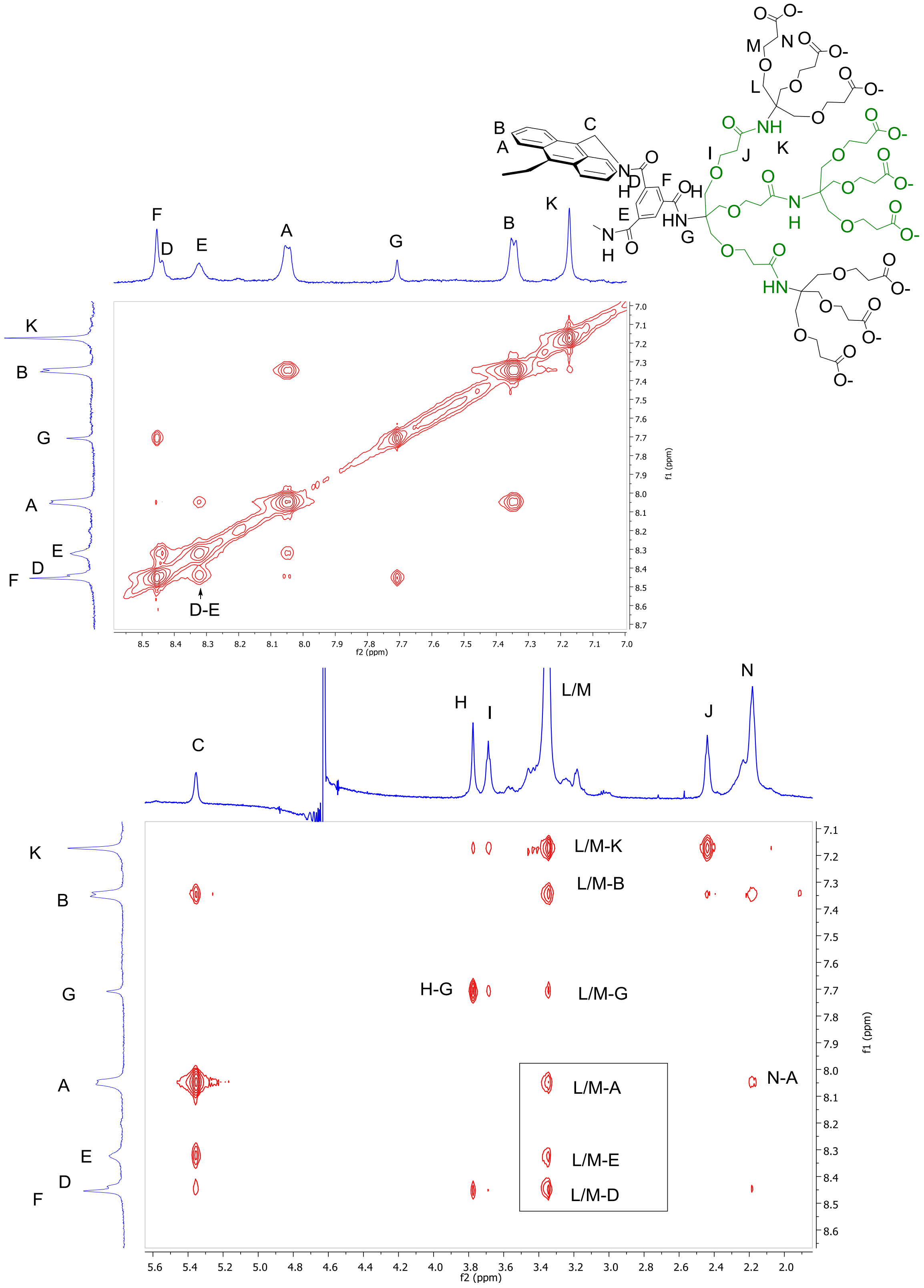

Figure S37. Partial NOESY spectrum (600 MHz, mixing time: 250 ms) of receptor **7** (1.0 mM) in D_2O/H_2O (v/v= 7/93) at 298 K. The spectrum shows a strong NOE connection between protons D and E which suggests that the core macrocycle is in expected “NH-in” conformation. Connections are observed between proton environments inside the cavity (E/D/A and B) and protons L and M on the solubilizing group arm, providing evidence that the solubilizing arm is threading through the cavity.

Receptor 8

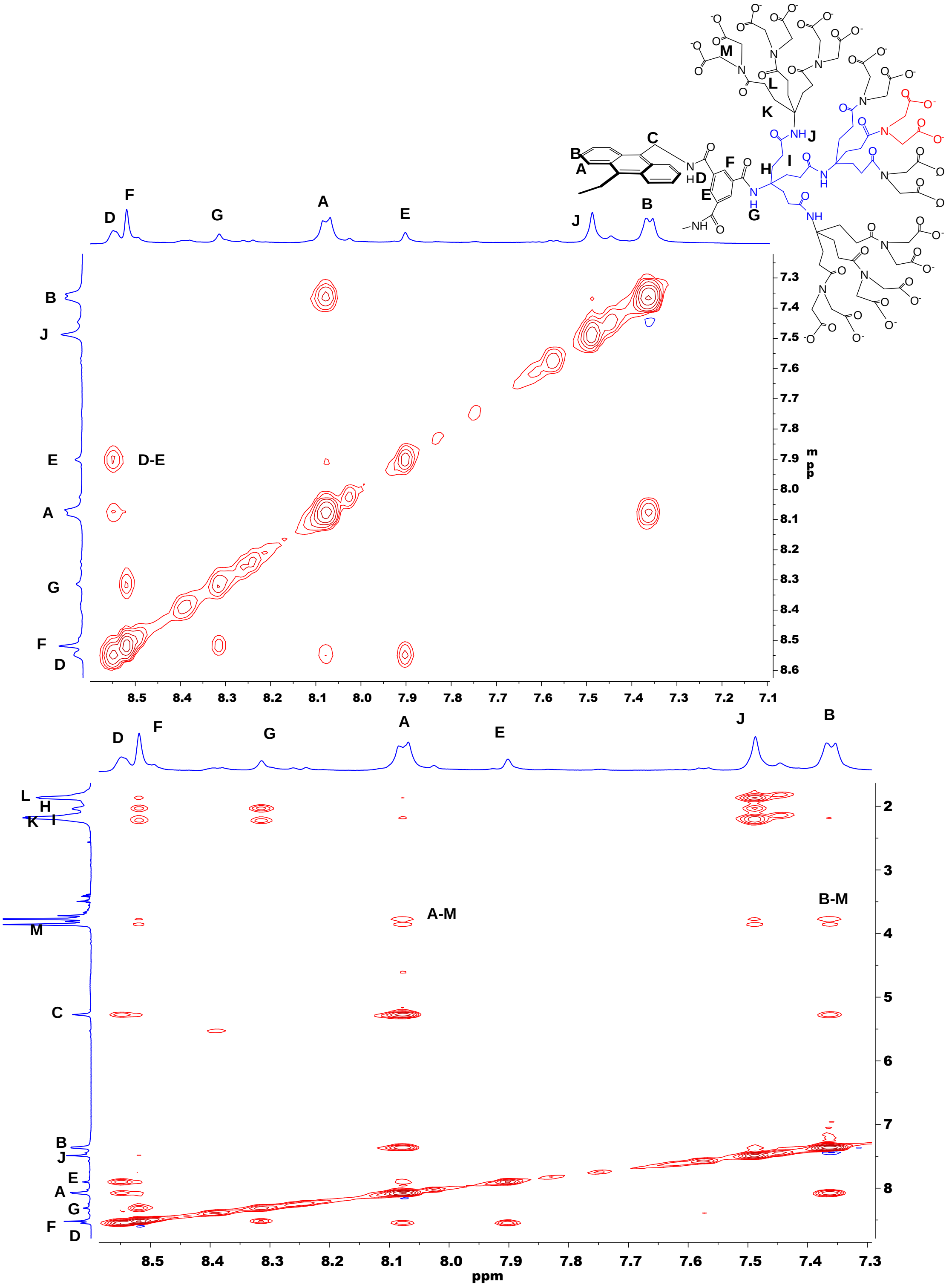

Figure S38. NOESY spectrum (600 MHz, mixing time: 250 ms) of receptor **8** (2.68 mM) in D_2O/H_2O (v/v = 1/9) at 298 K. Connections between side-chain CH_2 protons M and anthracene protons A/B show that the side-chain termini can approach the entrance to the cavity. However, no connection is observed between M and E, implying that the side-chain remains outside the cavity. Note that protons M show as two signals due slow rotation about the tertiary amide CO-N bond.

Receptor 8 + glucosamine

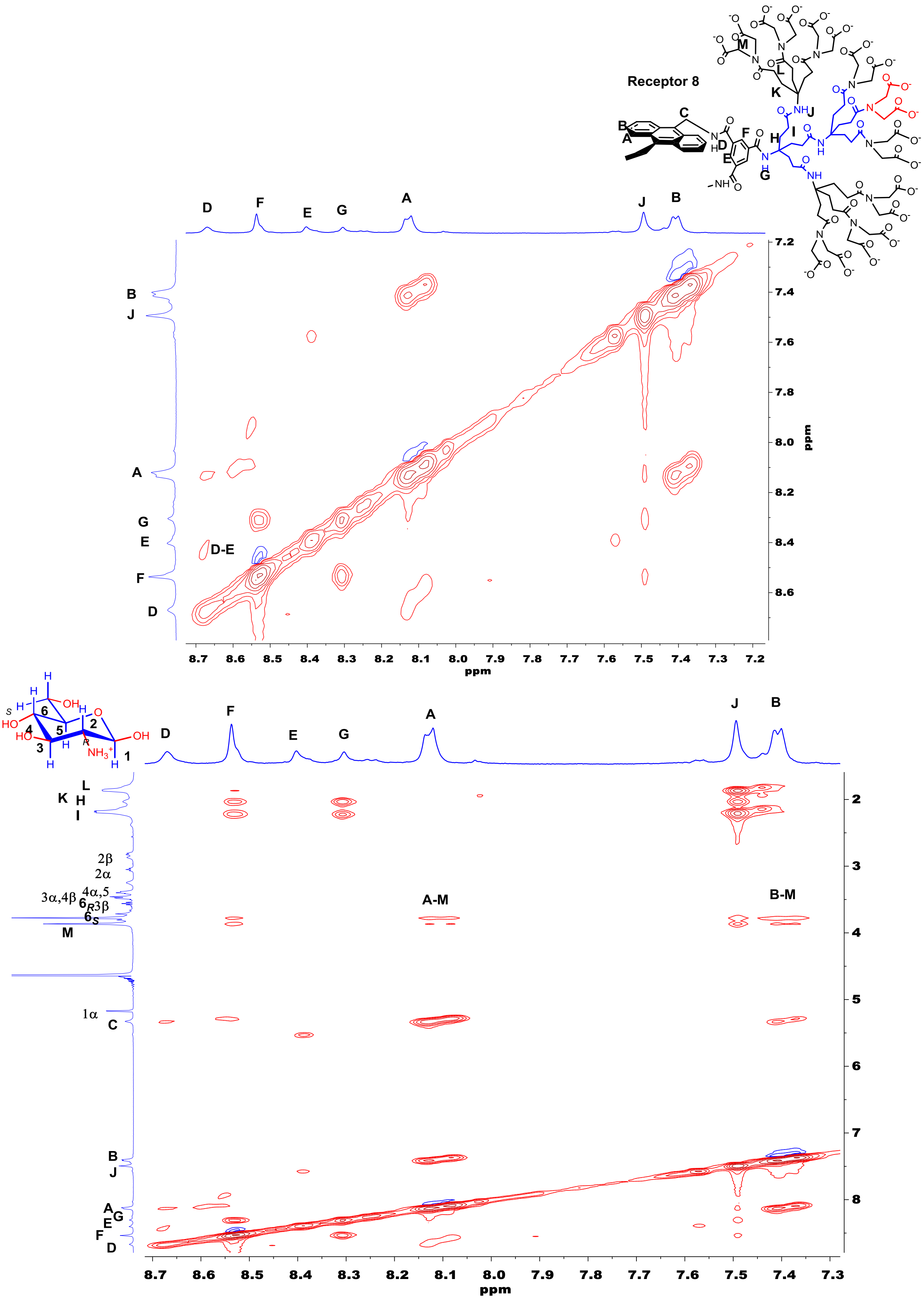

Figure S39. NOESY spectrum (600 MHz, mixing time: 250 ms) of Receptor **8** (1.89 mM) + D-glucosamine (8.04 mM) in D_2O/H_2O (v/v = 1/9) at 298 K. The connections between side-chain CH_2 protons M and anthracene protons A/B are still present.

Receptor 9

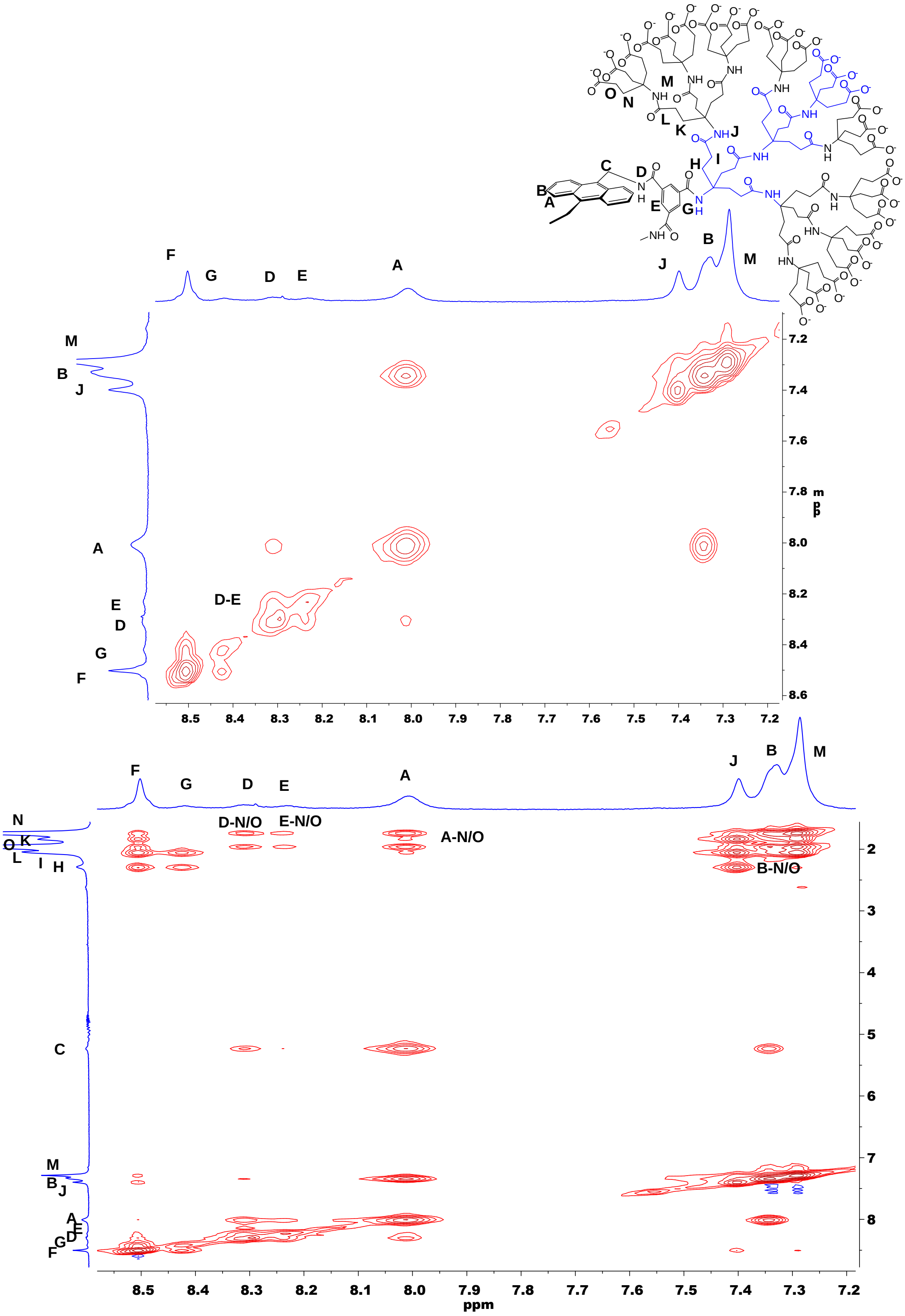

Figure S40. NOESY spectrum (600 MHz, mixing time: 250 ms) of receptor **9** (0.87 mM) in D_2O/H_2O (v/v = 1/9) at 298 K. Connections are observed between side-chain protons N/O and internally-directed protons D/E, implying that the ends of the side-chains can enter the cavity.

4. Binding Studies

^{1}H NMR titration experiments

^{1}H NMR titrations were performed on either a 500 or 600 MHz Varian spectrometer at 298 K. Stock solutions of carbohydrate guests were made up in D_2O the night before the experiment and left at RT to ensure the equilibration of anomers. A solution of host, typically 50-200 µM concentration, was placed in an NMR tube and host was also added at this concentration to the guest solution (to keep the concentration of host constant throughout the experiment). Aliquots of guest solution were added to the NMR tube and the ^{1}H NMR spectra recorded after each addition.

^{1}H NMR titrations with glucosamine were performed under three sets of conditions: (a) minimum ionic strength (no added NaCl), (b) 20 mM NaCl, (c) 154 mM NaCl.

(a) *Minimum ionic strength.* A stock solution (D_2O) of glucosamine hydrochloride was neutralised to pH = 7 with careful addition of NaOD in D_2O (~0.13 equiv), then left overnight at RT to ensure the equilibration of anomers. This solution was then used to perform the binding study as above. For these experiments the pH of the host solution was checked and confirmed to be 7 before the titration, as was the pH of the mixture in the NMR tube after the titration. Note that the salt concentrations increase slightly during these titrations due to NaCl present in the titrant. For further discussion of this issue see below under “Control of pH and salt concentrations”.

(b/c) *20 mM or 154 mM NaCl*. The experiment was performed as described under (a) above, except that NaCl was added to both titrant and titrand solutions to achieve the desired concentrations. Note that slightly less NaCl was required for the solution containing the glucosamine, as this solution already contained a small amount of NaCl from the neutralisation (pH = 7, ~0.13 equiv NaOD). The concentration of NaCl was thus held constant throughout these titrations. The experiments on galactosamine at 154 mM NaCl were performed similarly.

The association constants were calculated by entering the change in shift (ppm) of aromatic proton E into a specifically written non-linear least squares fitting program within Excel. This calculates the K_a and the limiting change in chemical shift Δδ assuming a 1:1 binding stoichiometry. Good fits were generally observed between experimental and predicted data, supporting the assumption of 1:1 stoichiometry. The programme also calculates errors as standard deviations for K_a values calculated from individual data points employing the limiting Δδ.

Fluorescence titration experiments

Fluorescence titration experiments were carried out at 298 K on a PerkinElmer LS45 spectrometer in quartz cuvette (3 mL, 10 mm path length).

Titrations with glucose were performed in PBS buffer solution (pH = 7.1 , 100 mM). Titrations with glucosamine hydrochloride were performed in water at pH = 7; the pH was controlled and checked as described above for the ^{1}H NMR titrations. Additions to receptor were performed using a procedure which kept [host] and the total volume constant while raising [guest]. Thus, receptor was added to a stock carbohydrate solution to give [host]. This solution was used as titrant. A solution of host was placed in a fluorescence cell containing a magnetic stir bar. For each addition, an aliquot of a certain volume was removed from the cell, and the same volume of titrant was then added. After each addition the solution was stirred for 2 min and left standing for 1 min before the fluorescence spectrum was recorded. The excitation wavelength was chosen at 394 nm, and the emission spectrum recorded from 400 -550 nm.

ITC titration experiments

ITC experiments were performed at 298 K. Stock solutions of carbohydrates were made up in HPLC grade water and allowed to equilibrate overnight. Receptor solutions were made up in HPLC water. The sample cell volume was 0.2005 mL. Each titration experiment included 25-40 successive injections.

Control of pH and salt concentrations

As described above the titrations with aminosugars were performed at constant pH = 7 without the addition of buffer. It was expected that this should be possible because of the many carboxylic acid groups in the dendritic side chains. These should show a range pK_a values from ~5 upwards and should therefore act as internal buffers, reducing the sensitivity of the solutions to adventitious acid/base. To confirm this was the case, a pH titration was performed of receptor **6** vs. NaOH. The results are shown in Fig. S41. The slope of the titration curve at pH = 7 is still quite shallow, demonstrating the buffering effect of the side-chains. For comparison, the plot from a similar titration against acetic acid is also shown (Fig. S41 inset). As expected, in this case the pH moves rapidly through 7 as NaOH is added.

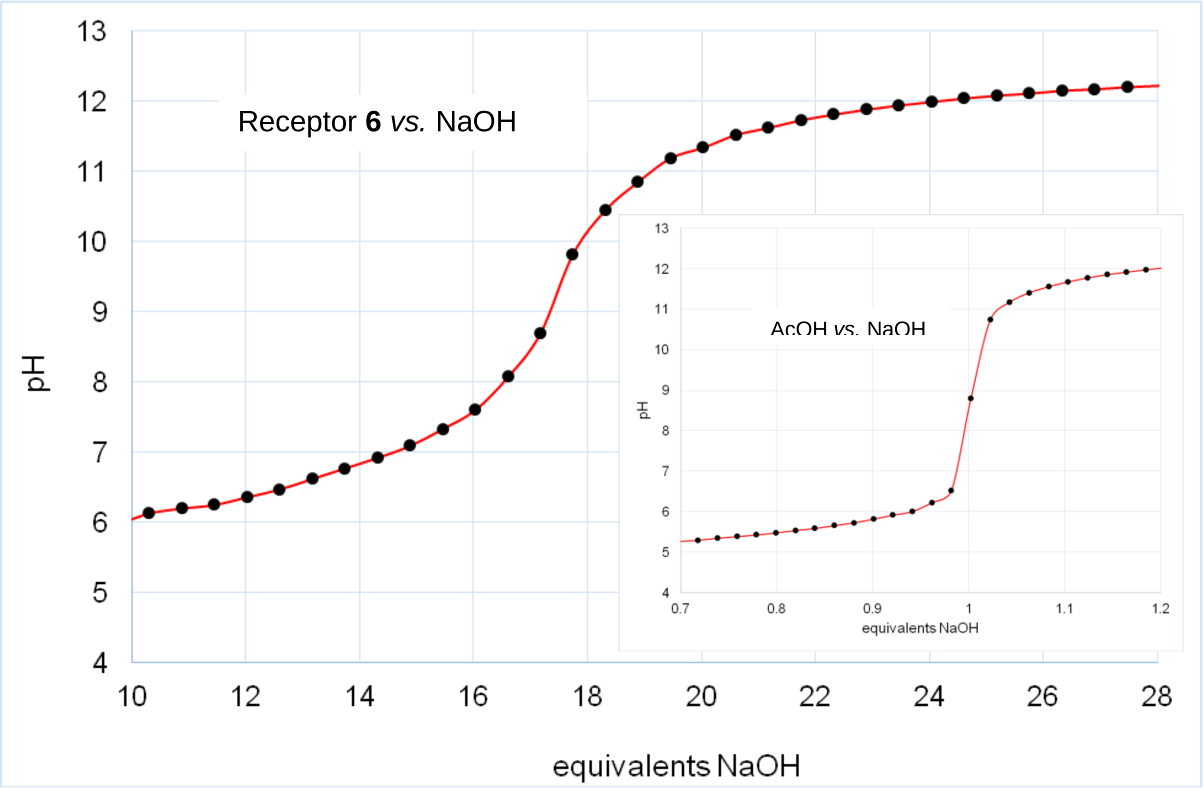

Figure S41. pH Titration of receptor **6** (3.4 μM) vs. NaOH. The amount of added NaOH is shown in equivalents relative to **6**. At pH = 7 ~14.5 equivalents have been added, implying that implying that ~3.5 carboxylic acid groups remain. The slope of the curve is still shallow at this point, reflecting the buffering effect of the unionised CO_2H groups. *Inset:* A comparison titration against AcOH. In this case the slope is much steeper at pH = 7.

Control of salt concentration was also significant for the titrations of aminosugars. For conditions with added NaCl (b/c; see above), this could be achieved exactly. For experiments at minimum ionic strength [conditions (a)], some increase in salt concentration could not be avoided because of the NaOH used to neutralise the hydrochloride substrate. However, the proportional increases tended to be small, especially where high binding constants allowed the determination of binding constants using small amounts of titrant. For example, in the case of **8** + glucosamine, neutralisation of the host contributes ~2.9 mM Na^+ to the titrand and titrant solutions. Because of the high binding constant (7000 M^{-1}) the titration can be stopped at ~2 mM substrate without compromising accuracy, at which point ~0.26 mM Na^+ has been added. $[Na^+]$ thus increases by ~9%. This small change does not seem likely to affect the validity of the titration. Indeed, as shown in the Figures below, the titrations with glucosamine generally give excellent fits. They do not show the distortions expected if the binding constants were changing significantly during the experiments.

Table of results

The full set of binding data is given in Table S1 below. In addition to the results given in the main paper, Table S1 includes binding constants to disaccharide substrates (maltose, cellobiose, lactose) and to glucose in the presence of NaCl.

Table S1. Data from measurements of binding constants to carbohydrates in aqueous solution[a]

Receptor:	2	4	5	6	8	9
Length of side-chain[b] (overall charge)	7 (-6)	8 (-12)	10 (-12)	10 (-18)	13 (-36)	15 (-54)
	Association Constant K_a (M^{-1})					
D-Glucosamine **13**[c]	160[d]	1400 (1500[e])	2000 (1700[e])	2400 (2100[e])	7000 (9700[e])	610
D-Glucosamine **13** (NaCl 20 mM)[c]	-[f]	330	420	690	1660	151
D-Glucosamine **13** (NaCl 154 mM)[c]	-[f]	97 (76[g])	135	222	340	53
D-Galactosamine **14** (NaCl 154 mM)[c]	-[f]	-[h]	27	33	98	4
D-Glucose **3**	56 (55[g], 58[e])	70 (65[g], 75[e])	89 (91[e])	90 (81[h], 87[e])	69 (41[e])	4 (6[e])
D-Glucose **3** (NaCl 154 mM)	-[f]	-[f]	-[f]	89	-[f]	-[f]
Methyl β-D-glucoside	96 (101[g], 121[e])	87 (87[g])	124	115 (120[g])	92	-[f]
N-Acetyl-D-glucosamine **15**	9	19	25	31	33	-[f]
D-Galactose	4[d]	6	6	7	3	-[f]
D-Mannose	~0[i]	~0[i]	~0[i]	~0[i]	~0[i]	-[f]
D-Maltose	36	41	45	49	41	-[f]
D-Cellobiose	27	29	38	29	35	-[f]
D-Lactose	16	23	27	31	27	-[f]
	Limiting Fluorescence Change (F/F_0)[j]					
D-Glucose	2.5	3.7	3.4	3.7	2.0	2.2

[a] Association constants K_a were measured by ^1H NMR titration in D_2O at 298 K unless otherwise noted. Data for **2** are from ref [3] unless otherwise noted. [b] Atoms from C1 outwards. [c] pH = 7. [d] Measured/remeasured as part of the present work. [e] Measured by fluorescence titration in H_2O. [f] Not determined. At these salt concentrations receptor **2** gives broadened ^1H NMR spectra, presumably due to aggregation. [g] Measured by ITC in H_2O. [h] Poor fit to 1:1 binding model, suggesting multiple stoichiometries. [i] Signal movements almost linear with concentration. [j] Emission 423 nm, excitation at 395 nm.

Receptor 2 Carbohydrate Binding Studies

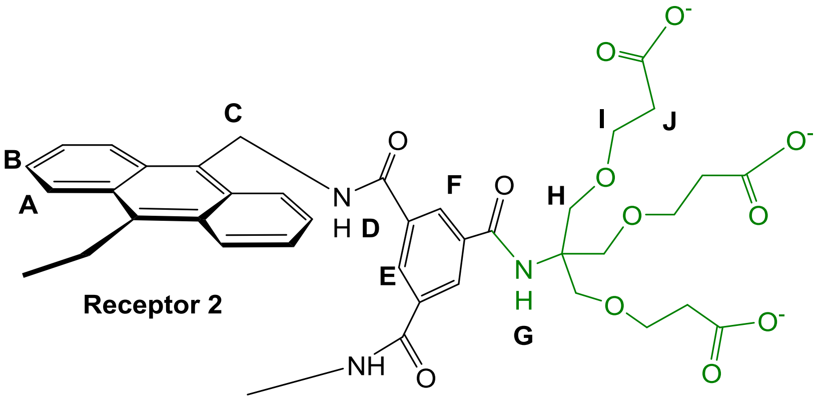

NMR spectra and binding analyses

D-Glucosamine 13

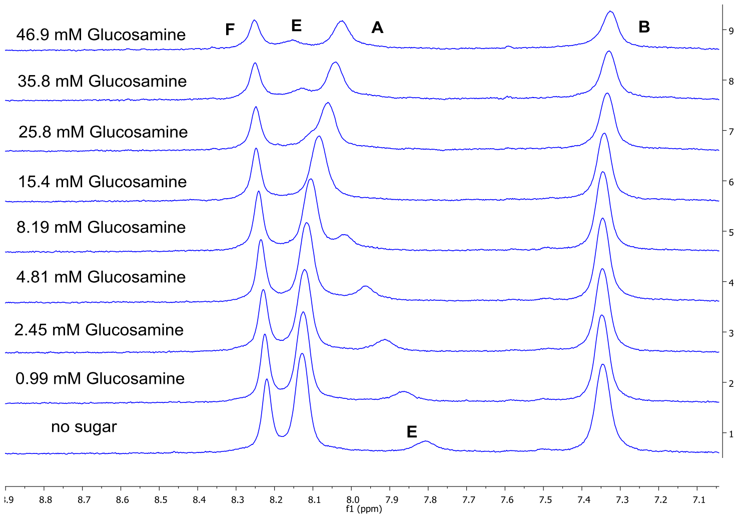

Figure S42. Partial H NMR spectra from the titration of receptor **2** (1 mM) with D-glucosamine **13** in D_2O (pH = 7) at 298 K.

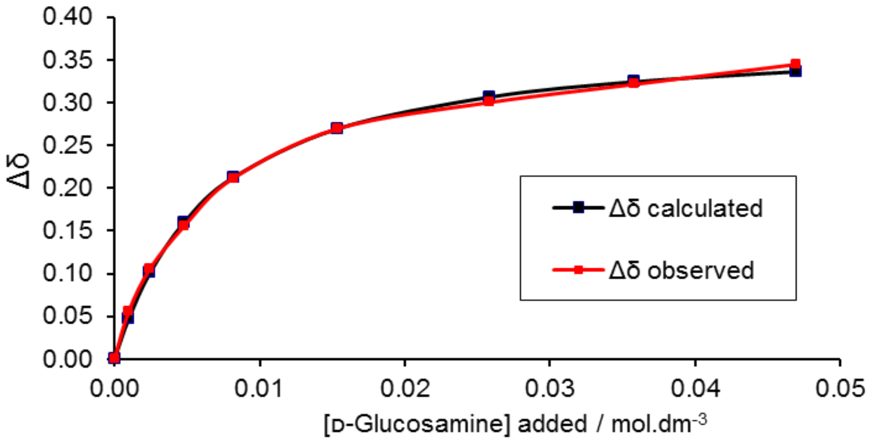

Figure S43. Experimental and calculated values for the NMR binding study of receptor **2** (1.00 mM) with D-glucosamine **13** in D_2O (pH = 7) at 298 K. Proton E: K_a = 165 M^{-1} ± 13%, Δδ = 0.38

Receptor 4 Carbohydrate Binding Studies

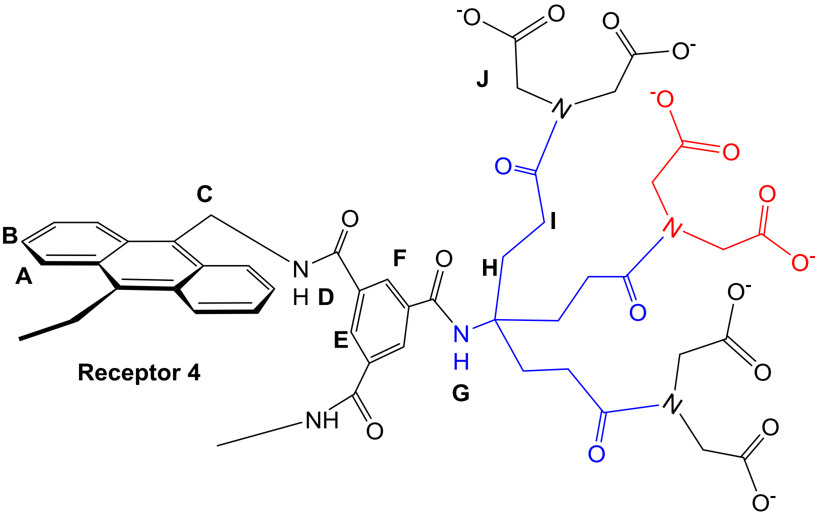

Receptor 4

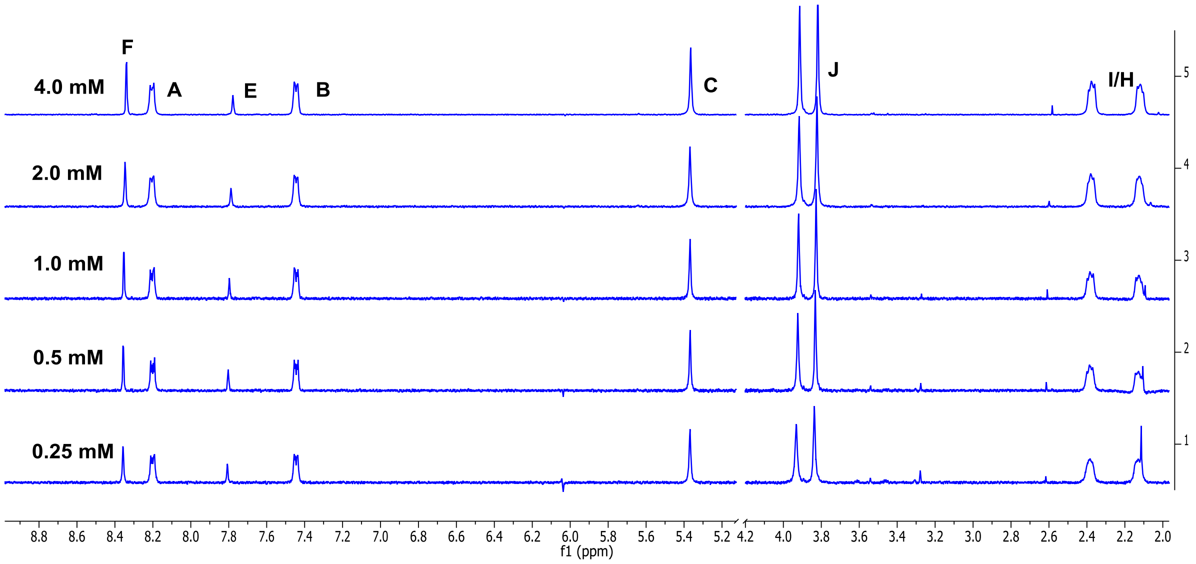

Figure S44. Partial ^{1}H NMR spectra of receptor **4** at concentrations from 0.25 mM to 4 mM in D_2O at 298 K, with assignments.

NMR spectra and binding analyses

D-Glucose 3

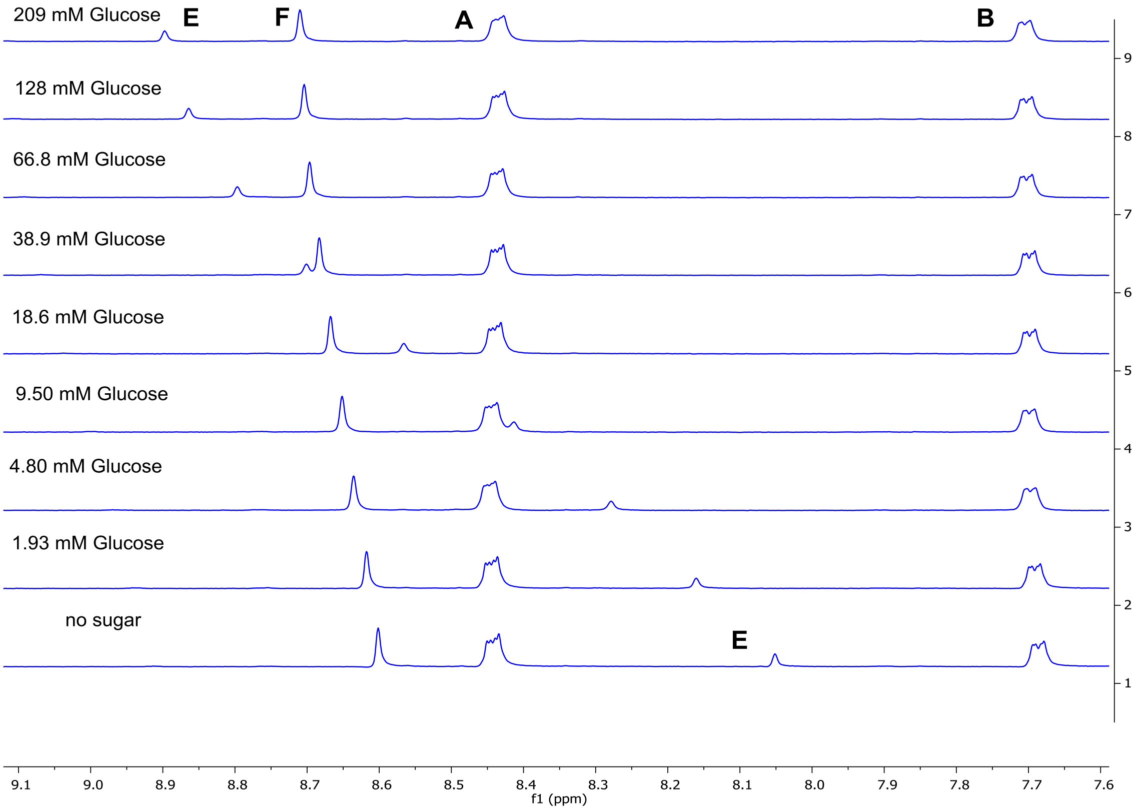

Figure S45. Partial ^{1}H NMR spectra from the titration of receptor **4** (0.18 mM) with D-glucose **3** in D_2O at 298 K.

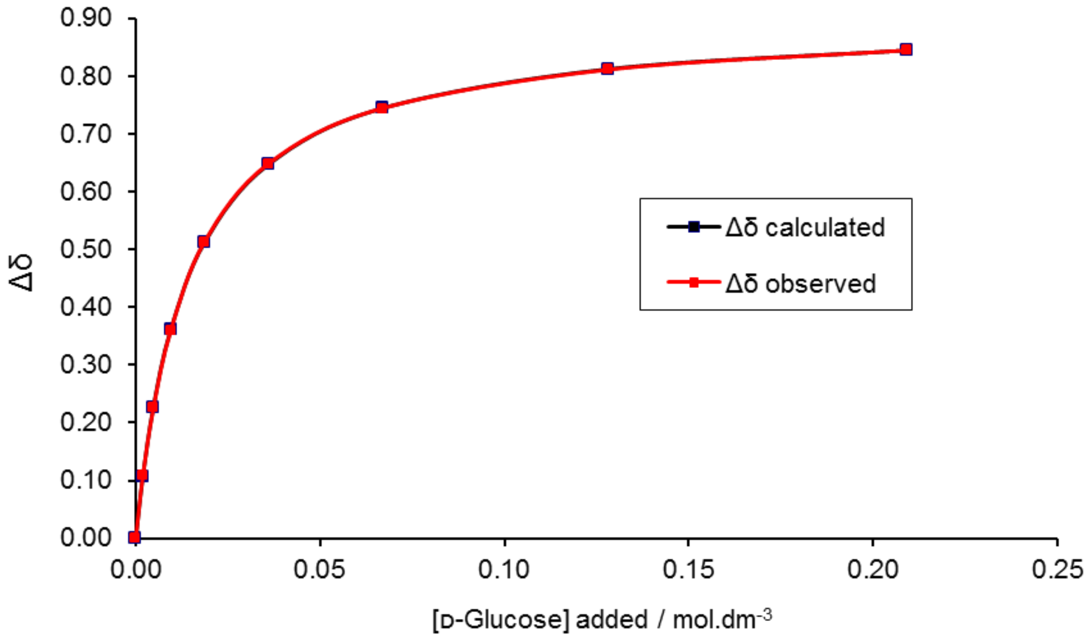

Figure S46. Experimental and calculated values for the NMR binding study of receptor **4** (0.18 mM) with D-glucose in D_2O at 298 K. Proton E: K_a = 70 M^{-1} ± 1%, Δδ = 0.90.

Methyl β-D-glucoside

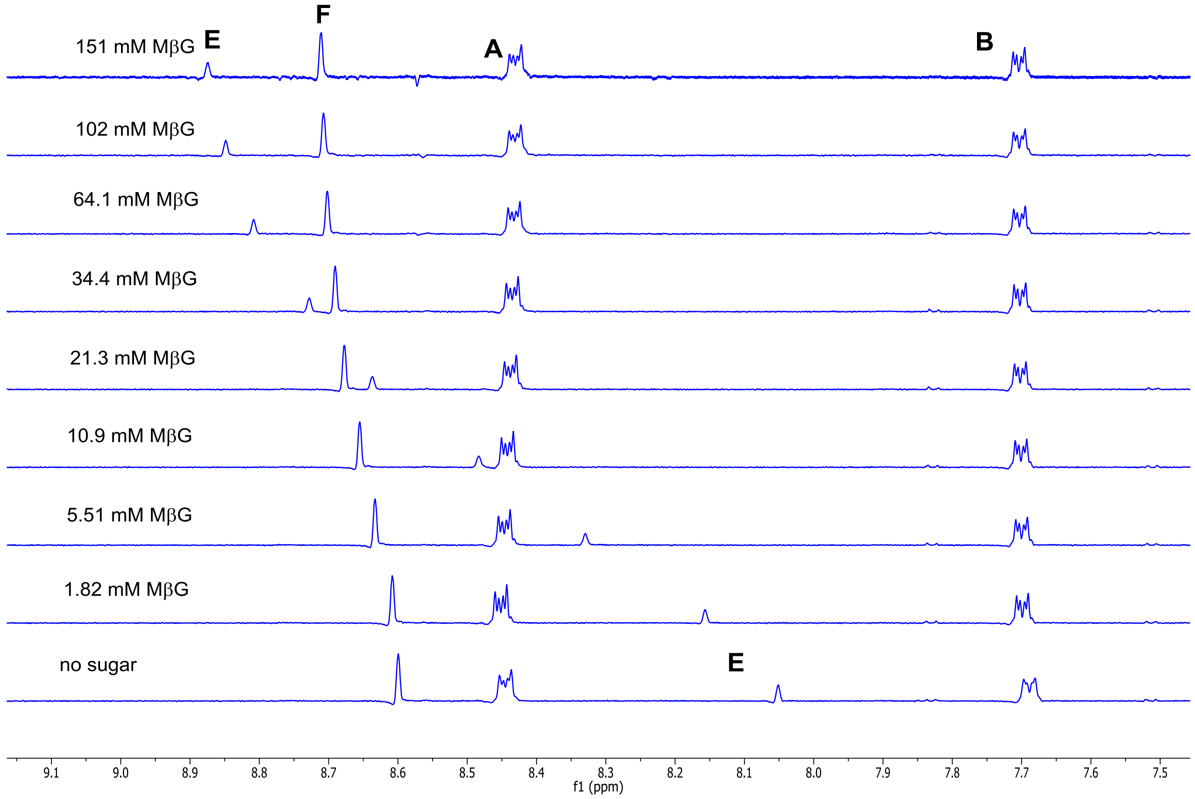

Figure S47. Partial ^{1}H NMR spectra from the titration of receptor **4** (0.21 mM) with methyl β-D-glucoside in D_2O at 298 K.

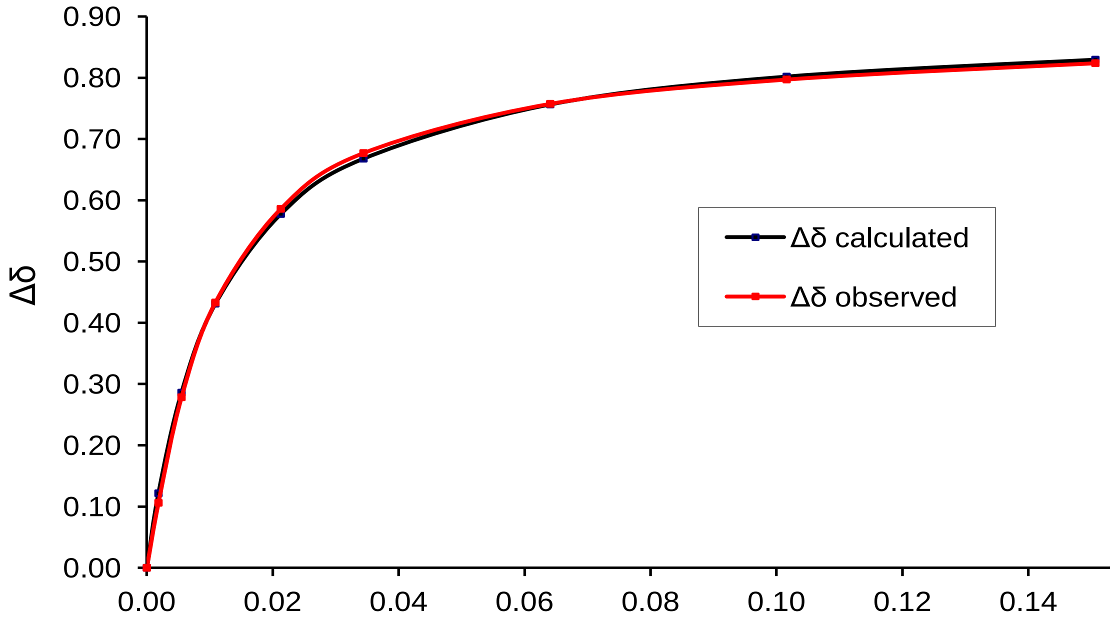

Figure S48. Experimental and calculated values for the NMR binding study of receptor **4** (0.21) with methyl β-D-glucoside in D_2O at 298 K. Proton E: K_a = 87 M^{-1} ± 6%, Δδ = 0.89.

N-Acetyl-D-glucosamine 15

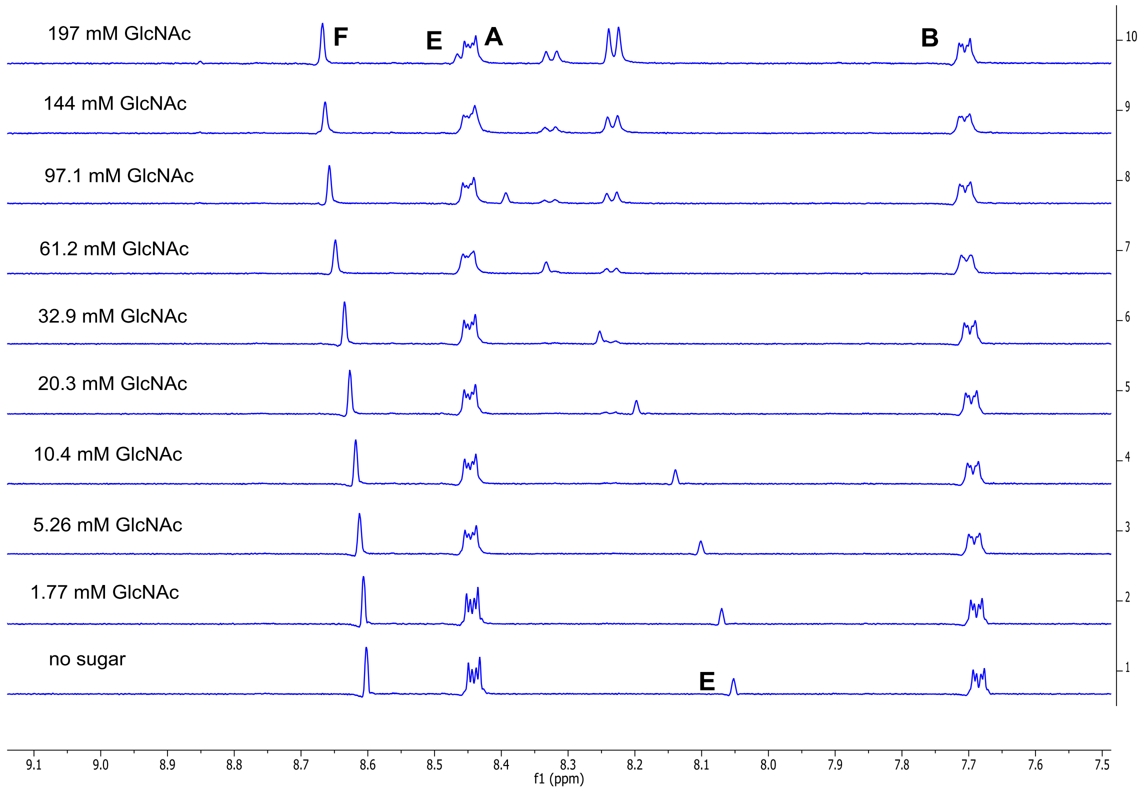

Figure S49. Partial ^{1}H NMR spectra from the titration of receptor **4** (210 µM) with GlcNAc **15** in D_2O at 298 K.

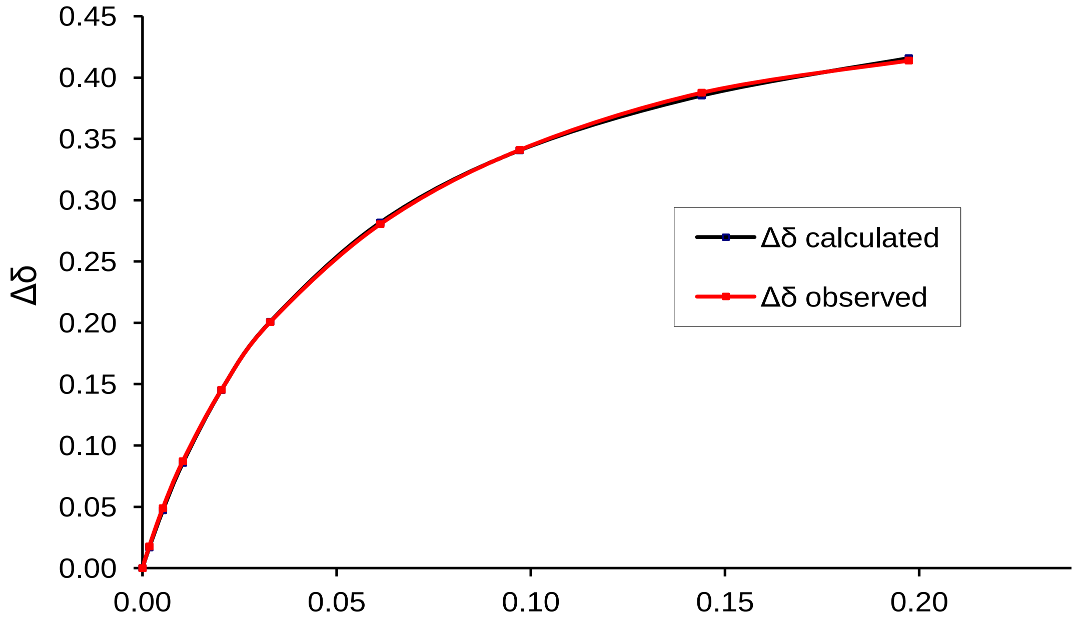

Figure S50. Experimental and calculated values for the NMR binding study of receptor **4** (210 µM) with GlcNAc **15** in D_2O at 298 K. Proton E: K_a = 19 M^{-1} ± 2%, Δδ = 0.53.

D-Galactose

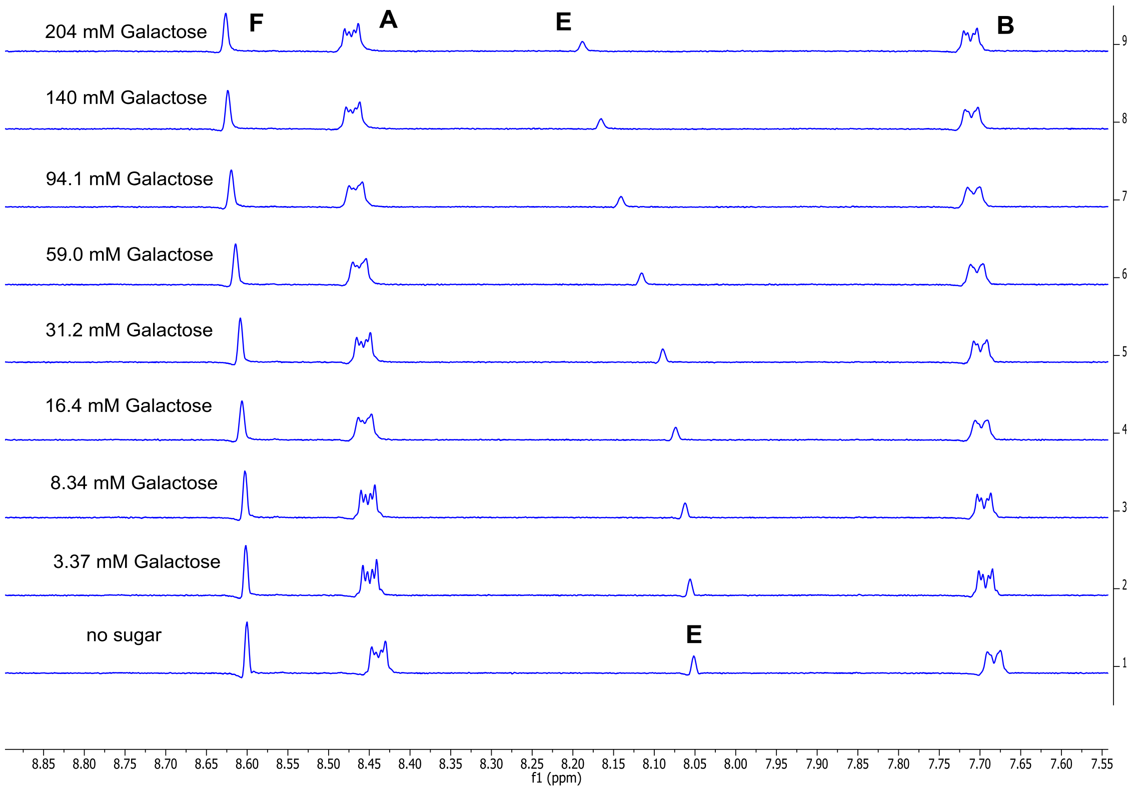

Figure S51. Partial ^{1}H NMR spectra from the titration of receptor **4** (210 µM) with D-galactose in D_2O at 298 K.

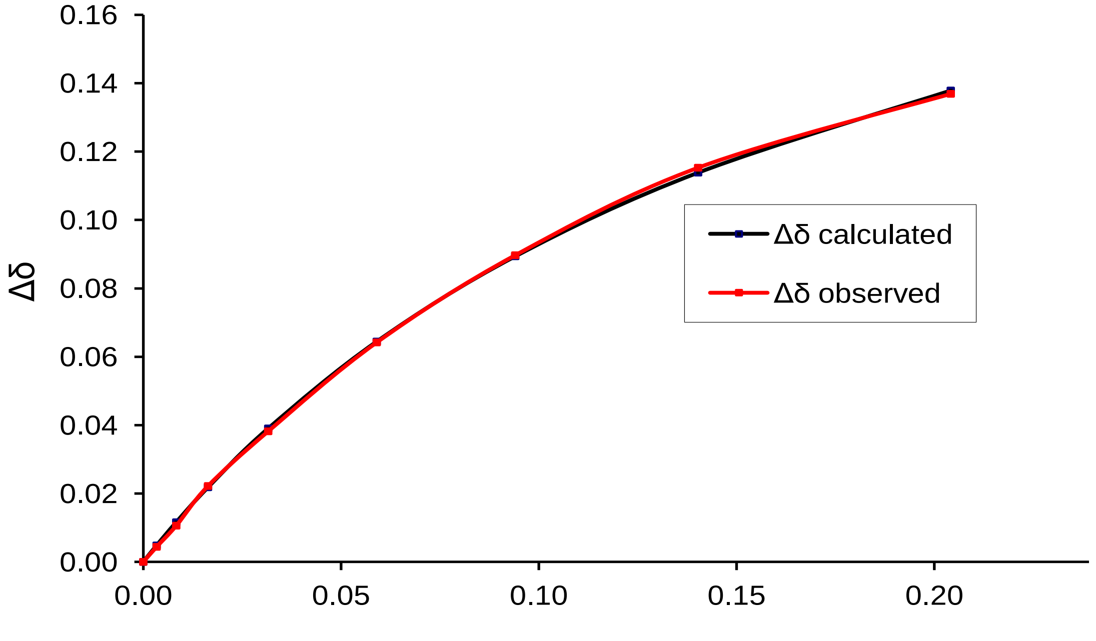

Figure S52. Experimental and calculated values for the NMR binding study of receptor **4** (210 µM) with D-galactose in D_2O at 298 K. Proton E: K_a = 5.7 M^{-1} ± 4%, Δδ = 0.26.

D-Mannose

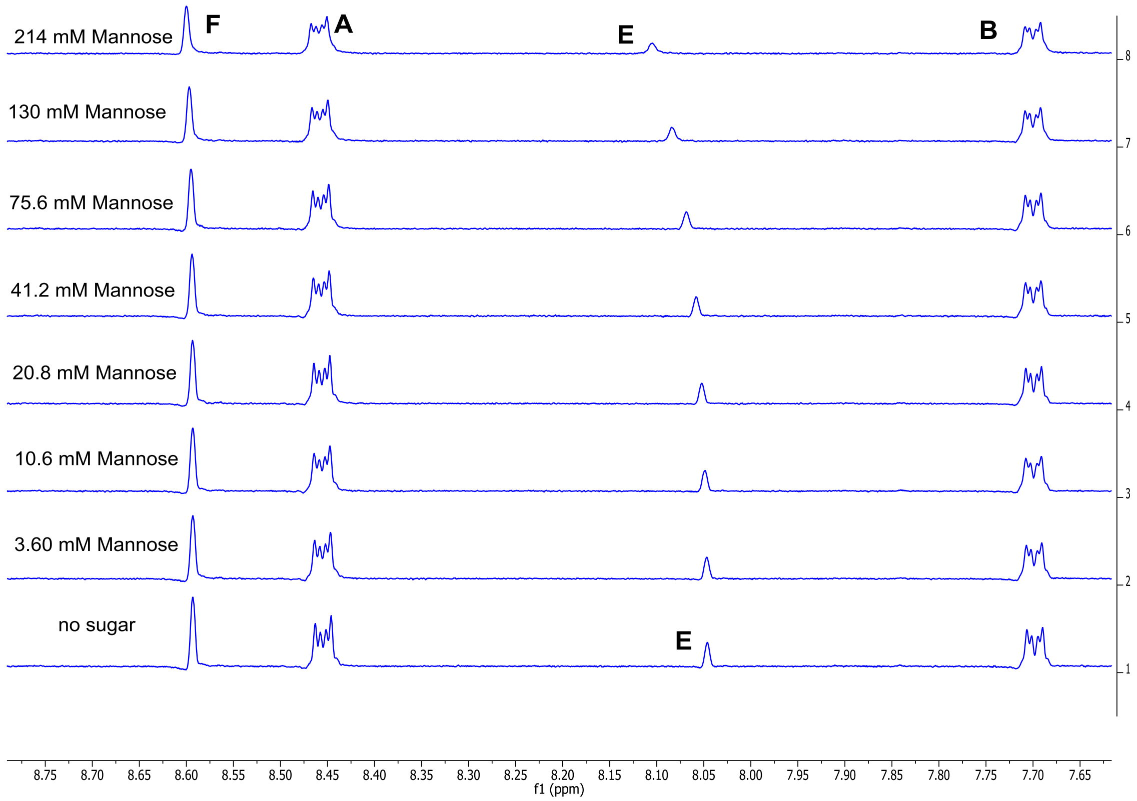

Figure S53. Partial ^{1}H NMR spectra from the titration of receptor **4** (210 µM) with mannose in D_2O at 298 K.

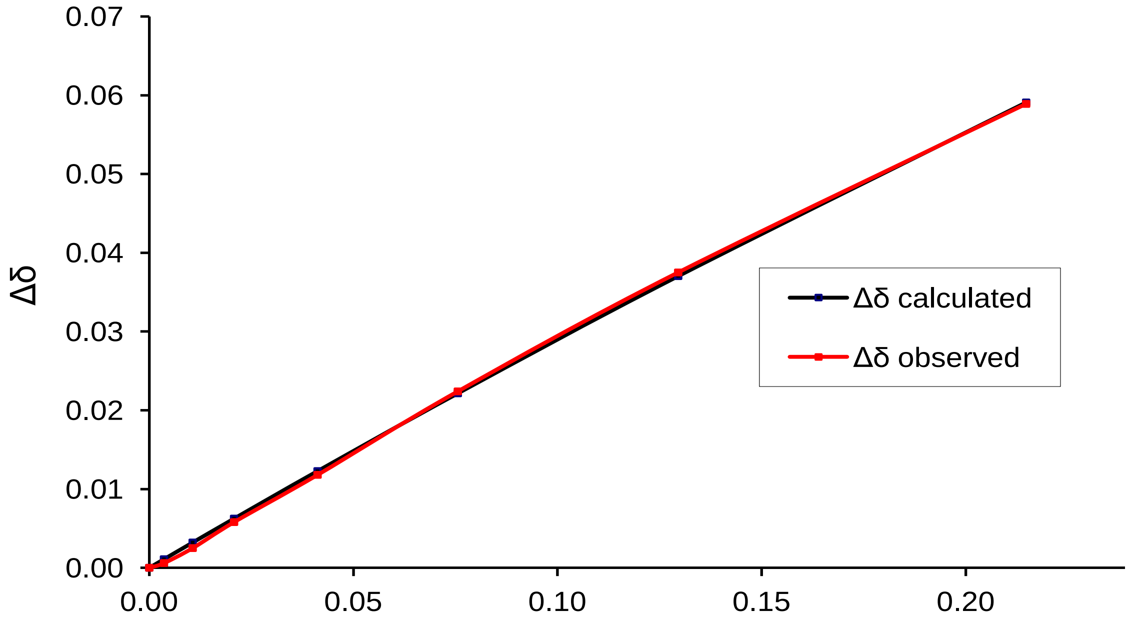

Figure S54. Experimental and calculated values for the NMR binding study of receptor **4** (210 µM) with mannose in D_2O at 298 K. Proton E: K_a too small to be evaluated with reasonable accuracy.

D-Maltose

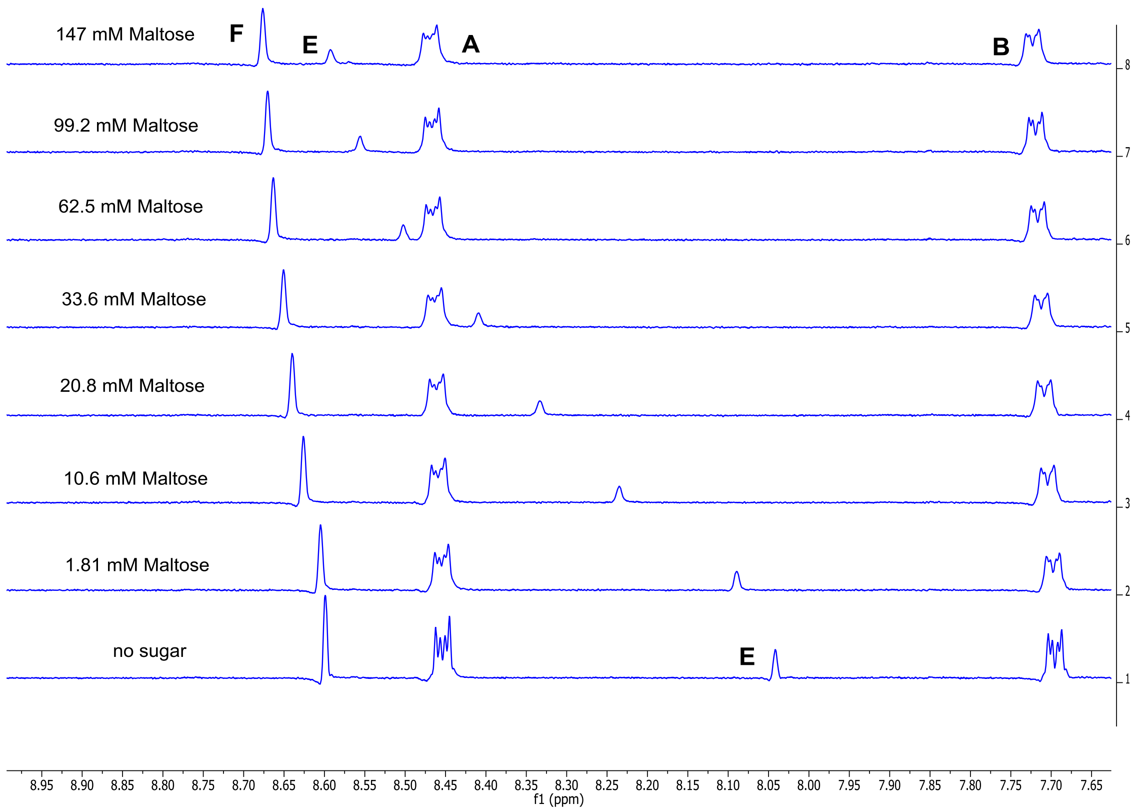

Figure S55. Partial ^{1}H NMR spectra from the titration of receptor **4** (210 µM) with D-maltose in D_2O at 298 K.

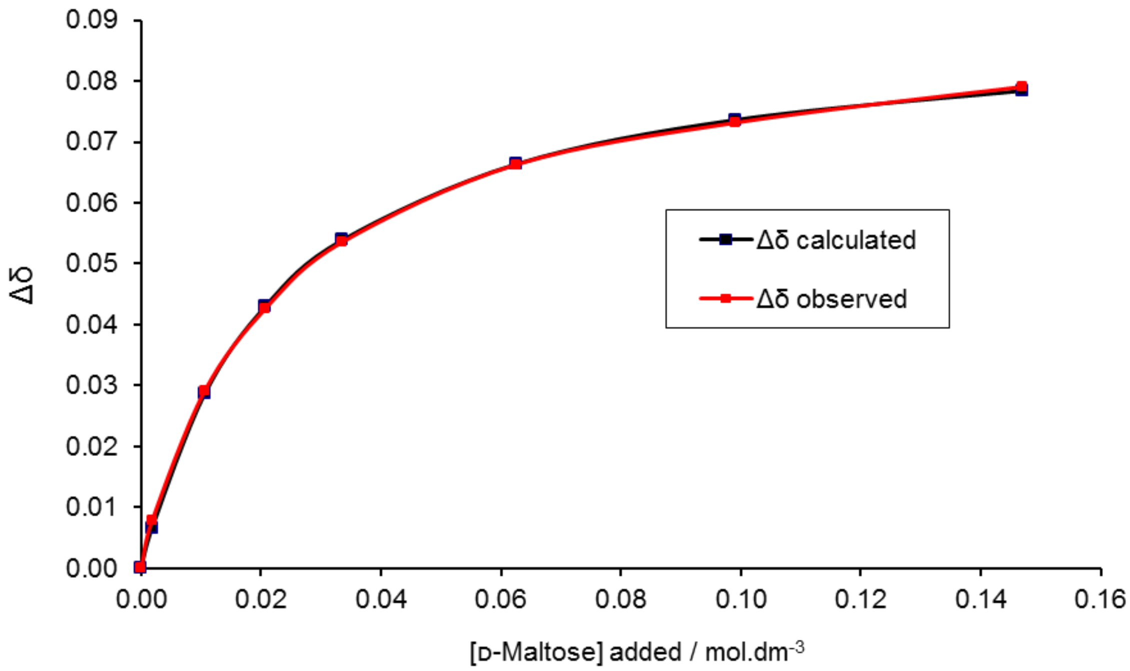

Figure S56. Experimental and calculated values for the NMR binding study of receptor **4** (0.21 mM) with D-lactose in D_2O at 298 K. Proton E: K_a = 41 M^{-1} ± 5%, Δδ = 0.64.

D-Cellobiose

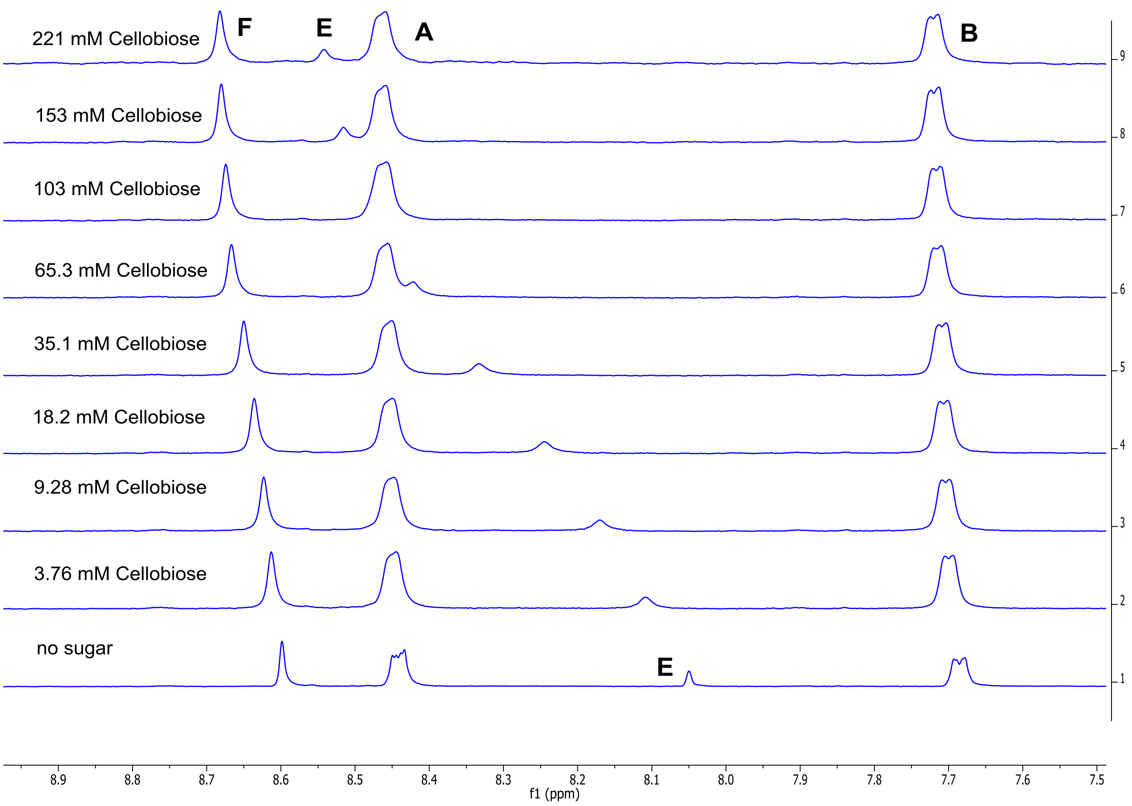

Figure S57. Partial ^{1}H NMR spectra from the titration of receptor **4** (0.21 mM) with D-cellobiose in D_2O at 298 K.

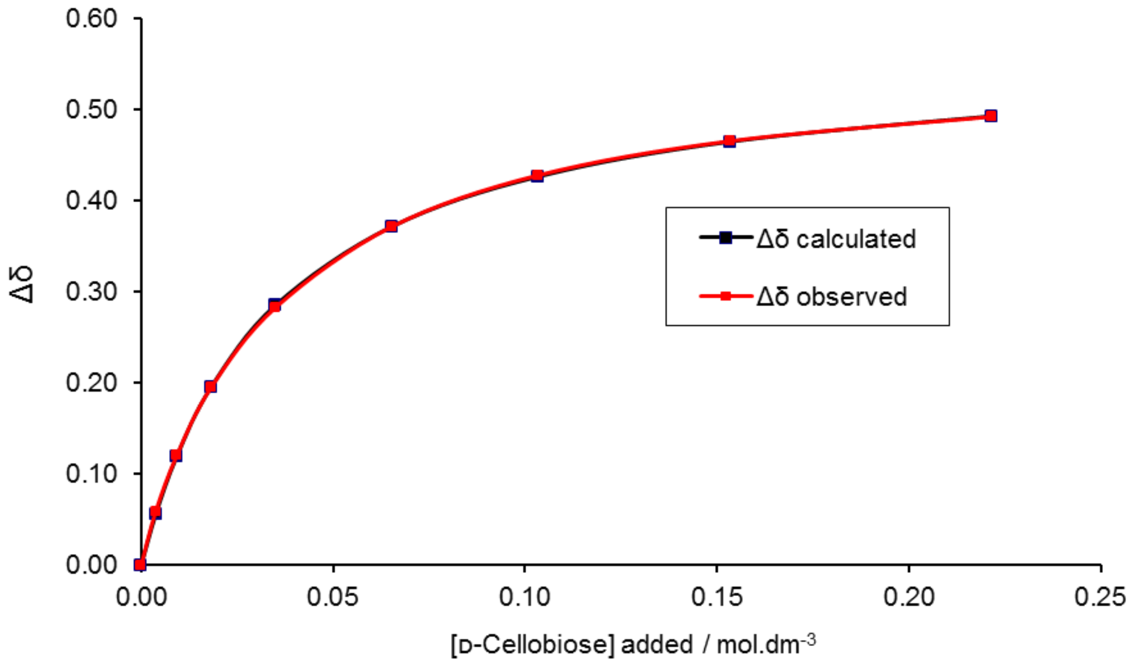

Figure S58. Experimental and calculated values for the NMR binding study of receptor **4** (0.21 mM) with D-cellobiose in D_2O at 298 K. Proton E: K_a = 29 M^{-1} ± 2%, Δδ = 0.57.

D-Lactose

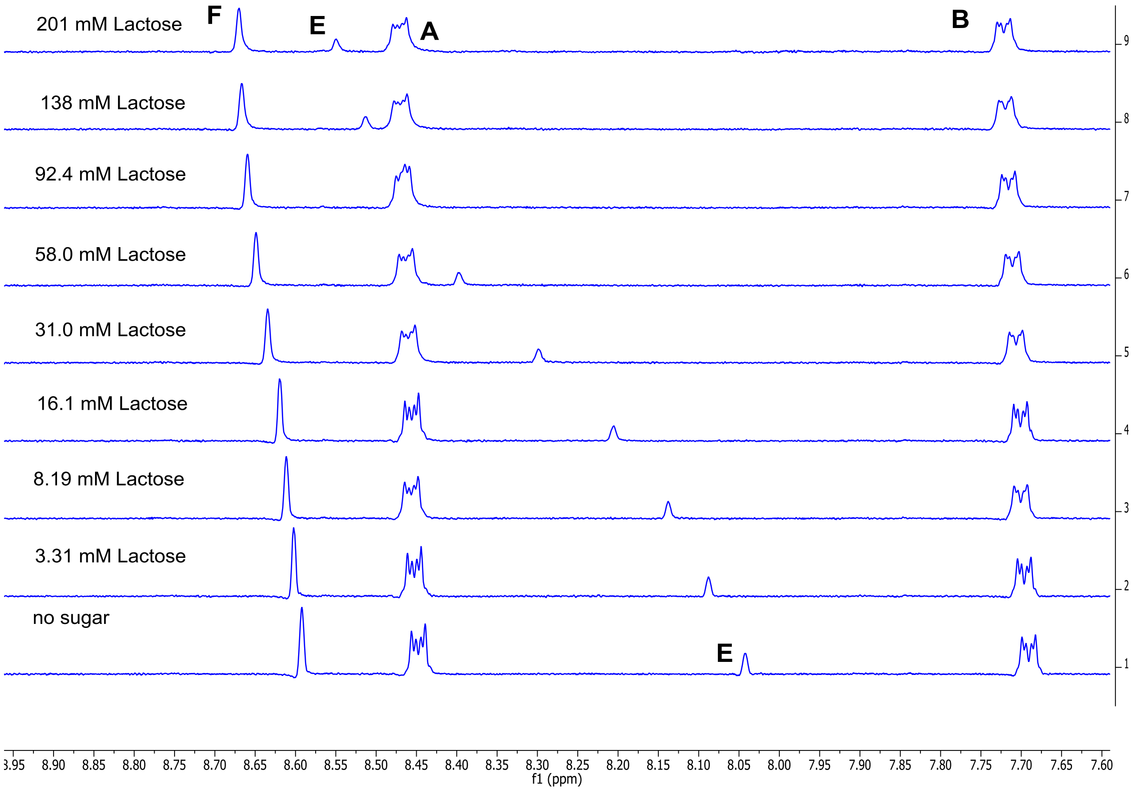

Figure S59. Partial ^{1}H NMR spectra from the titration of receptor **4** (0.21 mM) with D-lactose in D_2O at 298 K.

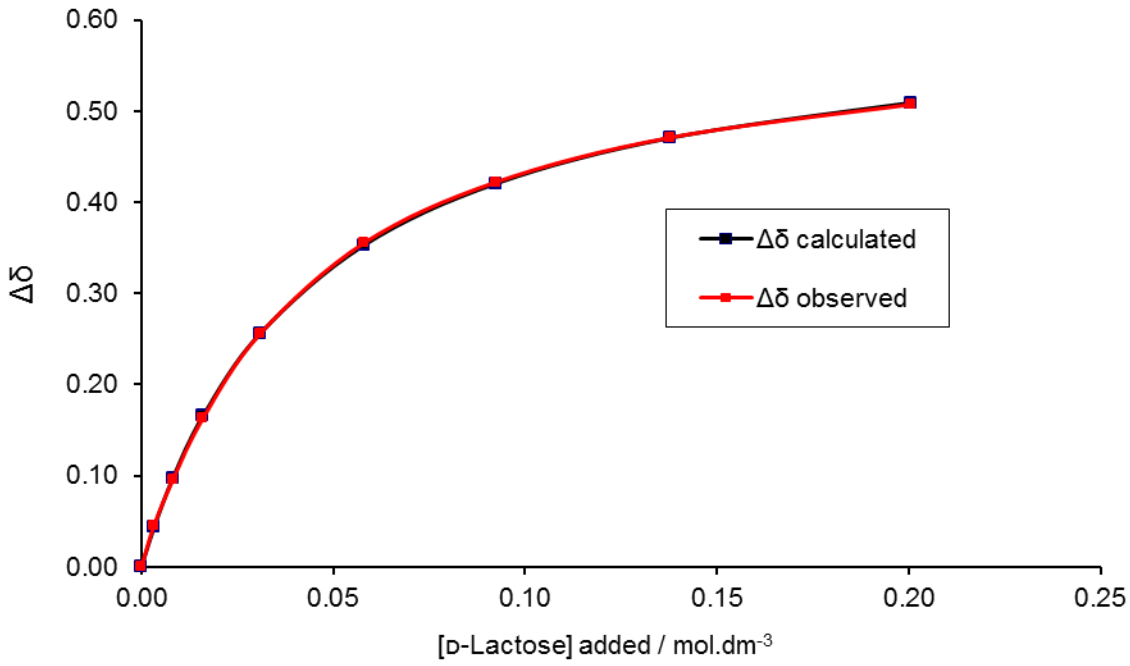

Figure S60. Experimental and calculated values for the NMR binding study of receptor **4** (0.21 mM) with D-lactose in D_2O at 298 K. Proton E: K_a = 23 M^{-1} ± 2%, Δδ = 0.62.

D-Glucosamine 13

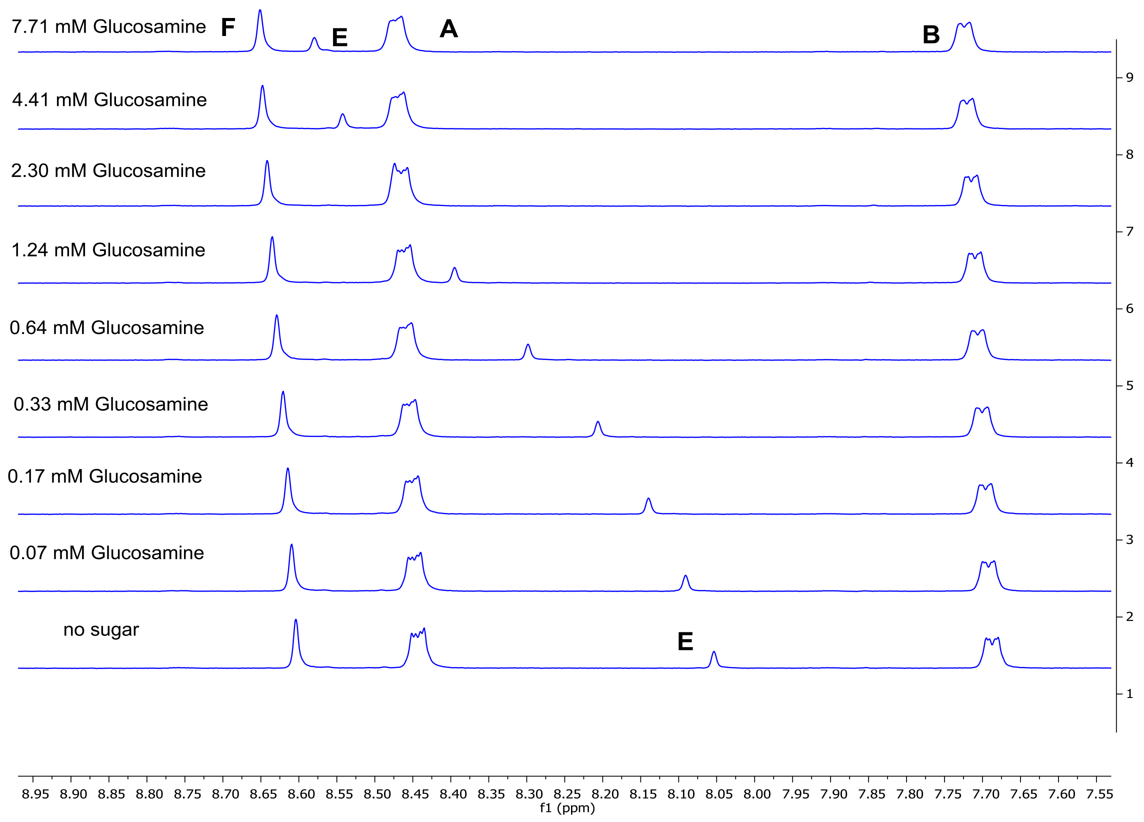

Figure S61. Partial ^{1}H NMR spectra from the titration of receptor **4** (0.20 mM) with D-glucosamine **13** in D_2O (pH = 7) at 298 K.

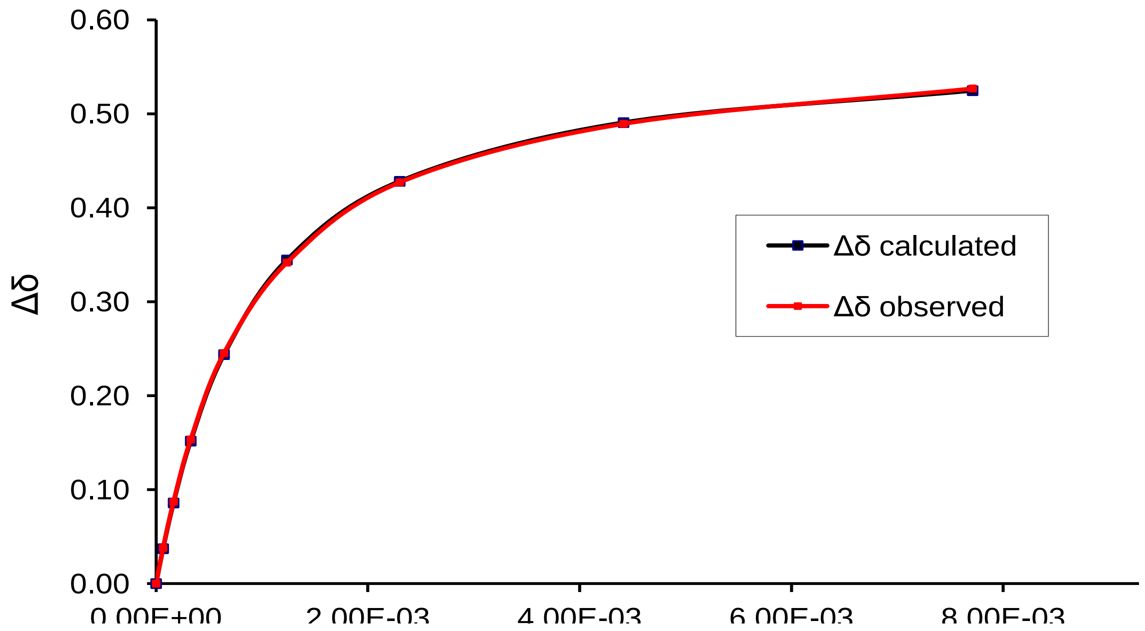

Figure S62. Experimental and calculated values for the NMR binding study of receptor **4** (0.20 mM) with D-glucosamine **13** in D_2O (pH = 7) at 298 K. Proton E: K_a = 1400 M^{-1} ± 2%, Δδ = 0.57.

D-Glucosamine (NaCl 20 mM)

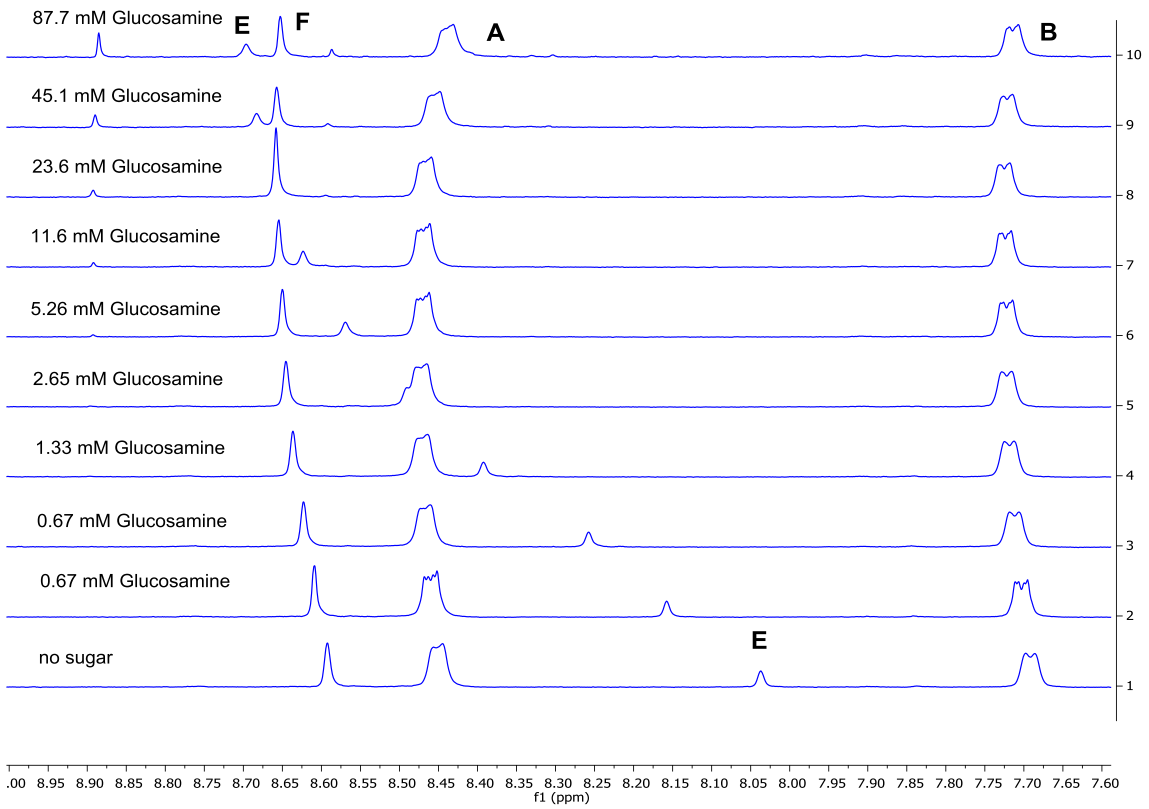

Figure S63. Partial ^{1}H NMR spectra from the titration of receptor **4** (0.18 mM) with D-glucosamine **13** in D_2O (pH = 7, 20 mM, NaCl) at 298 K.

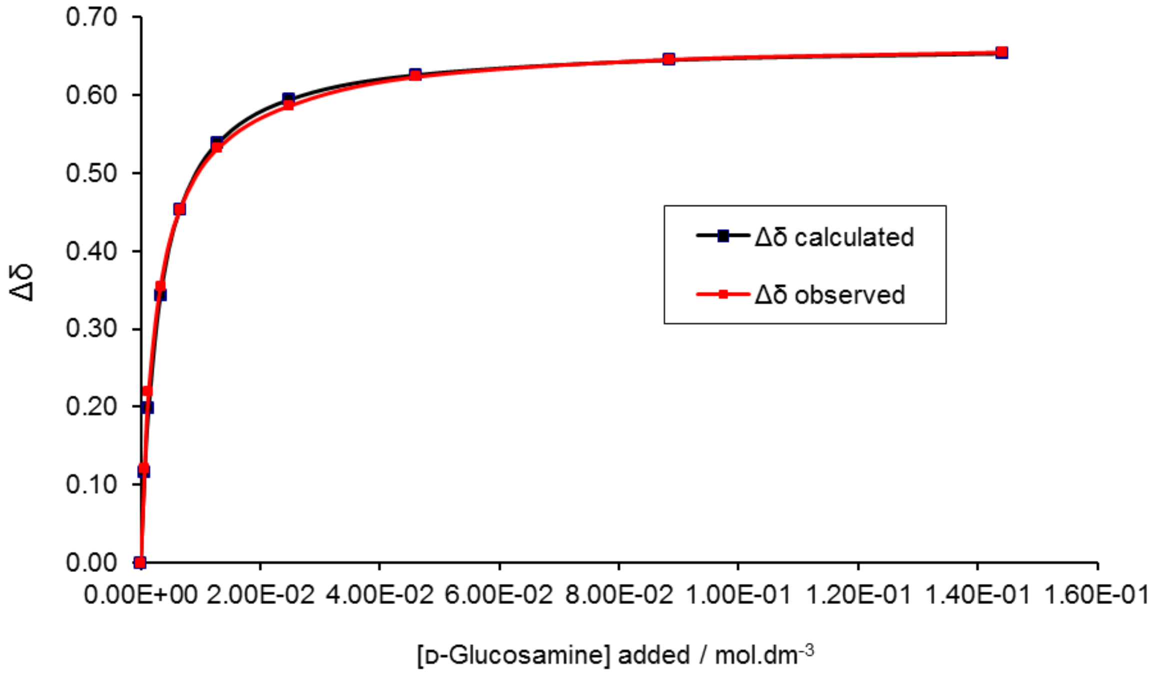

Figure S64. Experimental and calculated values for the NMR binding study of receptor **4** (0.18 mM) with D-glucosamine **13** in D_2O (pH = 7, 20 mM, NaCl) at 298 K. Proton E: K_a = 330 M^{-1} ± 8%, $\Delta\delta$ = 0.67.

ᴅ-Glucosamine (NaCl 154 mM)

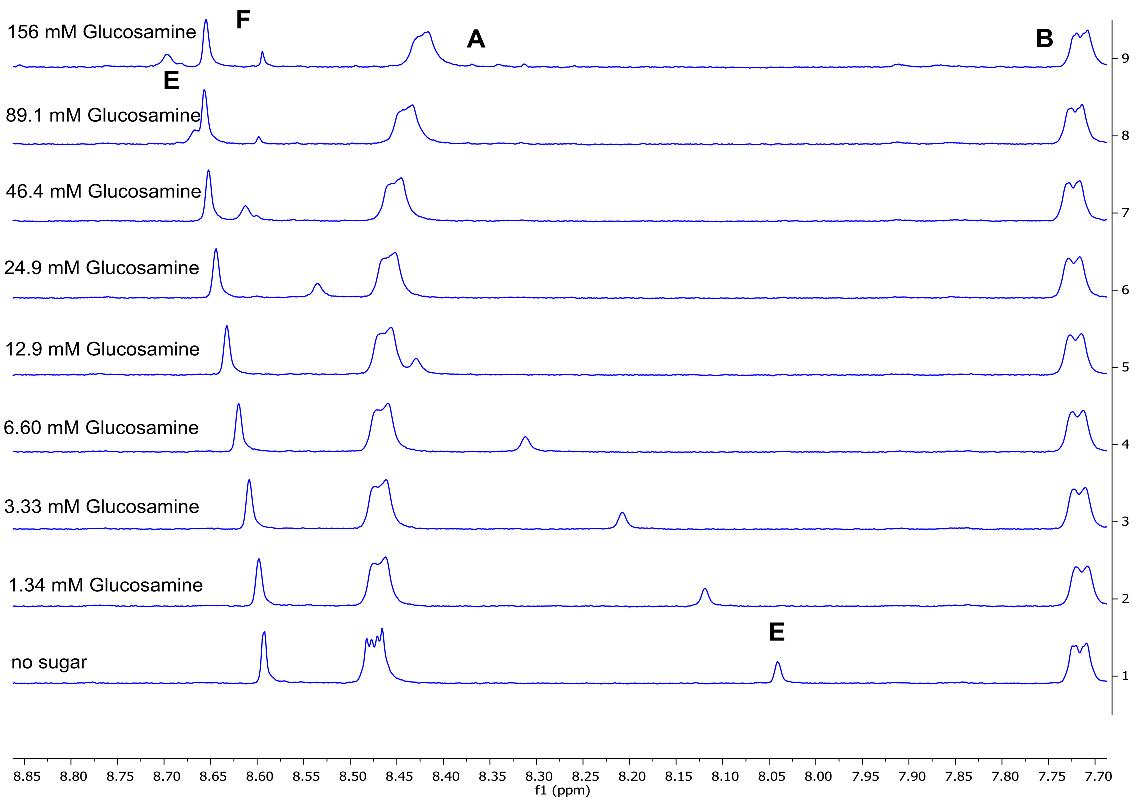

Figure S65. Partial ^{1}H NMR spectra from the titration of receptor **4** (0.18 mM) with D-glucosamine **13** in D_2O (pH = 7, 154 mM, NaCl) at 298 K.

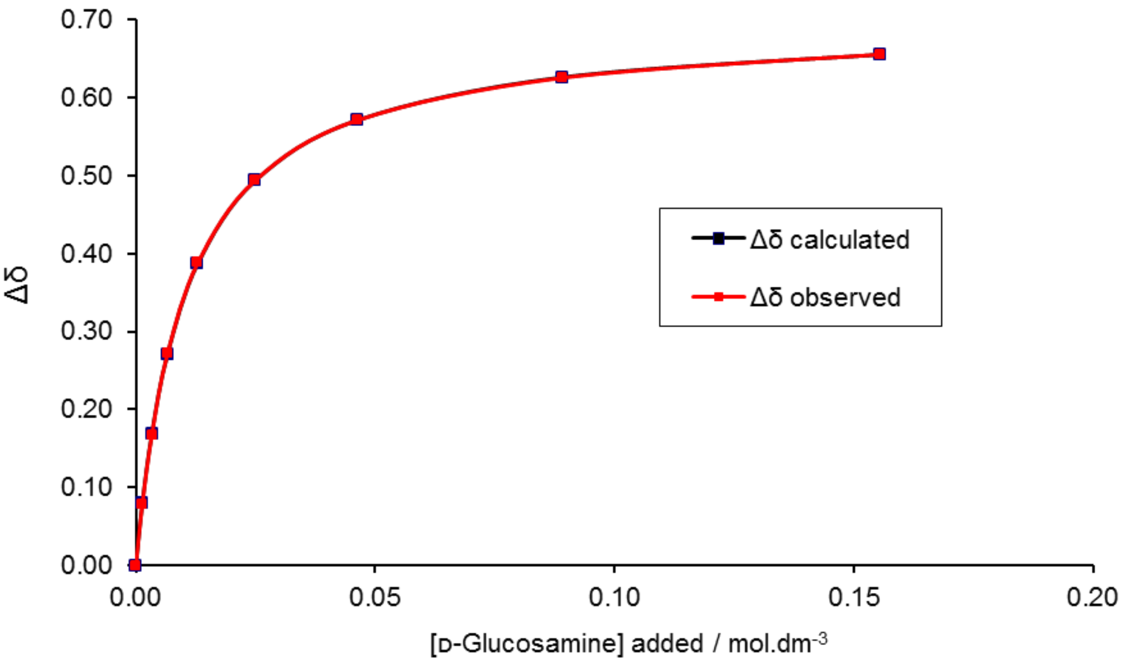

Figure S66. Experimental and calculated values for the NMR binding study of receptor **4** (0.18 mM) with D-glucosamine **13** in D_2O (pH = 7, 154 mM, NaCl) at 298 K. Proton E: K_a = 97 M^{-1} ± 1%, Δδ = 0.70.

D-Galactosamine (NaCl 154 mM)

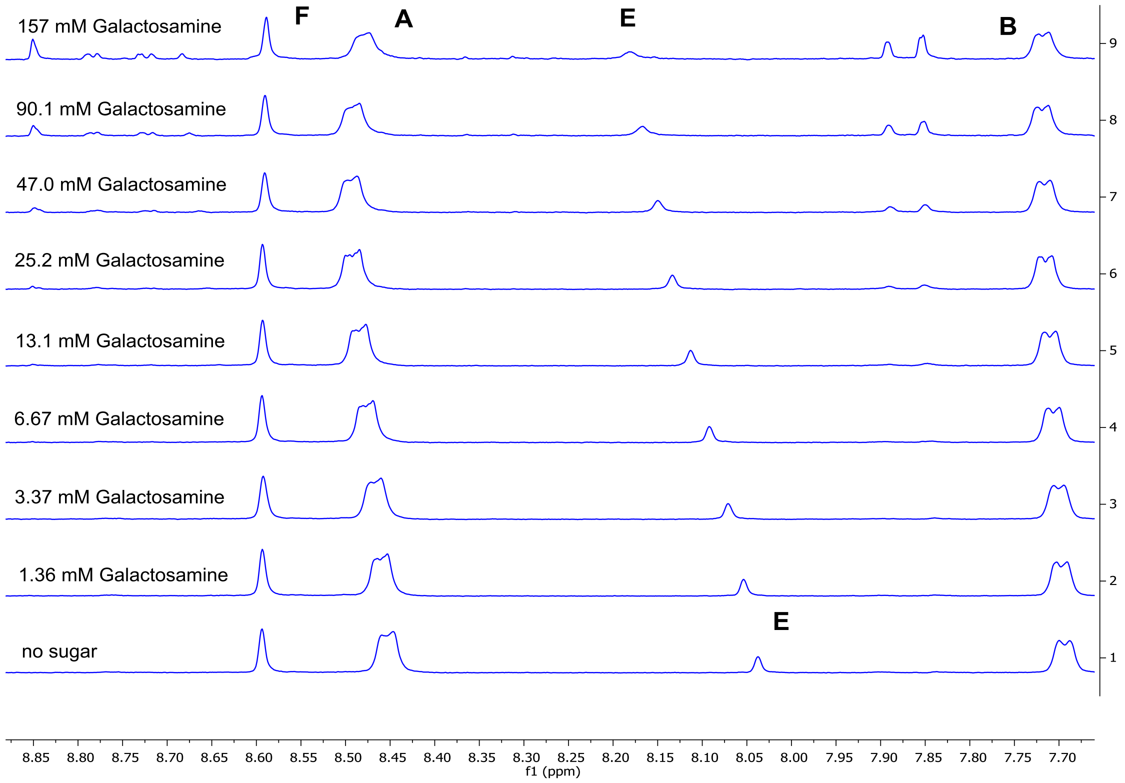

Figure S67. Partial ^{1}H NMR spectra from the titration of receptor **4** (0.18 mM) with D-galactosamine in D_2O (pH = 7, 154 mM, NaCl) at 298 K.

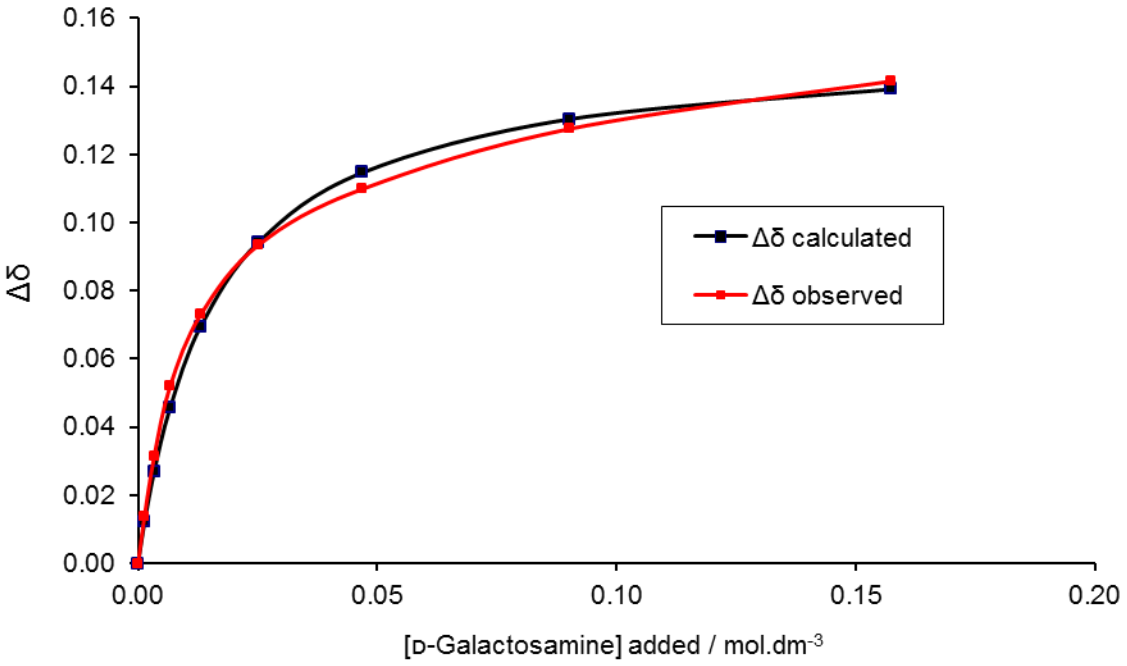

Figure S68. Experimental and calculated values for the NMR binding study of receptor **4** (0.18 mM) with D-galactosamine in D_2O (pH = 7, 154 mM, NaCl) at 298 K. Proton E: K_a = 64 M^{-1} ± 15%, Δδ = 0.15

ITC Titrations

D-Glucose 3

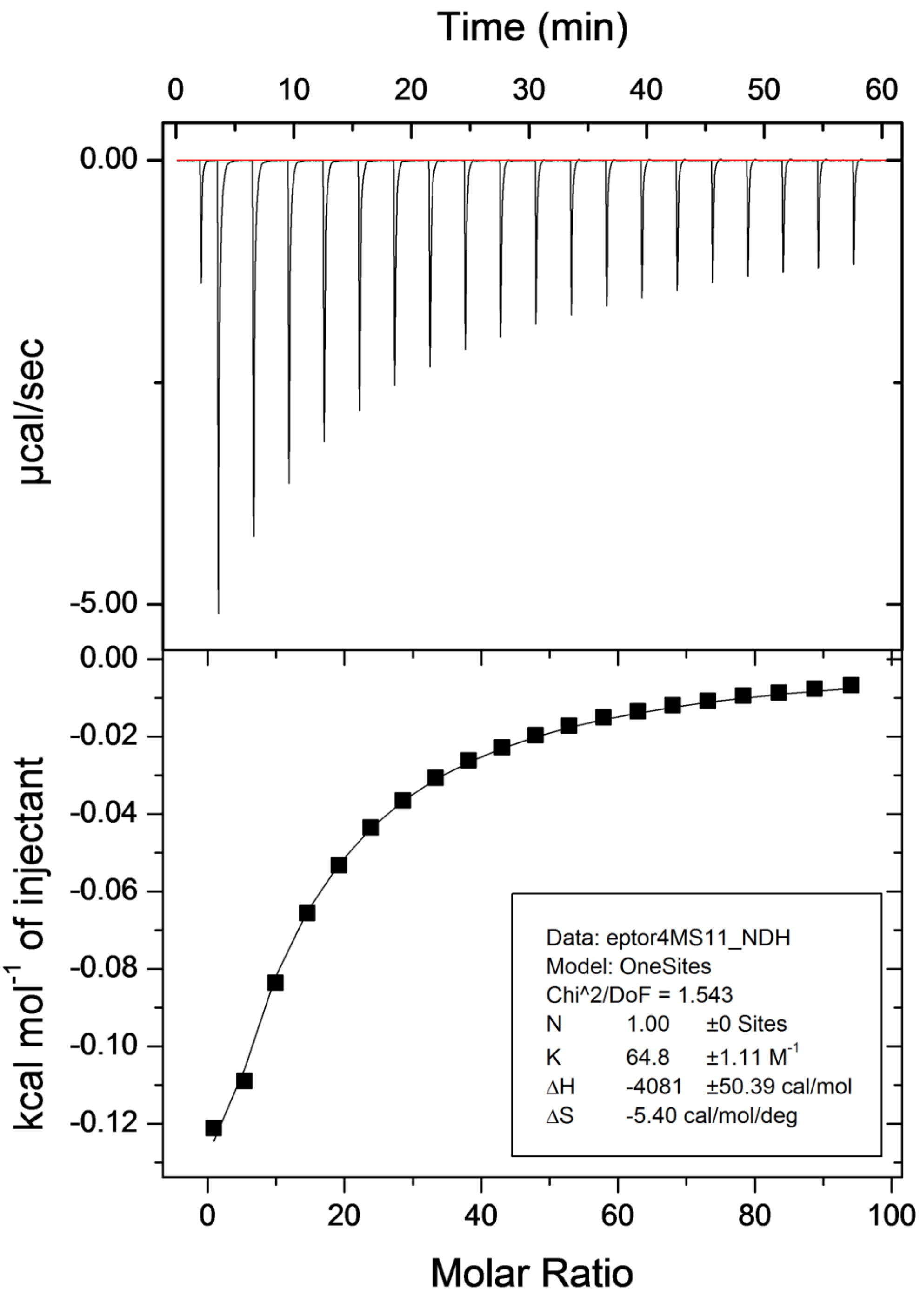

Figure S69. Output from the ITC experiment for the titration of receptor **4** (0.5 mM) with D-glucose **3** (225 mM) in H_2O at 298 K. K_a = 65 M^{-1}.

Methyl β-ᴅ-glucoside

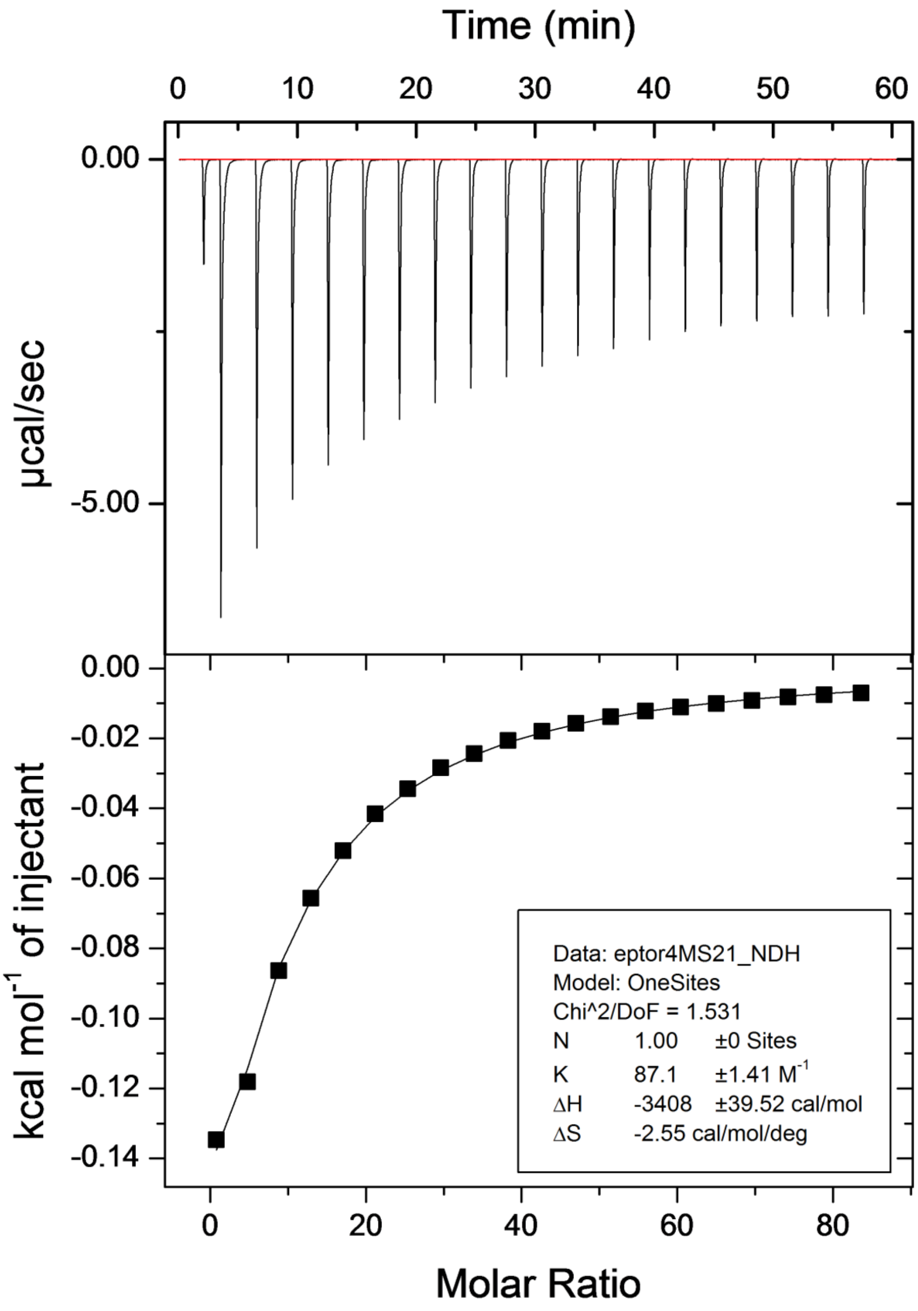

Figure S70. Output from the ITC experiment for the titration of receptor **4** (0.5 mM) with methyl β-D-glucoside (200 mM) in H_2O at 298 K. K_a = 87 M^{-1}.

D-Glucosamine 13 (NaCl 154 mM)

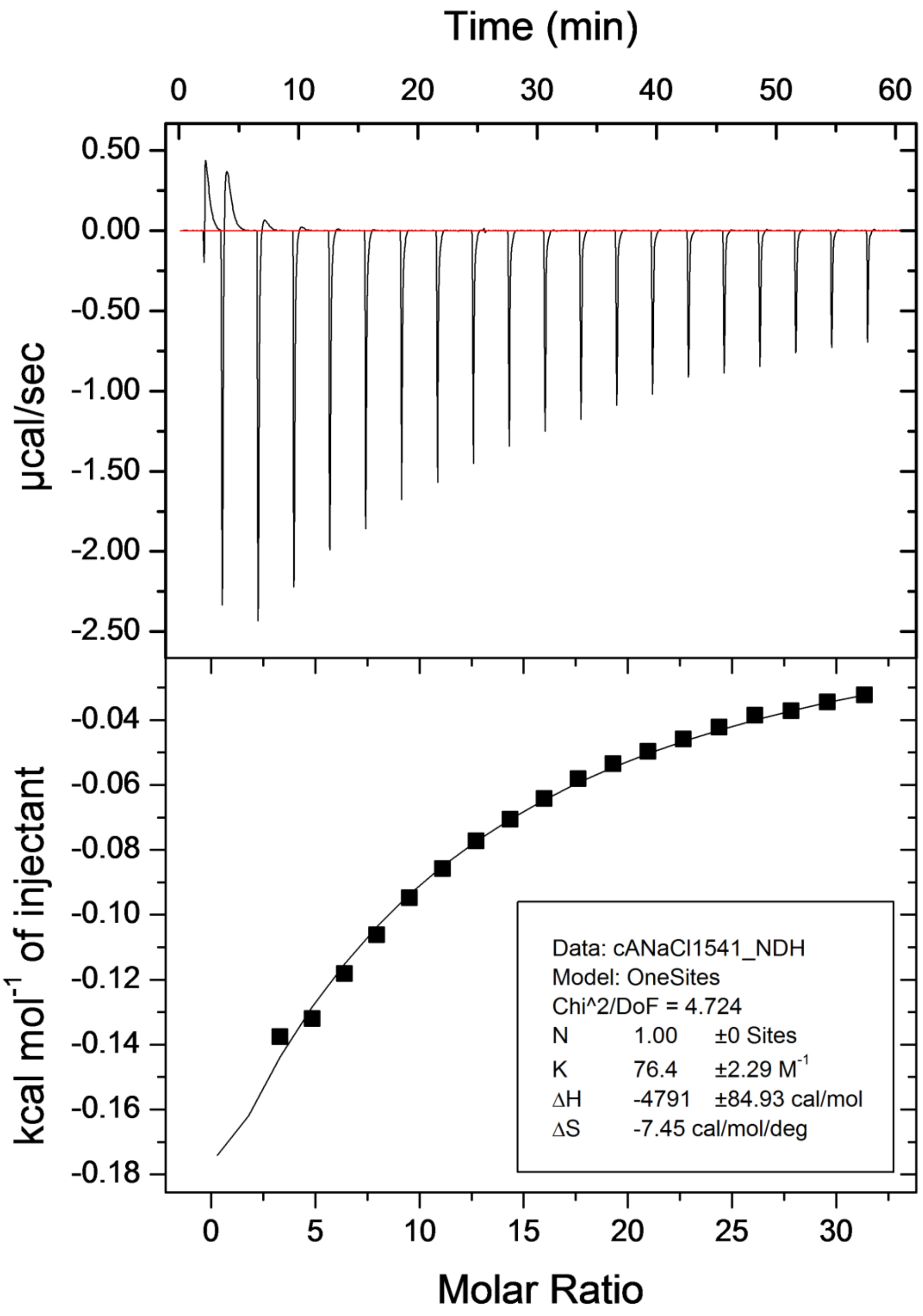

Figure S71. Output from the ITC experiment for the titration of receptor **4** (0.5 mM) with D-glucosamine **13** (75 mM) in H_2O with NaCl (154 mM, pH = 7.1) at 298 K. K_a = 76 M^{-1}.

Fluorescence Titrations

D-Glucose 3

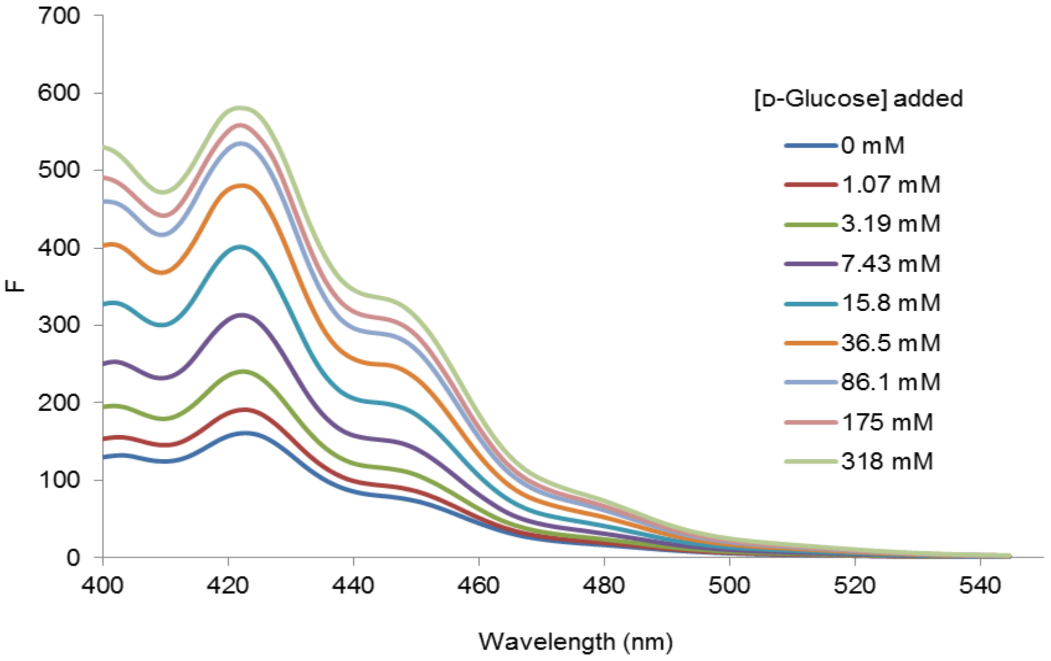

Figure S72. Fluorescence titration of receptor **4** (15 μM) with D-glucose **3** in phosphate buffer solution (pH = 7.1, 0.1 M) at 298 K. Excitation wavelength: 395 nm.

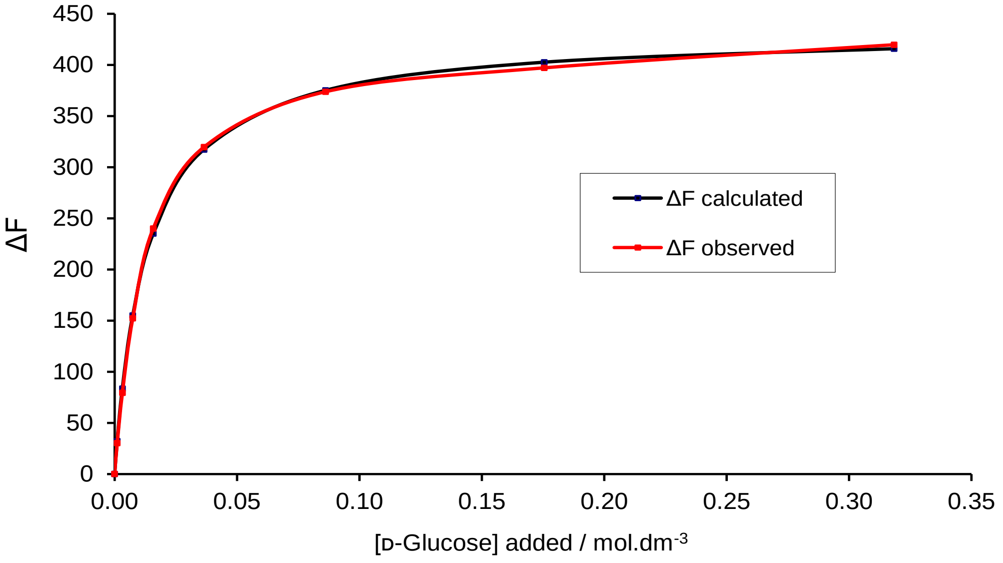

Figure S73. Experimental and calculated values for the fluorescence binding study of receptor **4** (15 μM) with D-glucose **3** in phosphate buffer solution (pH = 7.1, 0.1 M) at 298 K. Excitation: 395 nm. Emission observed at: 423 nm. K_a = 75 M^{-1} ± 12%. F/F^o = 3.70.

D-Glucosamine 13

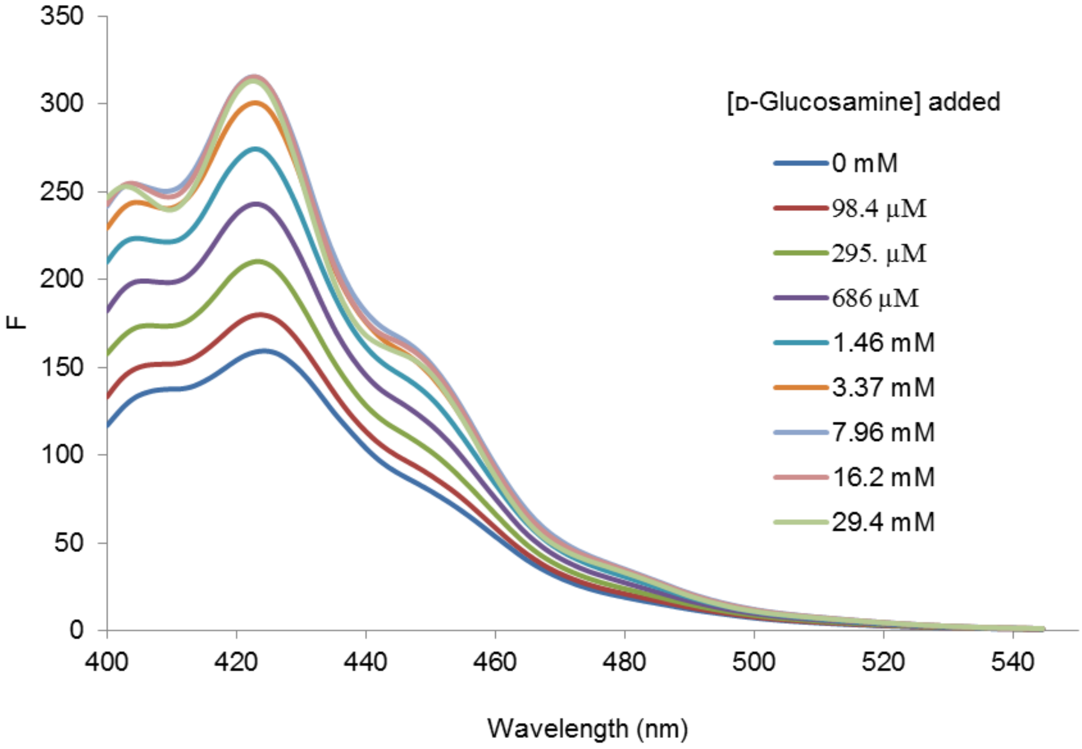

Figure S74. Fluorescence titration of receptor **4** (15 μM) with D-glucosamine **13** in H_2O (pH = 7.0) at 298 K. Excitation wavelength: 395 nm.

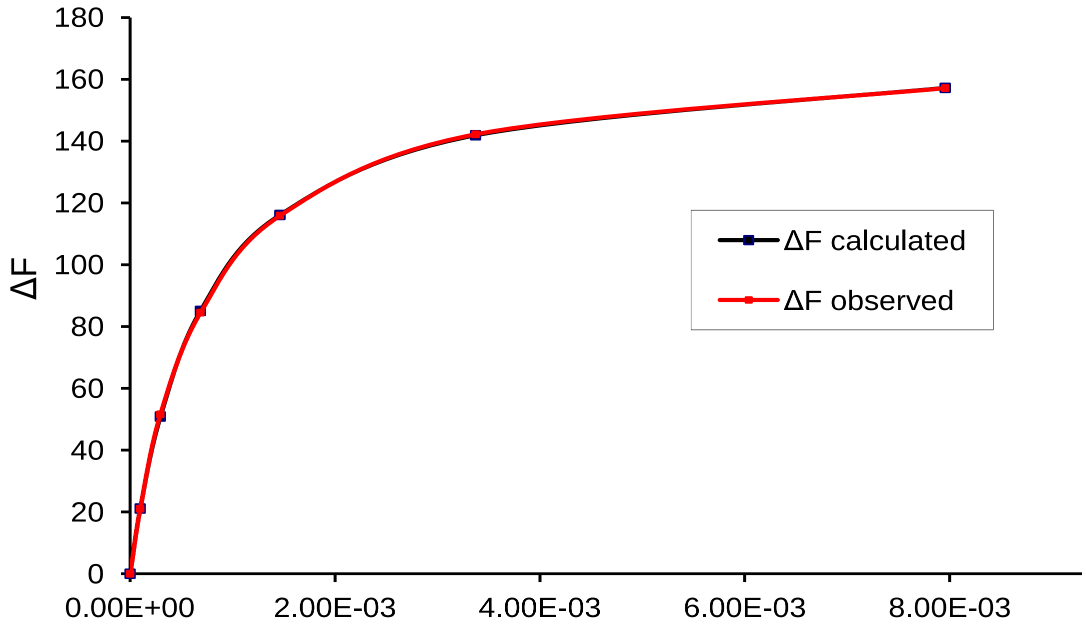

Figure S75. Experimental and calculated values for the fluorescence binding study of receptor **4** (15 μM) with D-glucosamine **13** in water (pH = 7.0) at 298 K. Excitation: 395 nm. Emission observed at: 423 nm. K_a = 1500 M^{-1} ± 1.16 %. F/F° = 2.07.

Receptor 5 Carbohydrate Binding Studies

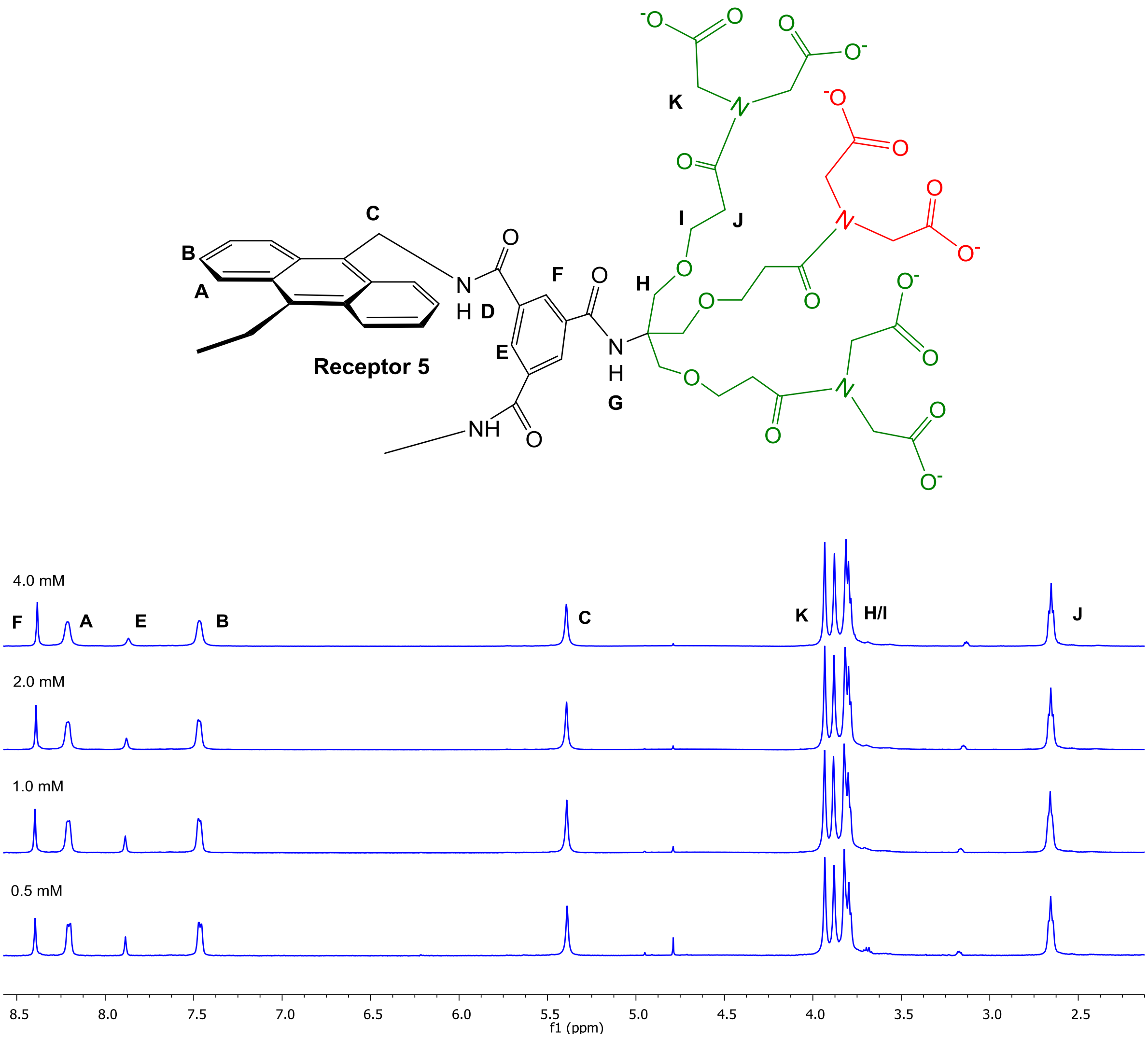

Figure S76. Partial ^{1}H NMR spectra of receptor **5** at concentrations from 0.5 mM to 4 mM in D_2O at 298 K, with assignments.

NMR spectra and binding analyses

D-Glucose 3

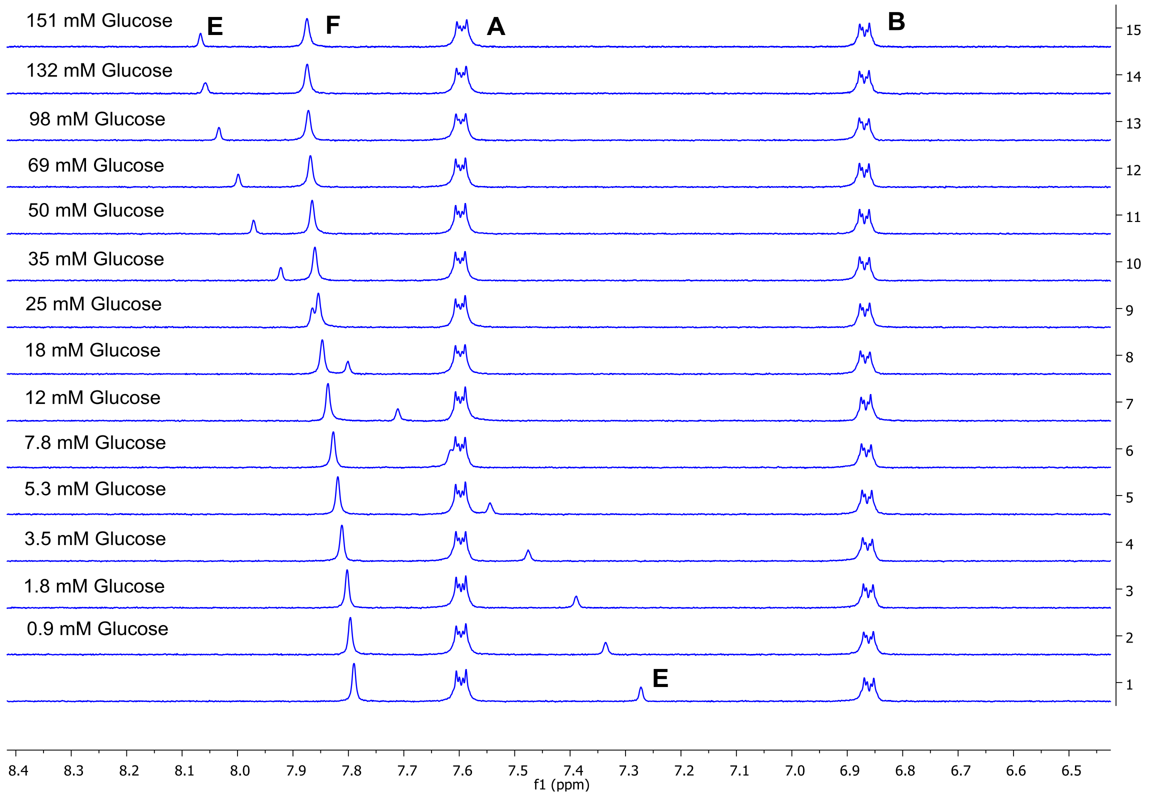

Figure S77. Partial ^{1}H NMR spectra from the titration of receptor **5** (0.25 mM) with D-glucose **3** in D_2O at 298 K.

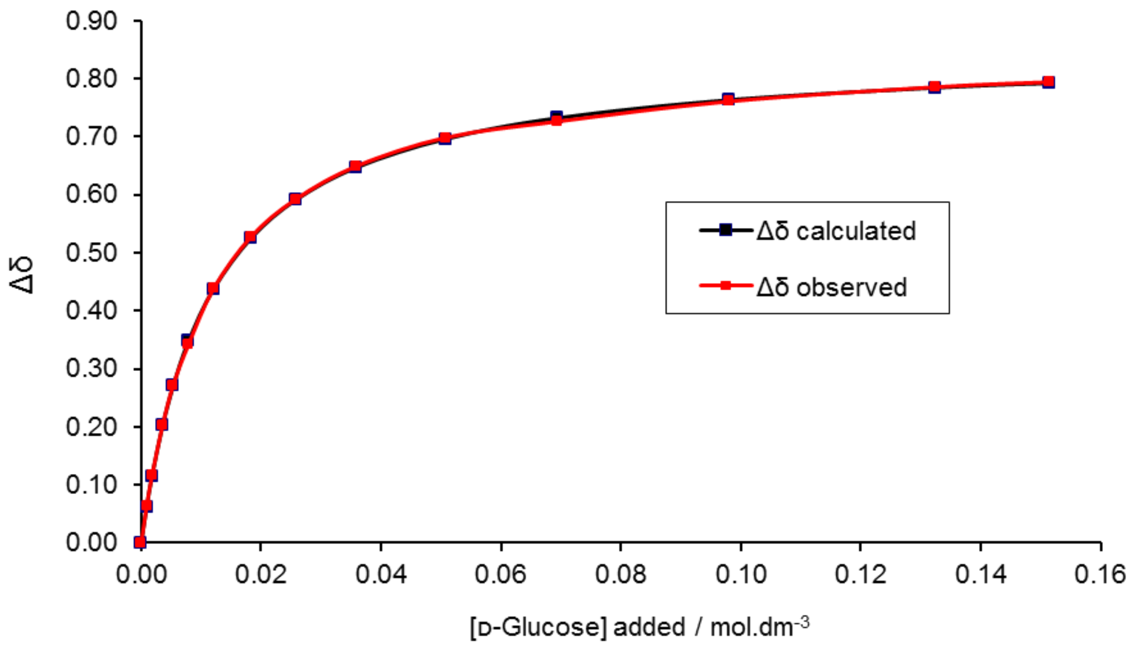

Figure S78. Experimental and calculated values for the NMR binding study of receptor **5** (0.25 mM) with D-glucose **3** in D_2O. Proton E: K_a = 89 M^{-1} ± 1%, Δδ = 0.85.

Methyl β-ᴅ-glucoside

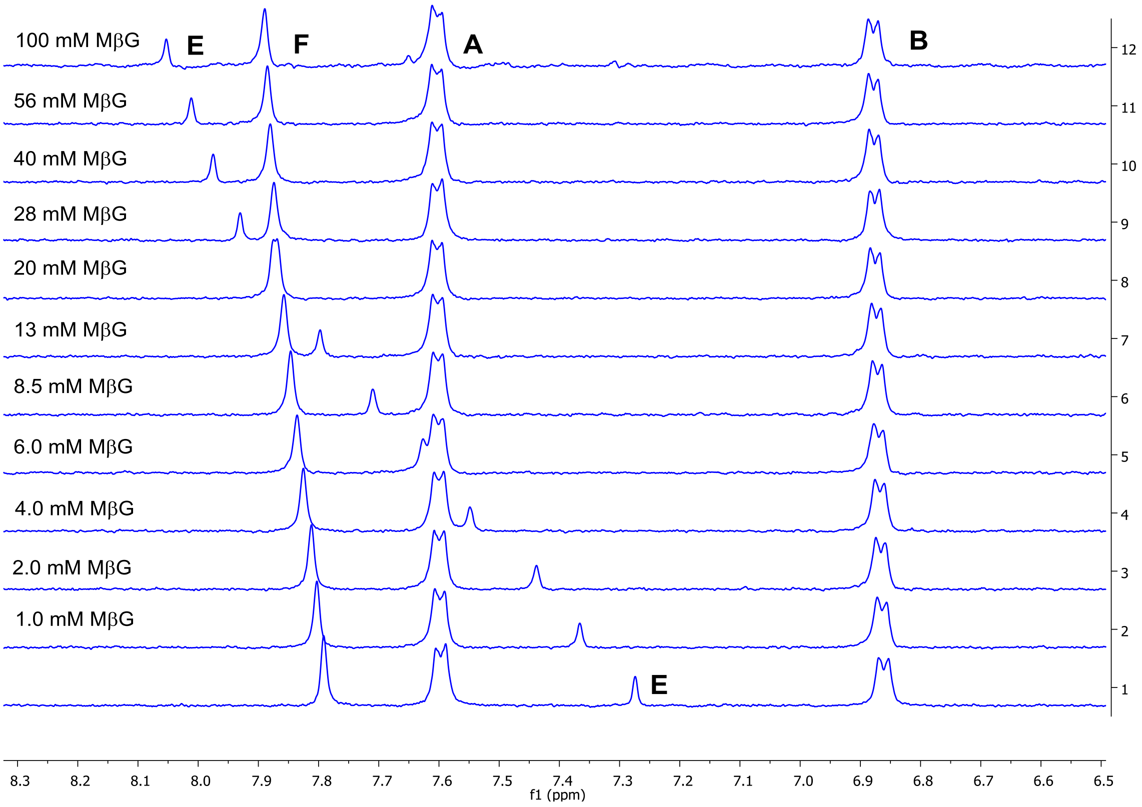

Figure S79. Partial ^{1}H NMR spectra from the titration of receptor **5** (0.25 mM) with methyl β-D-glucoside in D_2O at 298 K.

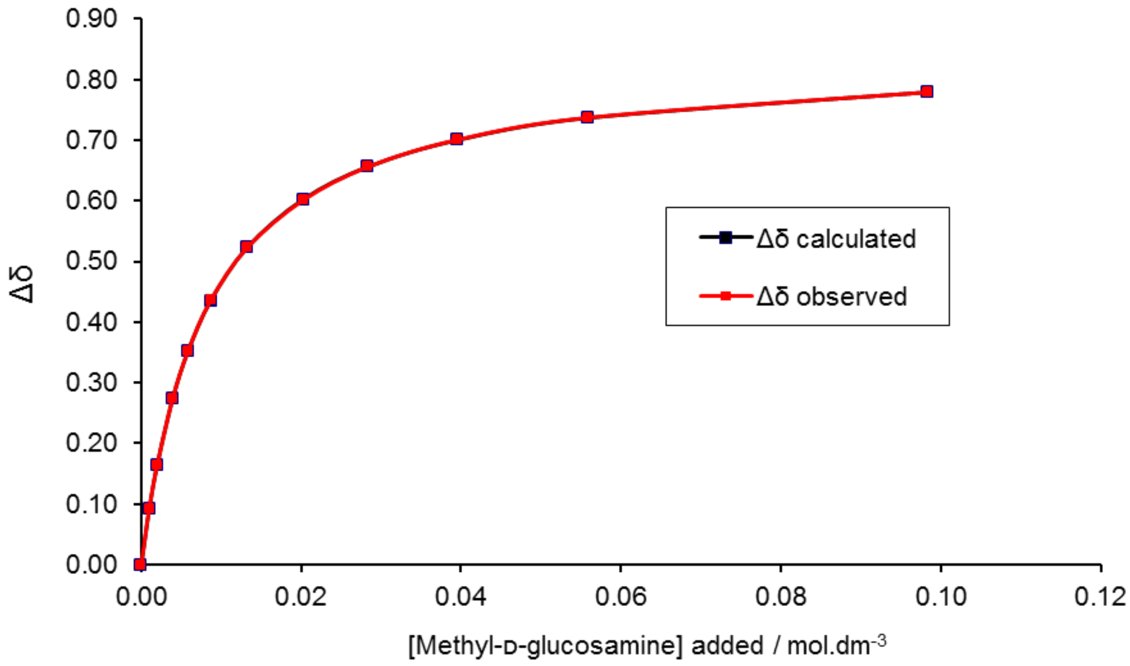

Figure S80. Experimental and calculated values for the NMR binding study of receptor **5** (0.25 mM) with methyl β-D-glucoside in D_2O. Proton E: K_a = 124 M^{-1}± 1%, Δδ = 0.84.

N-Acetyl-ᴅ-glucosamine 15

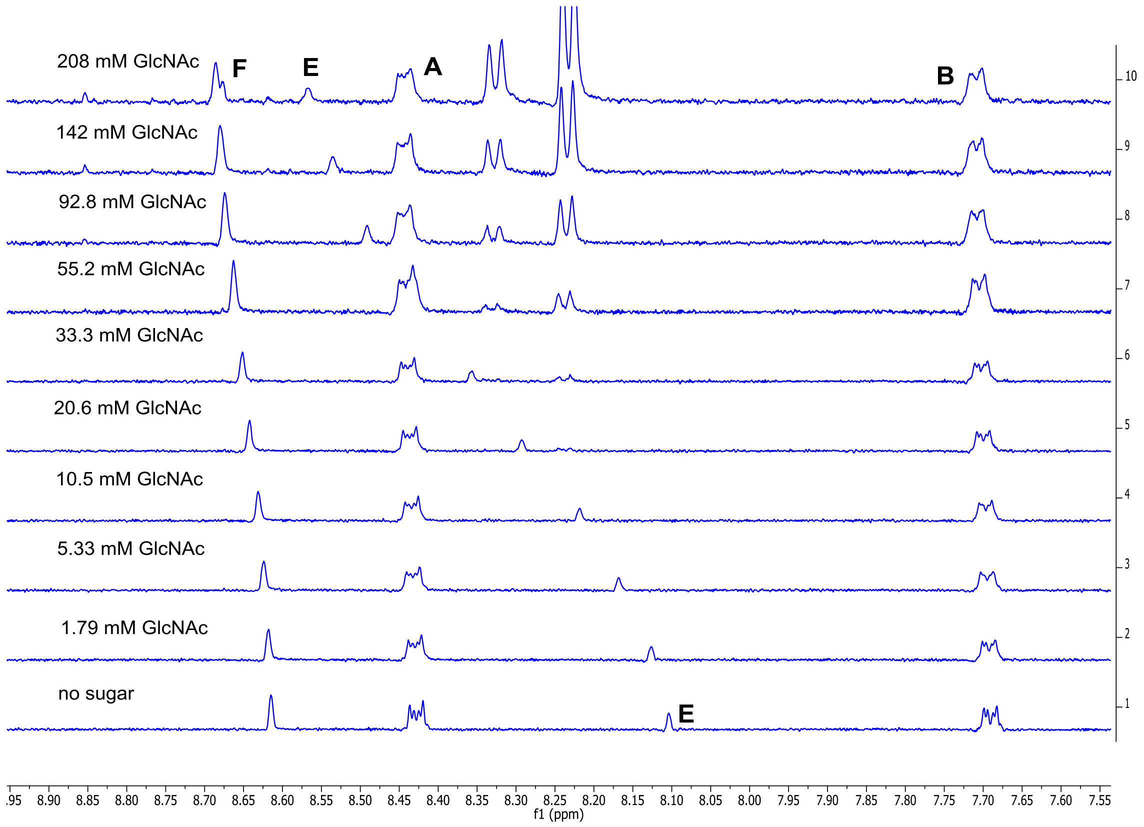

FigureS81. Partial ^{1}H NMR spectra from the titration of receptor **5** (0.20 mM) with GlcNAc **15** in D_2O at 298 K.

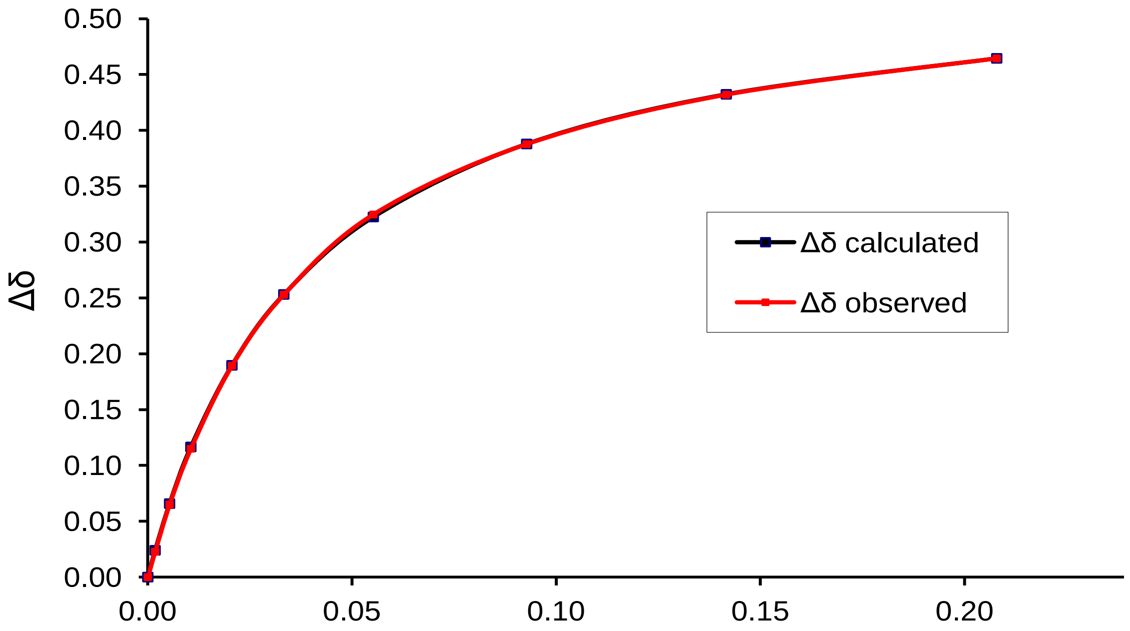

Figure 82. Experimental and calculated values for the NMR binding study of receptor **5** (0.20 mM) with GlcNAc **15** in D_2O at 298 K. Proton E: K_a = 25 M^{-1} ± 2%, Δδ = 0.55.

D-Galactose

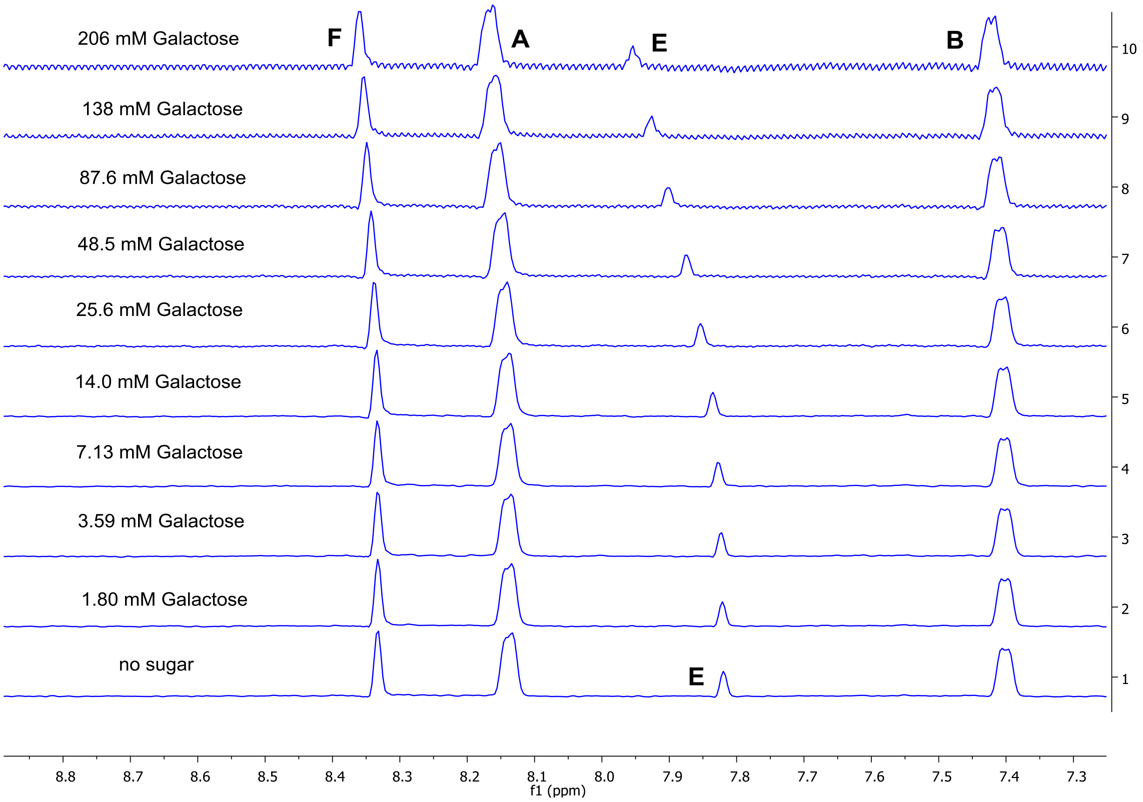

Figure S83. Partial ^{1}H NMR spectra from the titration of receptor **5** (0.10 mM) with D-galactose in D_2O at 298 K.

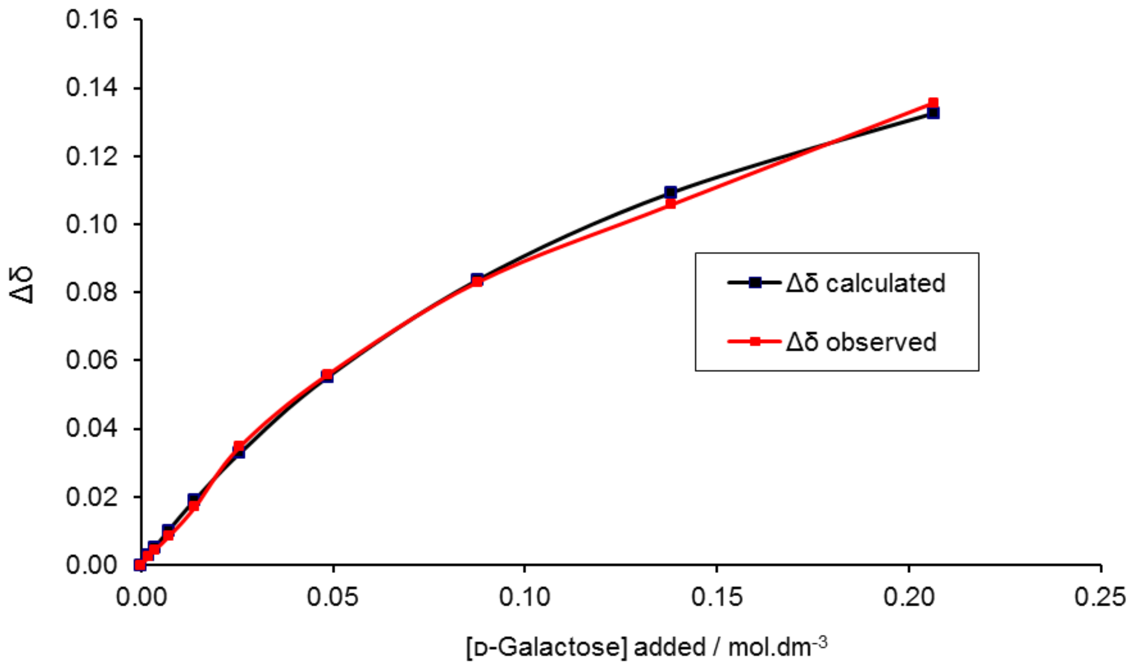

Figure S84. Experimental and calculated values for the NMR binding study of receptor **5** (0.10 mM) with D-galactose in D_2O at 298 K. Proton E: K_a = 6.4 M^{-1} ± 8%, Δδ = 0.23.

D-Mannose

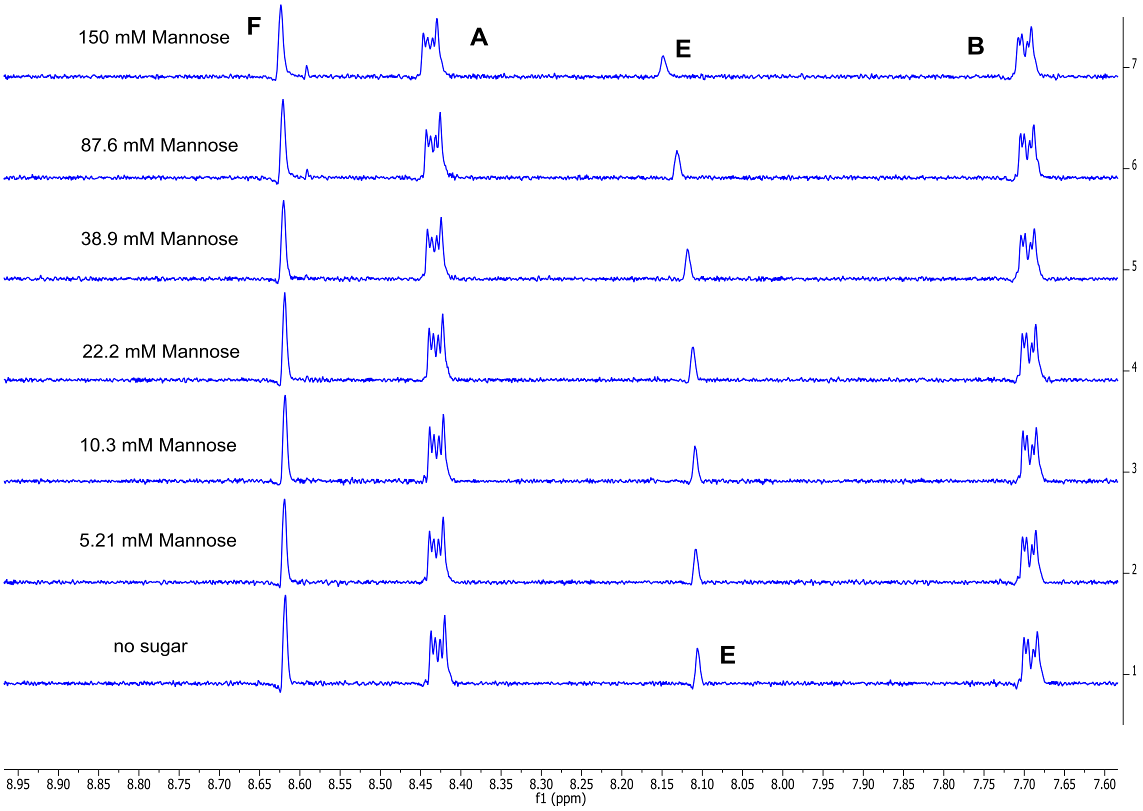

Figure S85. Partial ^1H NMR spectra from the titration of receptor **5** (0.10 mM) with D-mannose in D_2O at 298 K.

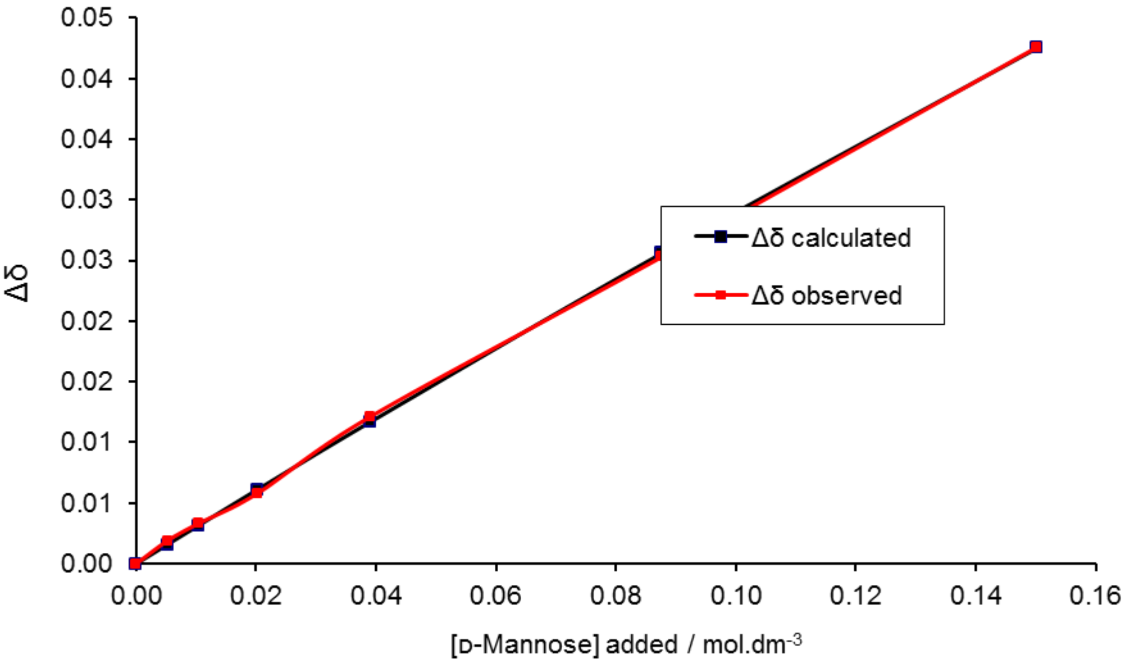

Figure S86. Experimental and calculated values for the NMR binding study of receptor **5** (0.10 mM) with D-mannose in D_2O at 298 K. Proton E: K_a too small to be evaluated with reasonable accuracy.

D-Maltose

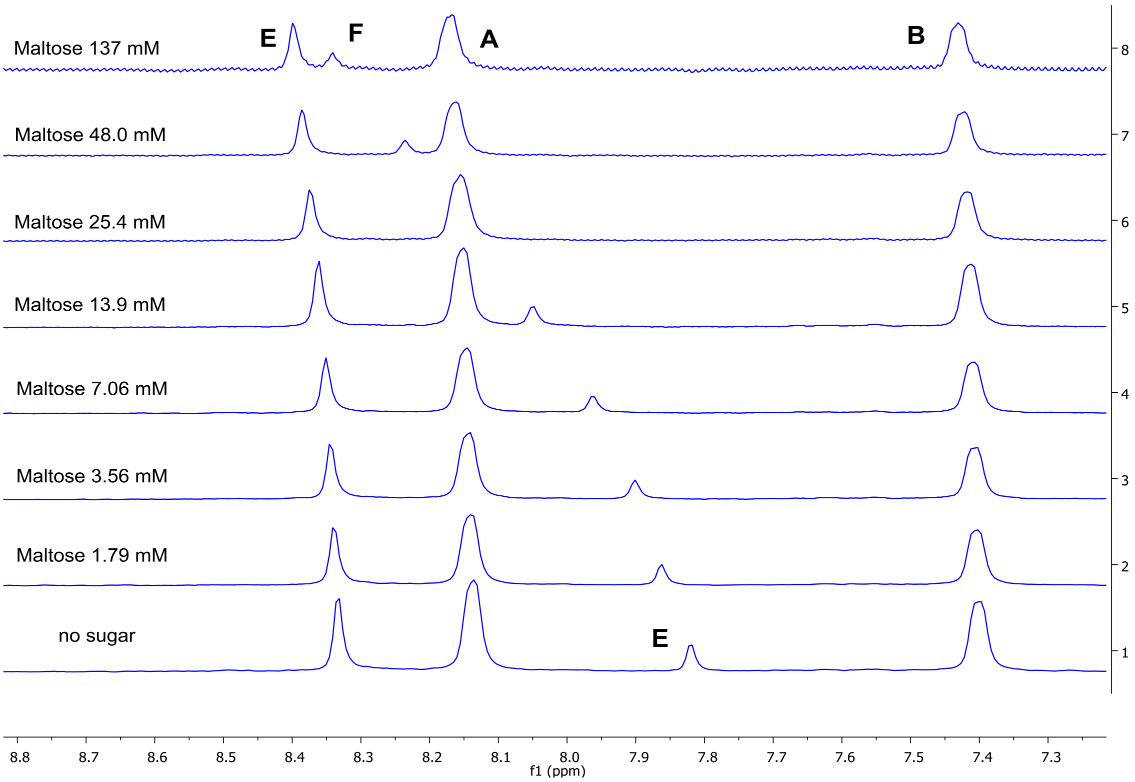

Figure S87. Partial ^{1}H NMR spectra from the titration of receptor **5** (0.10 mM) with D-maltose in D_2O at 298 K.

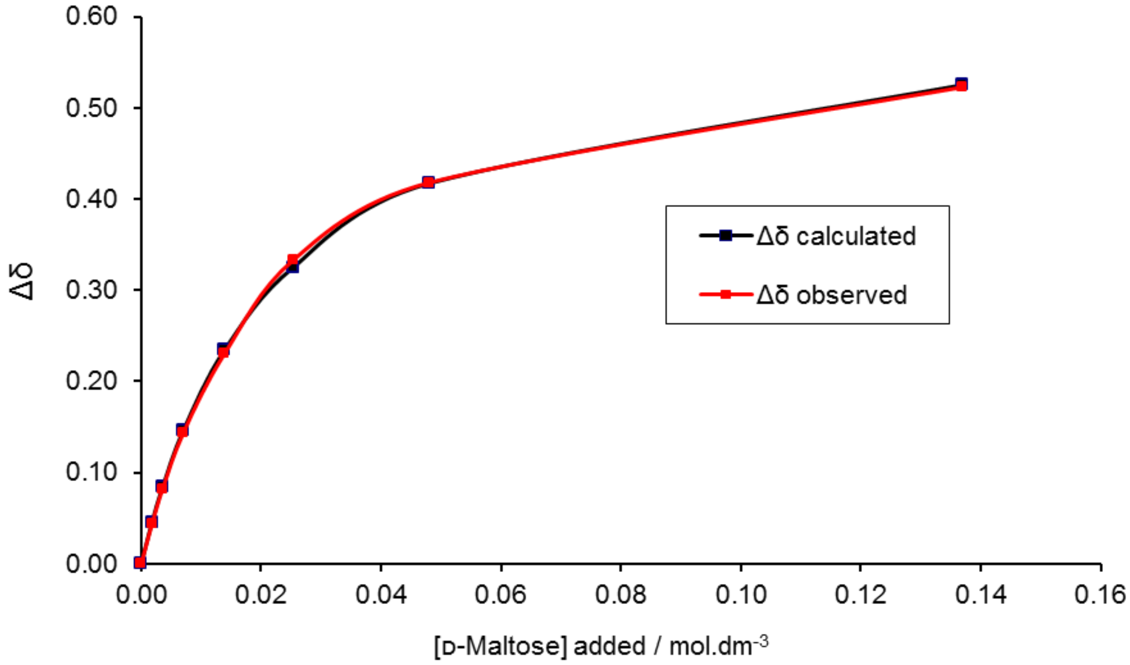

Figure S88. Experimental and calculated values for the NMR binding study of receptor **5** (0.10 mM) with D-maltose in D_2O at 298 K. Proton E: K_a = 45 M^{-1} ± 3%, Δδ = 0.61.

D-Cellobiose

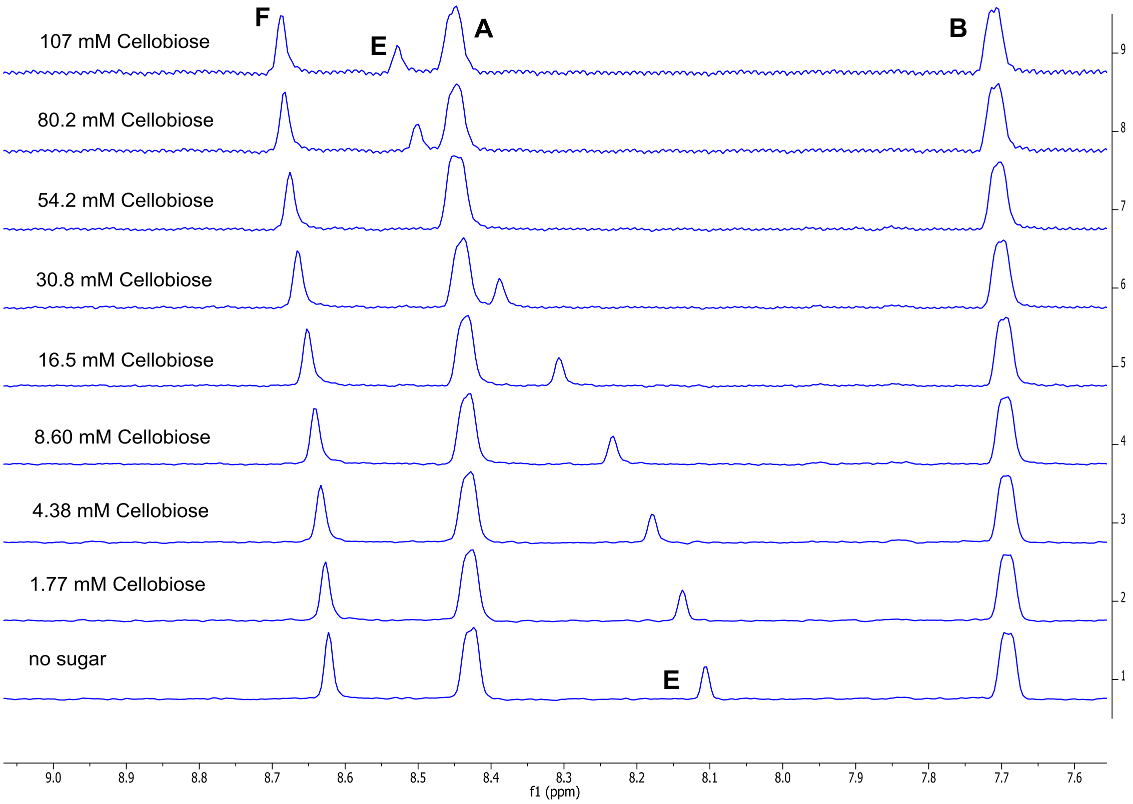

Figure S89. Partial ^1H NMR spectra from the titration of receptor **5** (0.05 mM) with D-cellobiose in D_2O at 298 K.

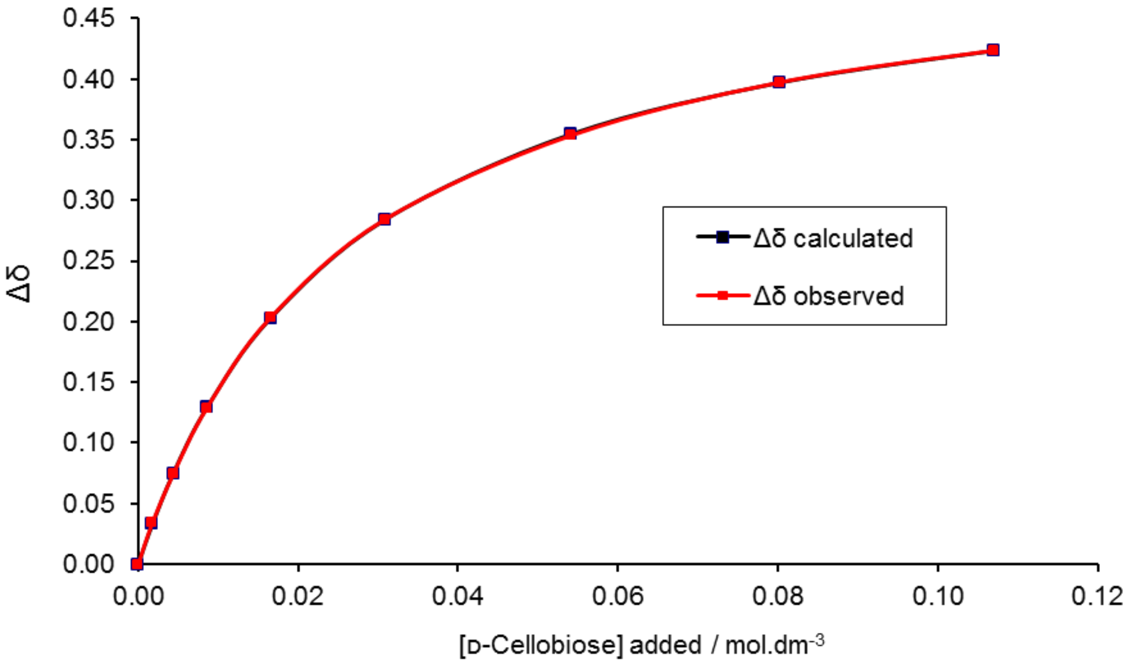

Figure S90. Experimental and calculated values for the NMR binding study of receptor **5** (0.05 mM) with D-cellobiose in D_2O at 298 K. Proton E: K_a = 38 M^{-1} ± 1%, Δδ = 0.53.

D-Lactose

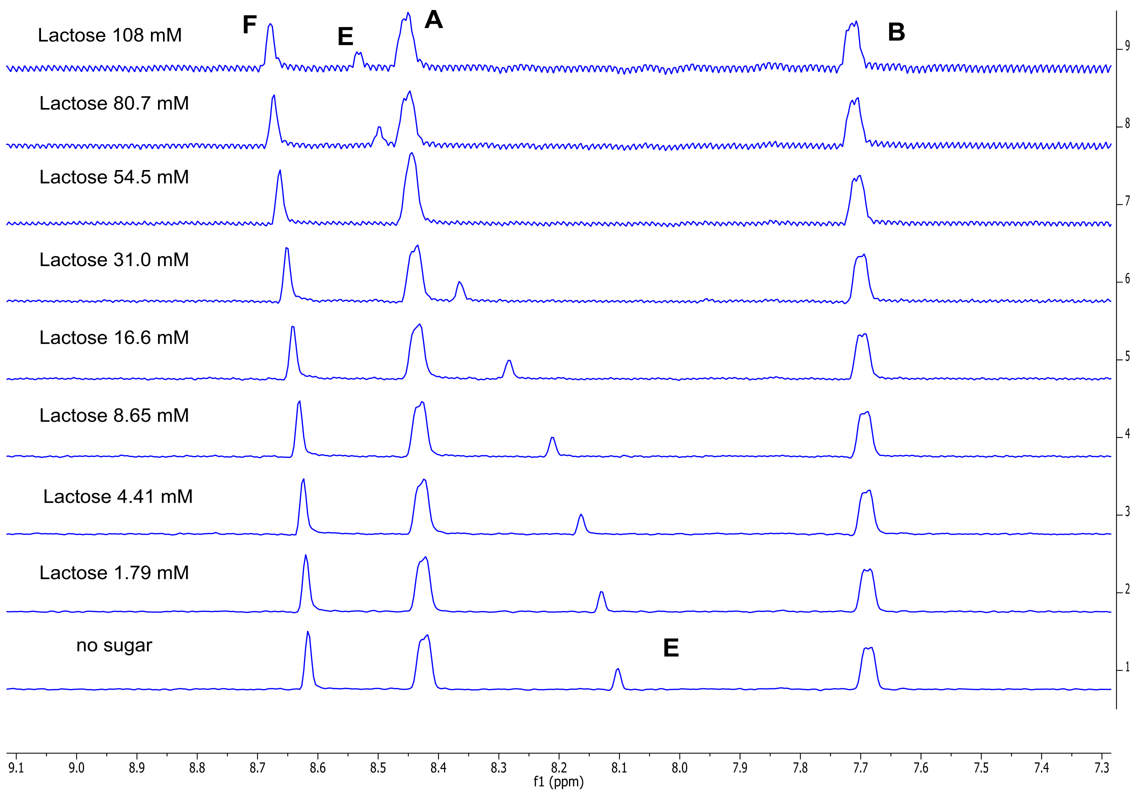

Figure S91. Partial ^{1}H NMR spectra from the titration of receptor **5** (0.05 mM) with D-lactose in D_2O at 298 K.

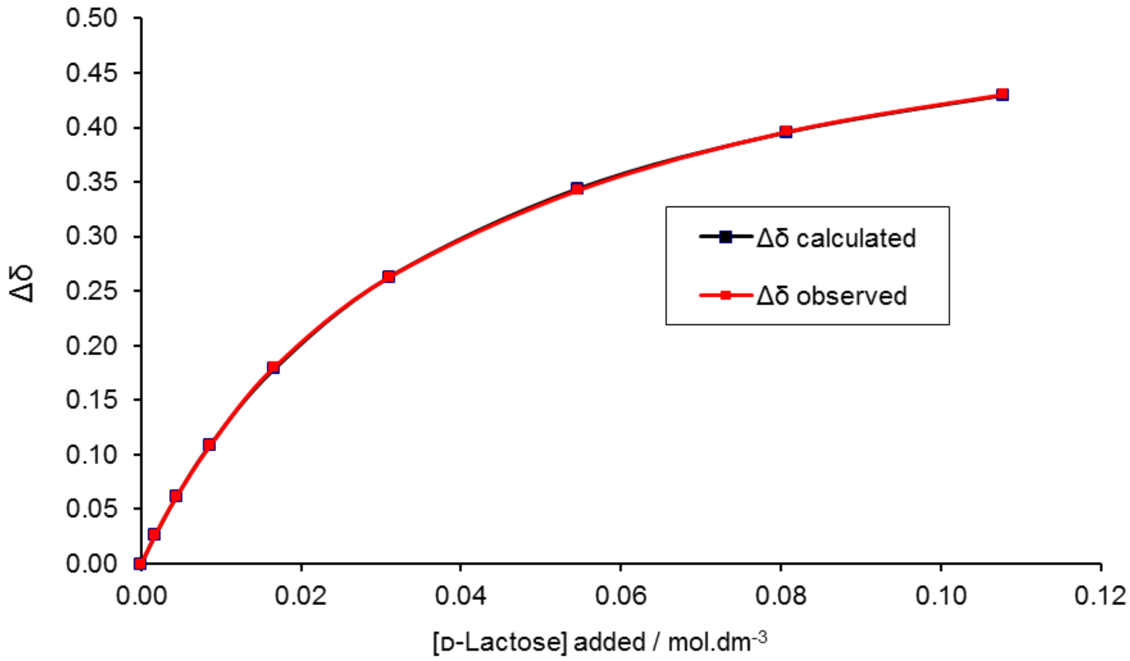

Figure S92. Experimental and calculated values for the NMR binding study of receptor **5** (0.05 mM) with D-lactose in D_2O at 298 K. Proton E: K_a = 27 M^{-1} ± 1 %, Δδ = 0.61.

ᴅ-Glucosamine 13

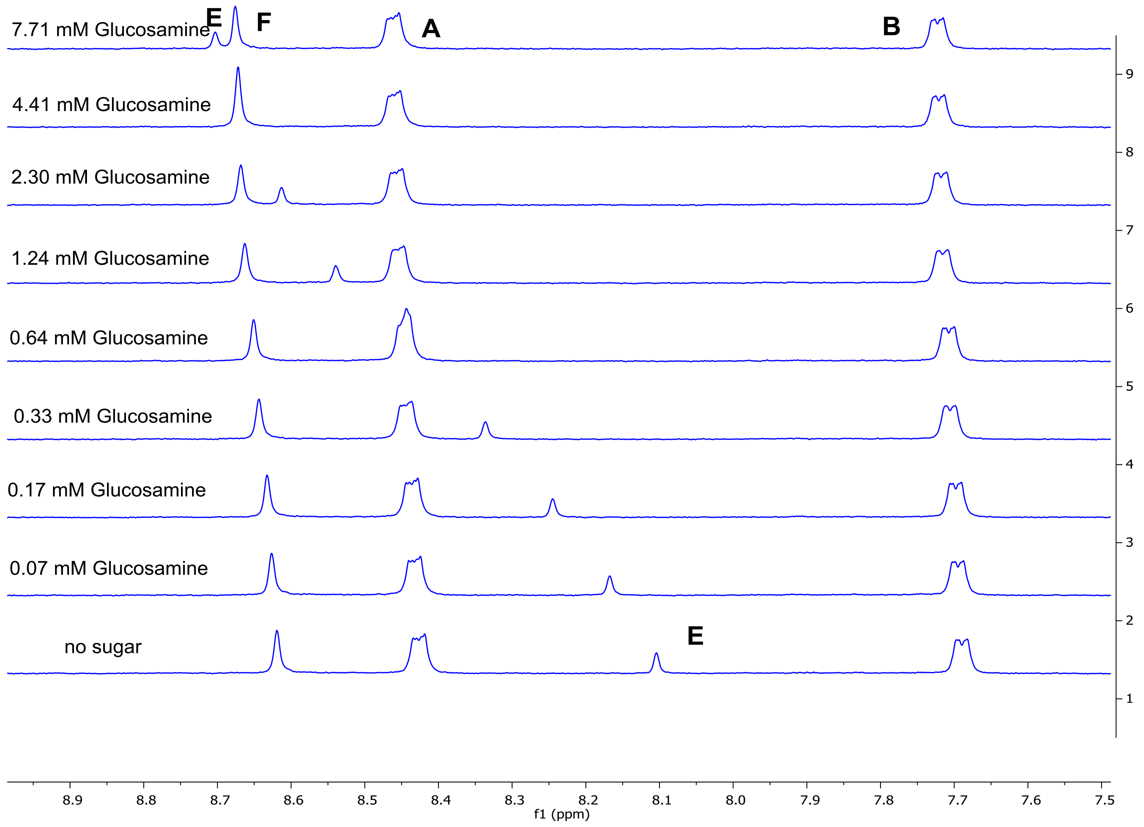

Figure S93. Partial ^{1}H NMR spectra from the titration of receptor **5** (0.11 mM) with D-glucosamine **13** in D_2O (pH = 7) at 298 K.

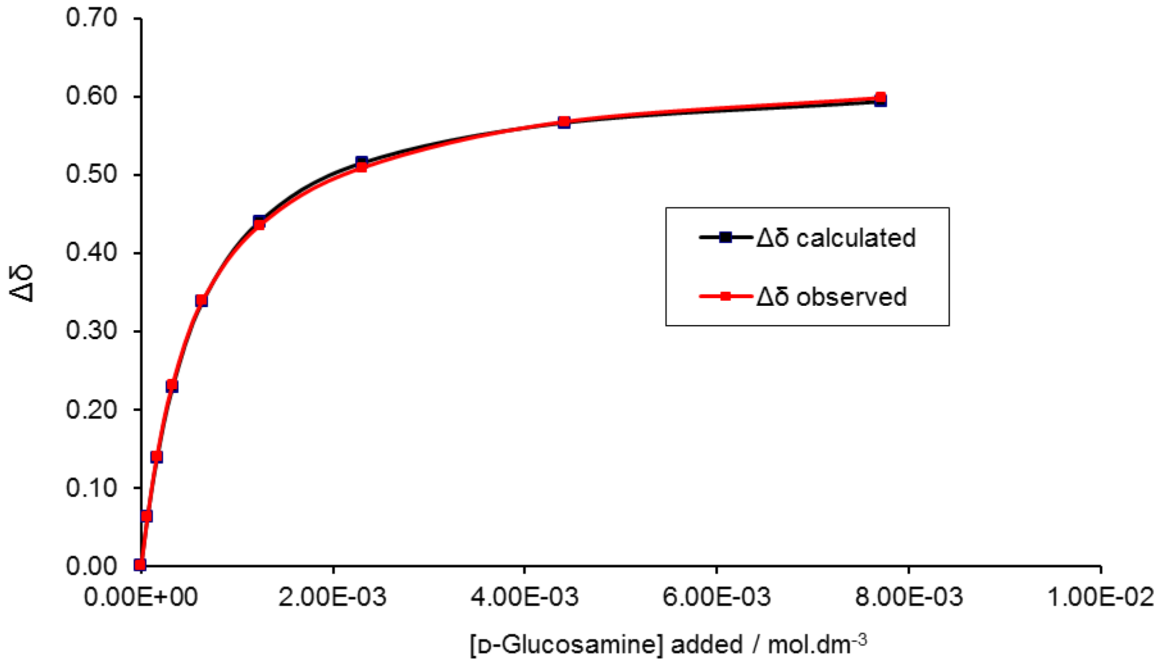

Figure S94. Experimental and calculated values for the NMR binding study of receptor **5** (0.11 mM) with D-glucosamine **13** in D_2O (pH = 7) at 298 K. Proton E: K_a = 2000 M^{-1} ± 6%, Δδ = 0.63.

D-Glucosamine (NaCl 20 mM)

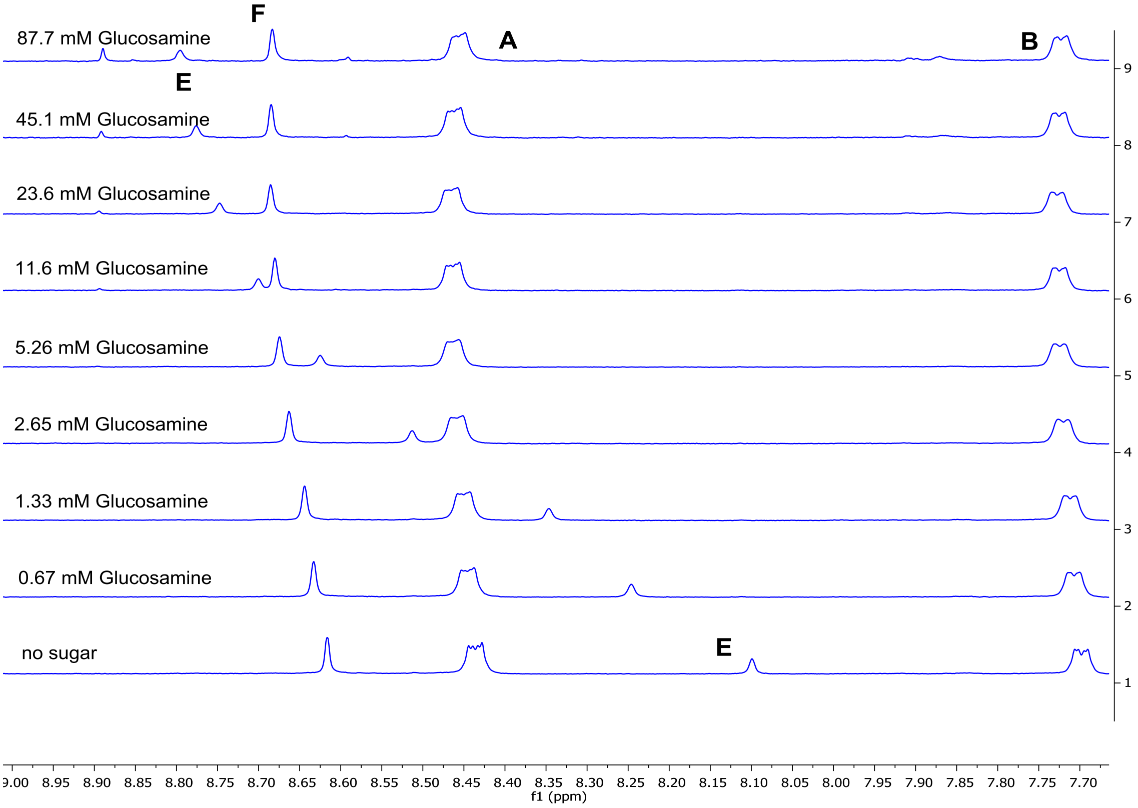

Figure S95. Partial ^{1}H NMR spectra from the titration of receptor **5** (0.13 mM) with D-glucosamine **13** in D_2O (pH = 7, 20 mM, NaCl) at 298 K.

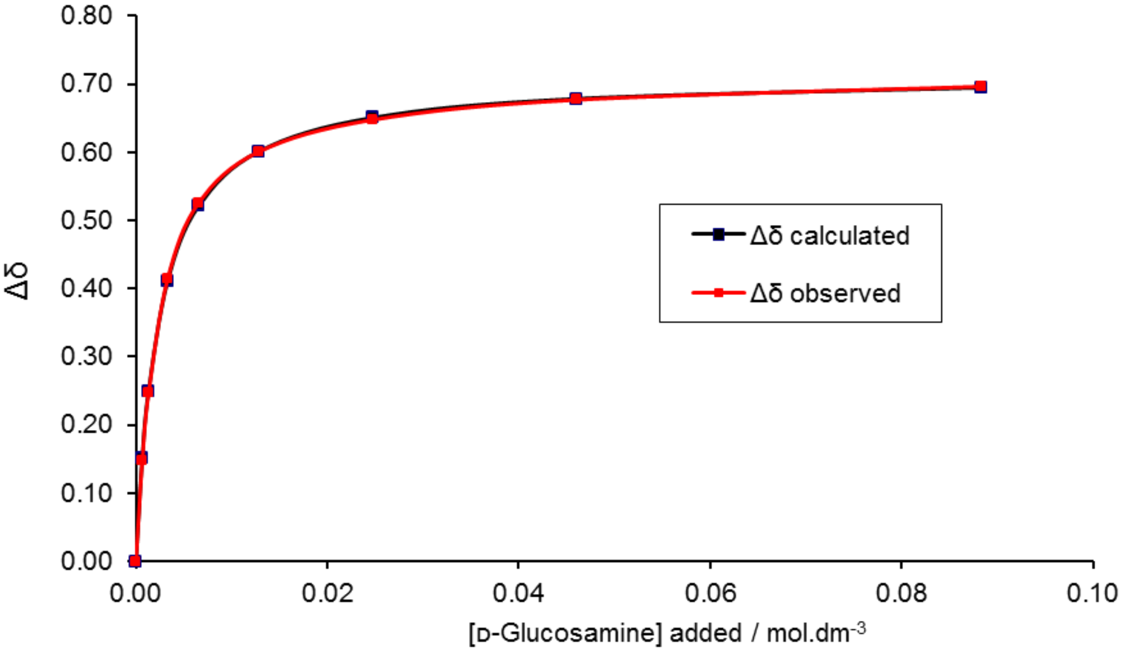

Figure S96. Experimental and calculated values for the NMR binding study of receptor **5** (0.13 mM) with D-glucosamine **13** in D_2O (pH = 7, 20 mM, NaCl) at 298 K. Proton E: K_a = 420 M^{-1} ± 4%, Δδ = 0.71.

ᴅ-Glucosamine (NaCl 154 mM)

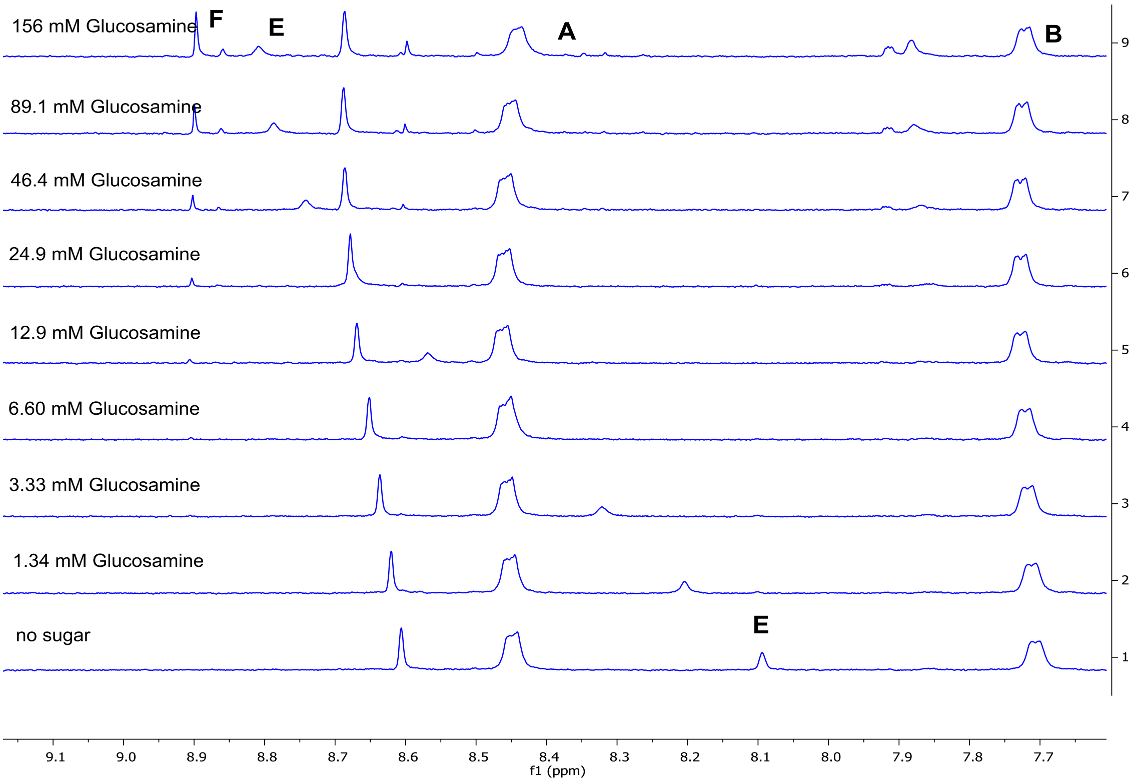

Figure S97. Partial ^{1}H NMR spectra from the titration of receptor **5** (0.13 mM) with D-glucosamine **13** in D_2O (pH = 7, 154 mM, NaCl) at 298 K.

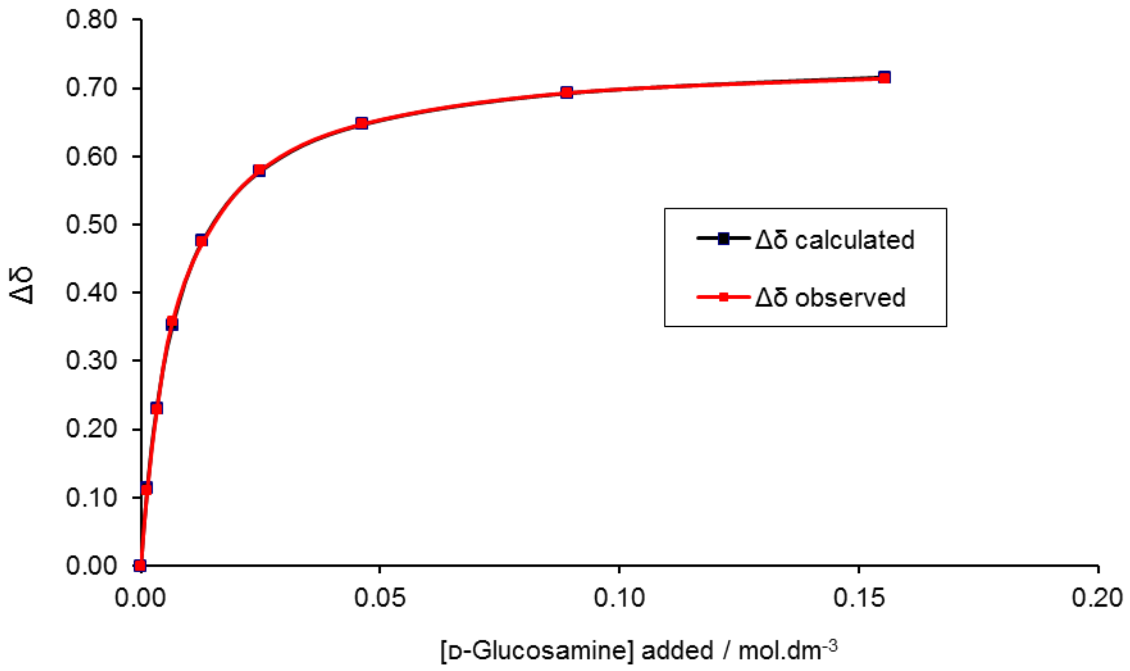

Figure S98. Experimental and calculated values for the NMR binding study of receptor **5** (0.13 mM) with D-glucosamine **13** in D_2O (pH = 7, 154 mM, NaCl) at 298 K. Proton E: K_a = 135 M^{-1} ± 3%, Δδ = 0.75.

D-Galactosamine (NaCl 154 mM)

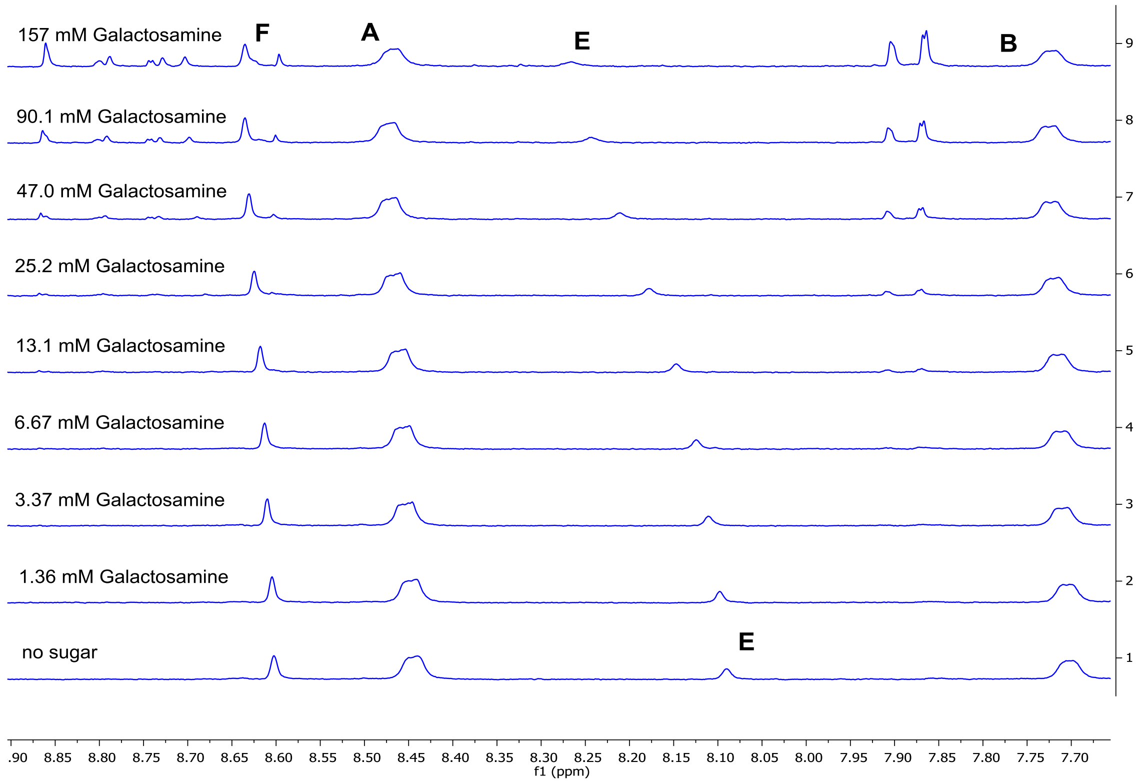

Figure S99. Partial ^{1}H NMR spectra from the titration of receptor **5** (0.13 mM) with D-galactosamine in D_2O (pH = 7, 154 mM, NaCl) at 298 K.

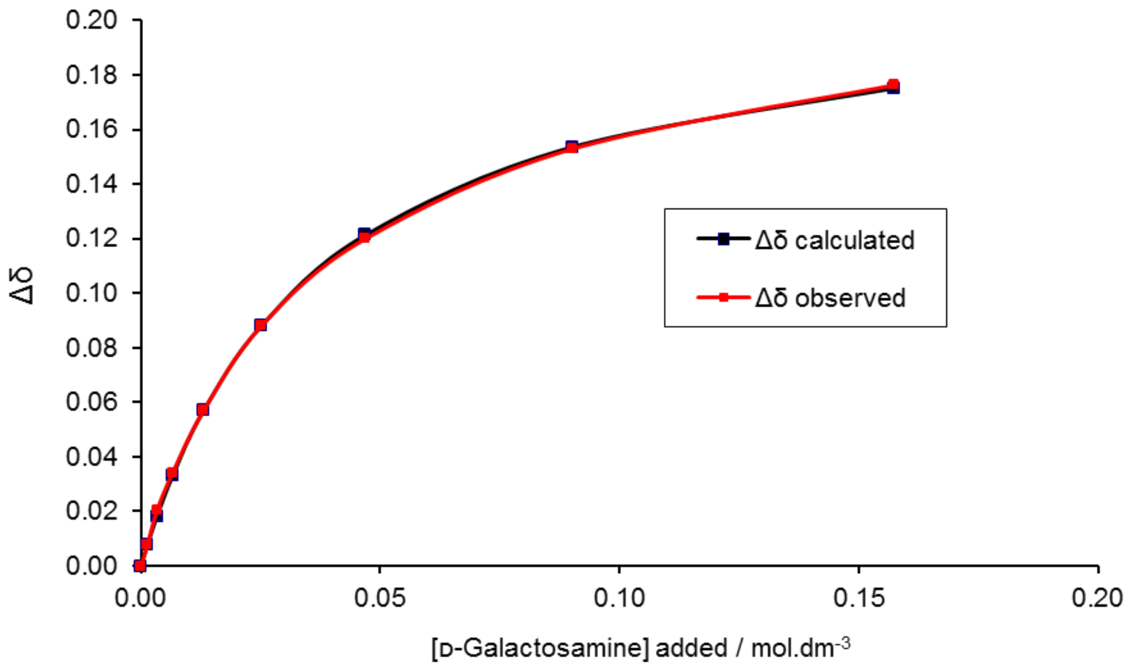

Figure S100. Experimental and calculated values for the NMR binding study of receptor **5** (0.13 mM) with D-galactosamine in D_2O (pH = 7, 154 mM, NaCl) at 298 K. Proton E: K_a = 27 M^{-1} ± 5%, Δδ = 0.22

Fluorescence Titrations

D-Glucose 3

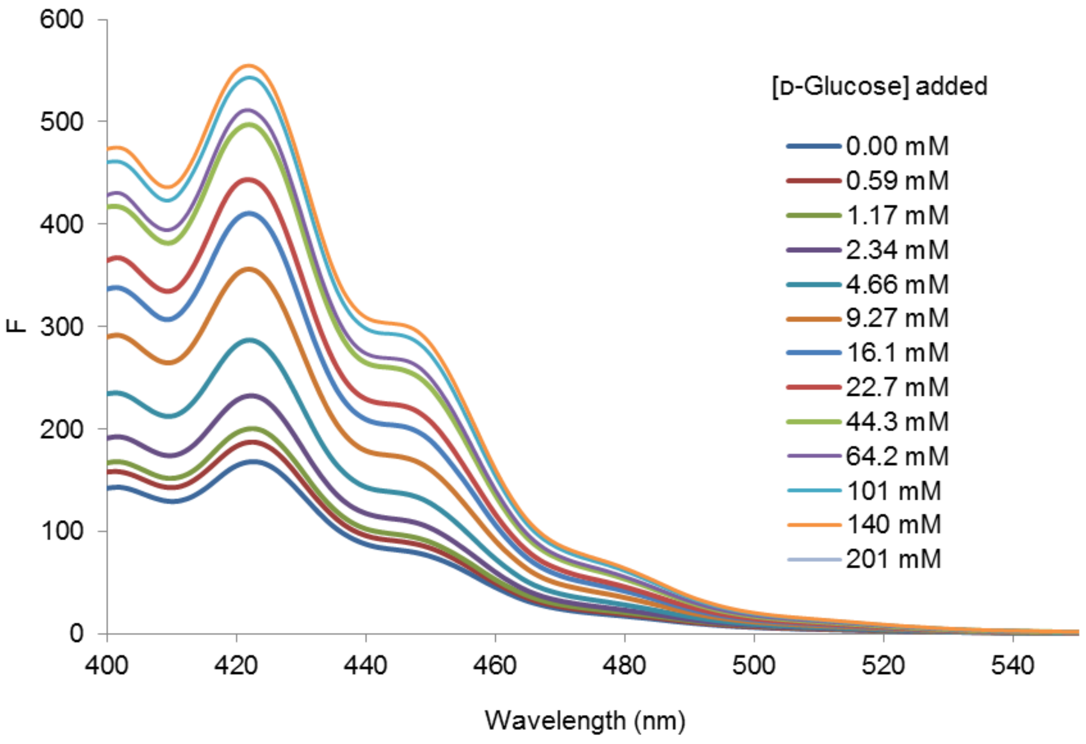

Figure S101. Fluorescence titration of receptor **5** (18.8 μM) with D-glucose **3** in phosphate buffer solution (pH = 7.1, 0.1 M) at 298 K. Excitation wavelength: 395 nm.

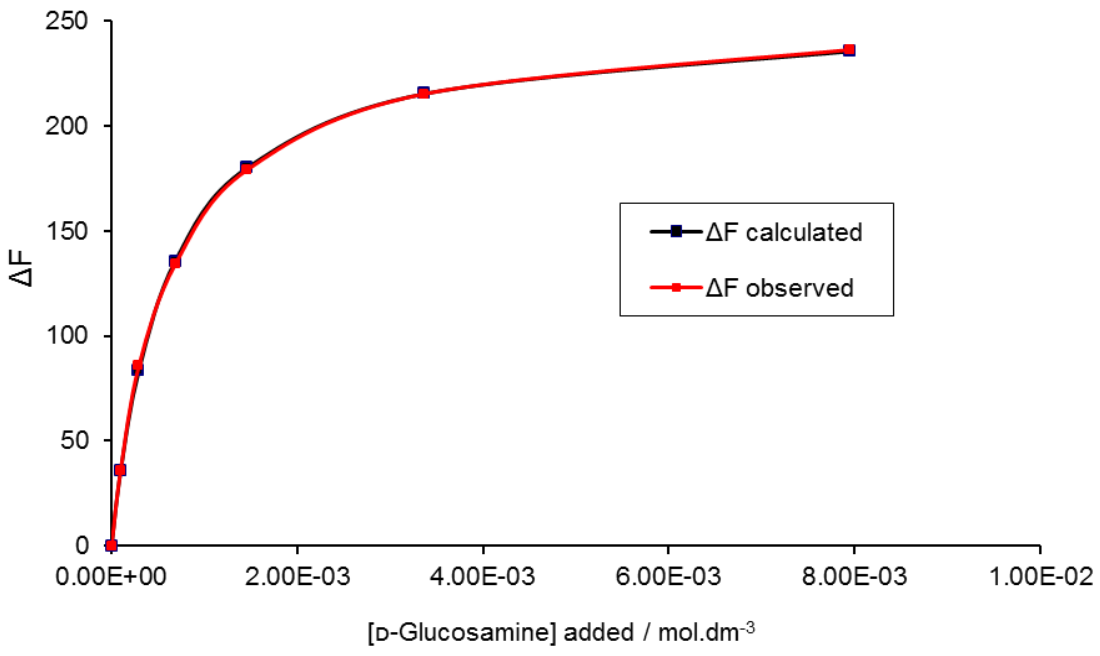

Figure 102. Experimental and calculated values for the fluorescence binding study of receptor **5** (18.8 μM) with D-glucose **3** in phosphate buffer solution (pH = 7.1, 0.1 M) at 298 K. Excitation: 395 nm. Emission observed at: 423 nm. K_a = 91 M^{-1} ± 7%. F/F^{o} = 3.40.

ᴅ-Glucosamine 13

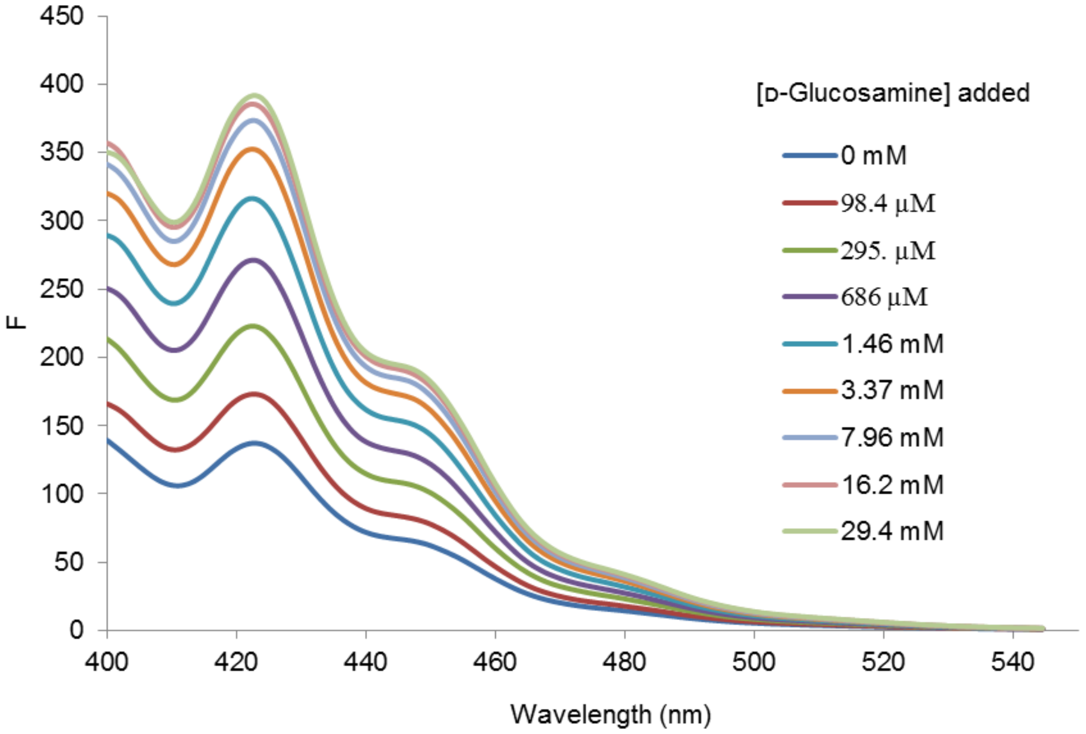

Figure S103. Fluorescence titration of receptor **5** (15.0 μM) with D-glucosamine **13** in H_2O (pH = 7.05) at 298 K. Excitation wavelength: 395 nm.

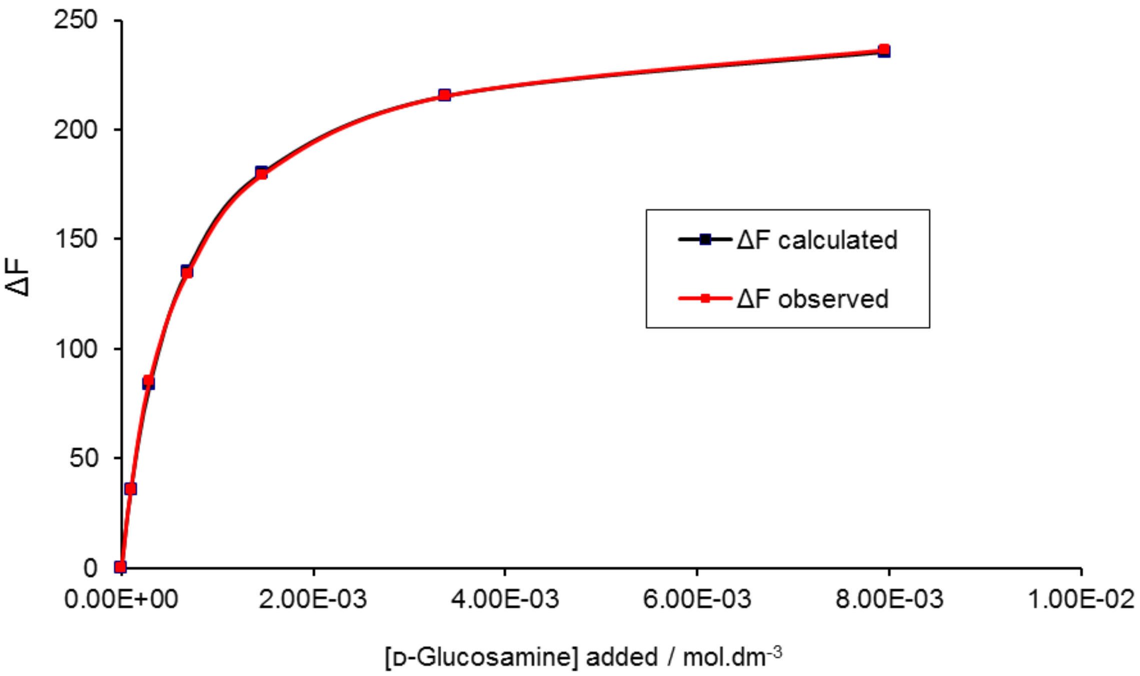

Figure S104. Experimental and calculated values for the fluorescence binding study of receptor **5** (15.0 μM) with D-glucosamine **13** in H_2O (pH = 7.05) at 298 K. Excitation: 395 nm. Emission observed at: 423 nm. K_a = 1700 M^{-1} ± 2.74 %. F/F^o = 2.84.

Receptor 6 Carbohydrate Binding Studies

NMR spectra and binding analyses

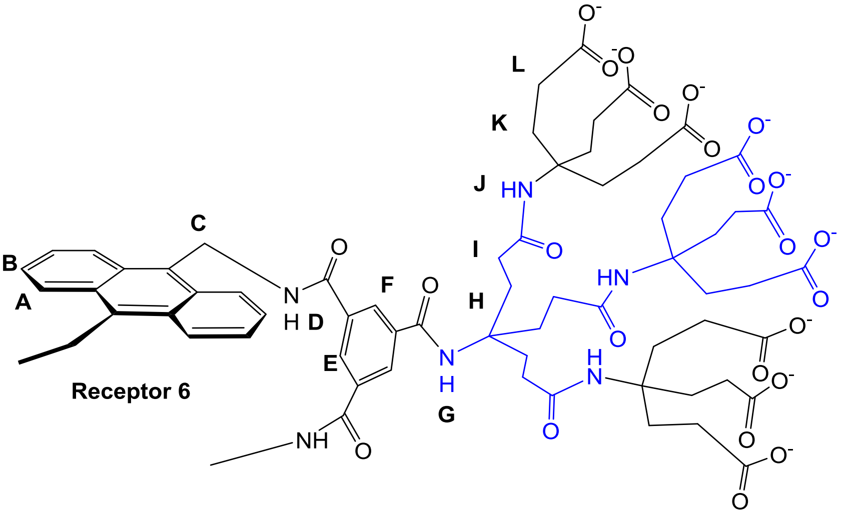

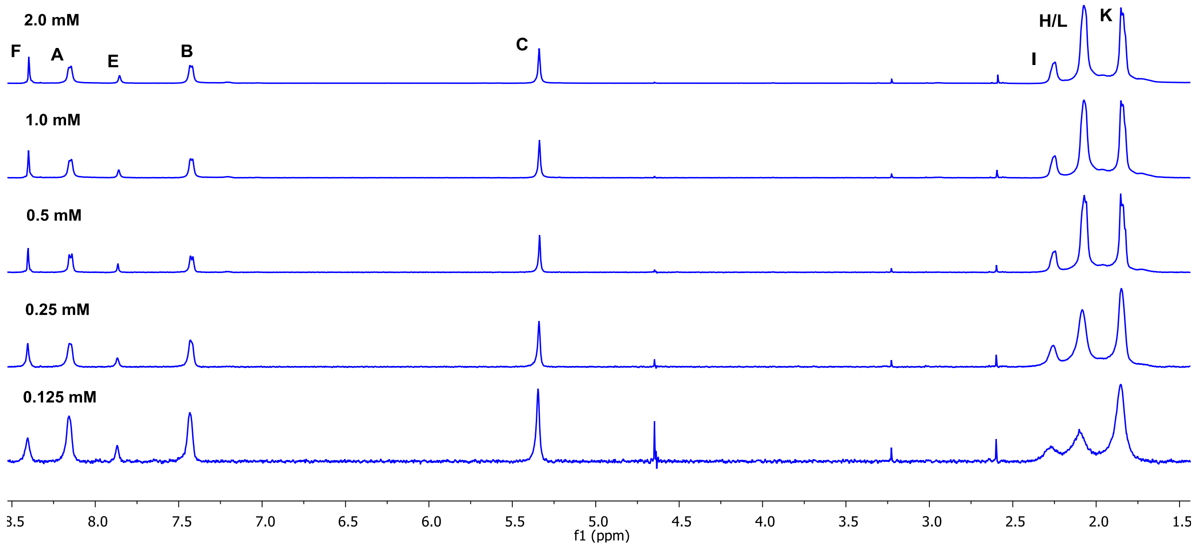

Figure S105. Partial ^{1}H NMR spectra of receptor **6** at concentrations from 0.25 mM to 2 mM in D_2O at 298 K, with assignments.

D-Glucose 3

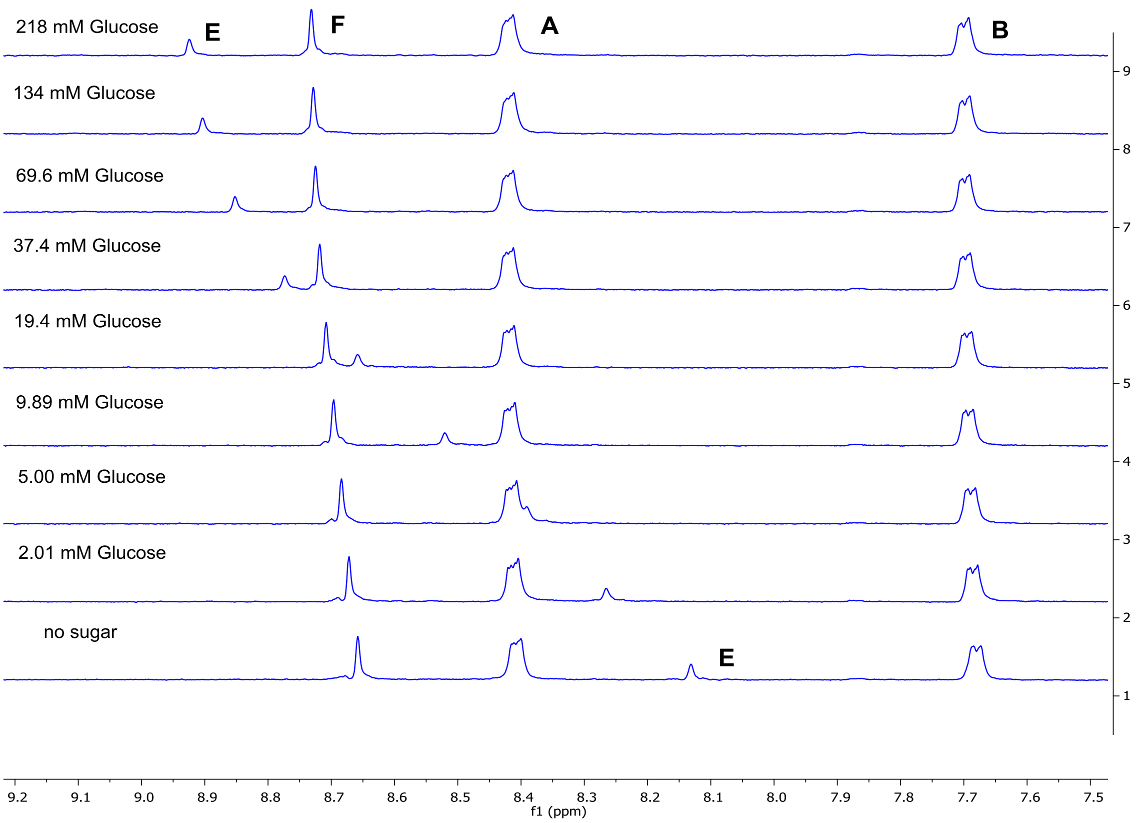

Figure S106. Partial ^{1}H NMR spectra from the titration of receptor **6** (0.23 mM) with D-glucose **3** in D_2O at 298 K.

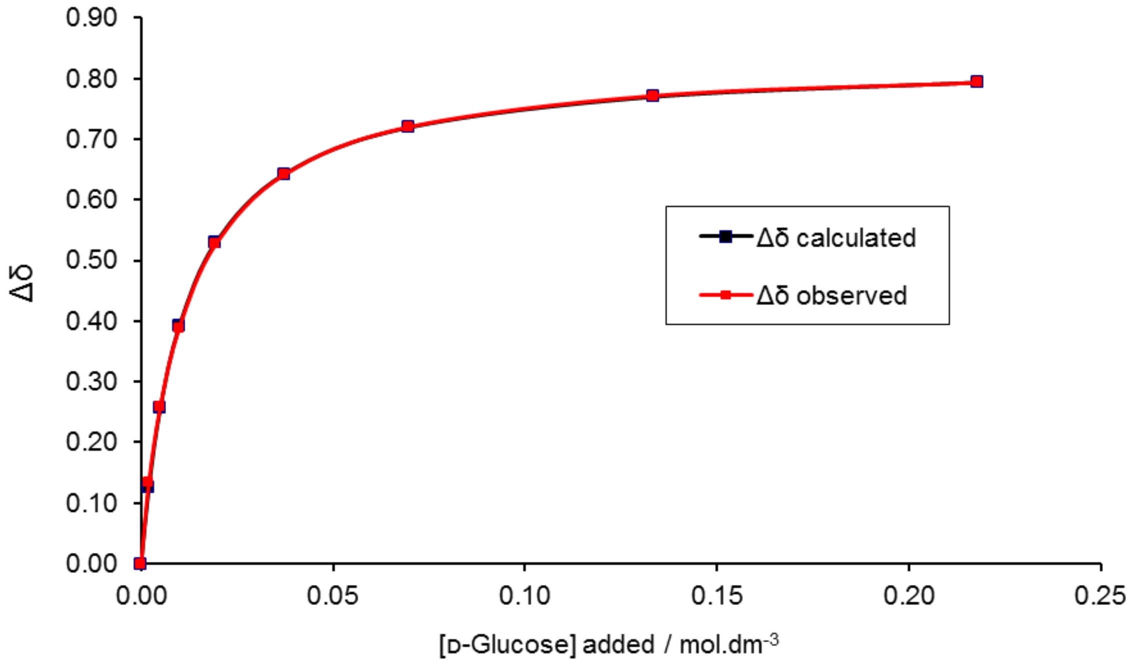

Figure S107. Experimental and calculated values for the NMR binding study of receptor **6** (0.23 mM) with D-glucose **3** in D_2O. Proton E: K_a = 90 M^{-1} ± 2%, δΔ= 0.83.

Methyl β-ᴅ-glucoside

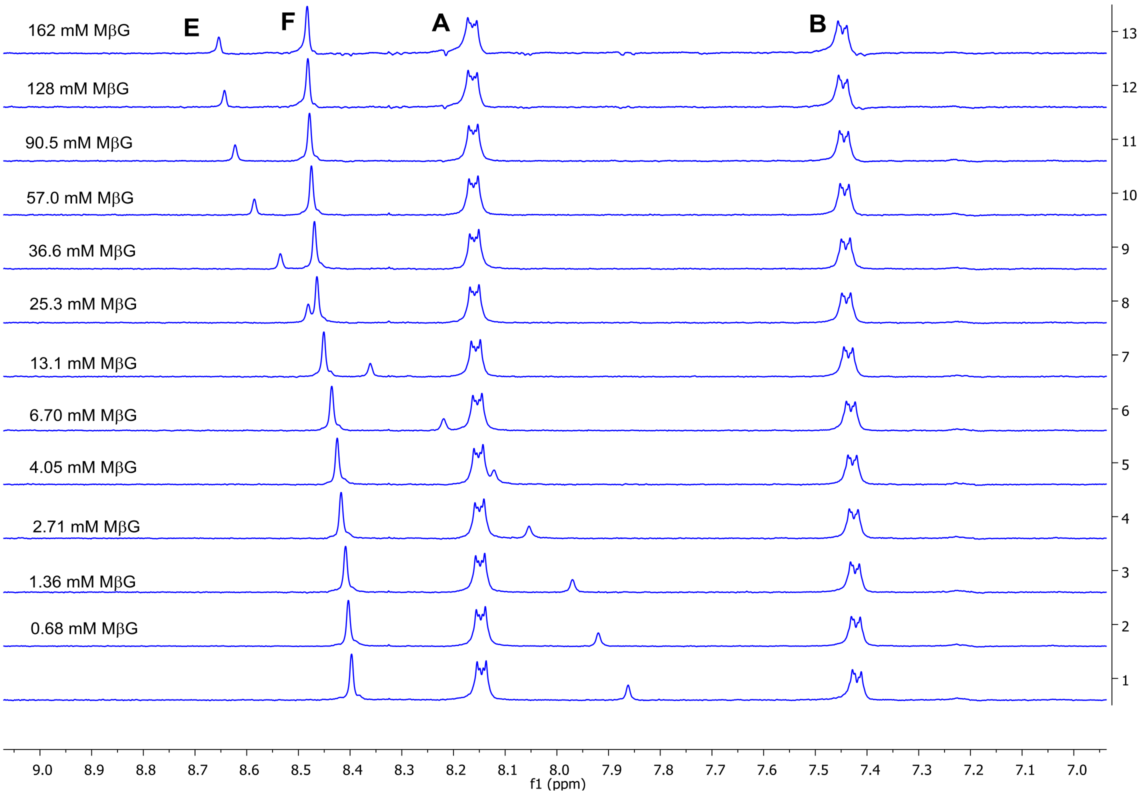

Figure S108. Partial ^{1}H NMR spectra from the titration of receptor **6** (0.44 mM) with methyl β-D-glucoside in D_2O at 298 K.

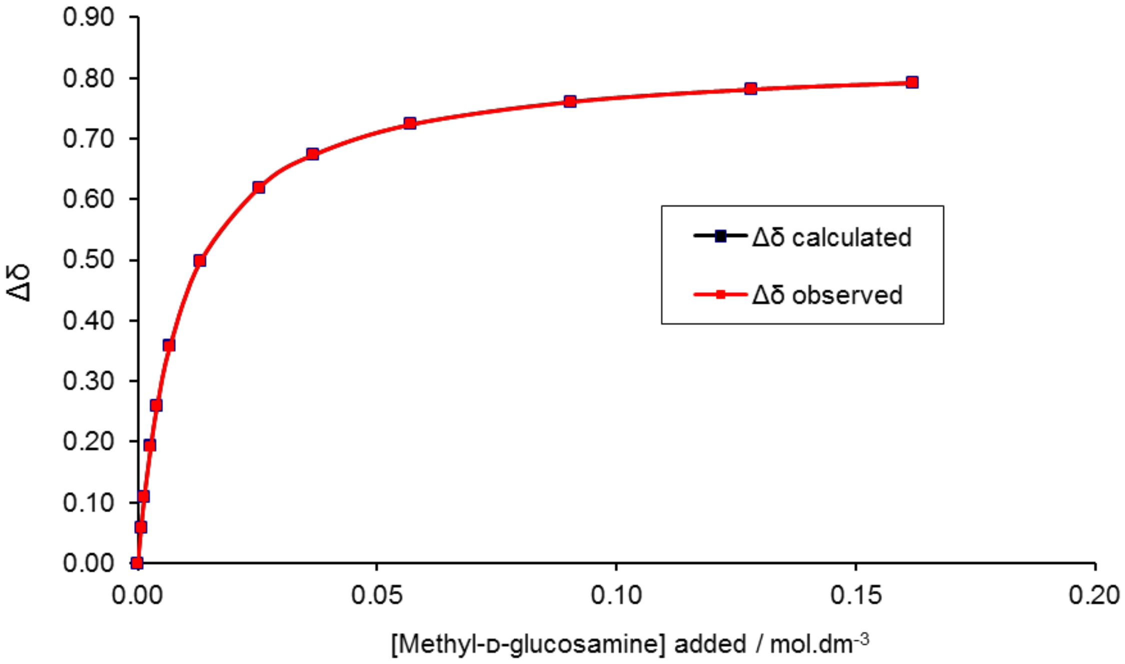

Figure S109. Experimental and calculated values for the NMR binding study of receptor **6** (0.44 mM) with methyl β-D-glucoside in D_2O at 298 K. Proton E. K_a = 115 M^{-1} ± 2%, δΔ= 0.84.

N-Acetyl-D-glucosamine 15

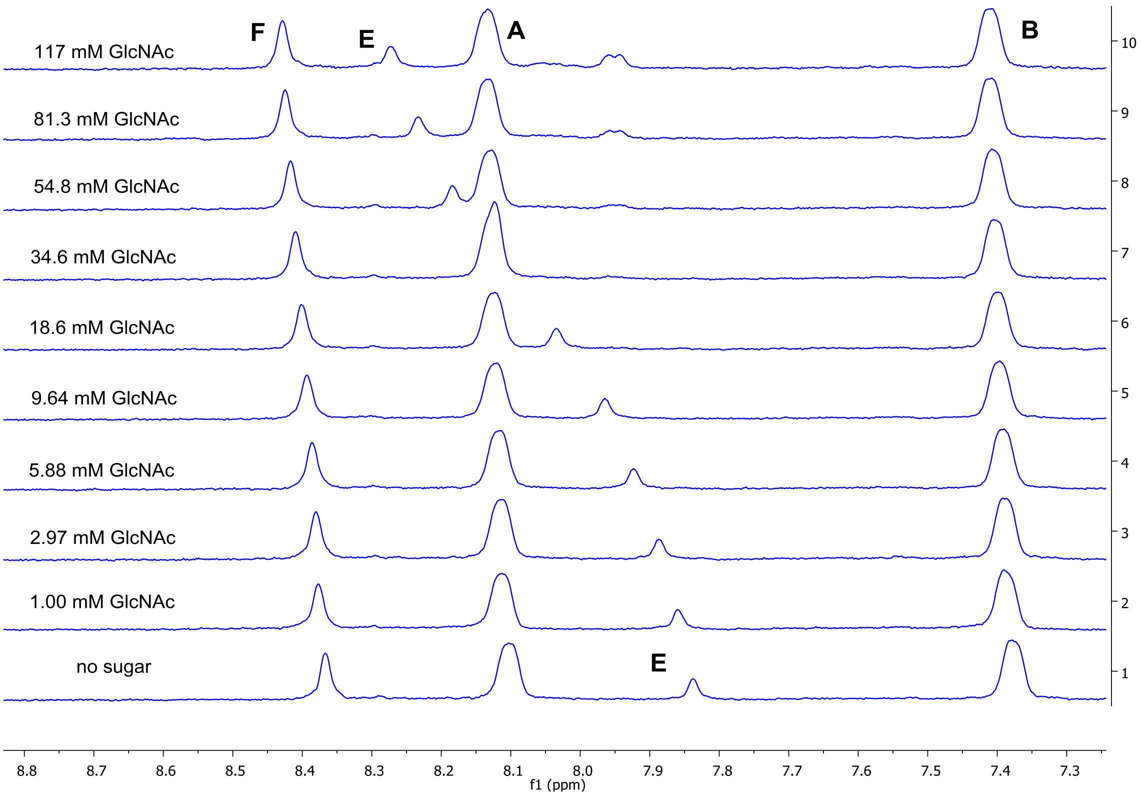

Figure S110. Partial ^1H NMR spectra from the titration of receptor **6** (0.27 mM) with GlcNAc **15** in D_2O at 298 K.

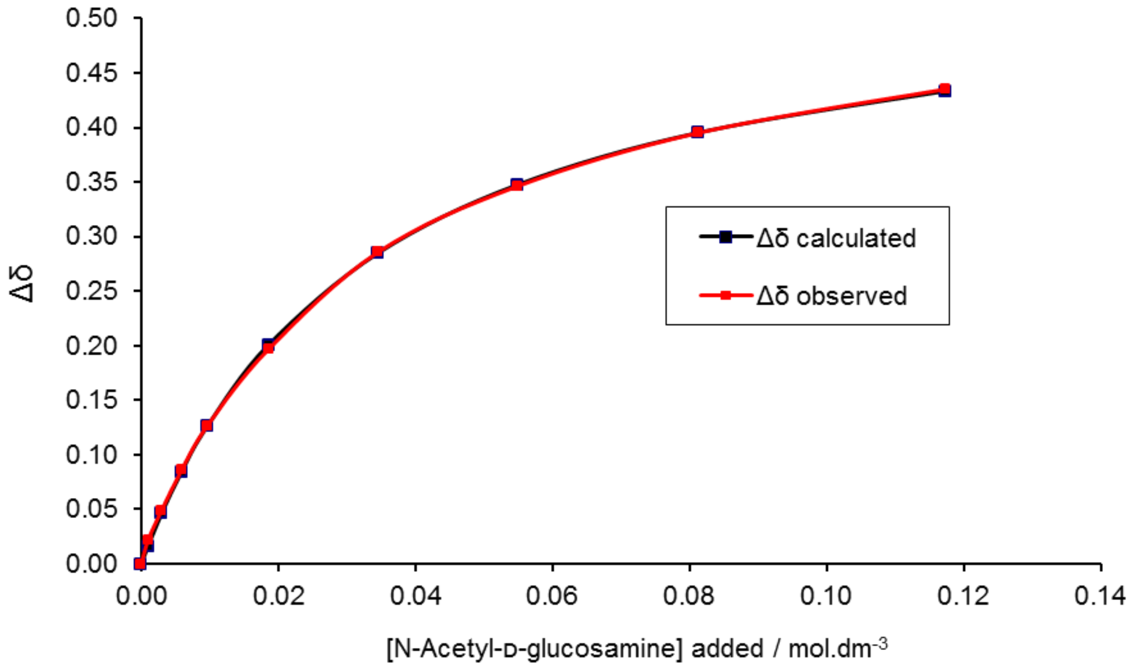

Figure S111. Experimental and calculated values for the NMR binding study of receptor **6** (0.27 mM) with GlcNAc **15** in D_2O at 298 K. Proton E: K_a = 31 M^{-1} ± 12%, Δδ = 0.55.

D-Galactose

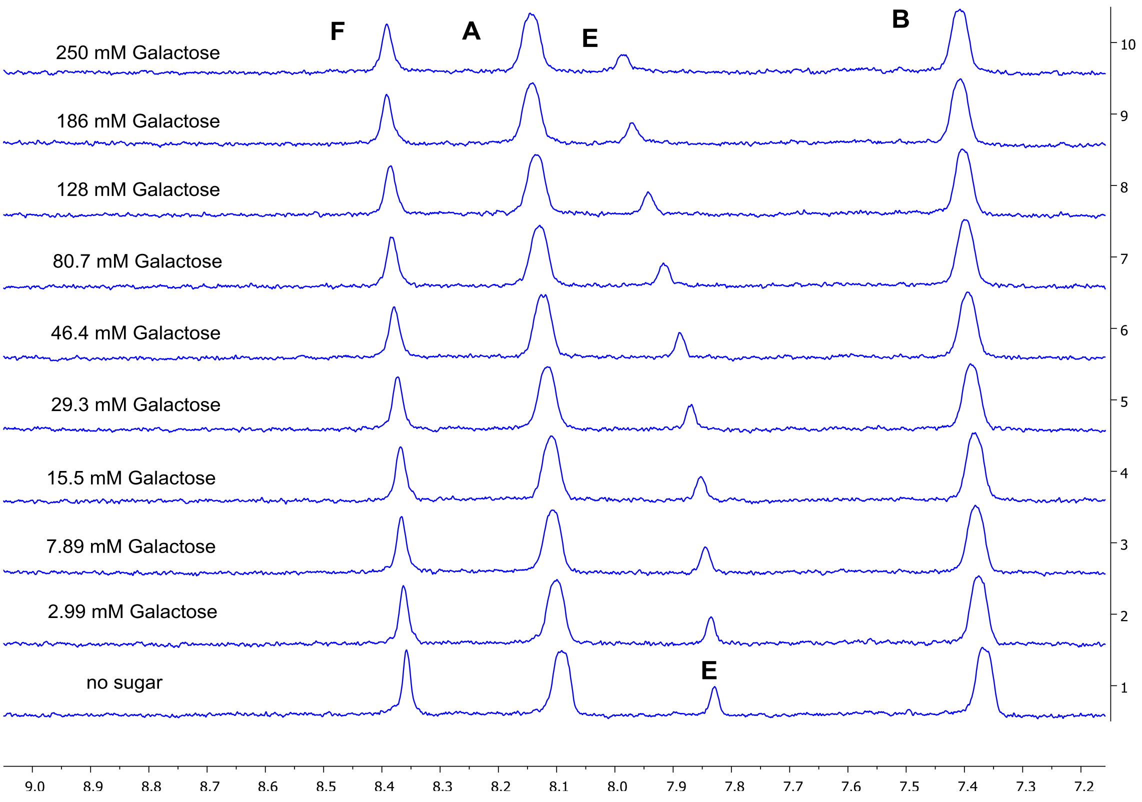

Figure S112. Partial ^{1}H NMR spectra from the titration of receptor **6** (0.13 mM) with D-galactose D_2O at 298 K.

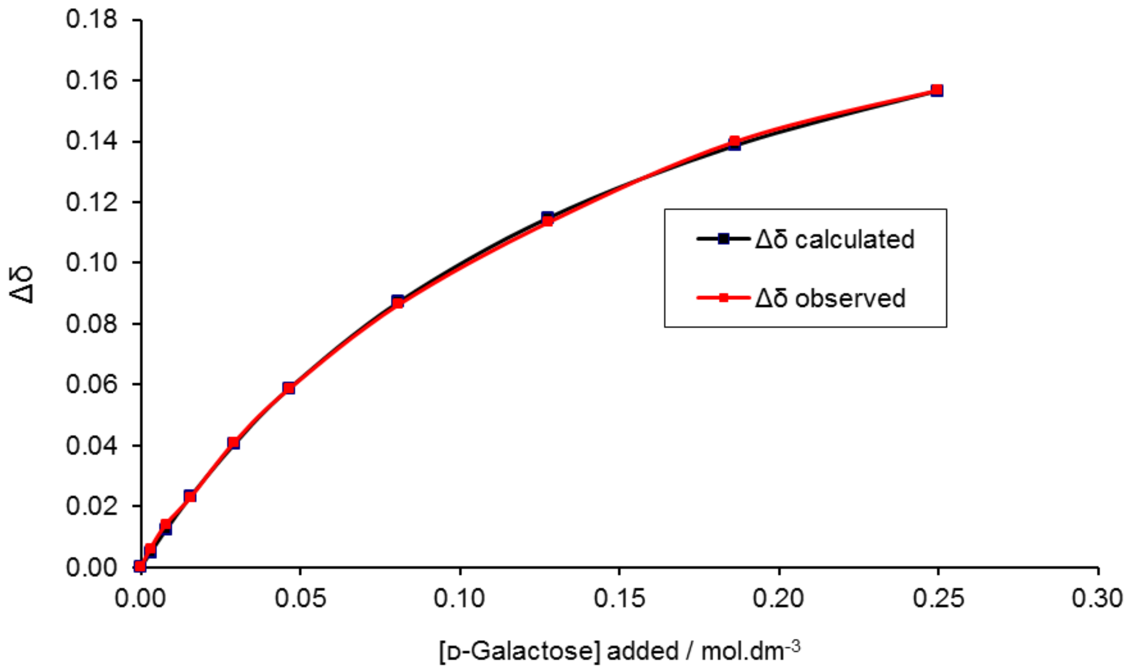

Figure S113. Experimental and calculated values for the NMR binding study of receptor **6** (0.13 mM) with D-galactose in D_2O at 298 K. Proton E: K_a = 6 M^{-1} ± 11%, Δδ = 0.25.

D-Mannose

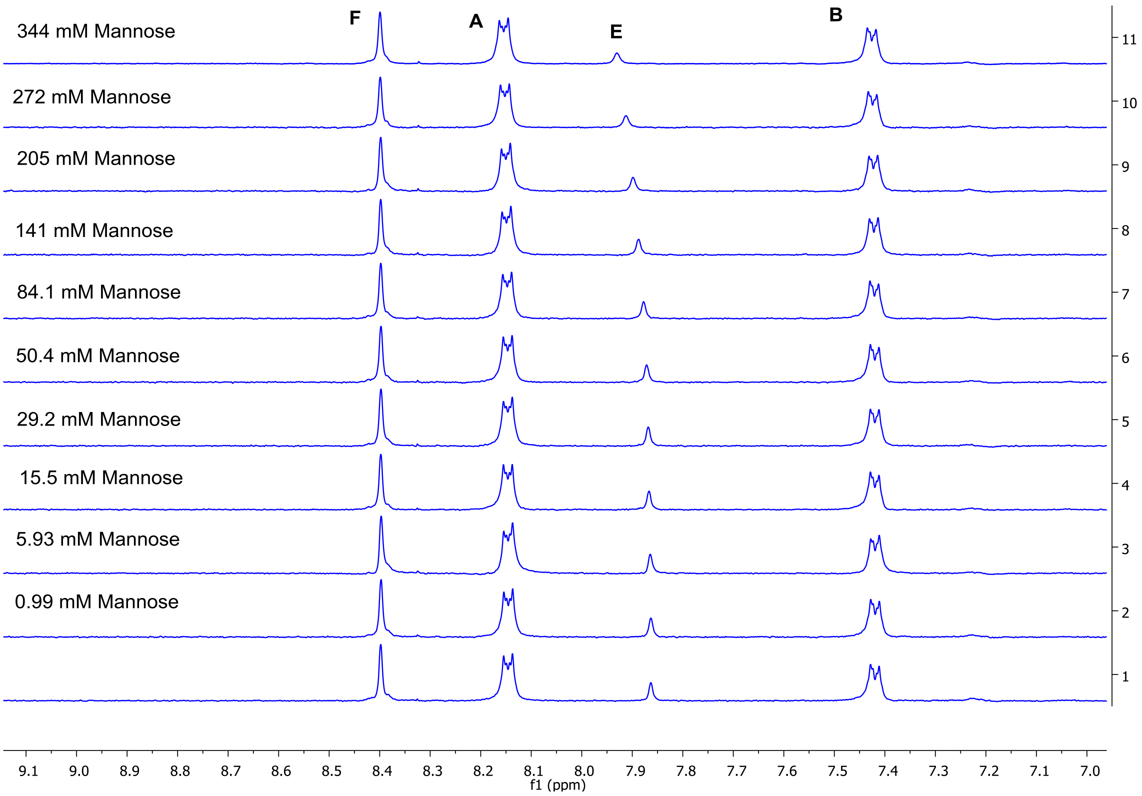

Figure S114. Partial ^{1}H NMR spectra from the titration of receptor **6** (0.52 mM) with D-mannose in D_2O at 298 K.

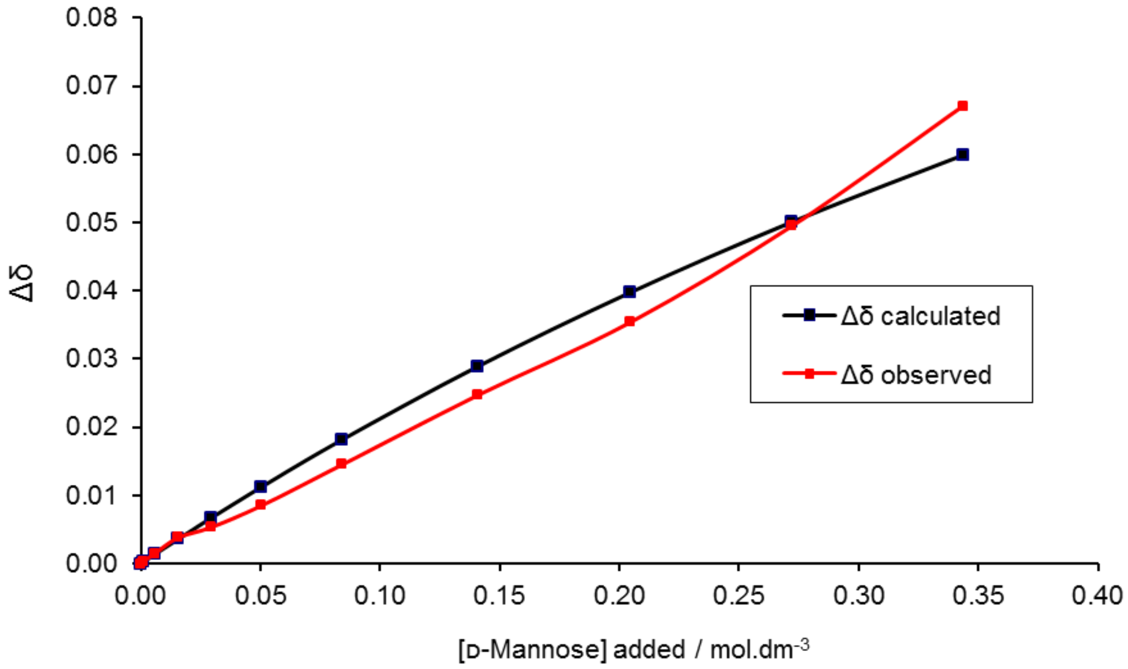

Figure S115. Experimental and calculated values for the NMR binding study of receptor **6** (0.52 mM) with D-mannose in D_2O. K_a too small to be evaluated with reasonable accuracy.

D-Maltose

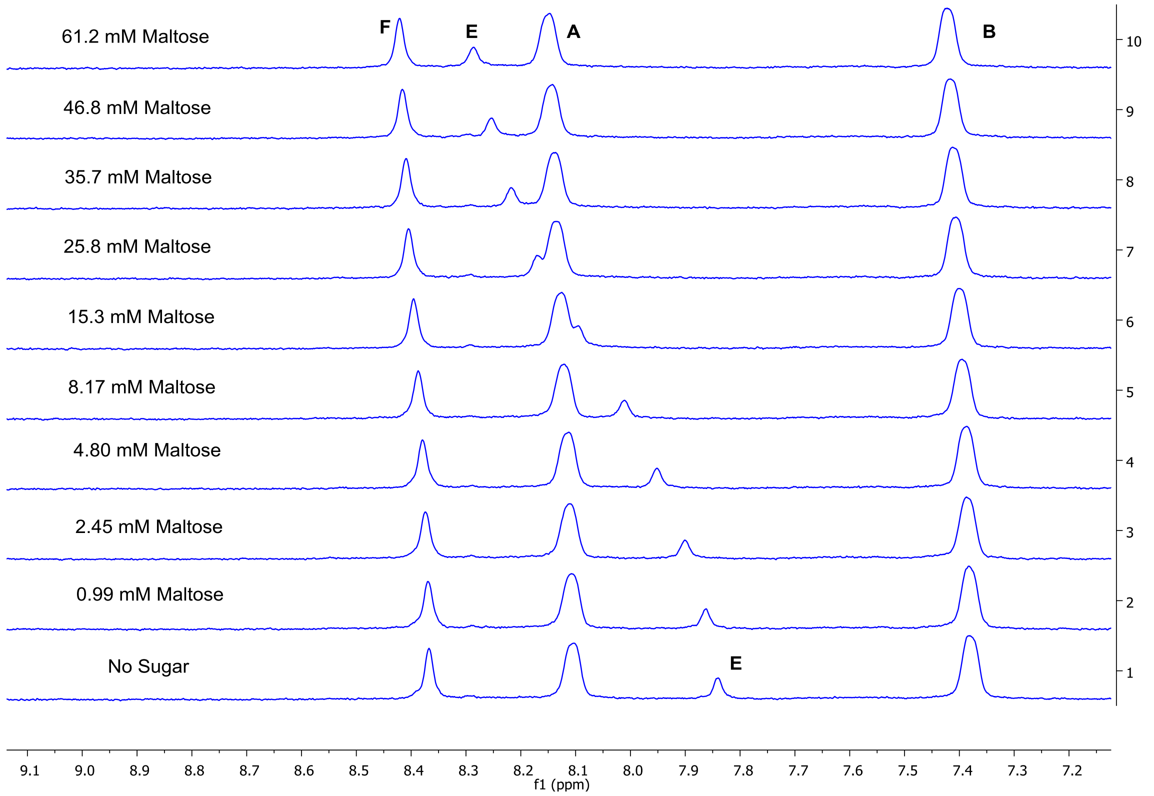

Figure S116. Partial ^{1}H NMR spectra from the titration of receptor **6** (0.29 mM) with D-maltose in D_2O at 298 K.

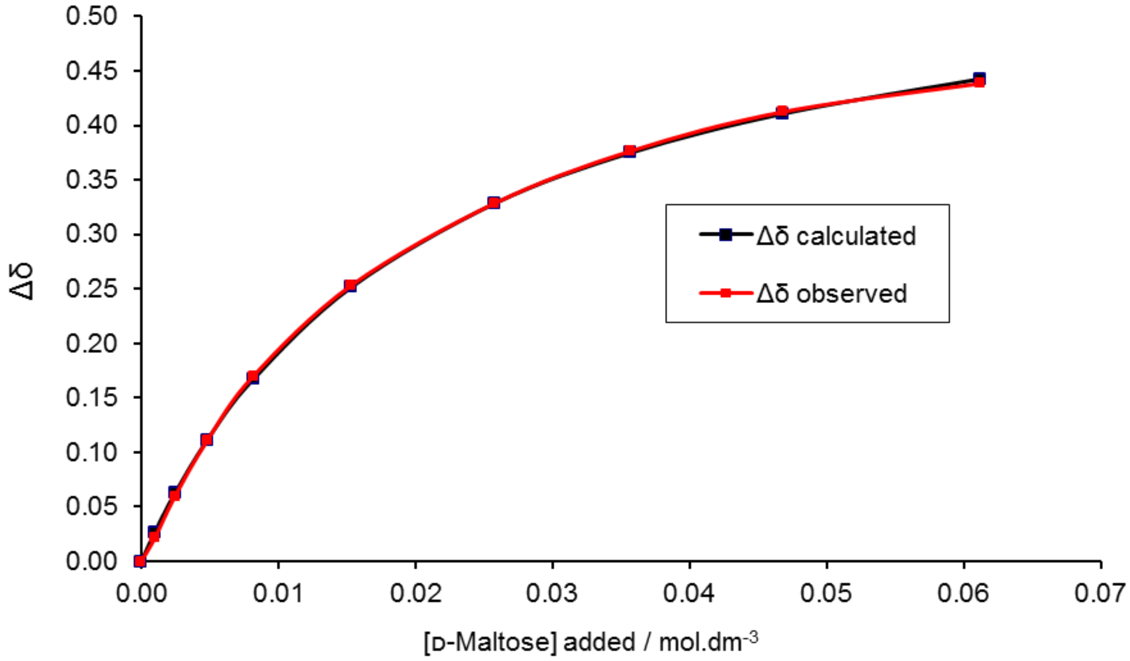

Figure S117. Experimental and calculated values for the NMR binding study of receptor **6** (0.29 mM) with D-maltose in D_2O at 298 K. Proton E: K_a = 49 M^{-1} ± 7%, Δδ = 0.59.

D-Cellobiose

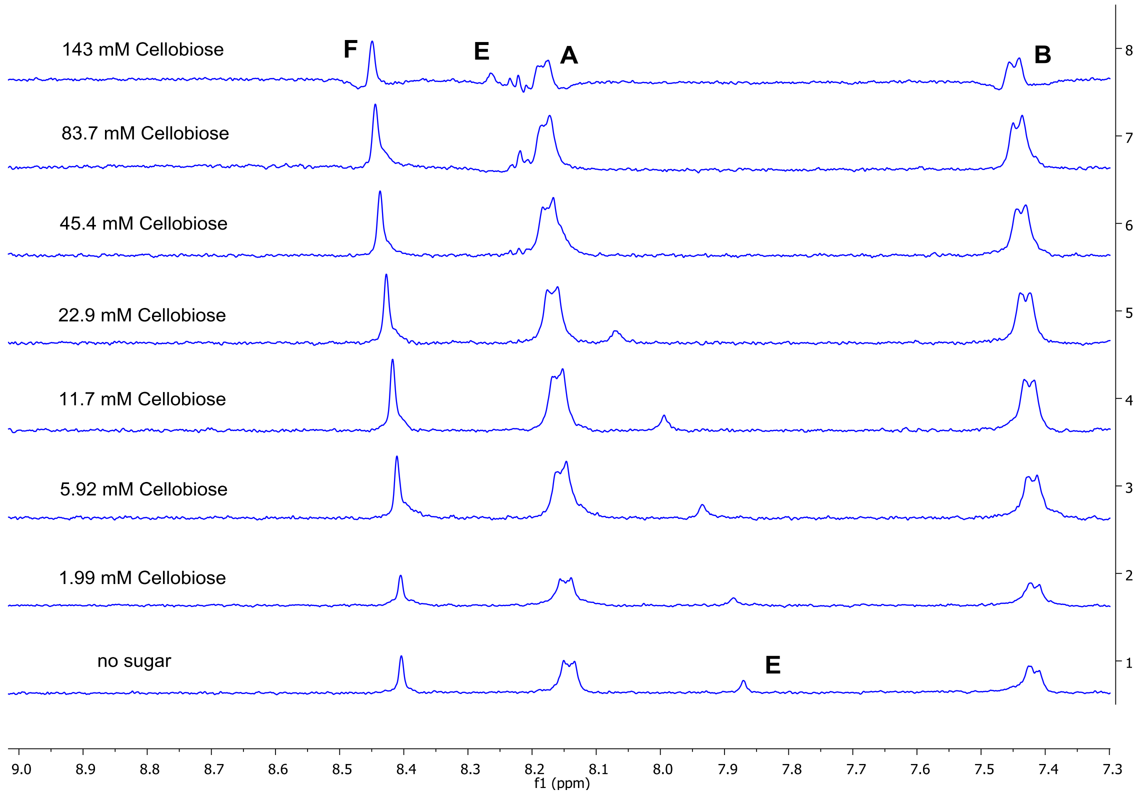

Figure S118. Partial ^{1}H NMR spectra from the titration of receptor **6** (0.11 mM) with D-cellobiose D_2O at 298 K.

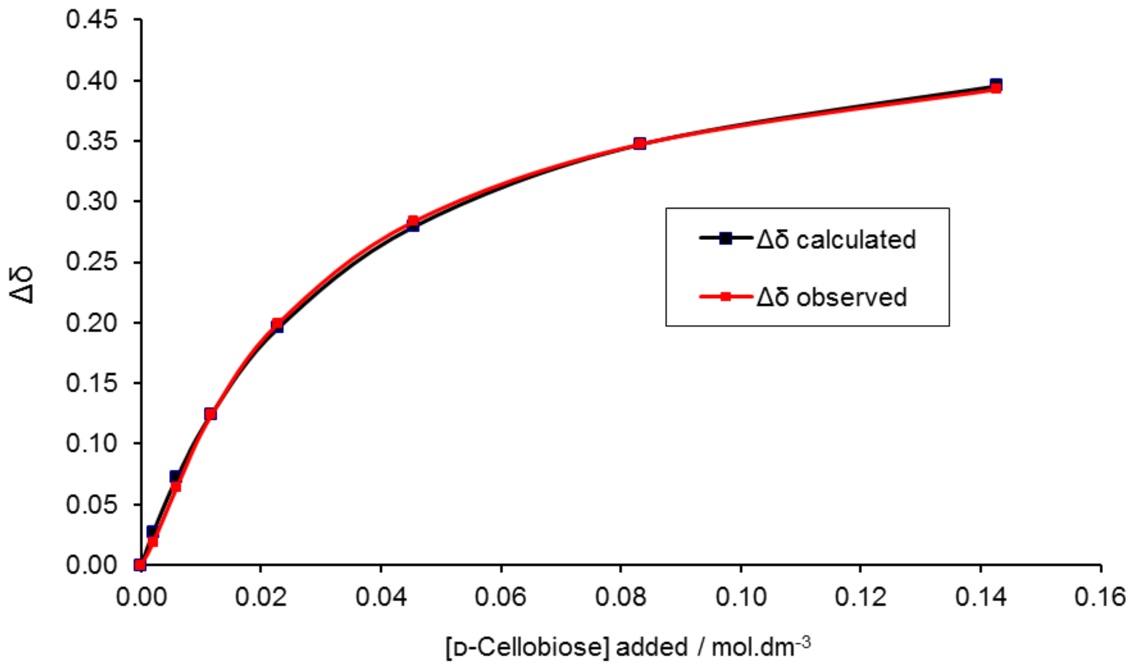

Figure S119. Experimental and calculated values for the NMR binding study of receptor **6** (0.11 mM) with D-cellobiose in D_2O at 298 K. Proton E: K_a = 29 M^{-1} ± 1%, Δδ = 0.49.

D-Lactose

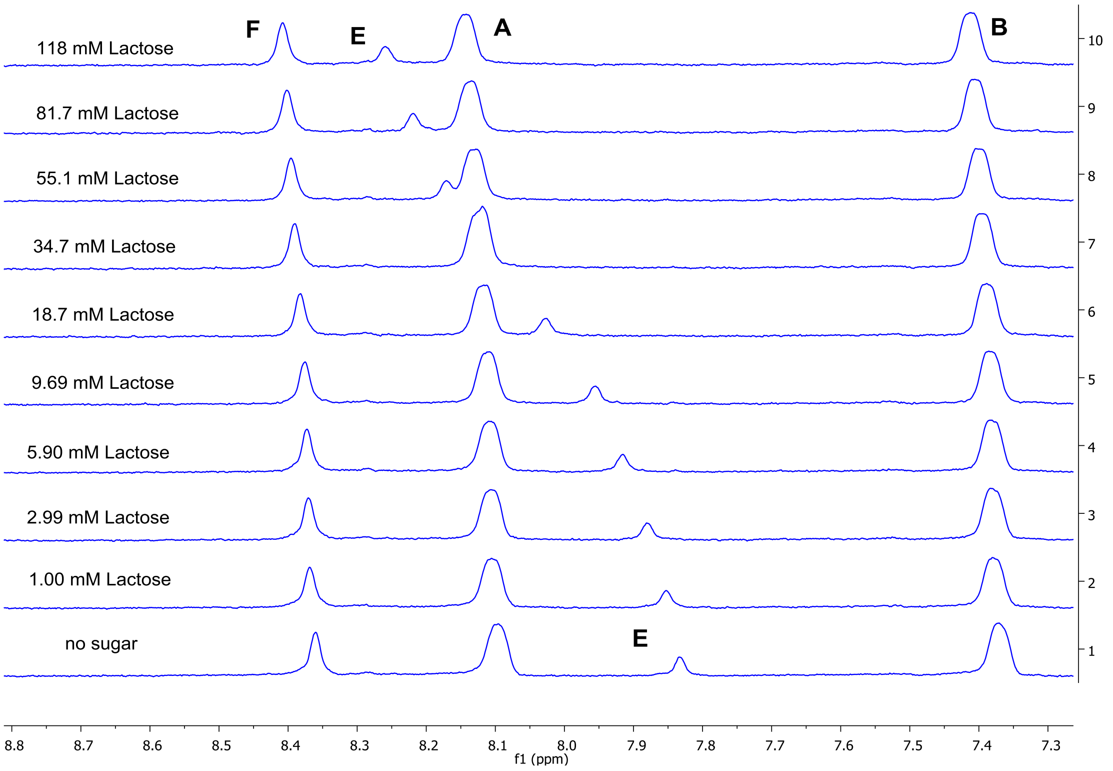

Figure S120. Partial ^1H NMR spectra from the titration of receptor **6** (0.11 mM) with D-Lactose D_2O at 298 K.

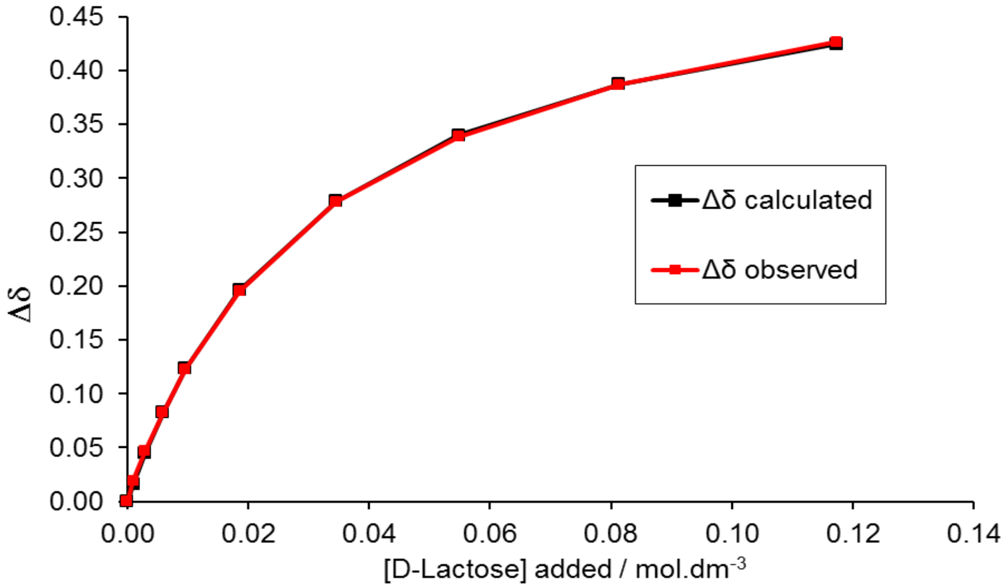

Figure S121. Experimental and calculated values for the NMR binding study of receptor **6** (0.11 mM) with D-Lactose in D_2O at 298 K. Proton E: K_a = 31 M^{-1} ± 8%, Δδ = 0.54.

D-Glucosamine 13

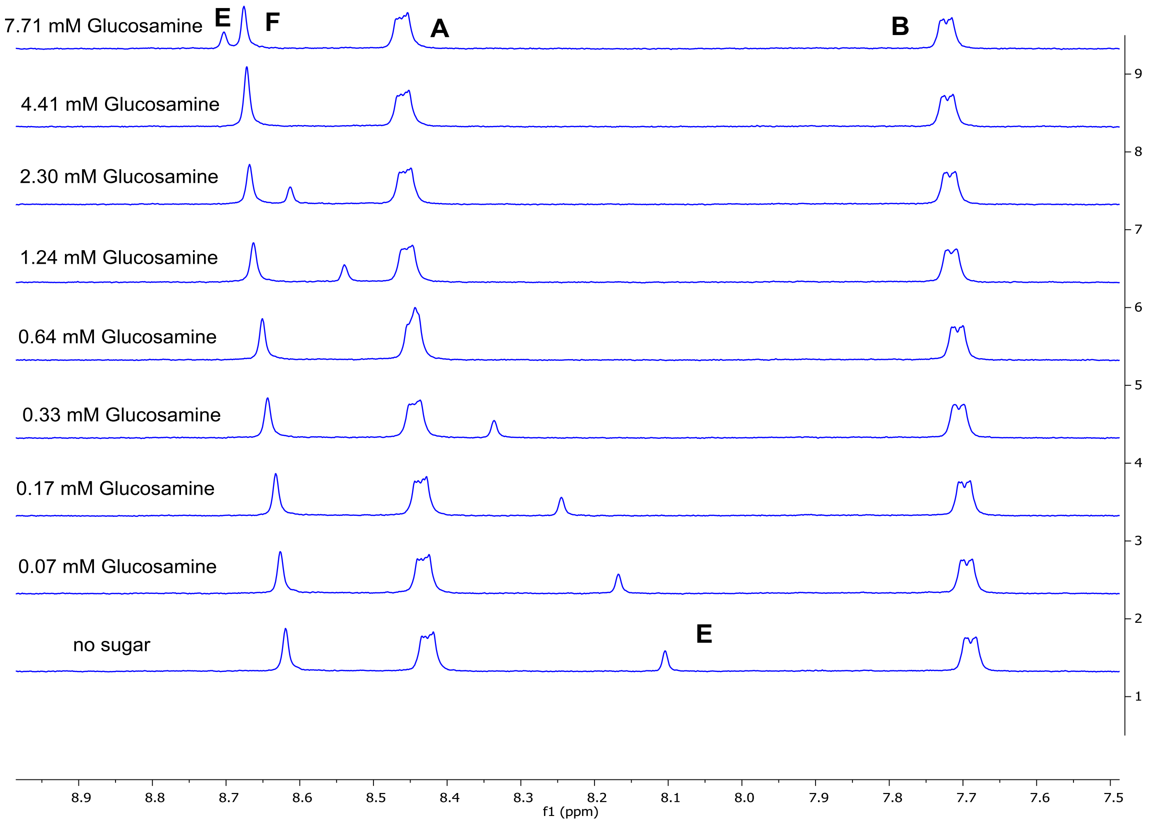

Figure S122. Partial ^{1}H NMR spectra from the titration of receptor **6** (0.20 mM) with D-glucosamine **13** in D_2O (pH = 7) at 298 K.

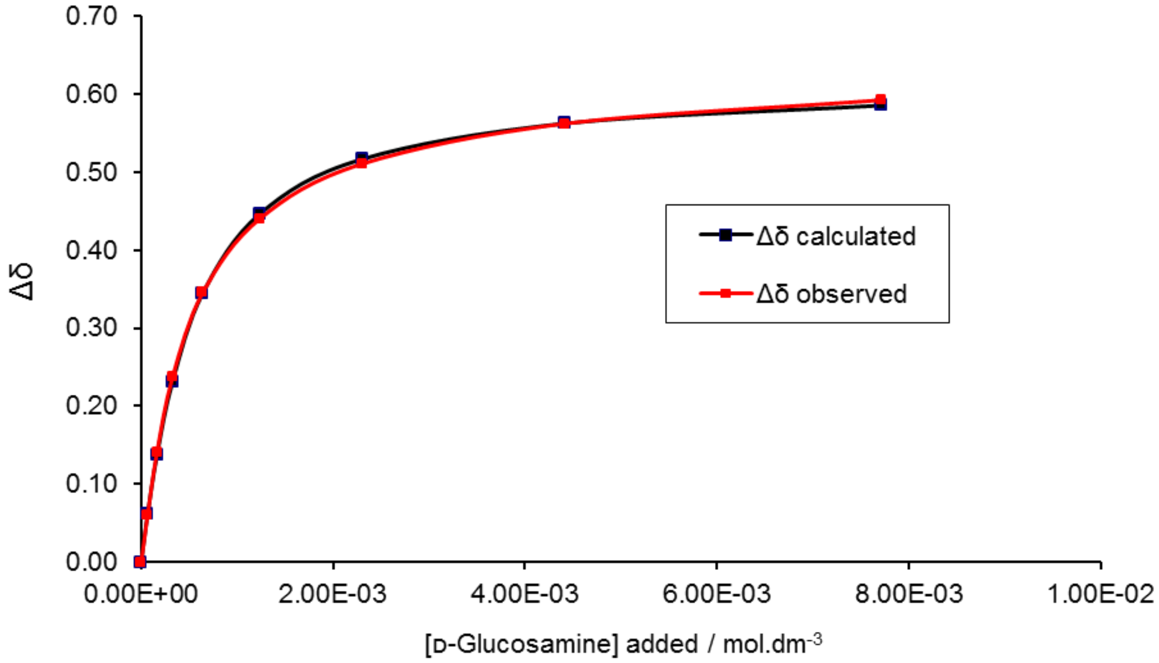

Figure S123. Experimental and calculated values for the NMR binding study of receptor **6** (0.20 mM) with D-glucosamine **13** in D_2O (pH = 7) at 298 K. Proton E: K_a = 2400 M^{-1} ± 10%, $\Delta\delta$ = 0.62

D-Glucosamine (NaCl 20 mM)

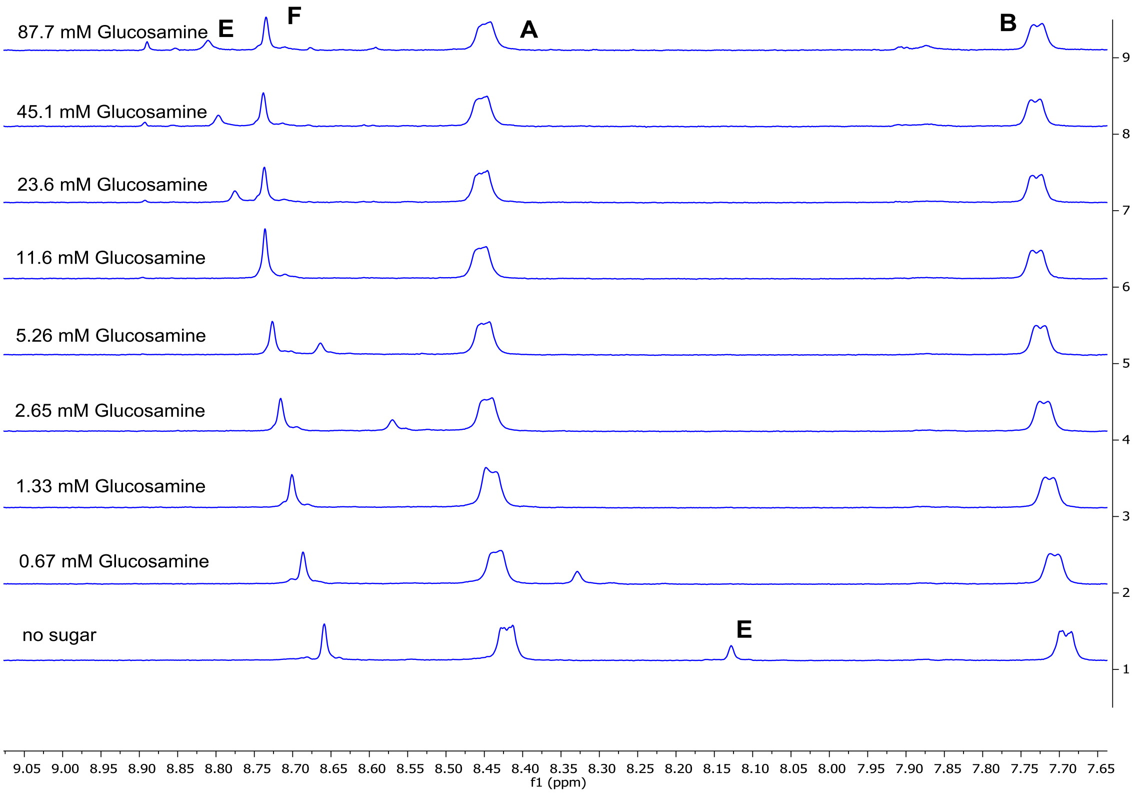

Figure S124. Partial ^{1}H NMR spectra from the titration of receptor **6** (0.19 mM) with D-glucosamine **13** in D_2O (pH = 7, 20 mM, NaCl) at 298 K

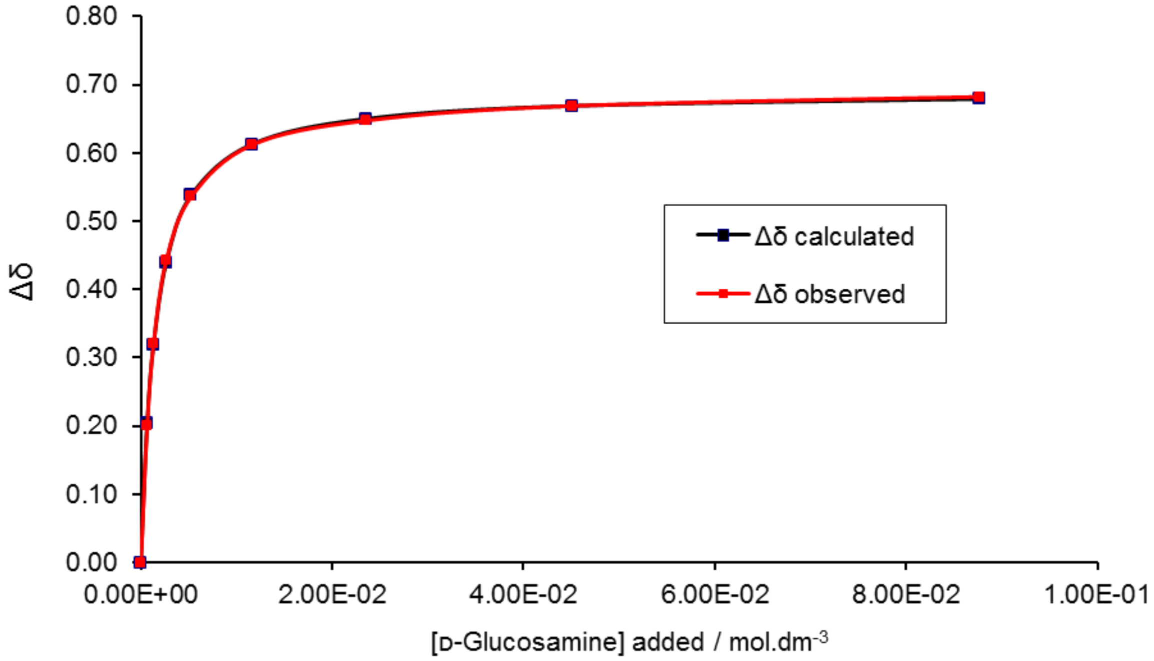

Figure S125. Experimental and calculated values for the NMR binding study of receptor **6** (0.19 mM) with D-glucosamine **13** in D_2O (pH = 7, 20 mM, NaCl) at 298 K. Proton E: K_a = 690 M^{-1} ± 13%, Δδ = 0.69.

D-Glucosamine (NaCl 154 mM)

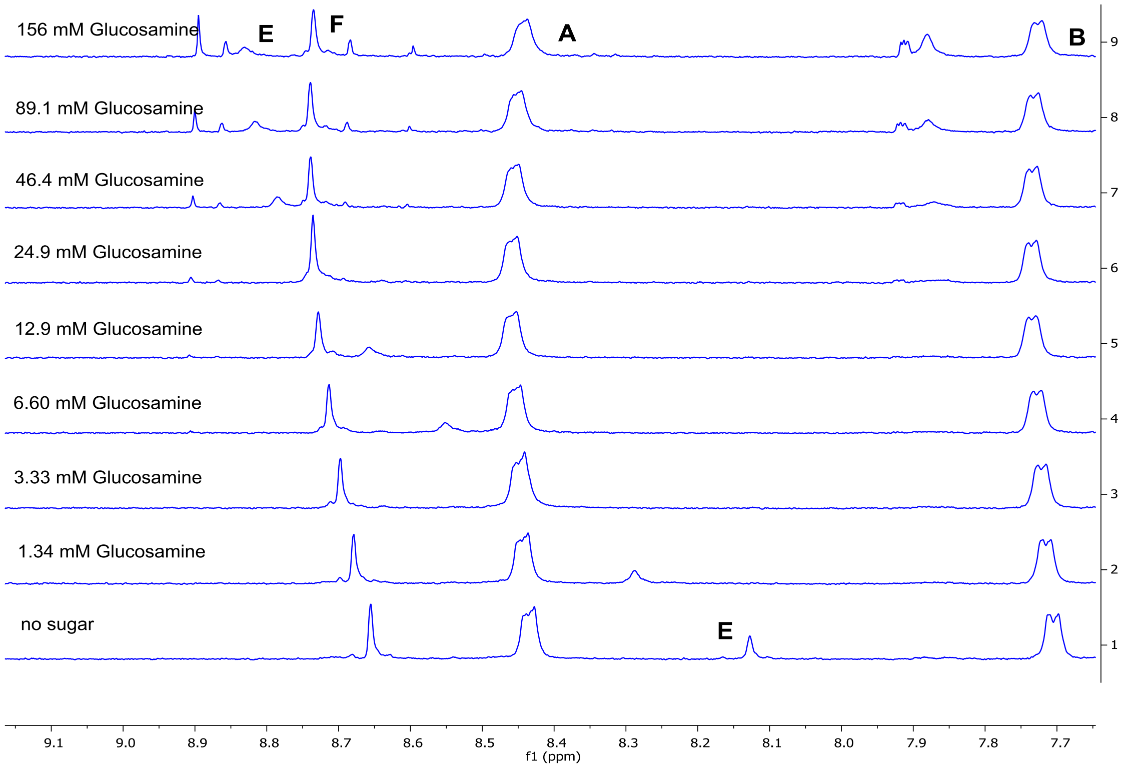

Figure S126. Partial ^{1}H NMR spectra from the titration of receptor **6** (0.19 mM) with D-glucosamine **13** in D_2O (pH = 7, 154 mM, NaCl) at 298 K.

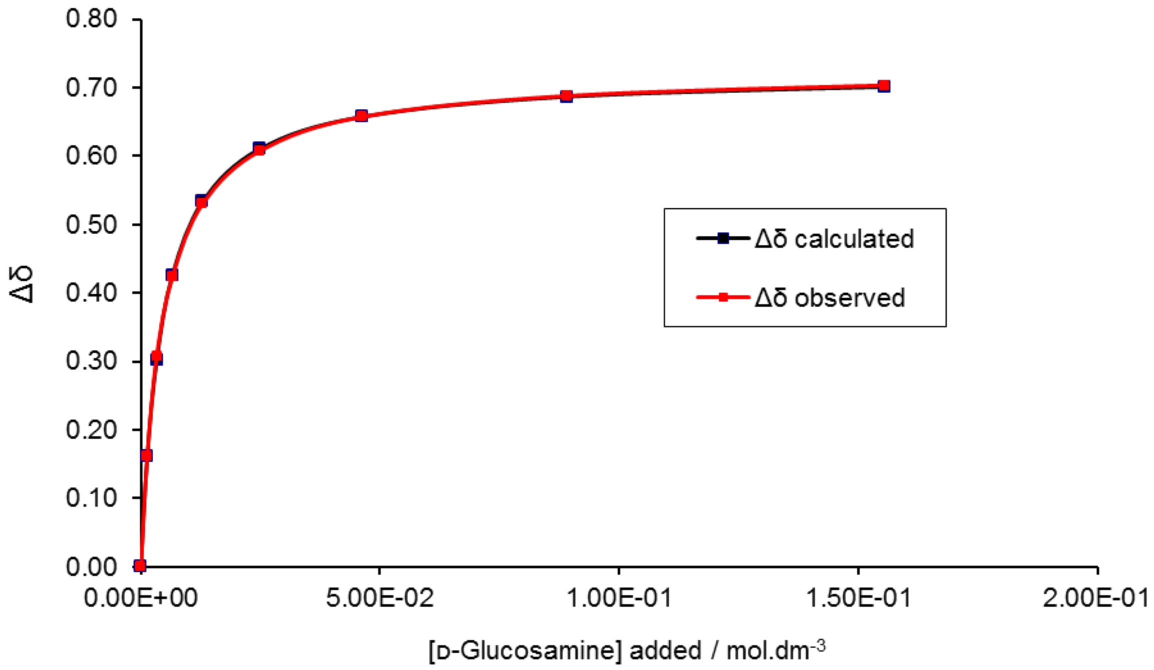

Figure S127. Experimental and calculated values for the NMR binding study of receptor **6** (0.19 mM) with D-glucosamine **13** in D_2O (pH = 7, 154 mM, NaCl) at 298 K. Proton E: K_a = 222 M^{-1} ± 5%, Δδ = 0.72.

D-Galactosamine (NaCl 154 mM)

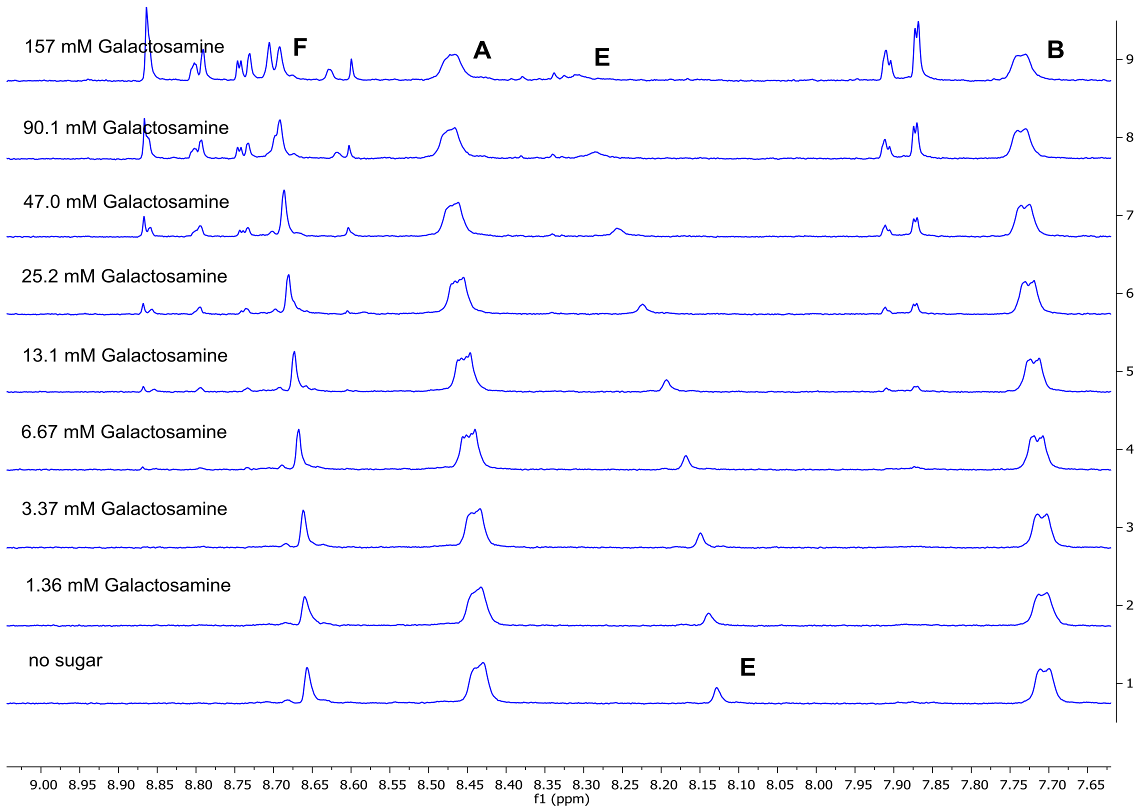

Figure S128. Partial ^{1}H NMR spectra from the titration of receptor **6** (0.19 mM) with D-galactosamine D_2O (pH = 7, 154 mM, NaCl) at 298 K.

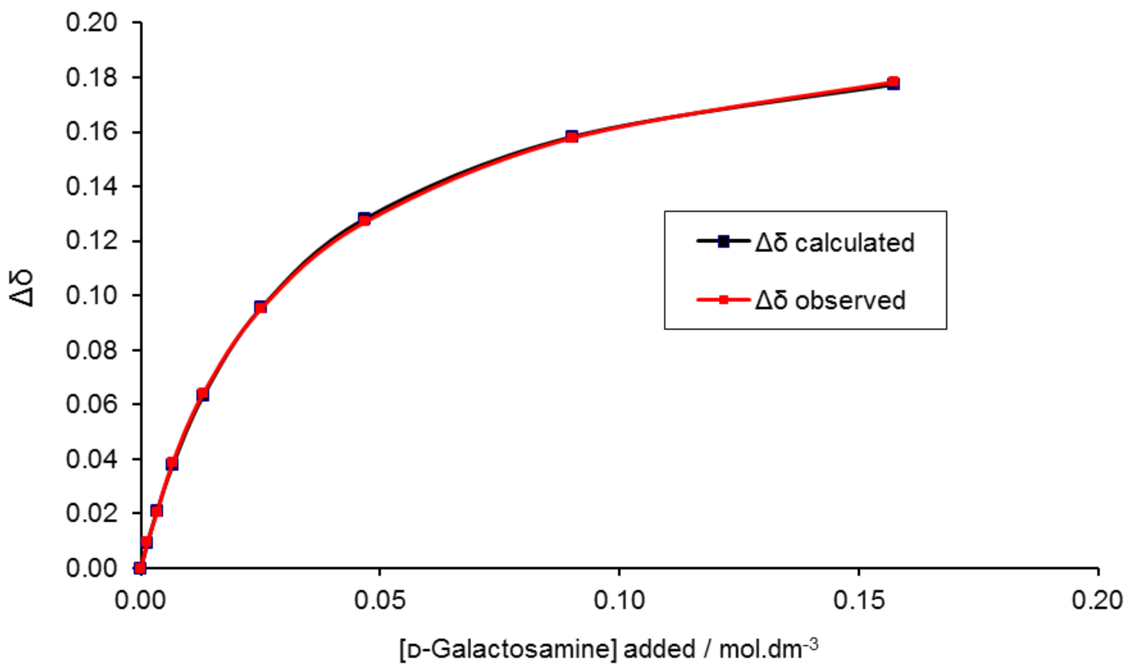

Figure S129. Experimental and calculated values for the NMR binding study of receptor **6** (0.19 mM) with D-galactosamine in D_2O (pH = 7, 154 mM, NaCl) at 298 K. Proton E: K_a = 33 M^{-1} ± 3%, Δδ = 0.21.

D-Glucose 3 (NaCl 154 mM)

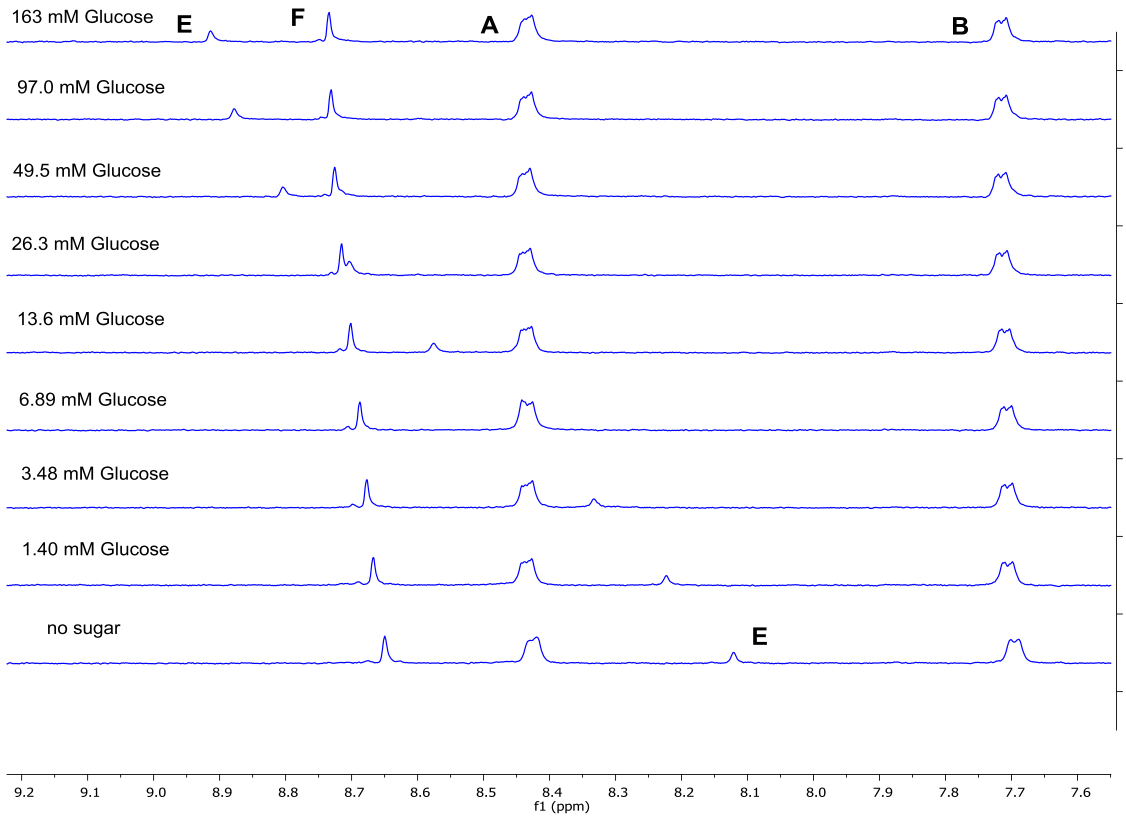

Figure S130. Partial ^{1}H NMR spectra from the titration of receptor **6** (0.19 mM) with D-glucose **3** in D_2O (154 mM, NaCl) at 298 K.

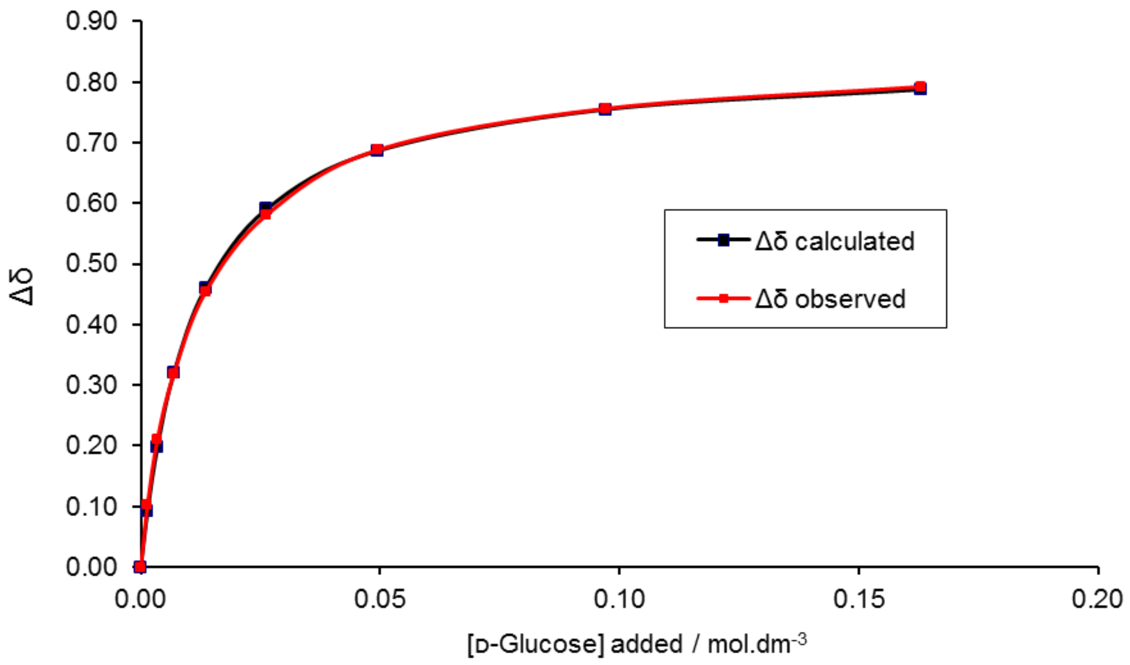

Figure S131. Experimental and calculated values for the NMR binding study of receptor **6** (0.19 mM) with D-glucose **3** in D_2O (154 mM, NaCl) at 298 K. Proton E: K_a = 90 M^{-1} ± 6%, Δδ = 0.84.

Tris-(hydroxymethyl)aminomethane

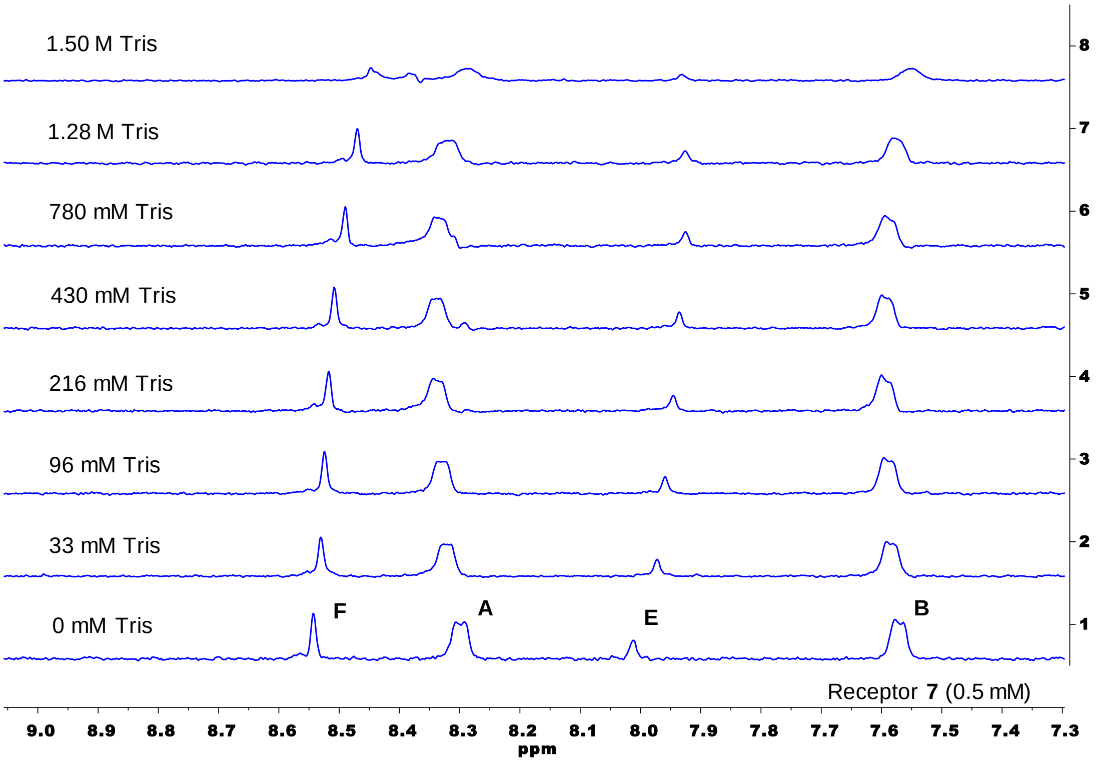

Figure S132. Partial H NMR spectra from the titration of receptor **6** (0.5 mM) with tris-(hydroxymethyl)aminomethane in D_2O (pH = 7) at 298 K. Signal movements are roughly linear with added amine until high concentrations (when some reverse direction).

ITC Titrations

D-Glucose 3

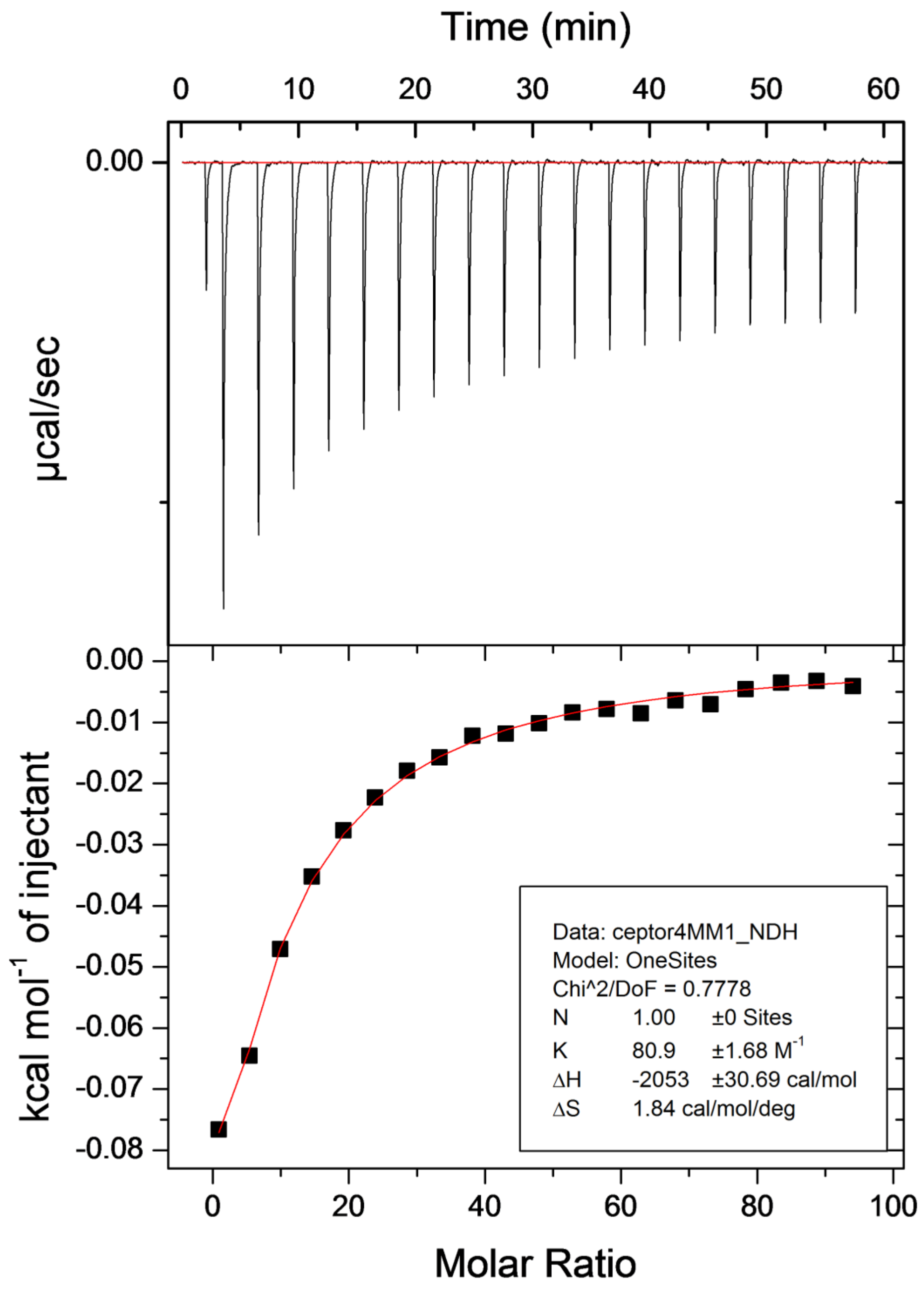

Figure S133. Output from the ITC experiment for the titration of receptor **6** (0.50 mM) with D-glucose **3** (225 mM) in H_2O at 298 K. $K_a = 81\ M^{-1}$.

Methyl β-ᴅ-glucoside

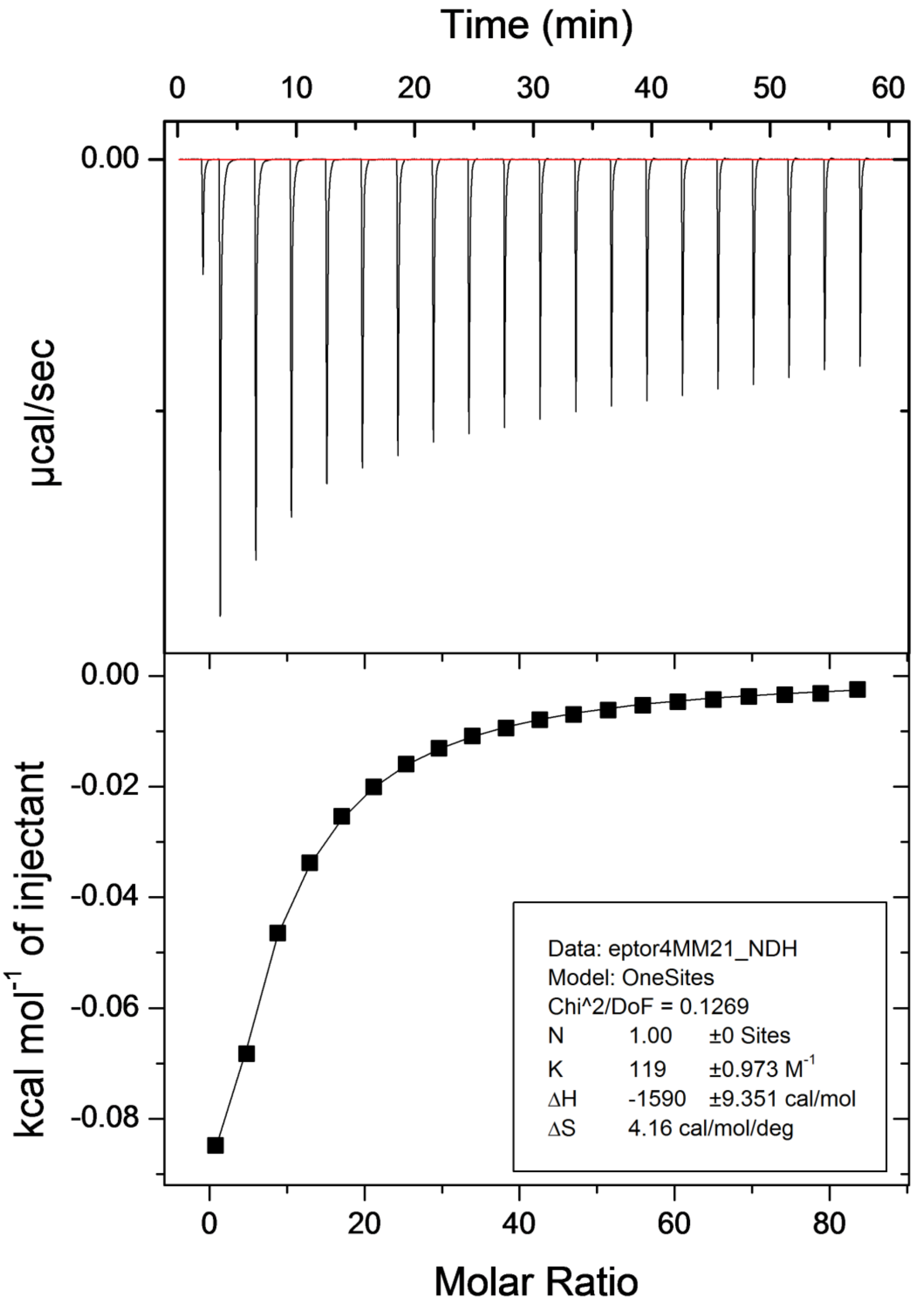

Figure S134. Output from the ITC experiment for the titration of receptor **6** (0.50 mM) with methyl β-D-glucoside (200 mM) in H_2O at 298 K. K_a = 119 M^{-1}.

D-Glucosamine 13 (NaCl 154 mM)

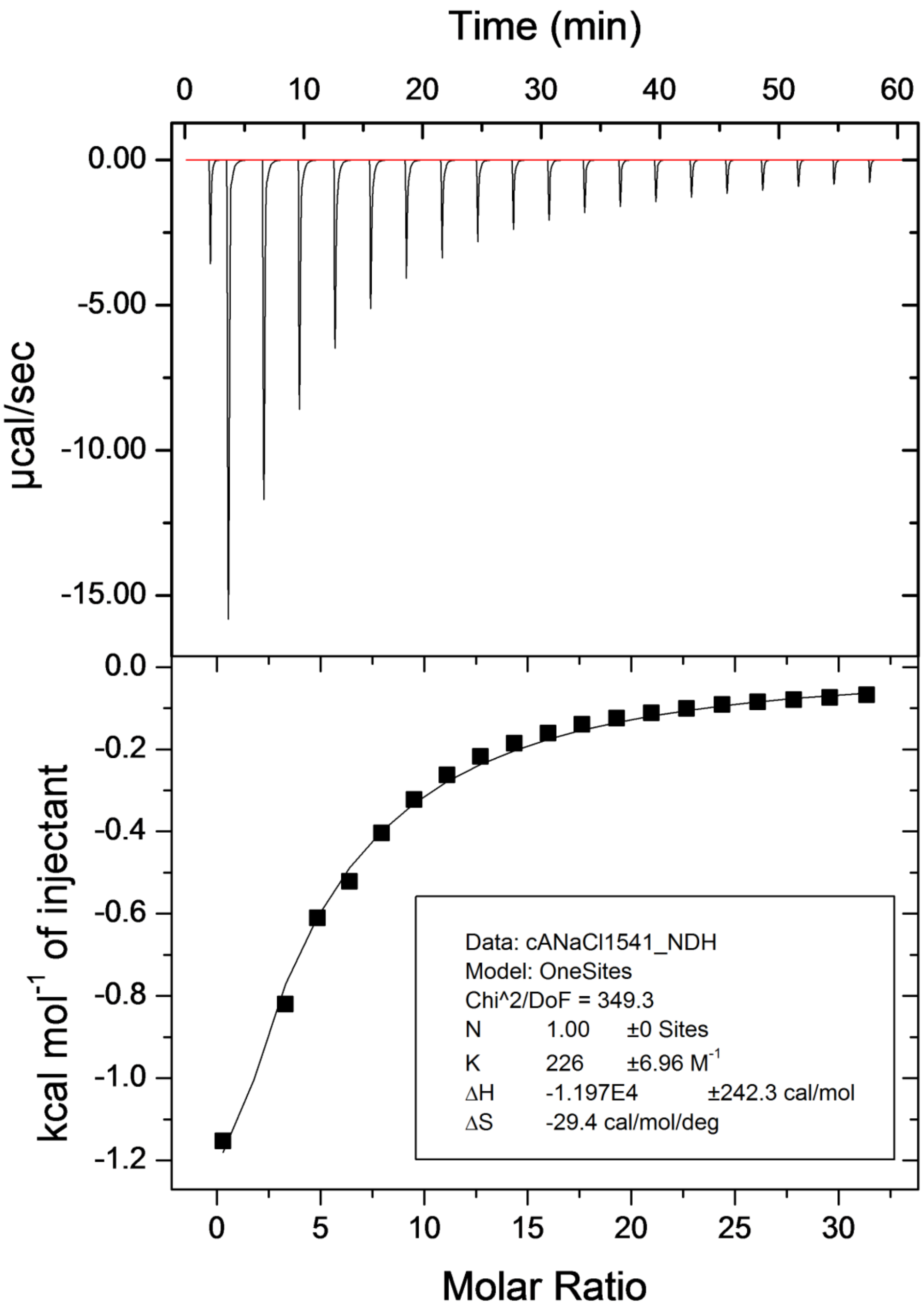

Figure S135. Output from the ITC experiment for the titration of receptor **6** (0.50 mM) with D-glucosamine **13** (75 mM) in H_2O with NaCl (154 mM, pH = 7.1) at 298 K. K_a = 226 M^{-1}.

Fluorescence Titrations

ᴅ-Glucose 3

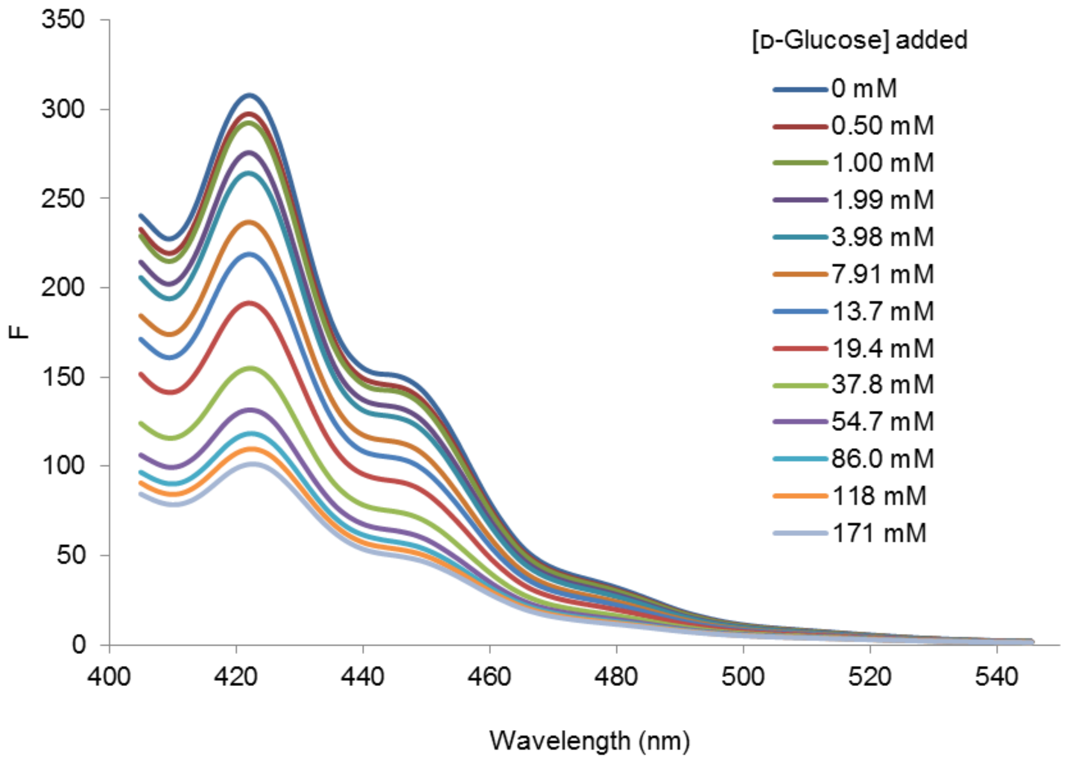

Figure S136. Fluorescence titration of receptor **6** (19 μM) with D-glucose **3** in phosphate buffer solution (pH = 7.1, 0.1 M) at 298 K. Excitation wavelength: 395nm.

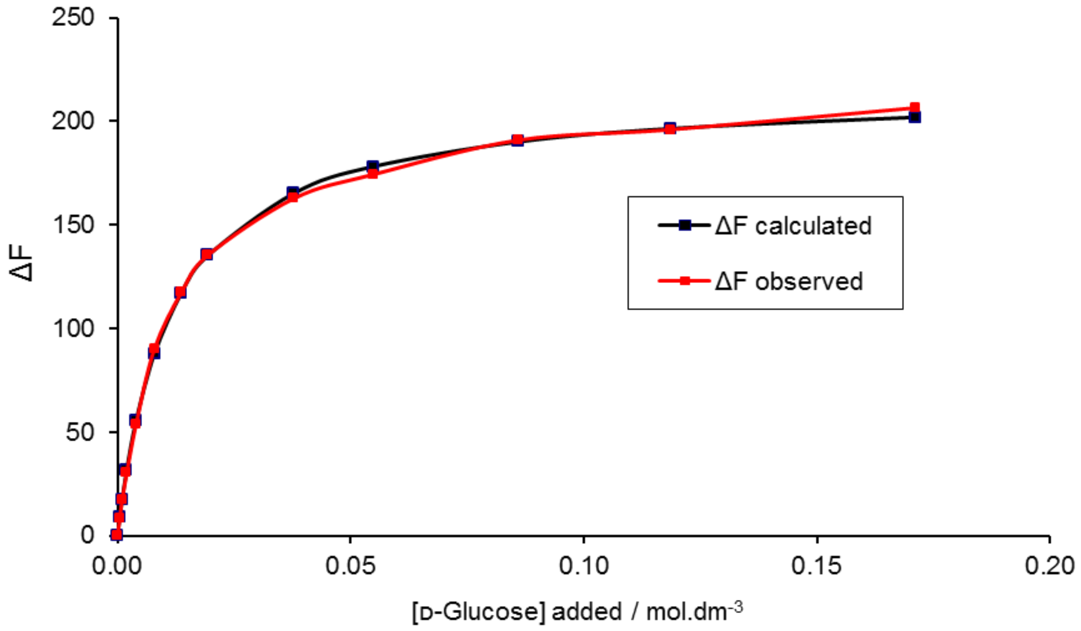

Figure S137. Experimental and calculated values for the fluorescence binding study of receptor **6** (19 μM) with D-glucose **3** in phosphate buffer solution (pH = 7.1, 0.1 M) at 298 K. Excitation: 395 nm. Emission observed at: 423 nm. K_a = 87 M^{-1} ± 5%. F/F^o = 3.13.

D-Glucosamine 13

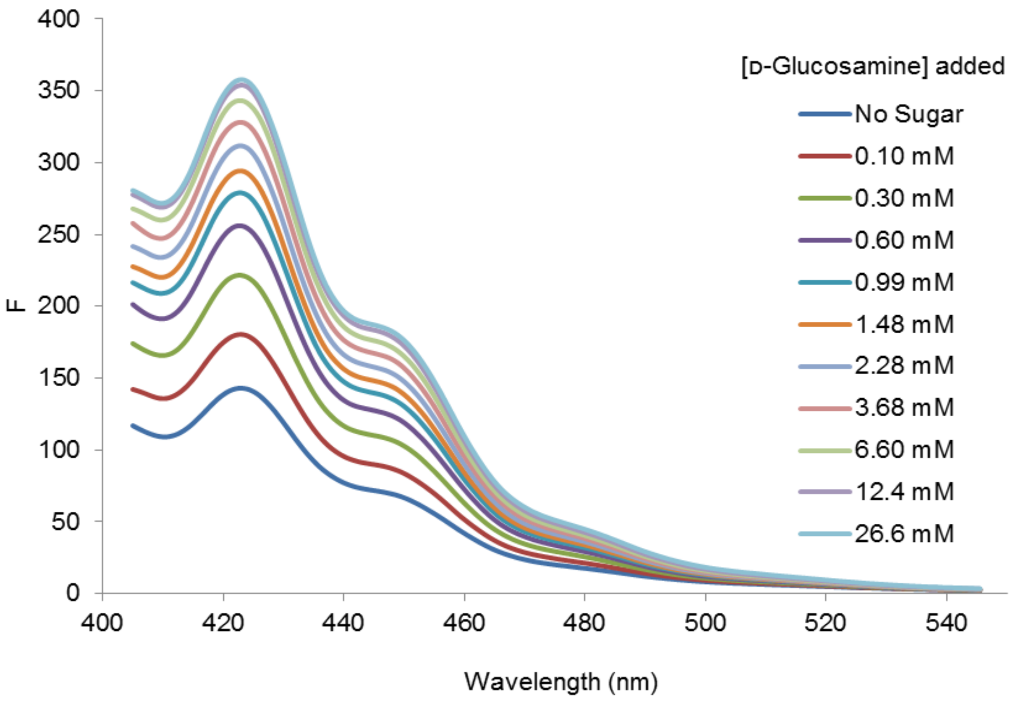

Figure S138. Fluorescence titration of receptor **6** (31.0 μM) with D-glucosamine **13** in H_2O (pH = 7.0) at 298 K. Excitation wavelength: 395 nm.

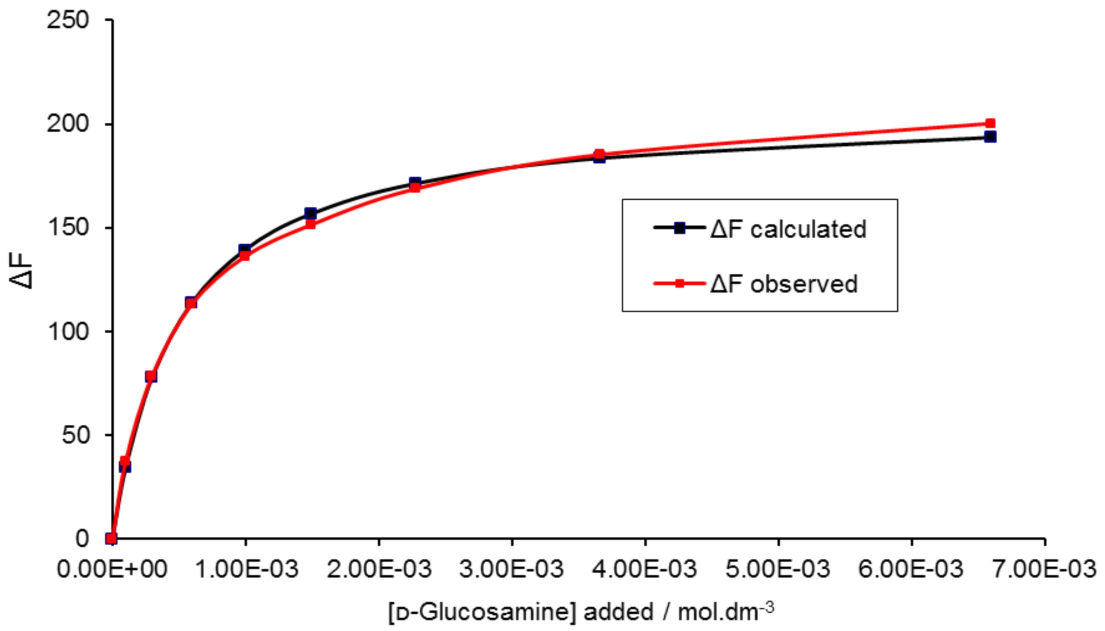

Figure S139. Experimental and calculated values for the fluorescence binding study of receptor **6** (31.0 μM) with D-glucosamine **13** in water (pH = 7.0) at 298 K. Excitation: 395 nm. Emission observed at: 423 nm. K_a = 2100 M^{-1} ± 8%. F/F^o = 2.45.

Receptor 7 Carbohydrate Binding Studies

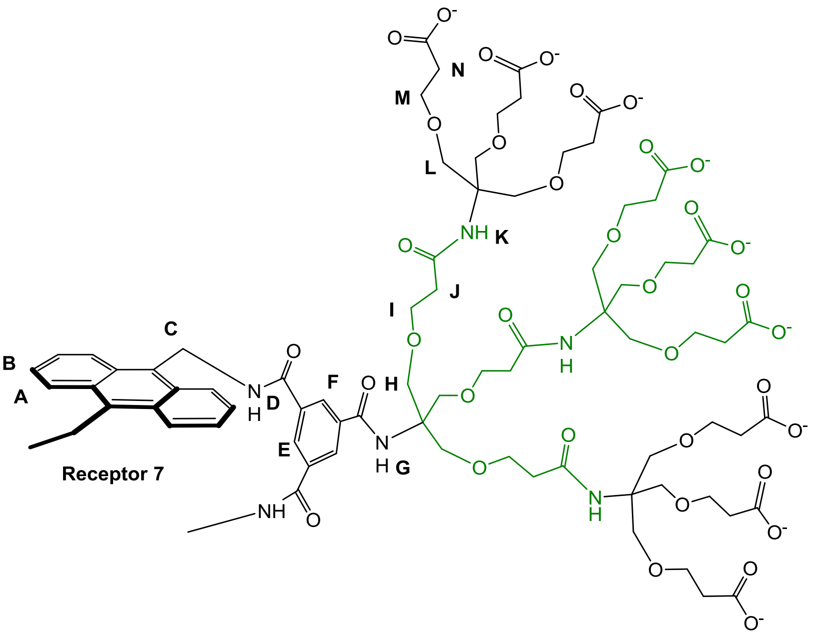

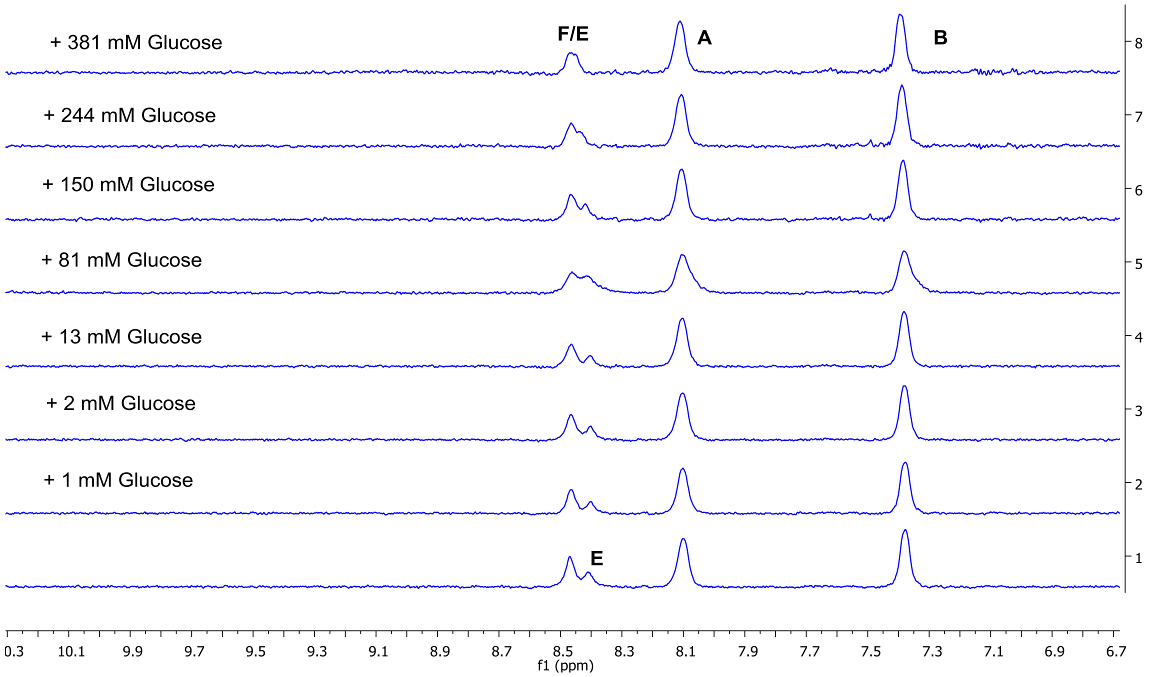

Figure S140. Partial ^{1}H NMR spectra from the titration of receptor **7** (0.32 mM) with D-glucose **3** in D_2O at 298 K.

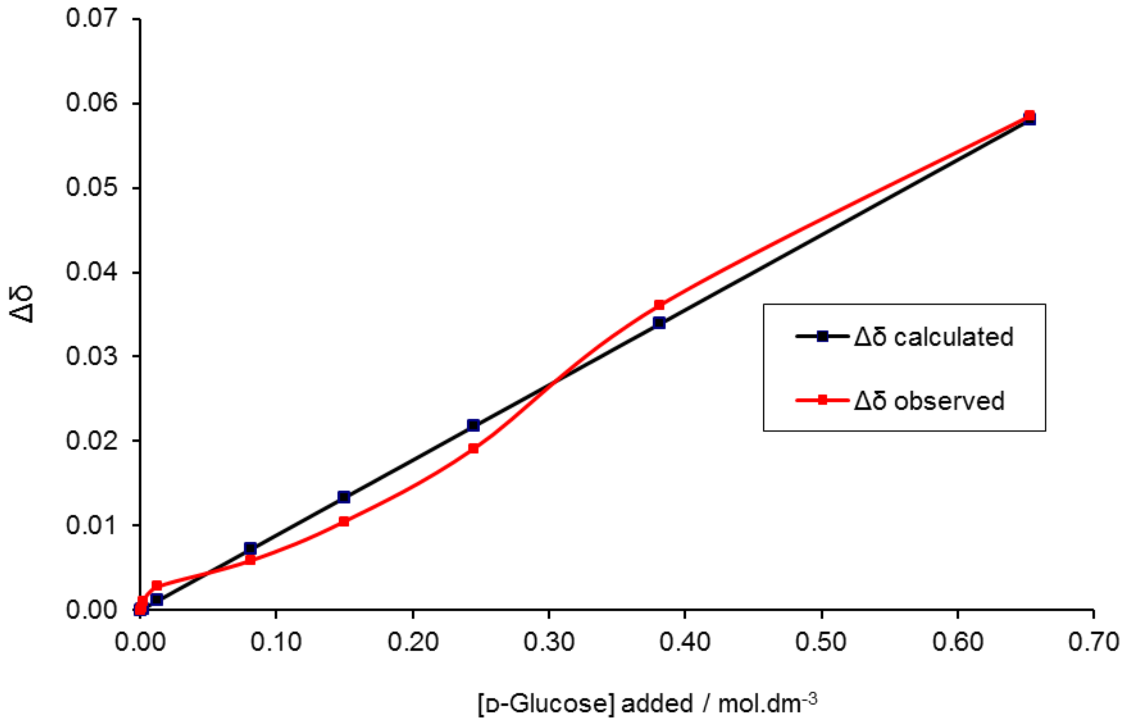

Figure S141. Experimental and calculated values for the fluorescence binding study of receptor **7** (0.32 mM) with D-glucose **3** in D_2O at 298 K. Proton E: K_a too small to be evaluated.

Receptor 8 Carbohydrate Binding Studies

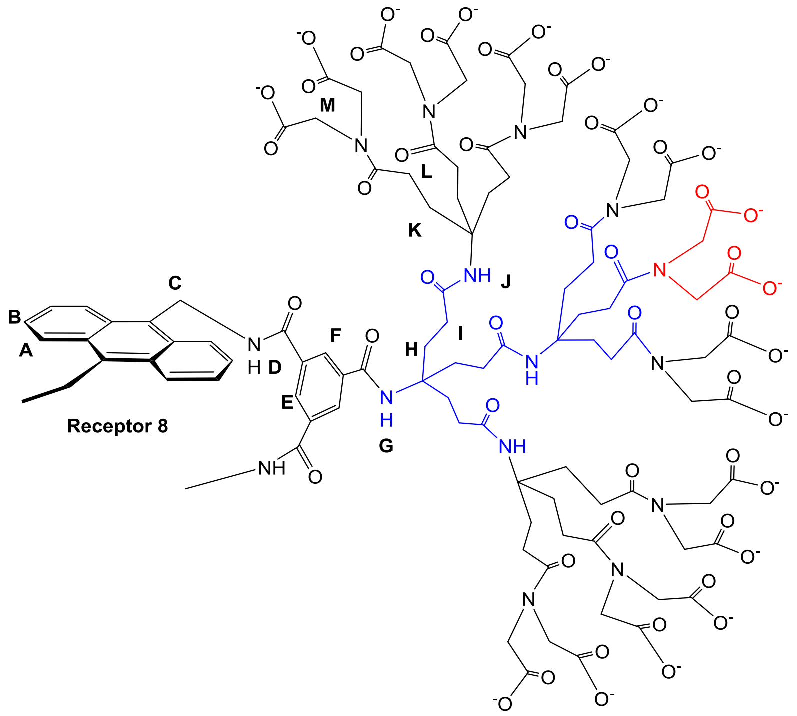

NMR spectra and binding analyses

D-Glucose 3

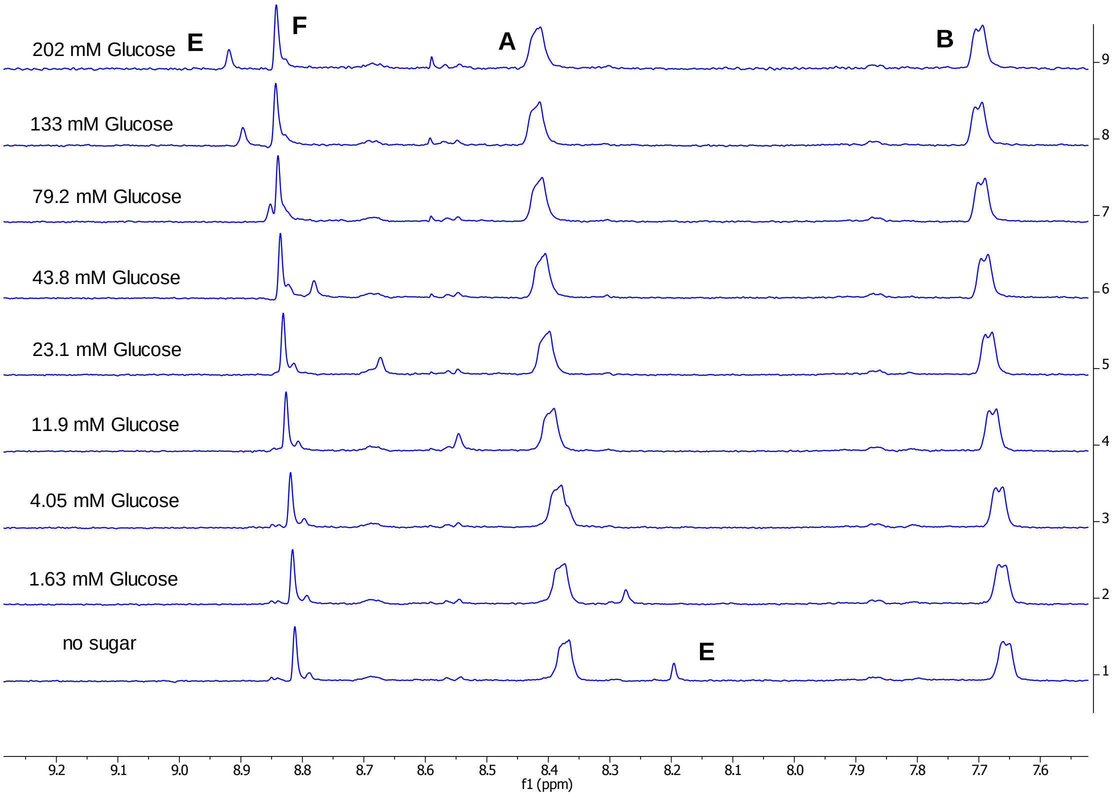

Figure S142. Partial ^{1}H NMR spectra from the titration of receptor **8** (0.09 mM) with D-glucose **3** in D_2O at 298 K.

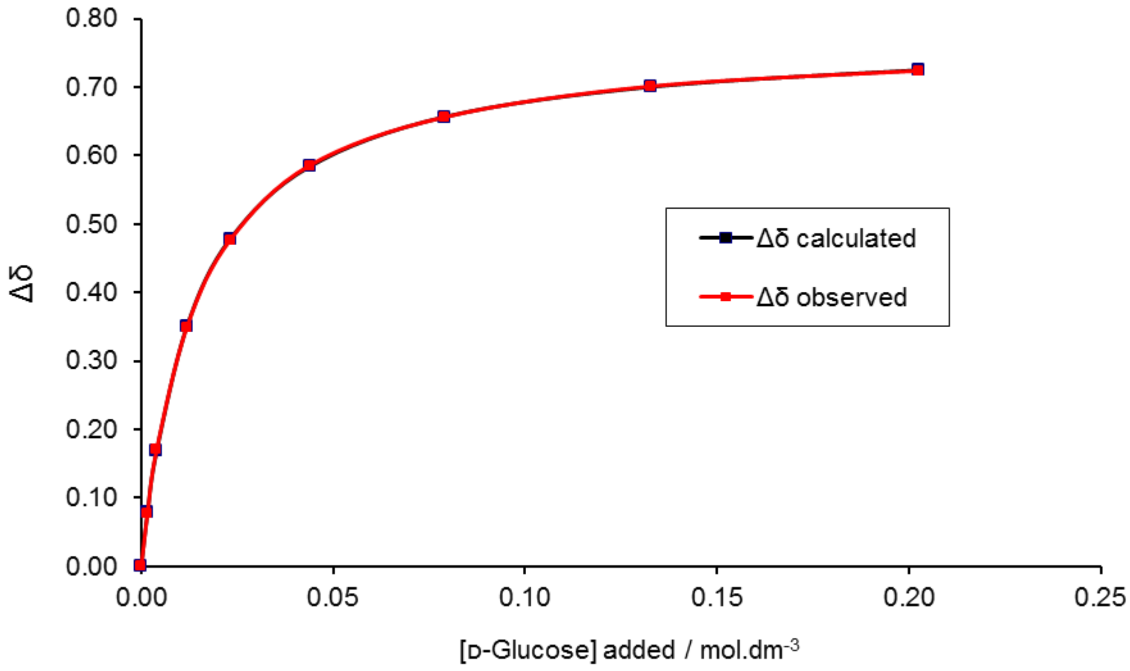

Figure S143. Experimental and calculated values for the NMR binding study of receptor **8** (0.09 mM) with D-glucose **3** in D_2O at 298 K. Proton E: K_a = 69 M^{-1} ± 1%, Δδ = 0.78.

Methyl β-ᴅ-glucoside

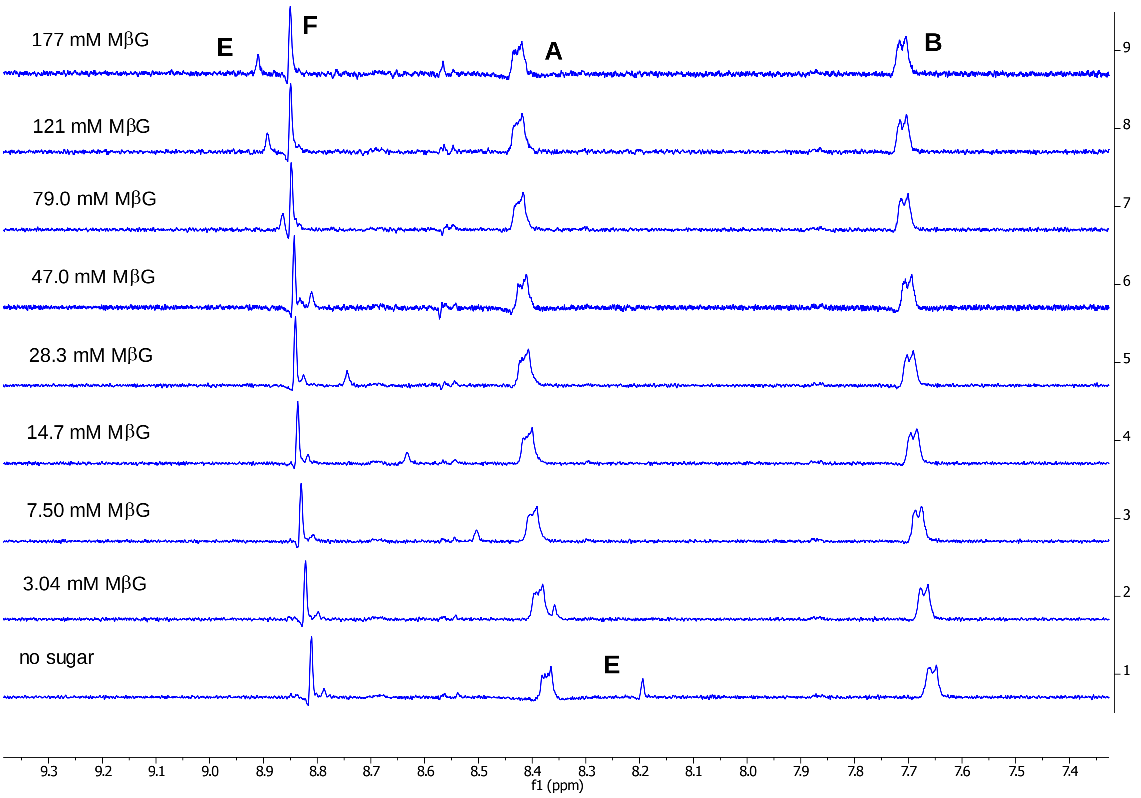

Figure S144. Partial ^{1}H NMR spectra from the titration of receptor **8** (0.10 mM) with methyl β-D-glucoside in D_2O at 298 K.

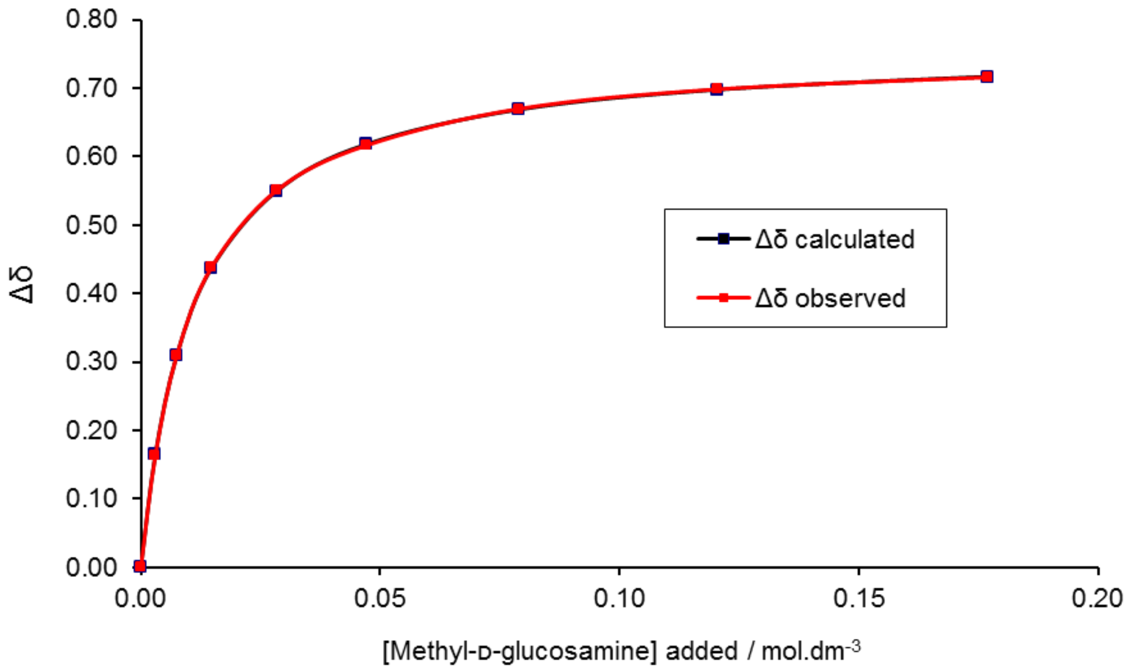

Figure S145. Experimental and calculated values for the NMR binding study receptor **8** (0.10 mM) with methyl β-D-glucoside in D_2O at 298 K. Proton E: K_a = 92 M^{-1} ± 1%, Δδ = 0.76.

N-Acetyl-D-glucosamine 15

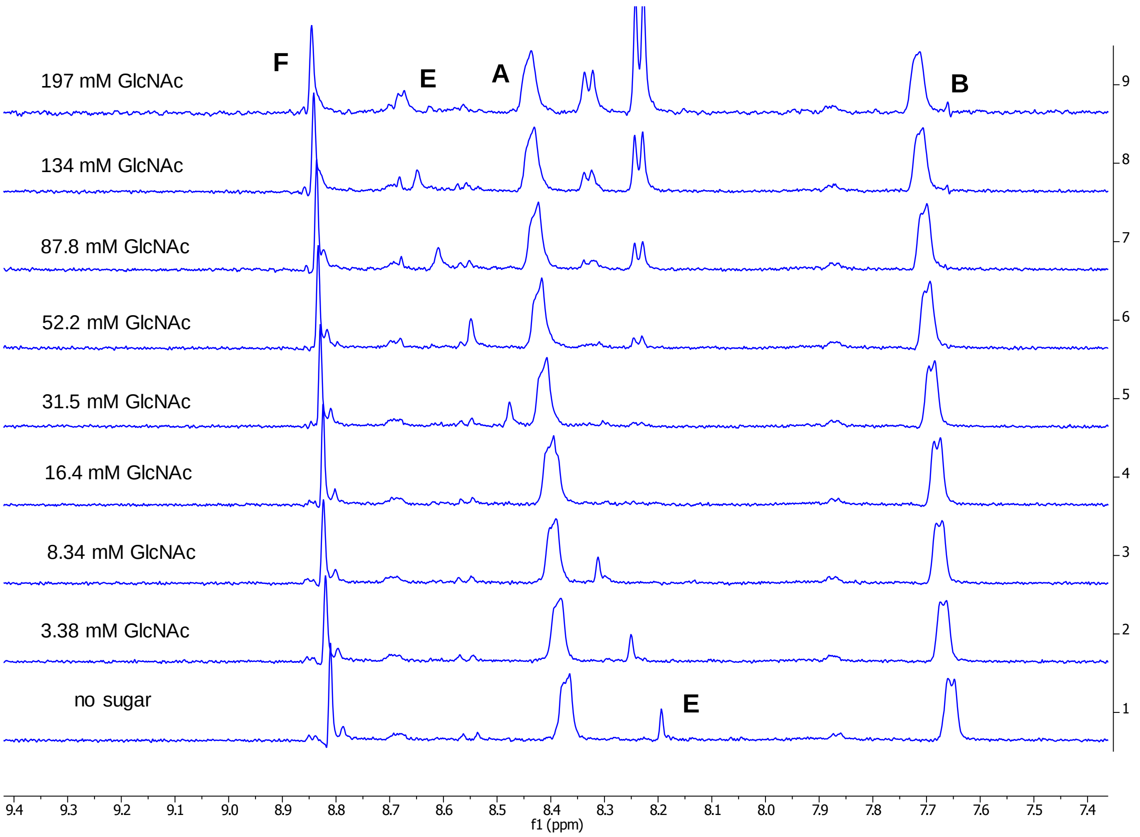

Figure S146. Partial ^{1}H NMR spectra from the titration of receptor **8** (0.10 mM) with GlcNAc **15** in D_2O at 298 K.

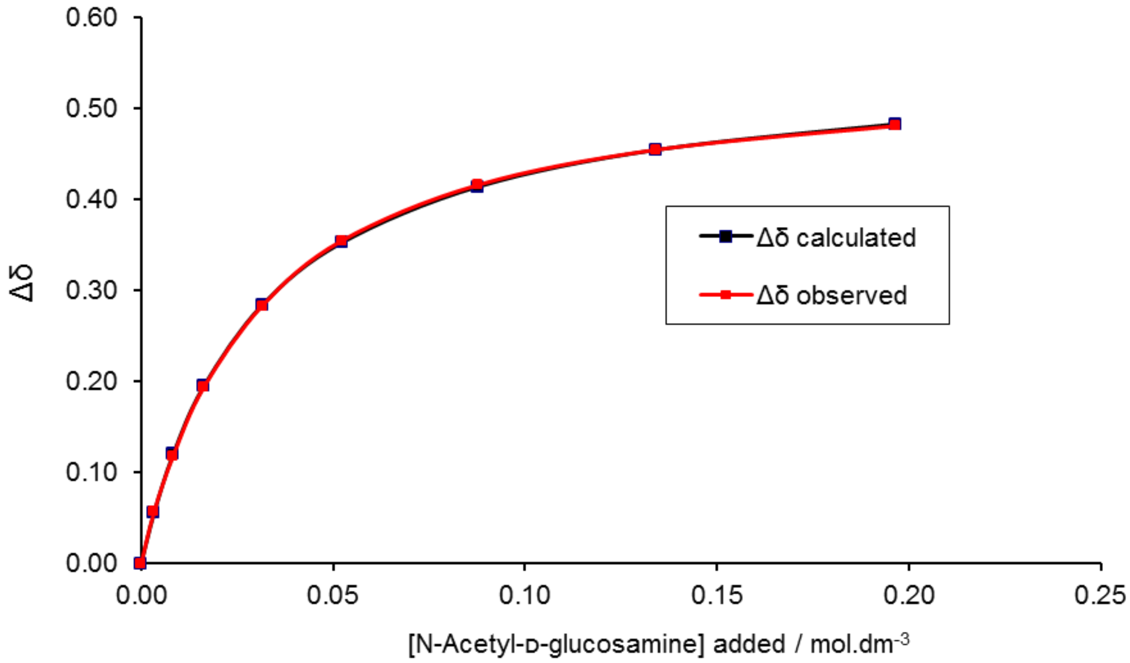

Figure S147. Experimental and calculated values for the NMR binding study of receptor **8** (0.10 mM) with GlcNAc **15** in D_2O at 298 K. Proton E: K_a = 33 M^{-1} ± 2%, Δδ = 0.56.

ᴅ-Galactose

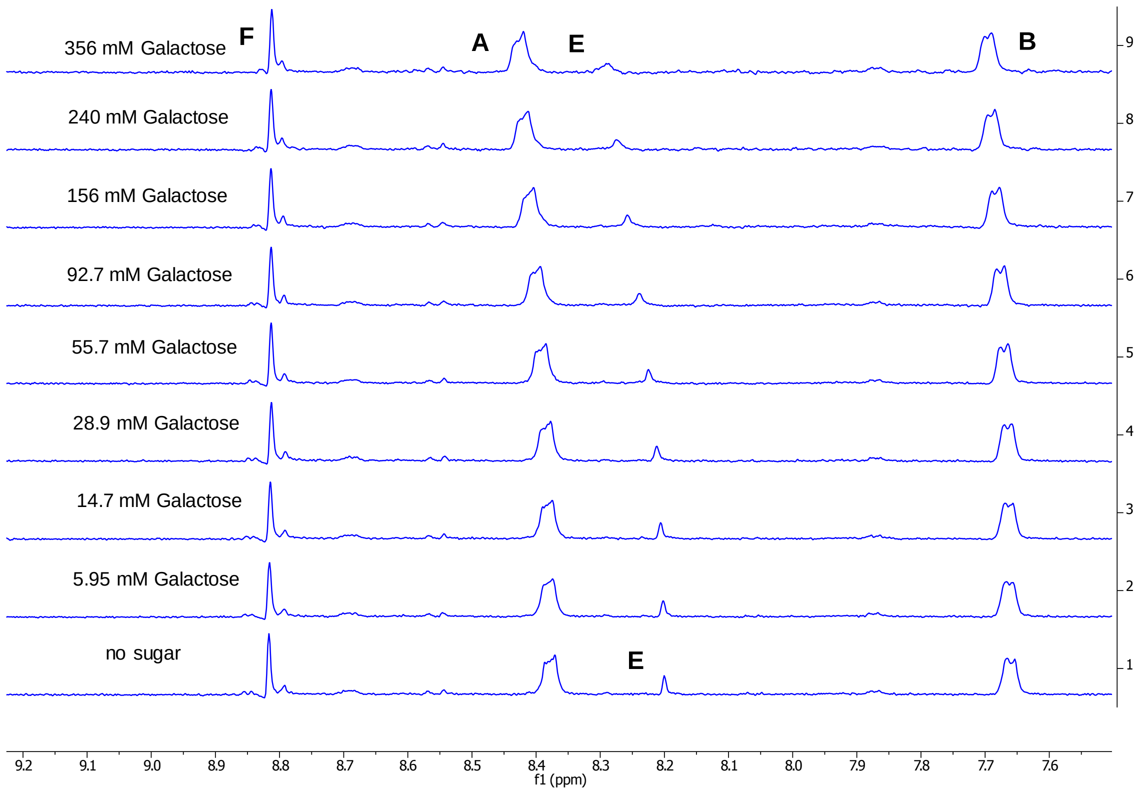

Figure S148. Partial ^{1}H NMR spectra from the titration of receptor **8** (0.10 mM) with D-galactose in D_2O at 298 K.

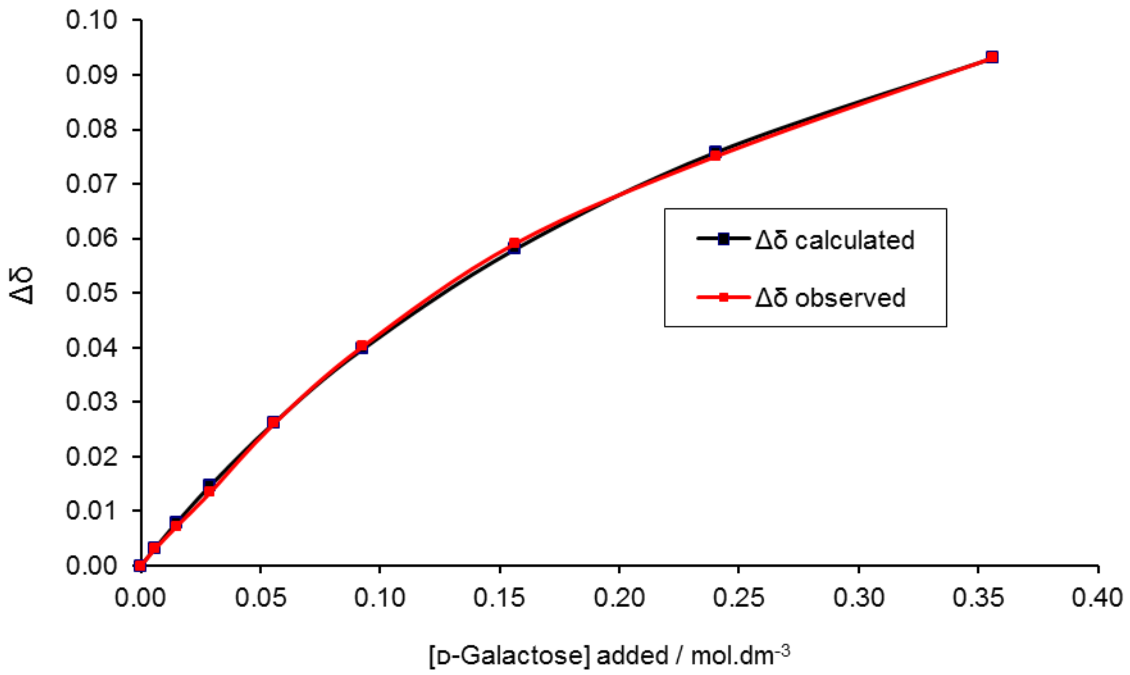

Figure S149. Experimental and calculated values for the NMR binding study of receptor **8** (0.10 mM) with D-galactose in D_2O at 298 K. Proton E: K_a = 3 M^{-1} ± 4%, Δδ = 0.18.

D-Mannose

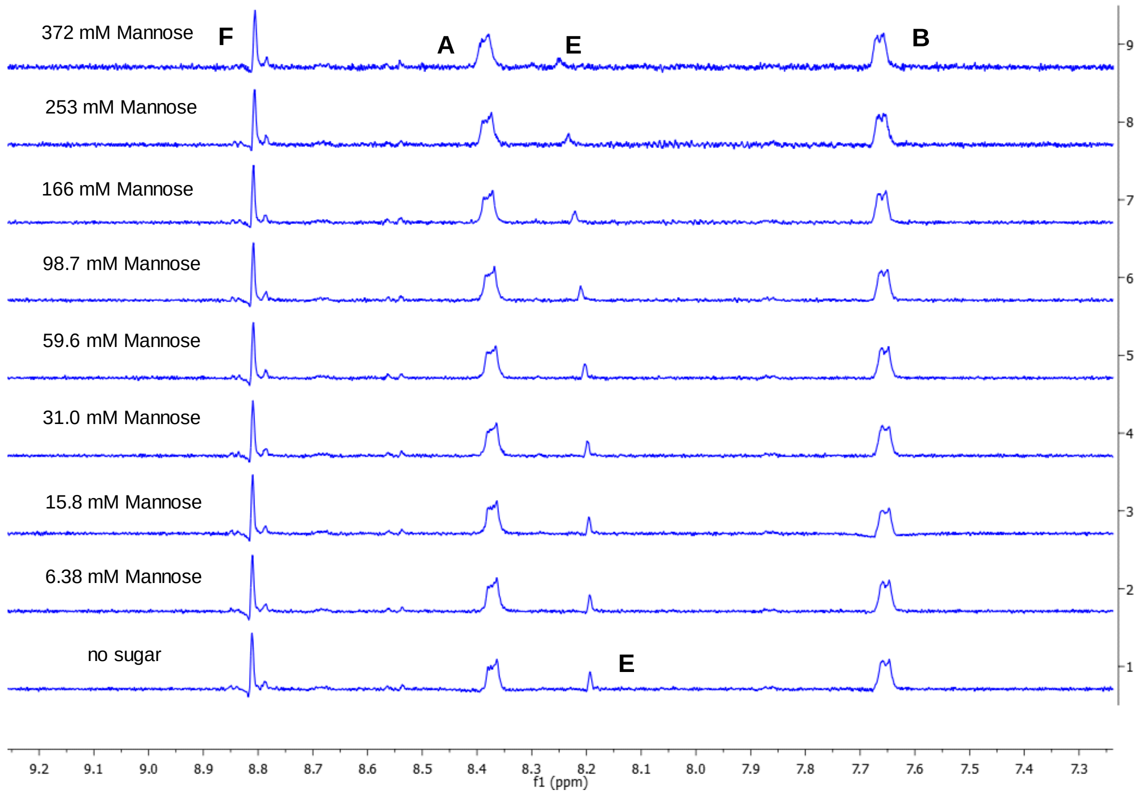

Figure S150. Partial ^{1}H NMR spectra from the titration of receptor **8** (0.10 mM) with D-mannose in D_2O at 298 K.

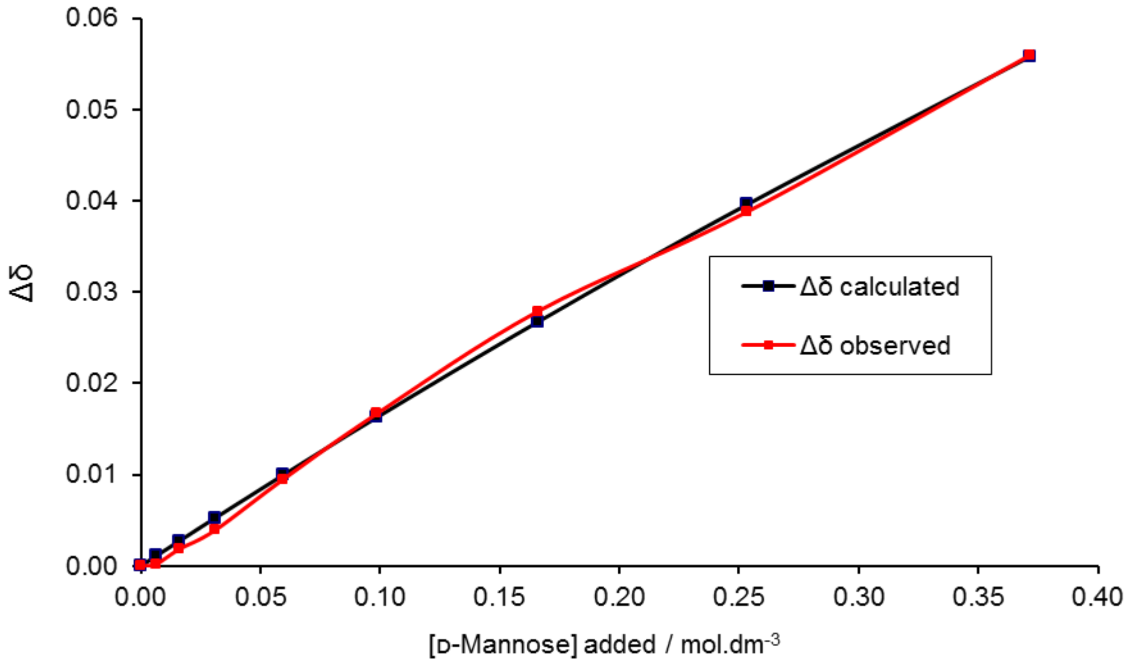

Figure S151. Experimental and calculated values for the NMR binding study of receptor **8** (0.10 mM) with mannose in D_2O at 298 K. Proton E: K_a too small to be evaluated with reasonable accuracy.

D-Maltose

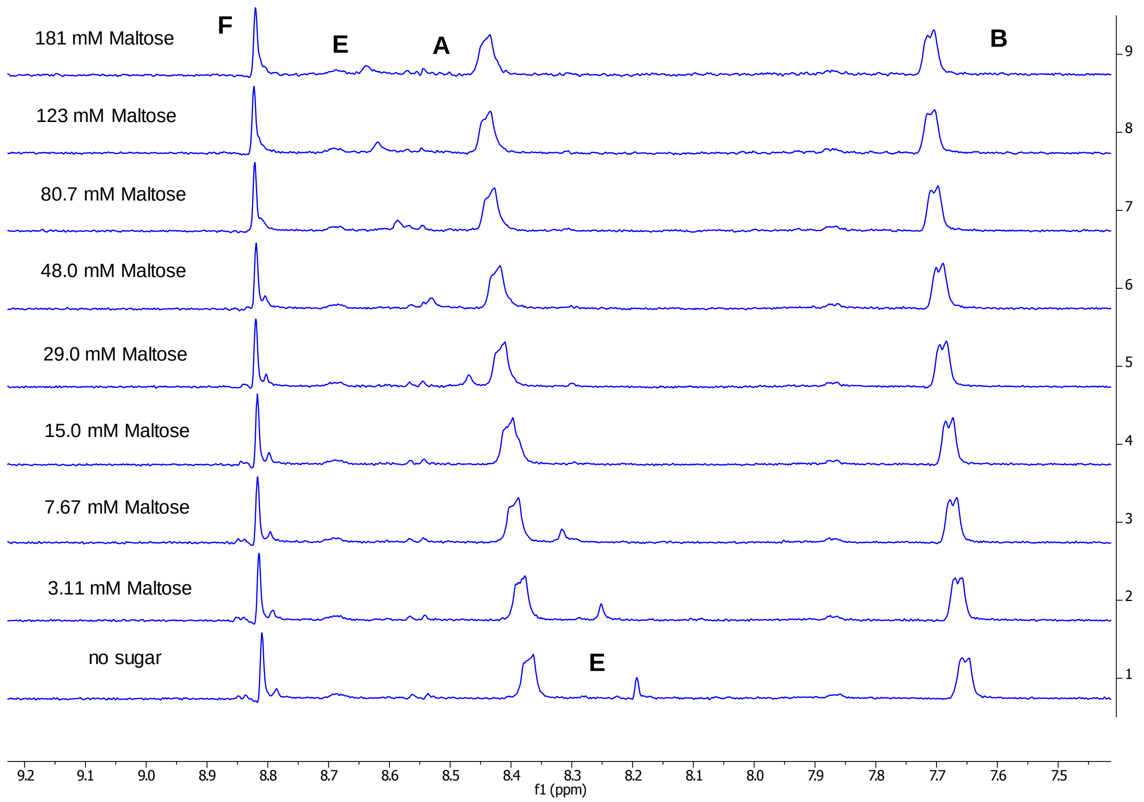

Figure S152. Partial ^{1}H NMR spectra from the titration of receptor **8** (0.10 mM) with D-maltose in D_2O at 298 K.

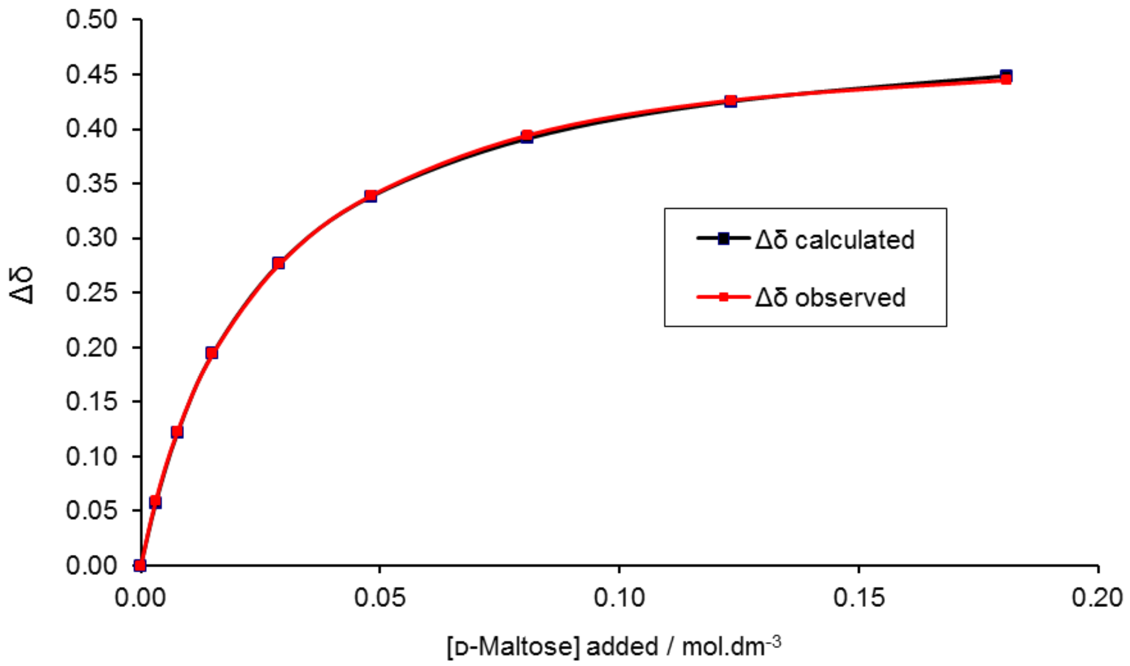

Figure S153. Experimental and calculated values for the NMR binding study of receptor **8** (98 µM) with D-maltose in D_2O at 298 K. Proton E: K_a = 41 M^{-1} ± 1%, Δδ = 0.51.

D-Cellobiose

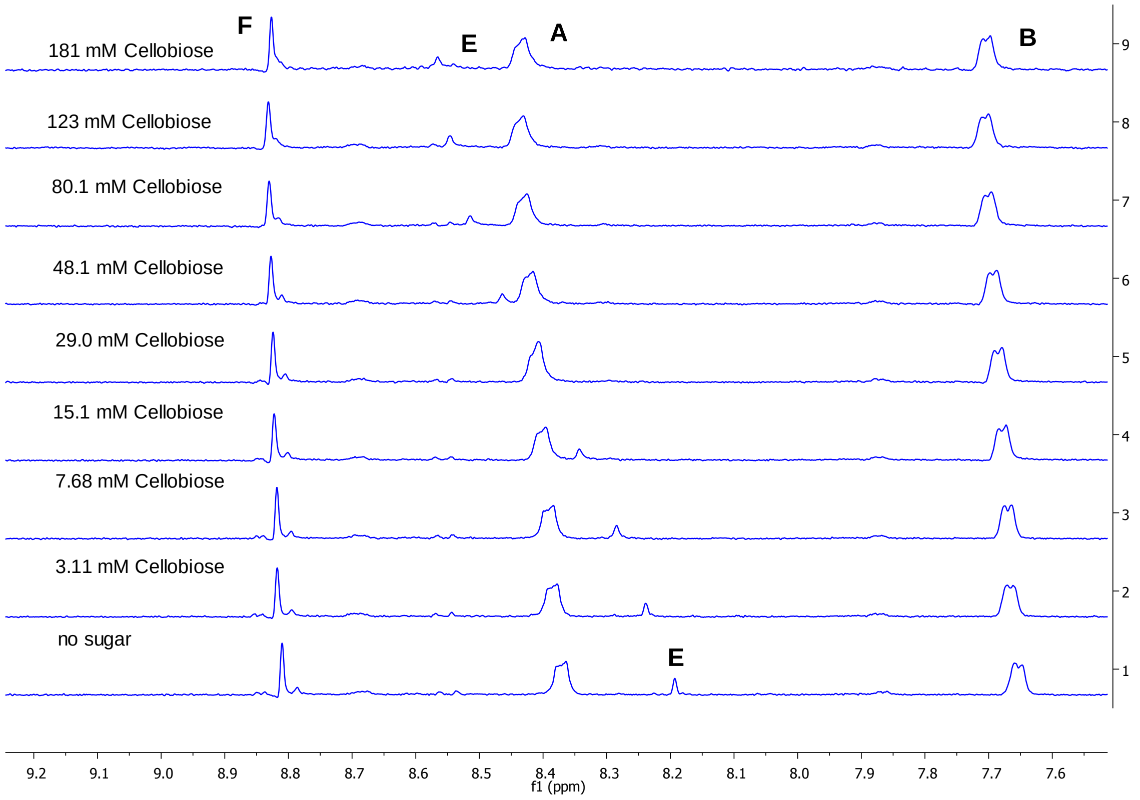

Figure S154. Partial ^{1}H NMR spectra from the titration of receptor **8** (0.10 mM) with D-cellobiose in D_2O at 298 K.

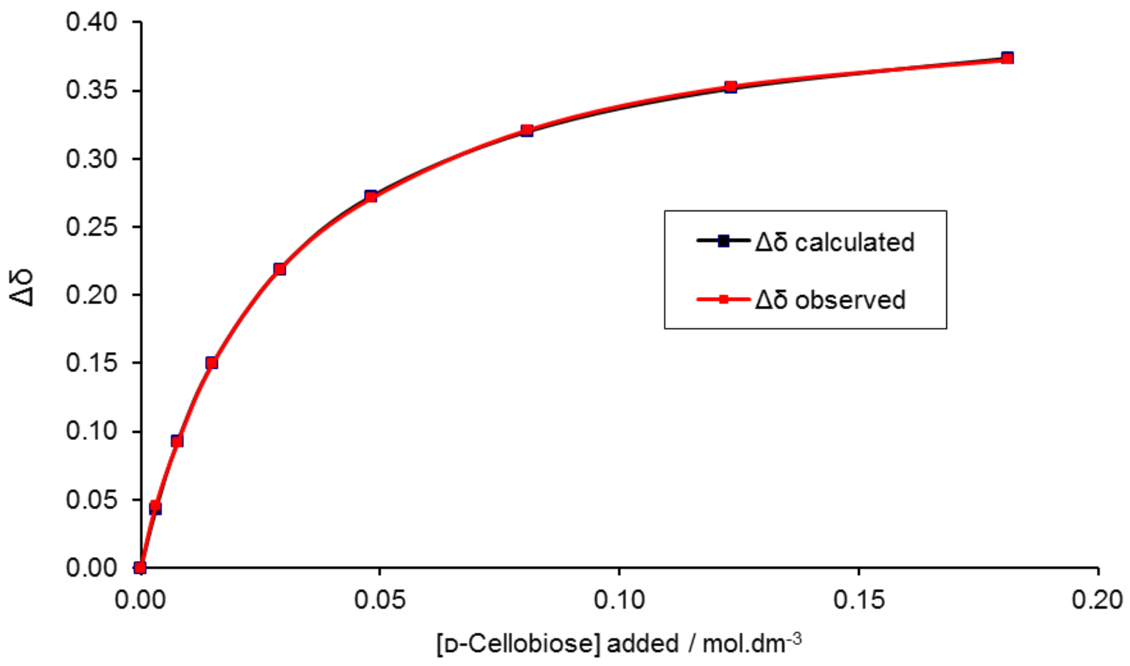

Figure S155. Experimental and calculated values for the NMR binding study of receptor **8** (98 µM) with D-cellobiose in D_2O at 298 K. Proton E: K_a = 35 M^{-1} ± 3%, Δδ = 0.43.

D-Lactose

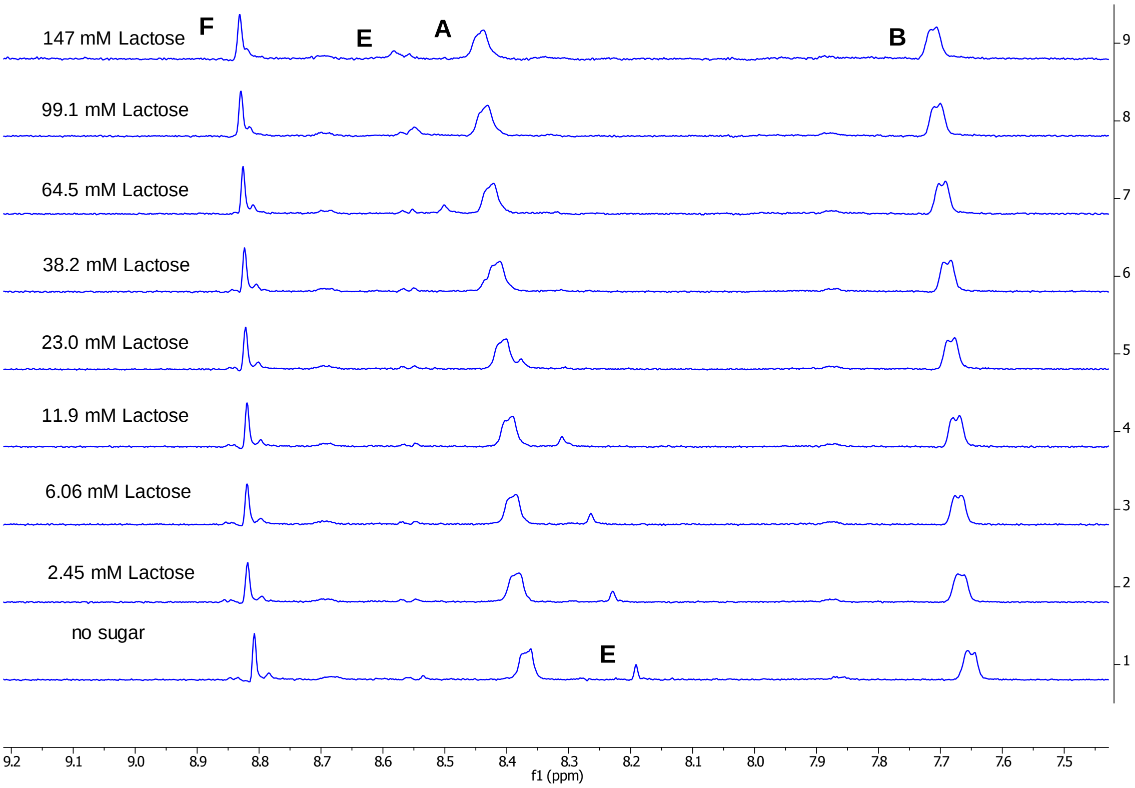

Figure S156. Partial ^{1}H NMR spectra from the titration of receptor **8** (0.10 mM) with D-lactose in D_2O at 298 K.

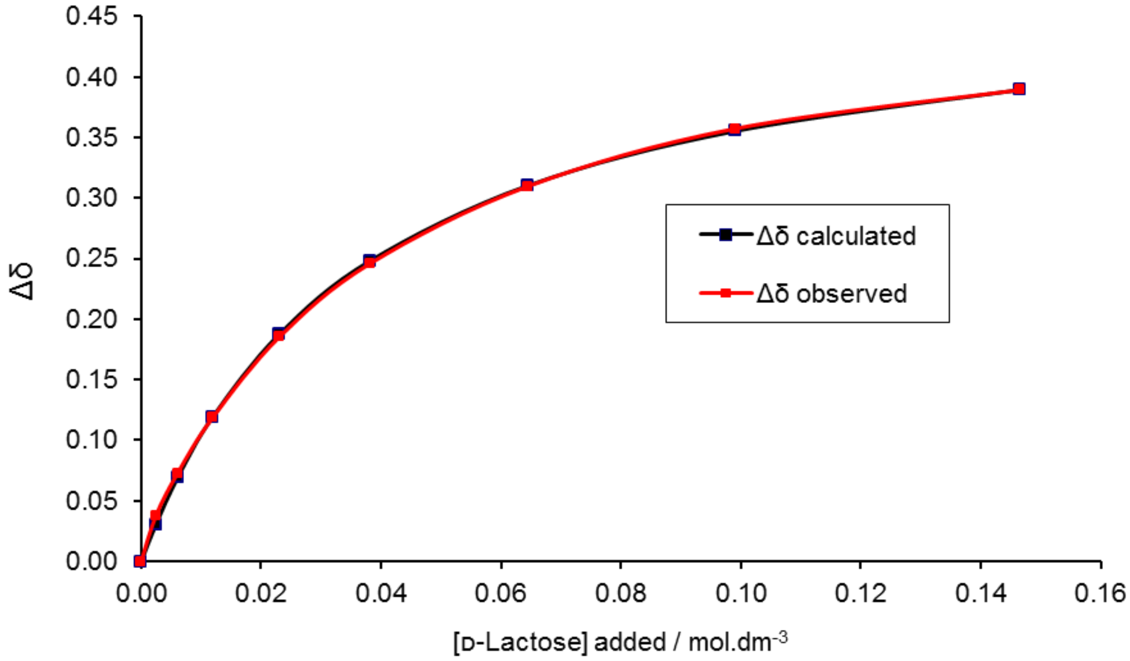

Figure S157. Experimental and calculated values for the NMR binding study receptor **8** (0.10 mM) with D-lactose in D_2O at 298 K. Proton E: K_a = 27 M^{-1} ± 9%, $\Delta\delta$ = 0.48.

D-Glucosamine 13

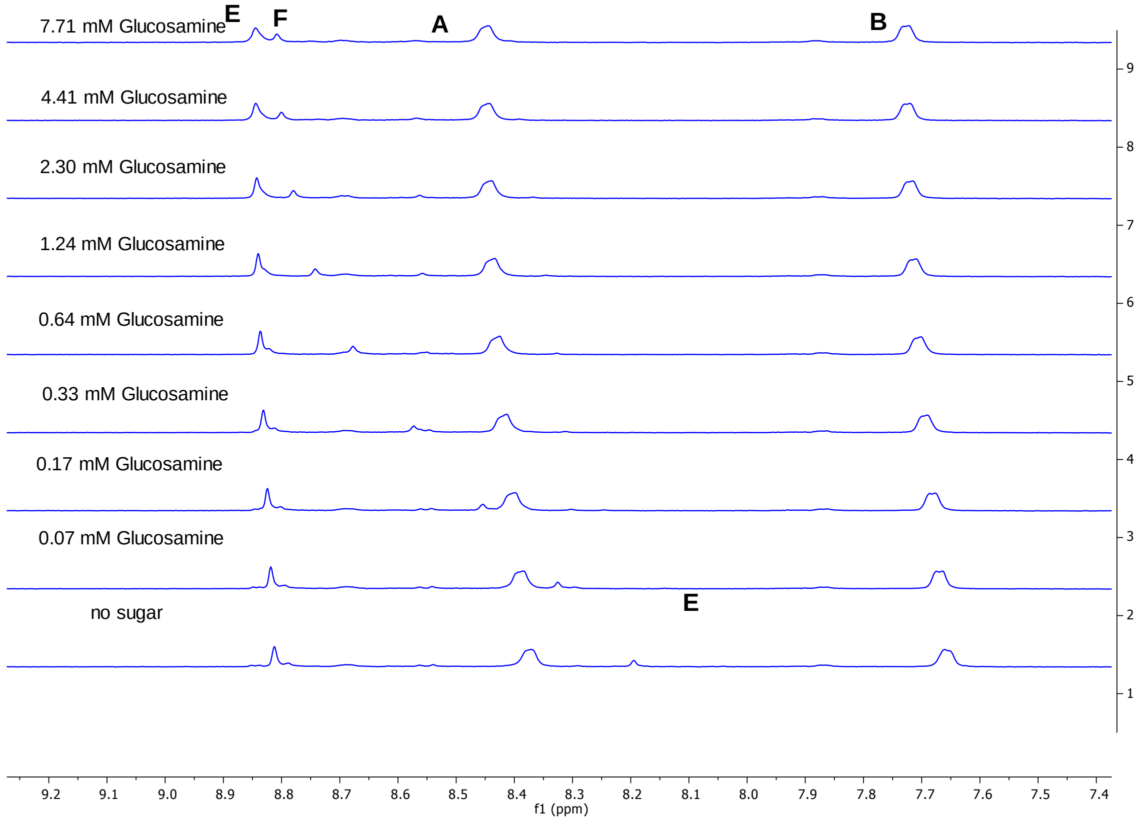

Figure S158. Partial ^1H NMR spectra from the titration of receptor **8** (0.10 mM) with D-glucosamine **13** in D_2O (pH = 7) at 298 K.

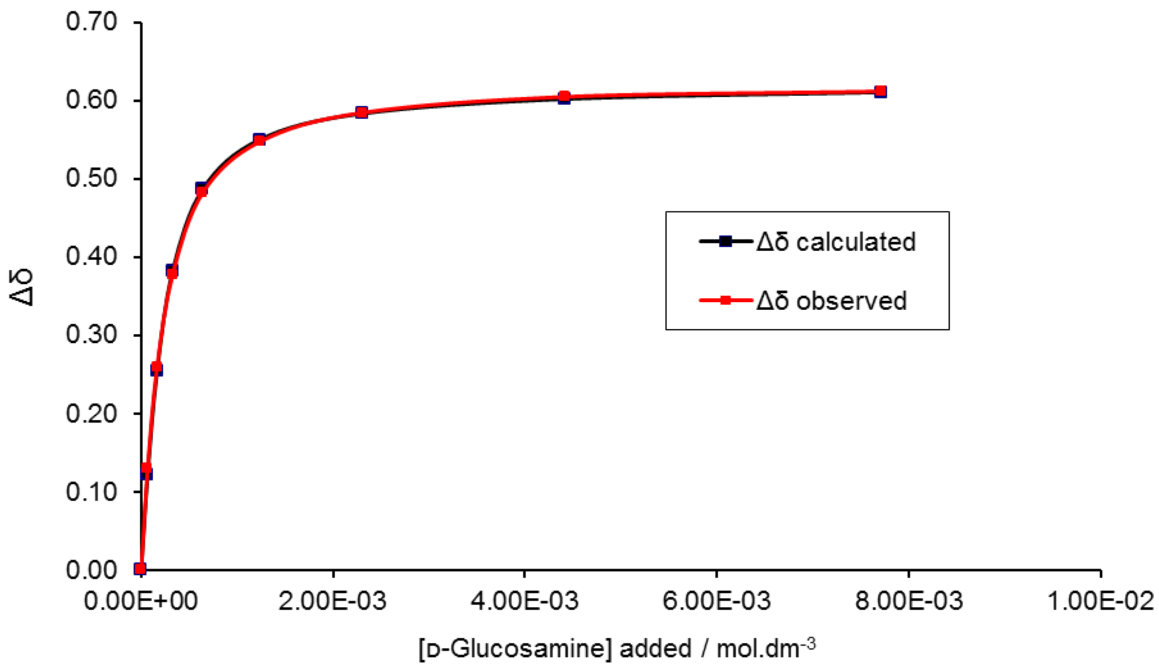

Figure S159. Experimental and calculated values for the NMR binding study of receptor **8** (0.10 mM) with D-glucosamine **13** in D_2O at 298 K. Proton E: K_a = 7000 M^{-1} ± 8%, Δδ = 0.62.

D-Glucosamine 13 (NaCl 20 mM)

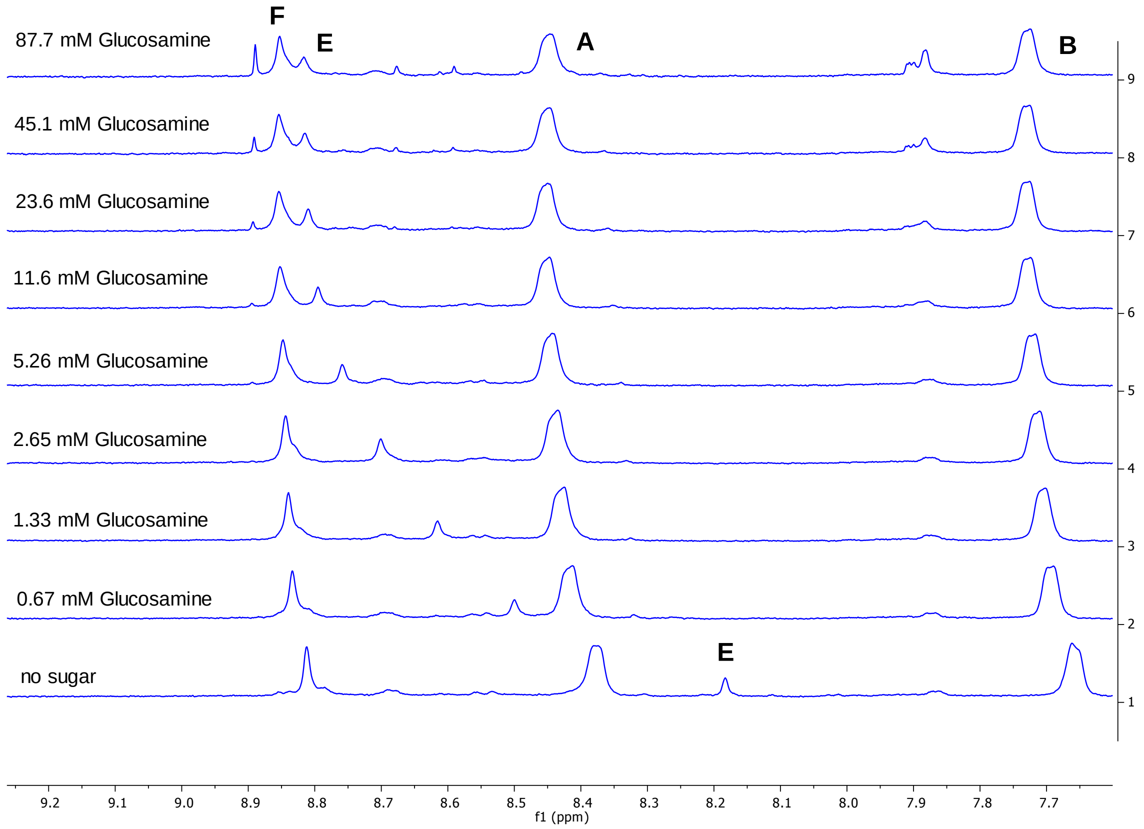

Figure S160. Partial ^{1}H NMR spectra from the titration of receptor **8** (0.14 mM) with D-glucosamine **13** in D_2O (pH = 7, 20 mM, NaCl) at 298 K.

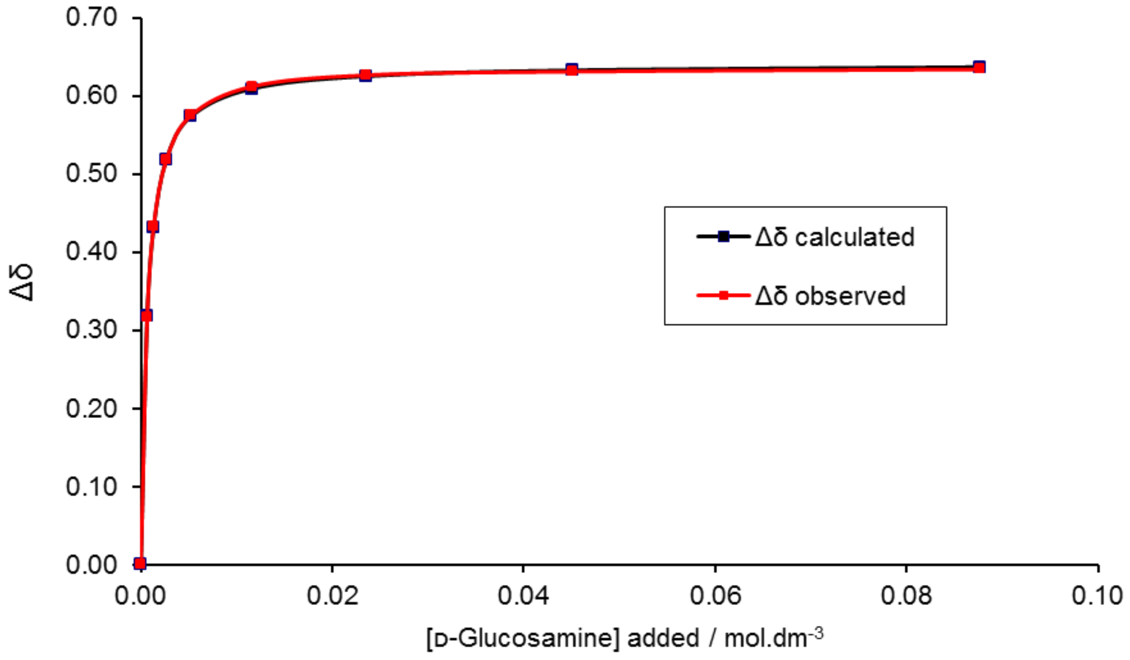

Figure S161. Experimental and calculated values for the NMR binding study of receptor **8** (0.14 mM) with D-glucosamine **13** in D_2O (pH = 7, 20 mM, NaCl) at 298 K. Proton E: K_a = 1660 M^{-1} ± 16%, Δδ = 0.64.

D-Glucosamine 13 (NaCl 154 mM)

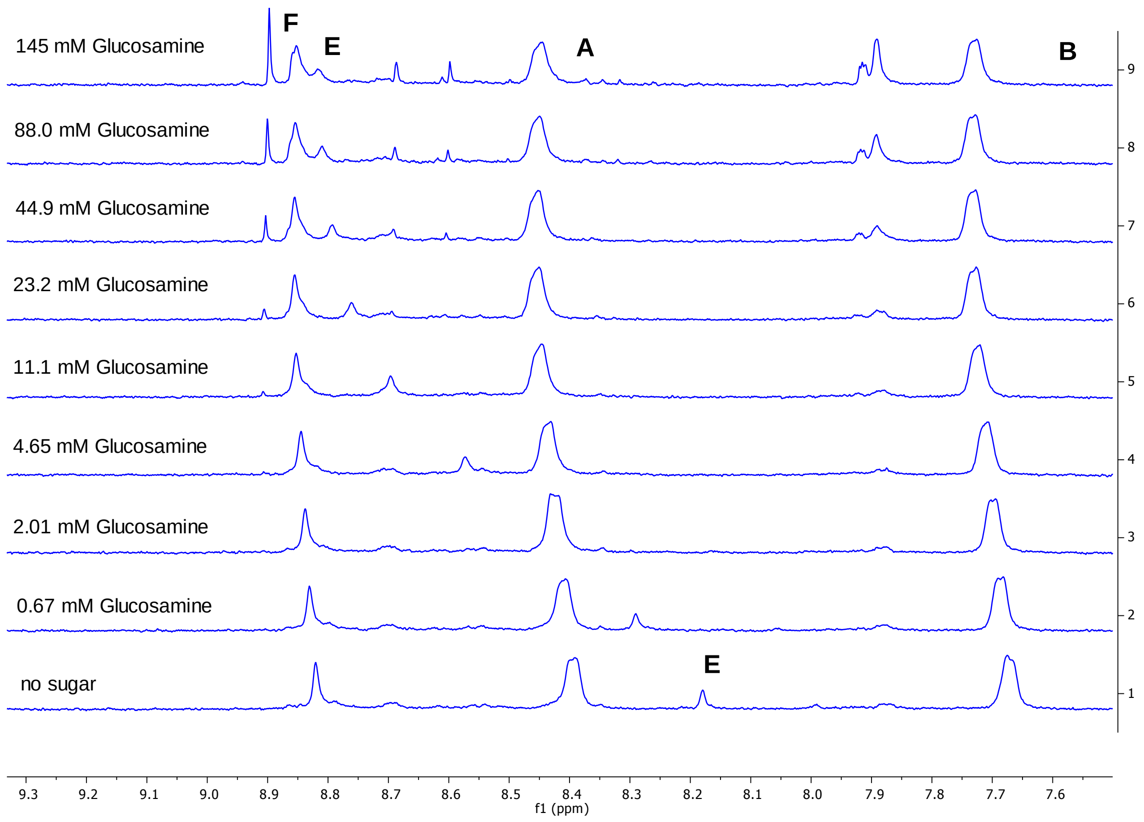

Figure S162. Partial ^{1}H NMR spectra from the titration of receptor **8** (0.14 mM) with D-glucosamine **13** in D_2O (pH = 7, 154 mM, NaCl) at 298 K.

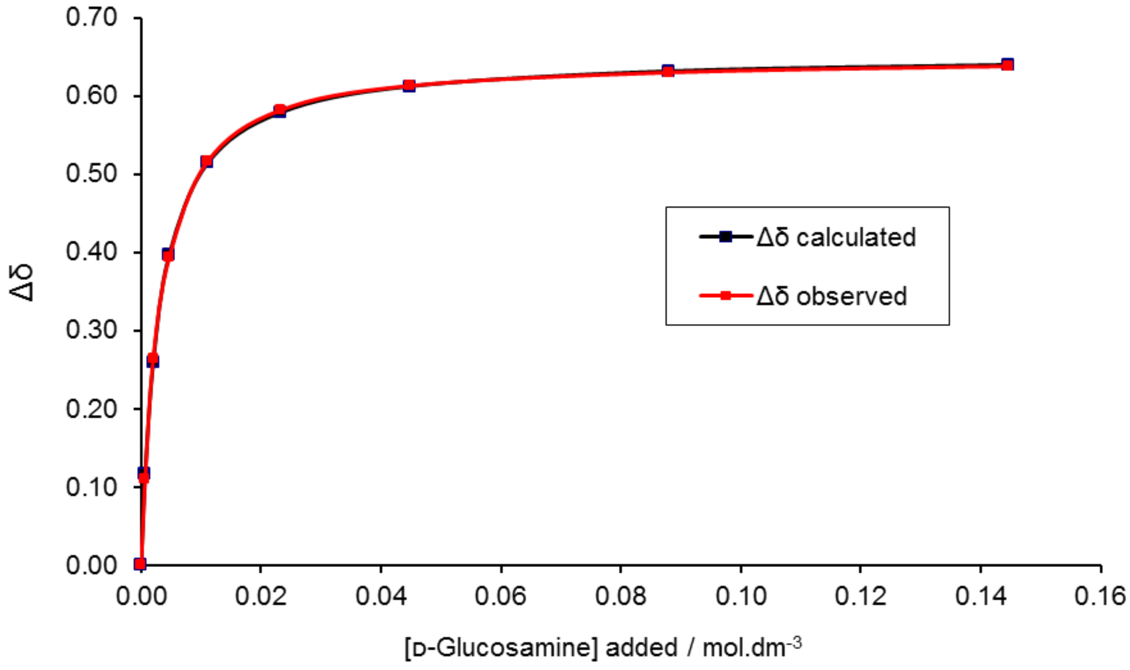

Figure S163. Experimental and calculated values for the NMR binding study of receptor **8** (0.14 mM) with D-glucosamine **13** in D_2O (pH = 7, 154 mM, NaCl) at 298 K. Proton E: K_a = 340 M^{-1} ± 6%, Δδ = 0.65.

D-Galactosamine (NaCl 154 mM)

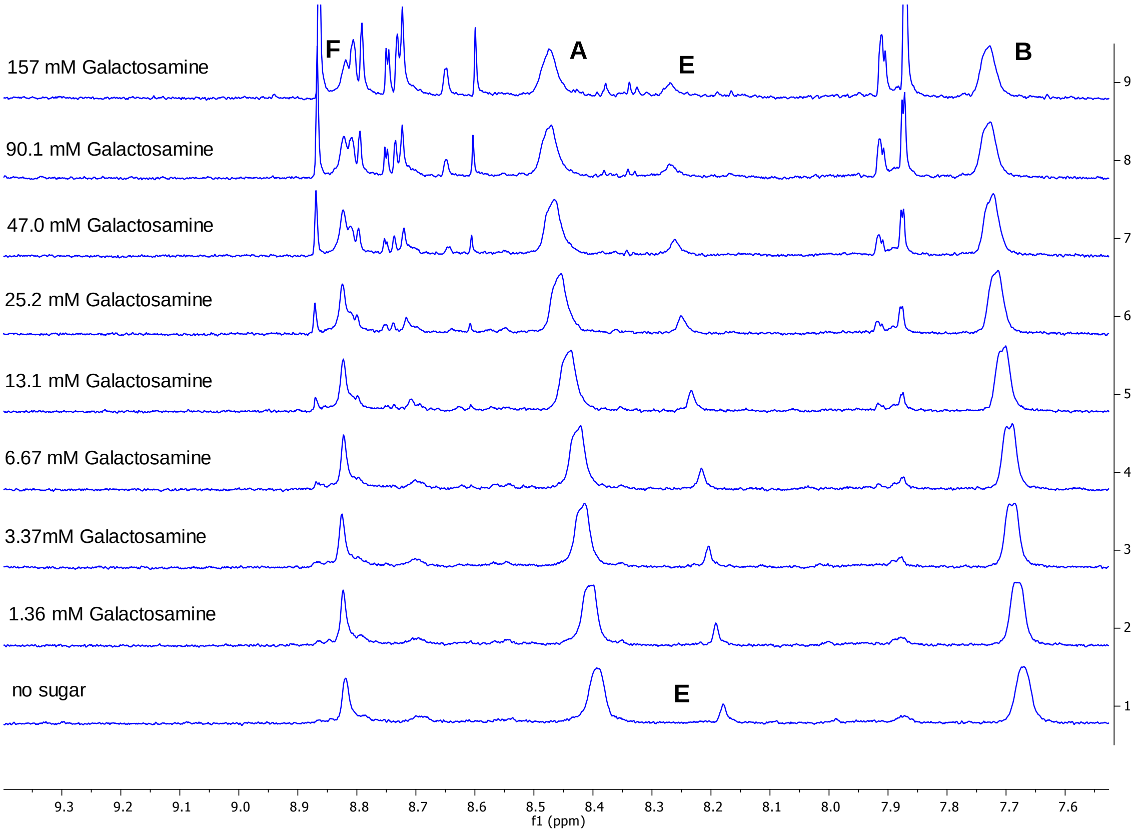

Figure S164. Partial ^{1}H NMR spectra from the titration of receptor **8** (0.14 mM) with D-galactosamine in D_2O (pH = 7, 154 mM, NaCl) at 298 K.

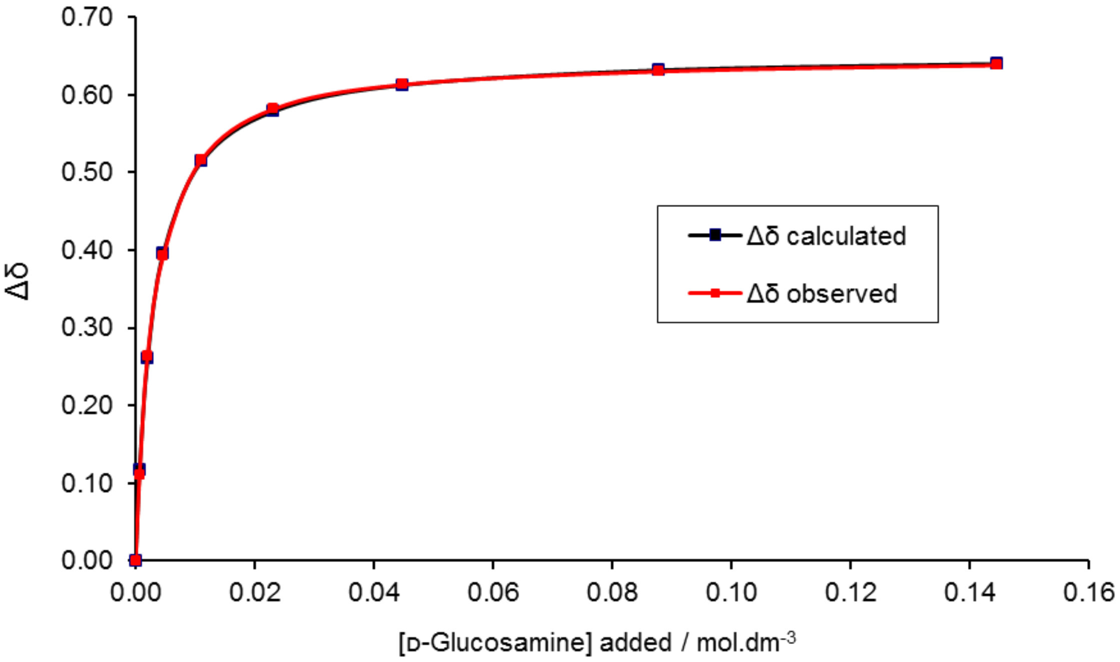

Figure S165. Experimental and calculated values for the NMR binding study of receptor **8** (0.14 mM) with D-galatosamine **13** in D_2O (pH = 7, 154 mM, NaCl) at 298 K. Proton E: K_a = 98 M^{-1} ± 10%, Δδ = 0.01.

Fluorescence Titrations

D-Glucose 3

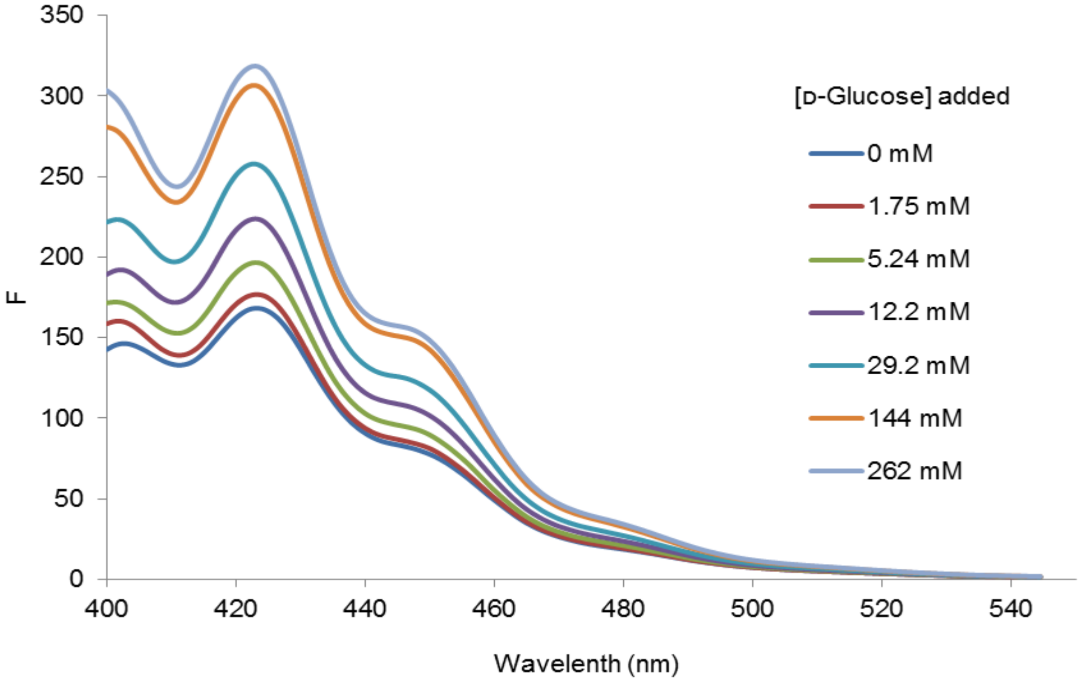

Figure S166. Fluorescence titration of receptor **8** (15.6 μM) with D-glucose **3** in phosphate buffer solution (pH = 7.1, 0.1 M) at 298 K. Excitation wavelength: 395 nm.

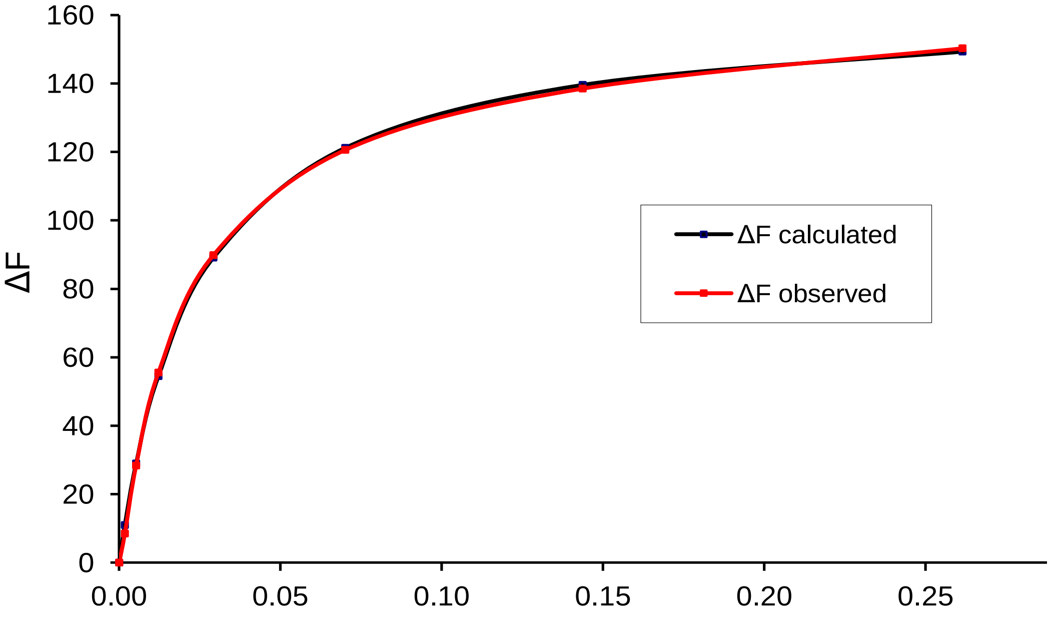

Figure S167. Experimental and calculated values for the fluorescence binding study of receptor **8** (15.6 μM) with D-glucose **3** in phosphate buffer solution (pH = 7.1, 0.1 M) at 298 K. Excitation: 395 nm. Emission observed at: 423 nm. K_a = 41M^{-1} ± 9.3 %. F/F^{o} = 2.03.

D-Glucosamine 13

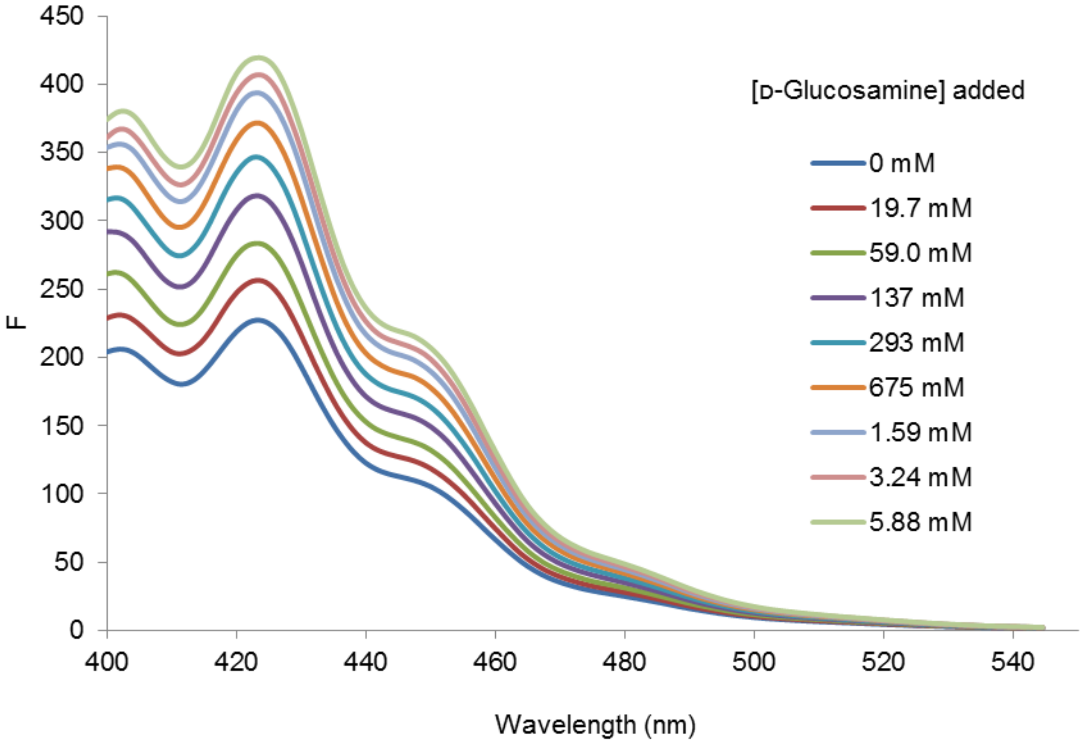

Figure S168. Fluorescence titration of receptor **8** (12.0 μM) with D-glucosamine **13** in H_2O (pH = 7.0) at 298 K. Excitation wavelength: 395 nm.

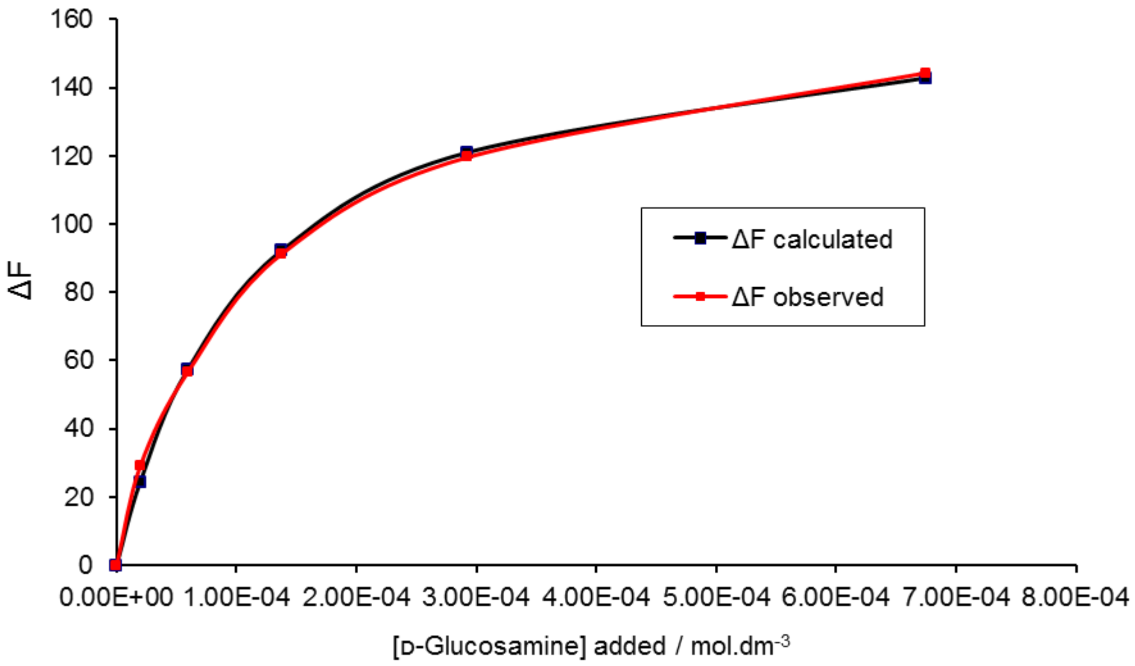

Figure S169 Experimental and calculated values for the fluorescence binding study of receptor **8** (12.0 μM) with D-glucosamine **13** in water (pH = 7.0) at 298 K. Excitation: 395 nm. Emission observed at: 423 nm. K_a = 9700 M^{-1} ± 11%. F/F^o = 1.72.

Receptor 9 Carbohydrate Binding Studies

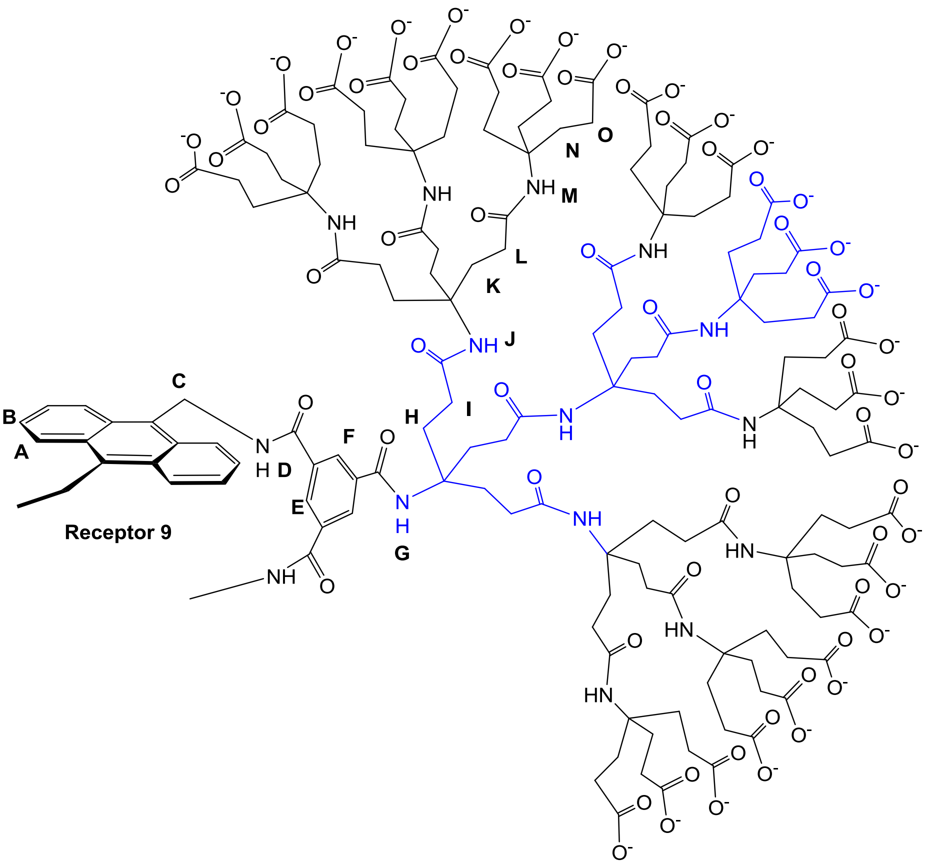

Receptor 9

NMR spectra and binding analyses

D-Glucose 3

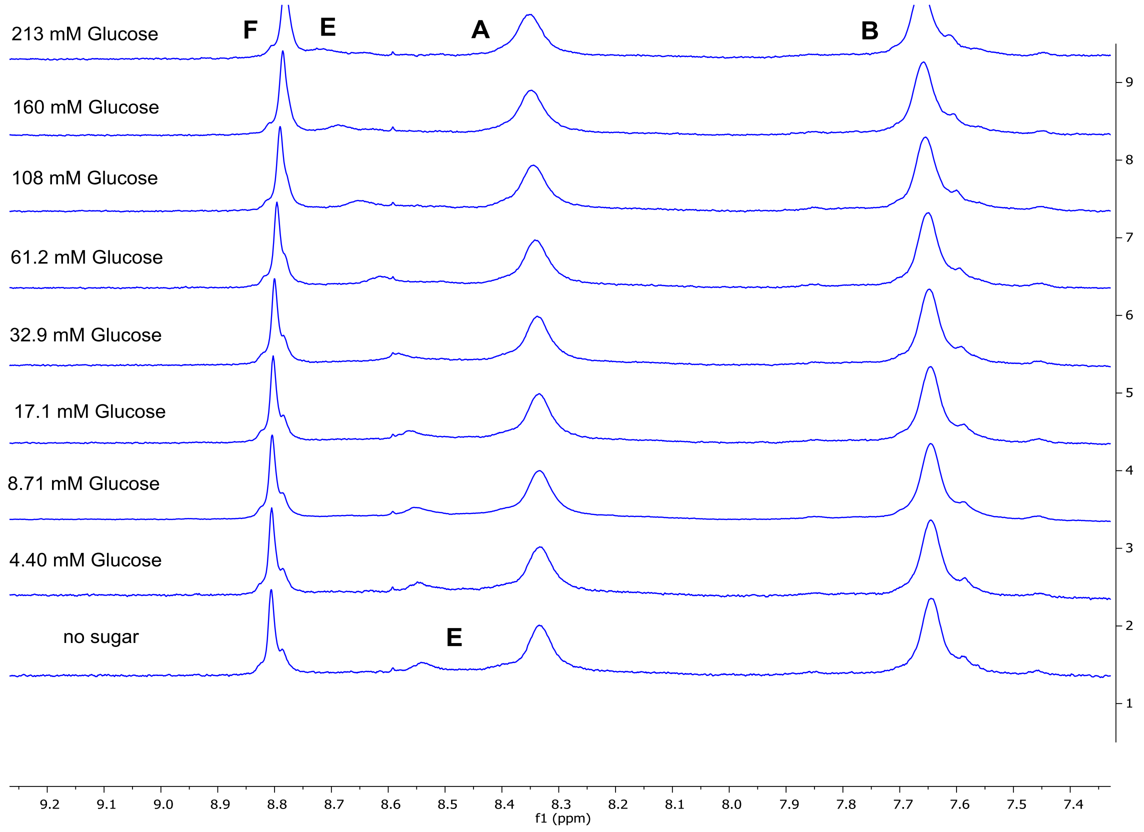

Figure S170. Partial ^{1}H NMR spectra from the titration of receptor **9** (0.24 mM) with D-glucose **3** in D_2O at 298 K.

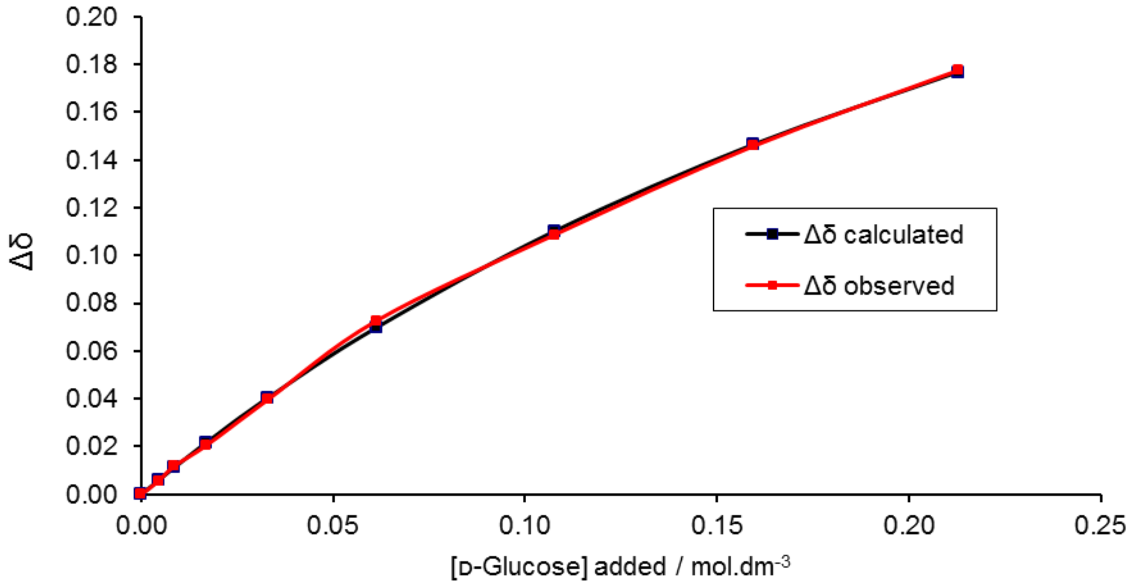

Figure S171. Experimental and calculated values for the NMR binding study of receptor **9** (0.24 mM) with D-glucose **3** in D_2O at 298 K. Proton E: K_a = 4 M^{-1} ± 4%, Δδ = 0.46.

ᴅ-Glucosamine 13

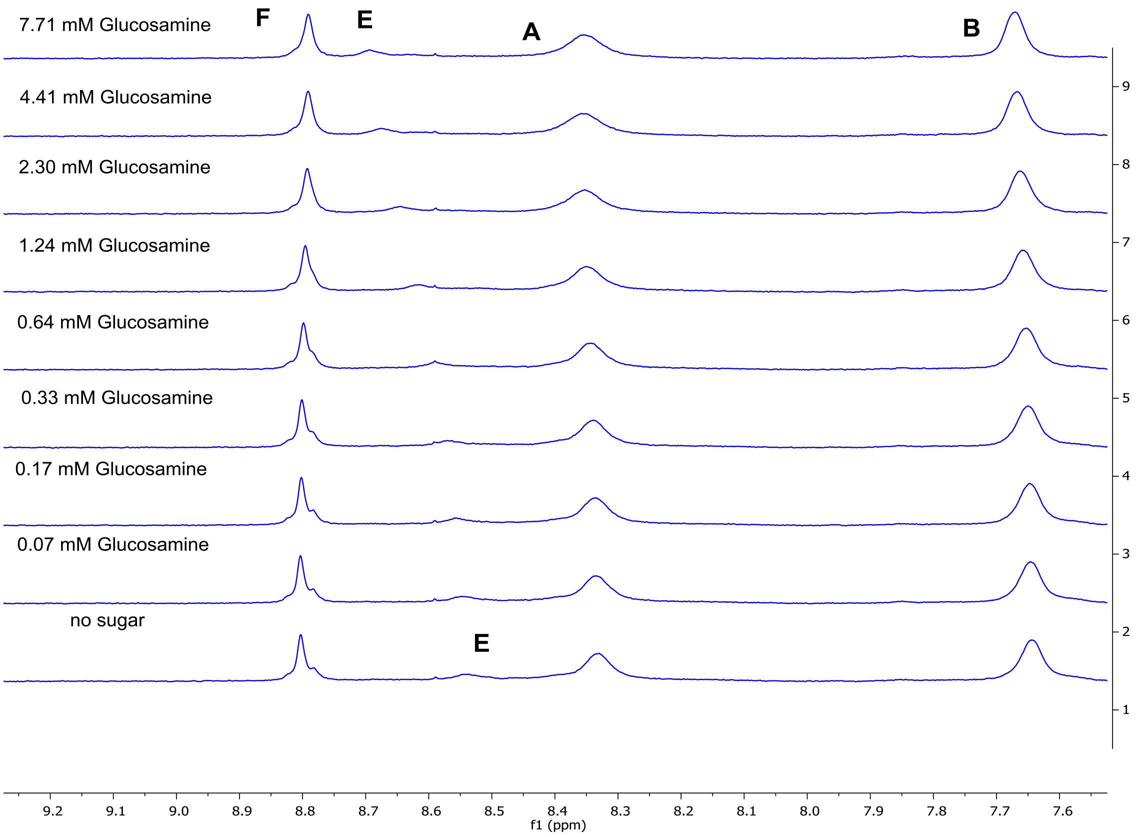

Figure 172. Partial ^{1}H NMR spectra from the titration of receptor **9** (0.24 mM) with D-glucosamine **13** in D_2O (pH = 7) at 298 K.

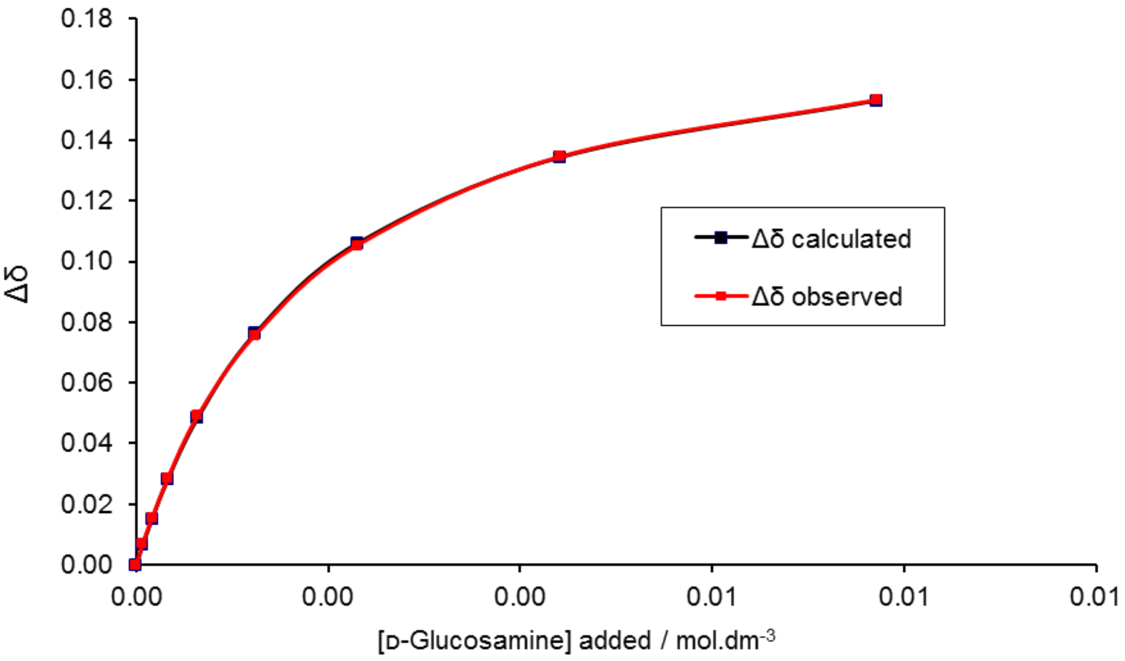

Figure S173. Experimental and calculated values for the NMR binding study of receptor **9** (0.24 mM) with D-glucosamine **13** in D_2O at 298 K. Proton E: K_a = 610 M^{-1} ± 4%, Δδ = 0.18.

D-Glucosamine 13 (NaCl 20 mM)

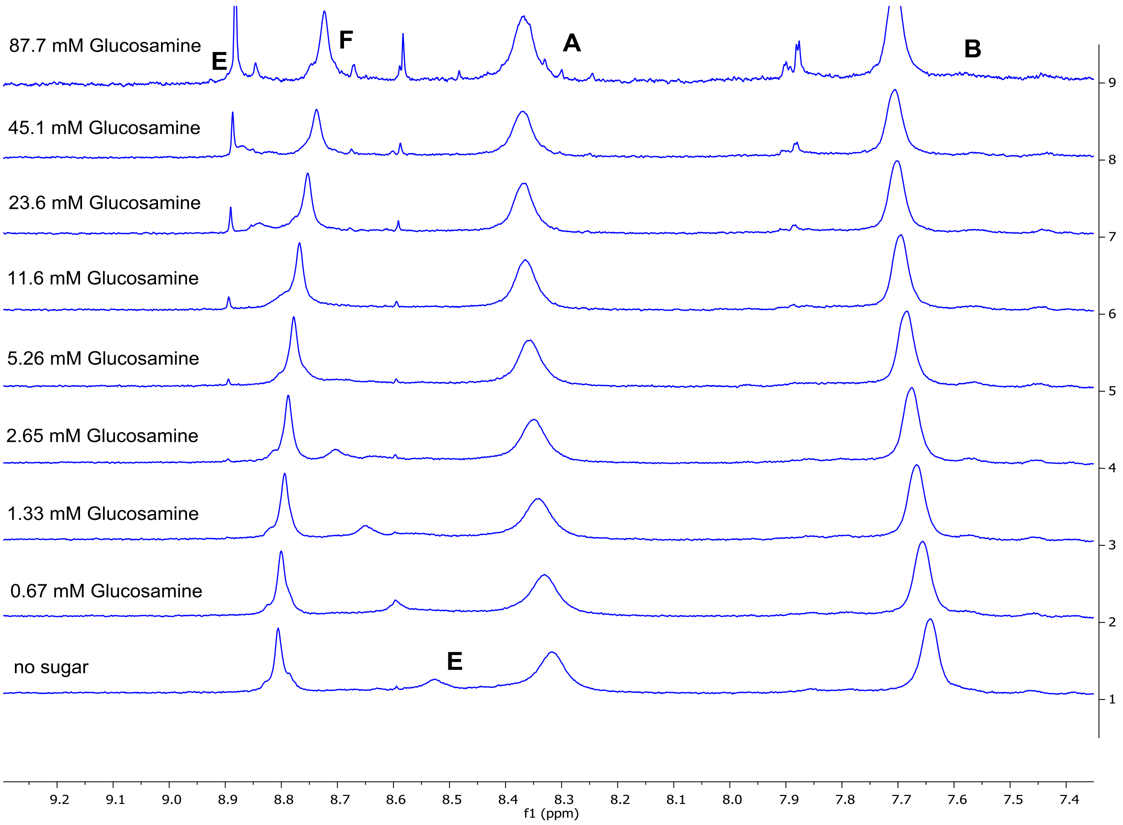

Figure S174. Partial ^{1}H NMR spectra from the titration of receptor **9** (0.19 mM) with D-glucosamine **13** in D_2O with NaCl (20 mM) at 298 K.

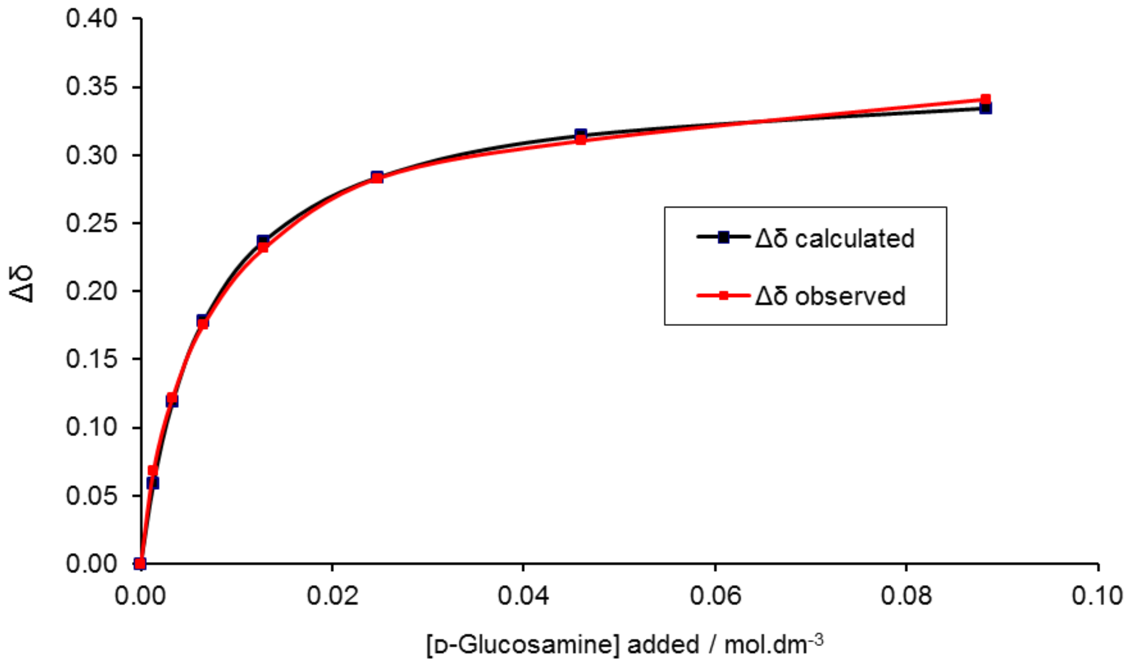

Figure S175. Experimental and calculated values for the NMR binding study of receptor **9** (0.19 mM) with D-glucosamine **13** in D_2O with NaCl (20 mM) at 298 K. Proton E: K_a = 151 M^{-1} ± 16%, Δδ = 0.35.

D-Glucosamine 13 (NaCl 154 mM)

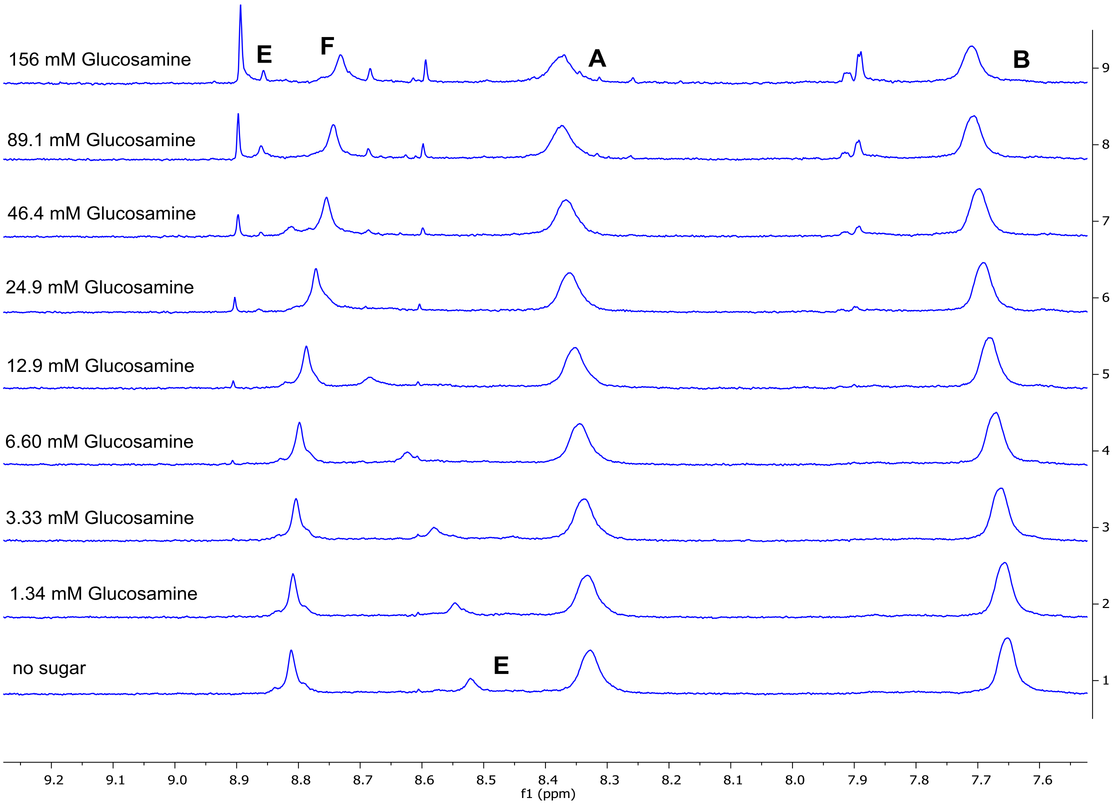

Figure 176. Partial ^{1}H NMR spectra from the titration of receptor **9** (0.19 mM) with D-glucosamine **13** in D_2O with NaCl (154 mM) at 298 K.

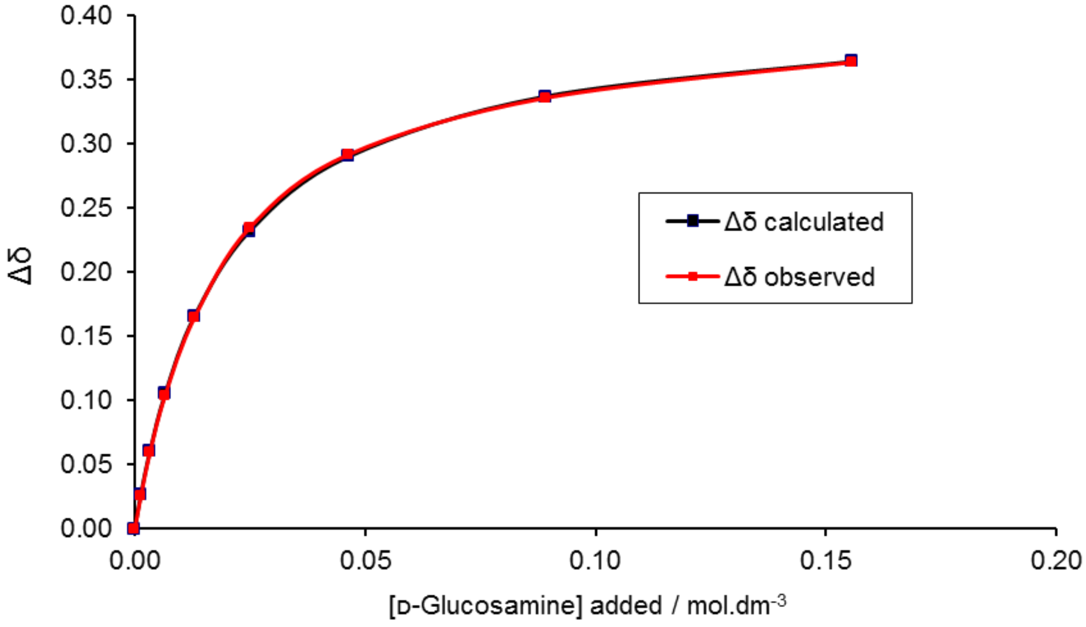

Figure S177. Experimental and calculated values for the NMR binding study of receptor **9** (0.186 mM) with D-glucosamine **13** in D_2O with NaCl (154 mM) at 298 K. Proton E: K_a = 53 M^{-1} ± 2%, Δδ = 0.40.

ᴅ-Galactosamine (NaCl 154 mM)

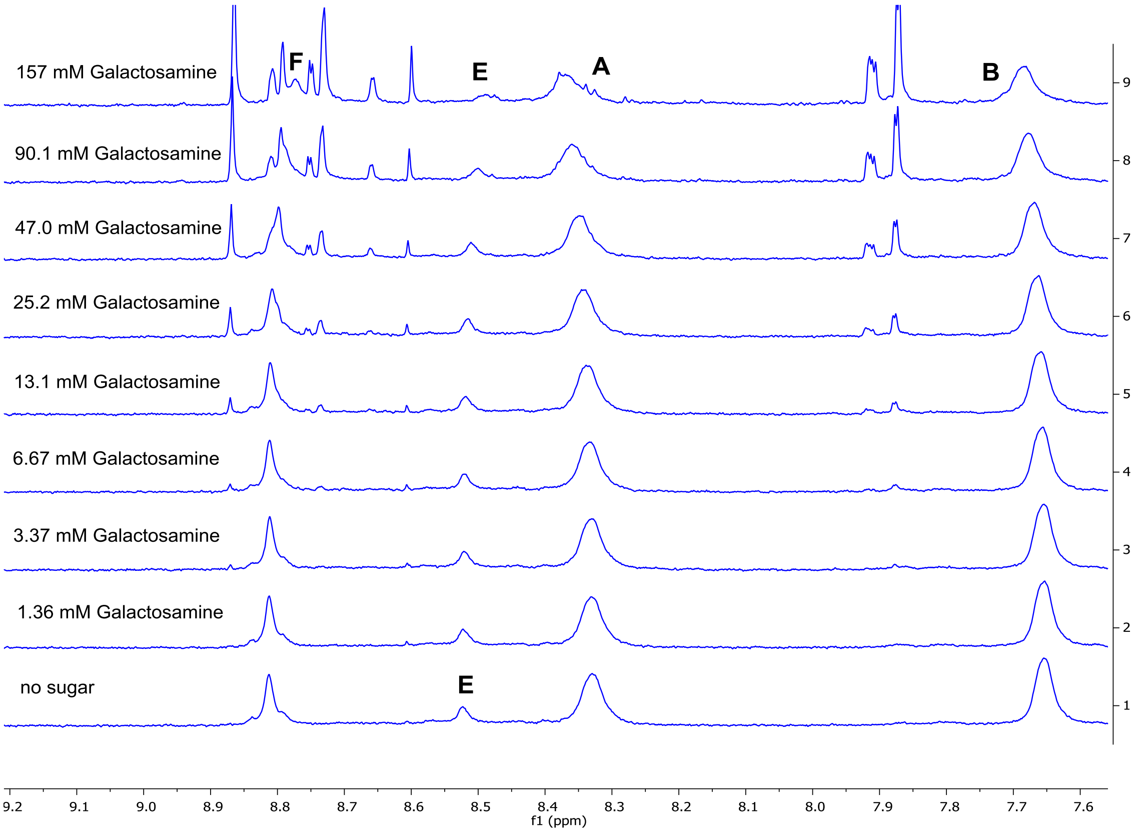

Figure S178. Partial ^{1}H NMR spectra from the titration of receptor **9** (0.186 mM) with D-galactosamine in D_2O with NaCl (154 mM) at 298 K.

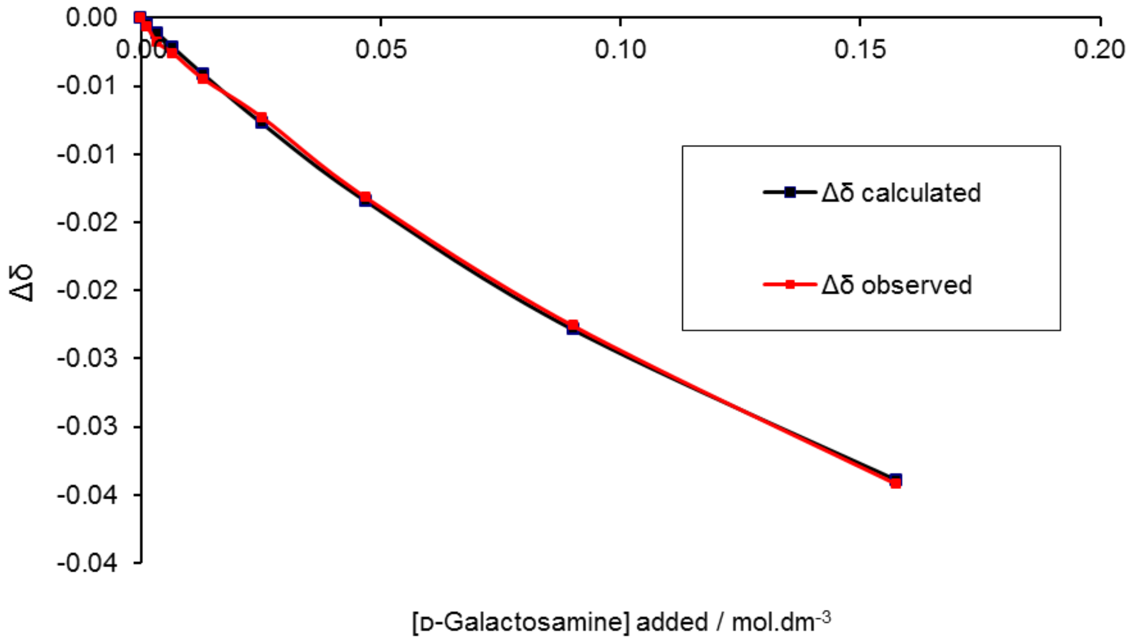

Figure S179. Experimental and calculated values for the NMR binding study of receptor **9** (0.19 mM) with D-galactosamine in D_2O with NaCl (154 mM) at 298 K. Proton E: K_a = 3 M^{-1} ± 25%, Δδ = -0.09.

Fluorescence Titrations

D-Glucose 3

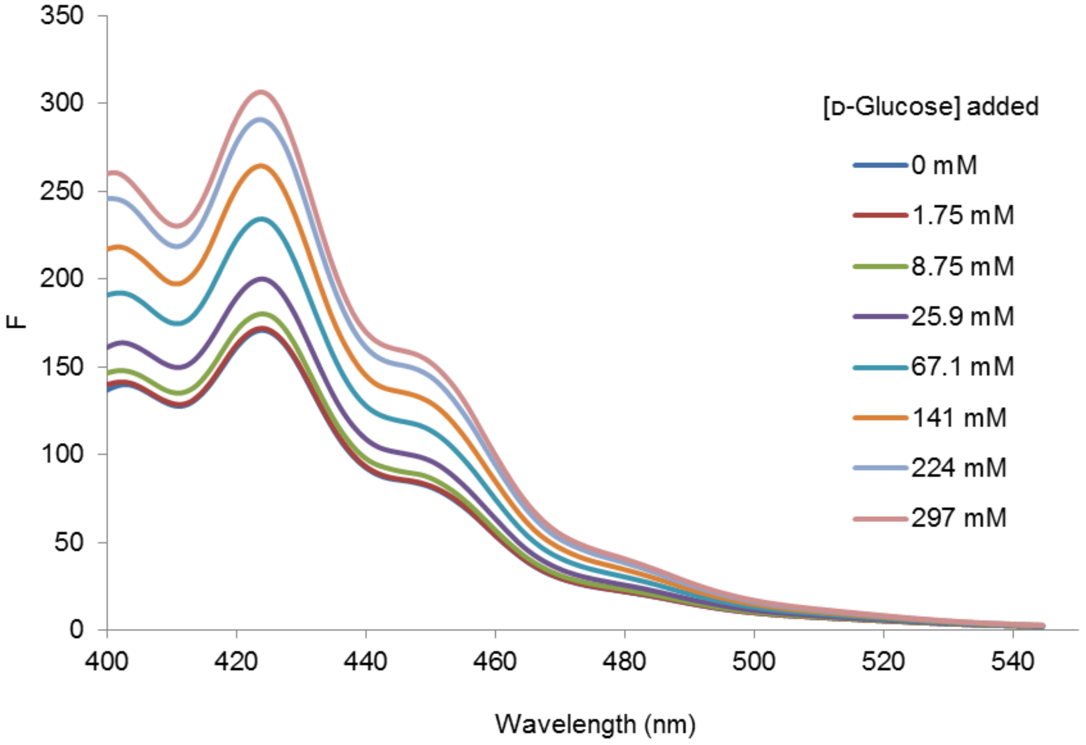

Figure S180. Fluorescence titration of receptor **9** (20.6 μM) with D-glucose **3** in phosphate buffer solution (pH = 7.1, 0.1 M) at 298 K. Excitation wavelength: 395 nm.

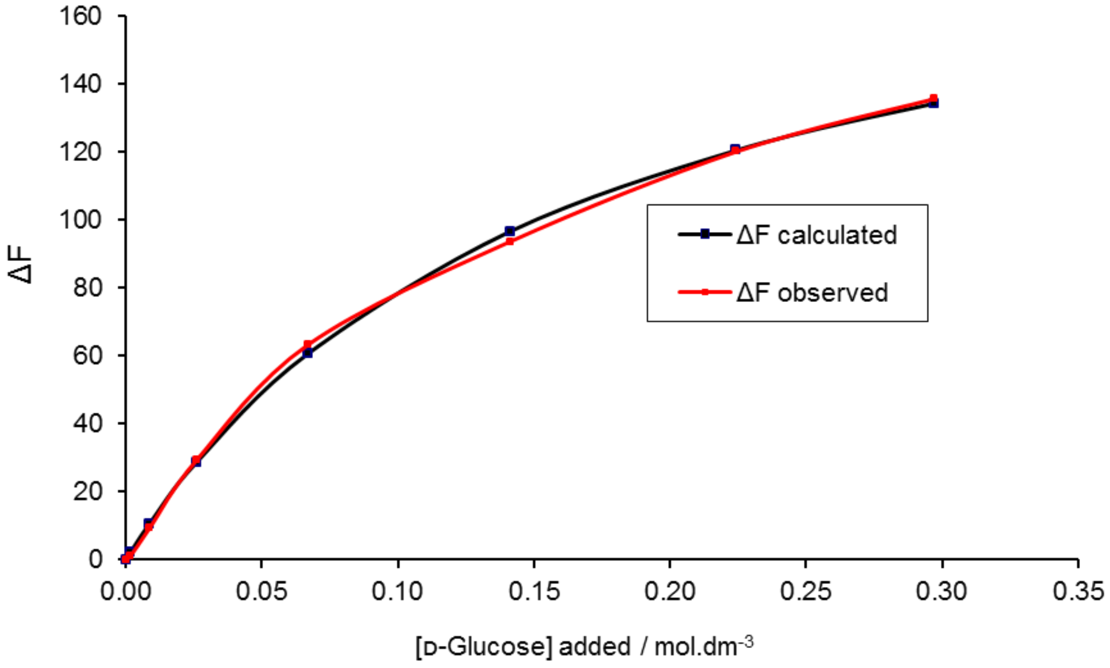

Figure S181. Experimental and calculated values for the fluorescence binding study of receptor **9** (20.6 μM) with D-glucose **3** in phosphate buffer solution (pH = 7.1, 0.1 M) at 298 K. Excitation: 395 nm. Emission observed at: 423 nm. K_a = 6 M^{-1} ± 18%. F/F^o = 1.21.

Comparison of Receptor Sensitivities Towards Glucose

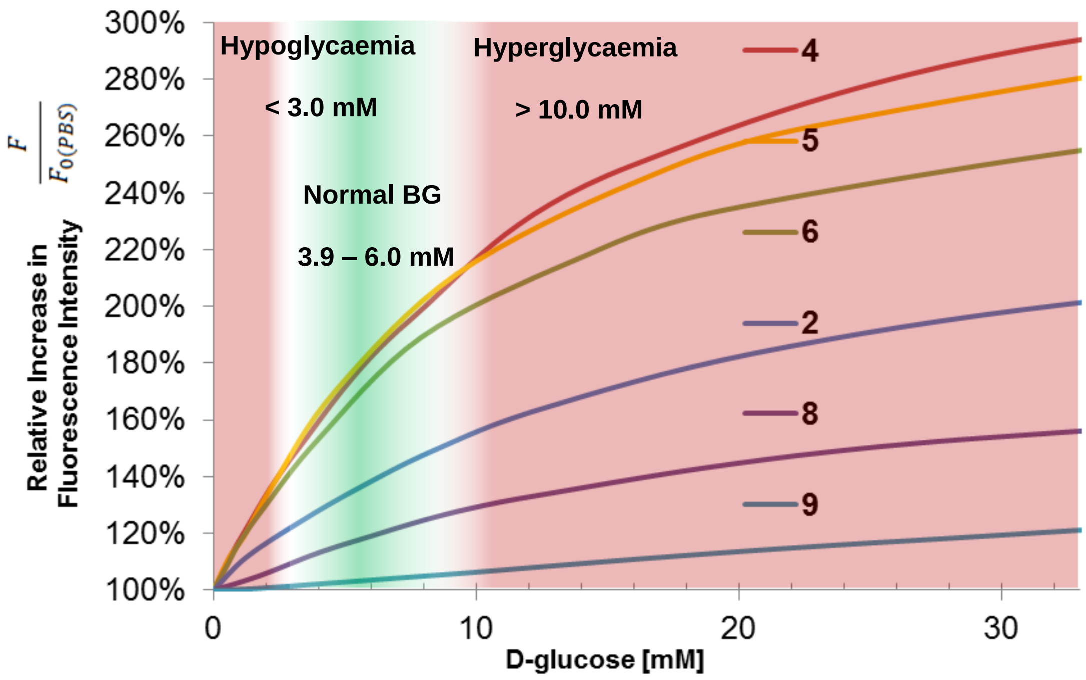

Figure S182. Overlaid fluorescence titrations from receptors **2** (18.8 µM), **4** (15.2 µM)**, 5** (18.8 µM)**, 6** (18.8 µM)**, 8** (15.6 µM) and **9** (20.6 µM) with D-glucose in PBS (0.1 M) at 298 K. Excitation wavelength: 394 nm, Emission wavelength: 423 nm. The slopes for **4**, **5** and **6** between 2 and 10 mM glucose are ~2 × higher than for previously published system **2**.

5. Molecular Modeling

General Methods

Structures were minimized using MacroModel 10.3, MMFFs force-field, aqueous GB/SA solvation. Constraints were used to arrange the complexes in chosen conformations, but were then removed before final minimizations. All the structures in Figures S183-S185 are the result of such unconstrained calculations.

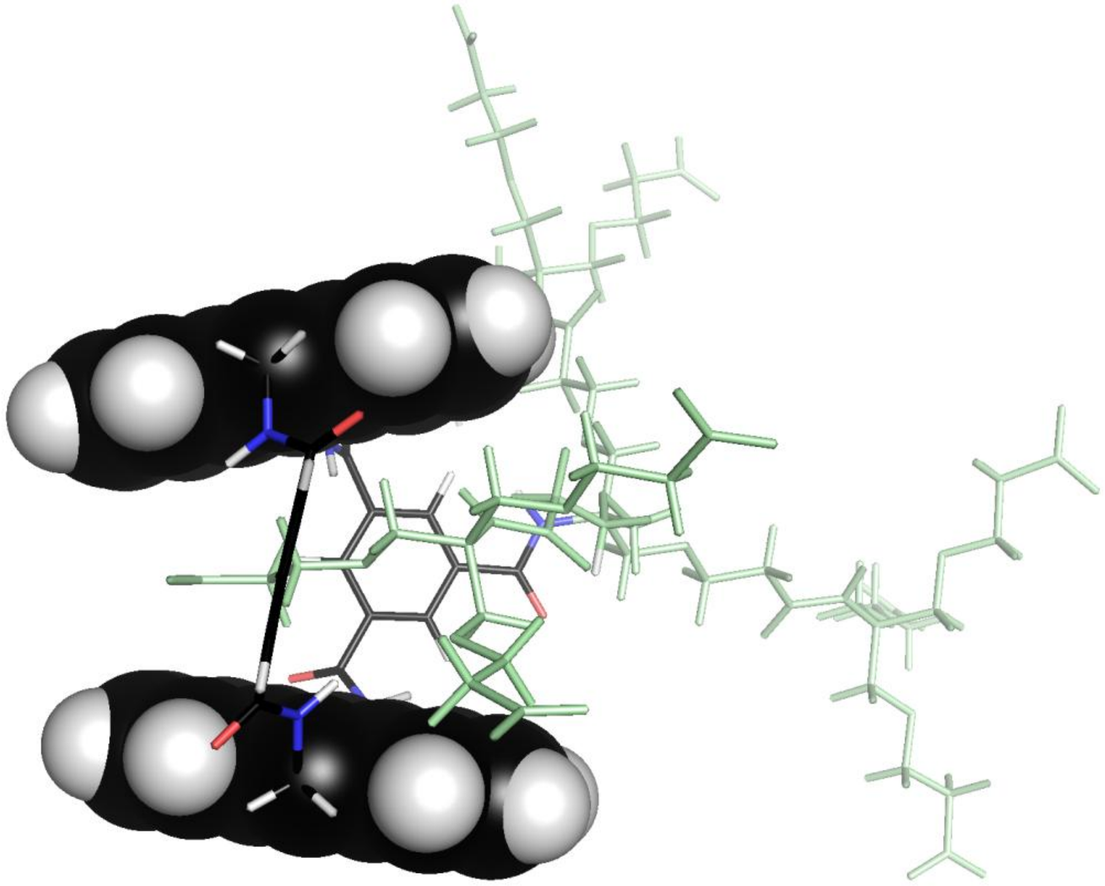

Figure S183. Model of **7** showing a terminal side-chain unit threading though the cavity. Anthracene units are shown in space-filling mode, side-chain atoms are pale green. The nearer side-chain has been omitted for clarity.

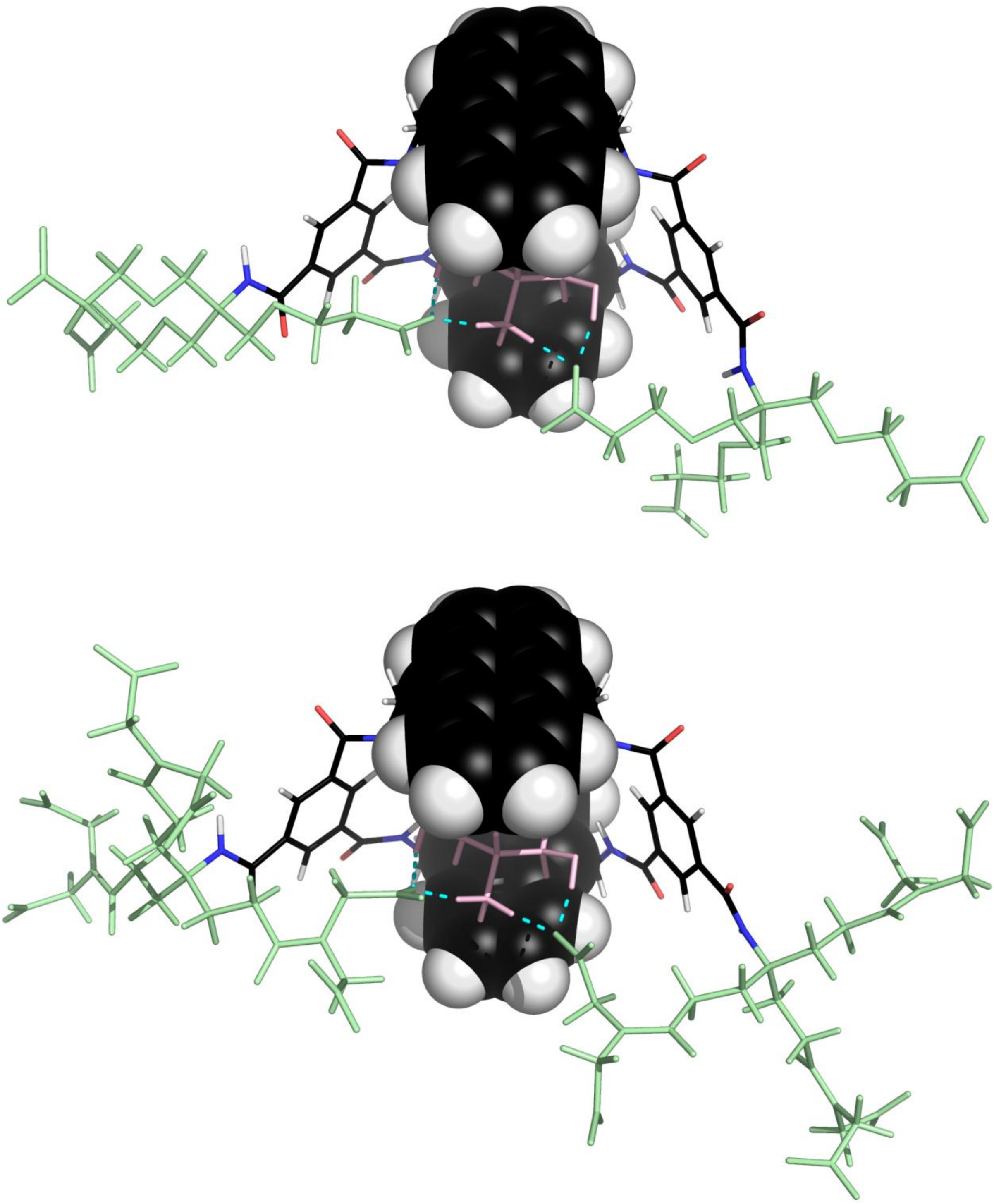

Figure S184. Models of **2** (above) and **4** (below) binding glucosammonium **13**·H$^+$, featuring salt bridge formation from side—chains on both sides of the receptor. Although the complexes remains intact, the angles of the isophthaloyl spacers (*cf.* Figure 3) suggest that both structures are significantly strained.

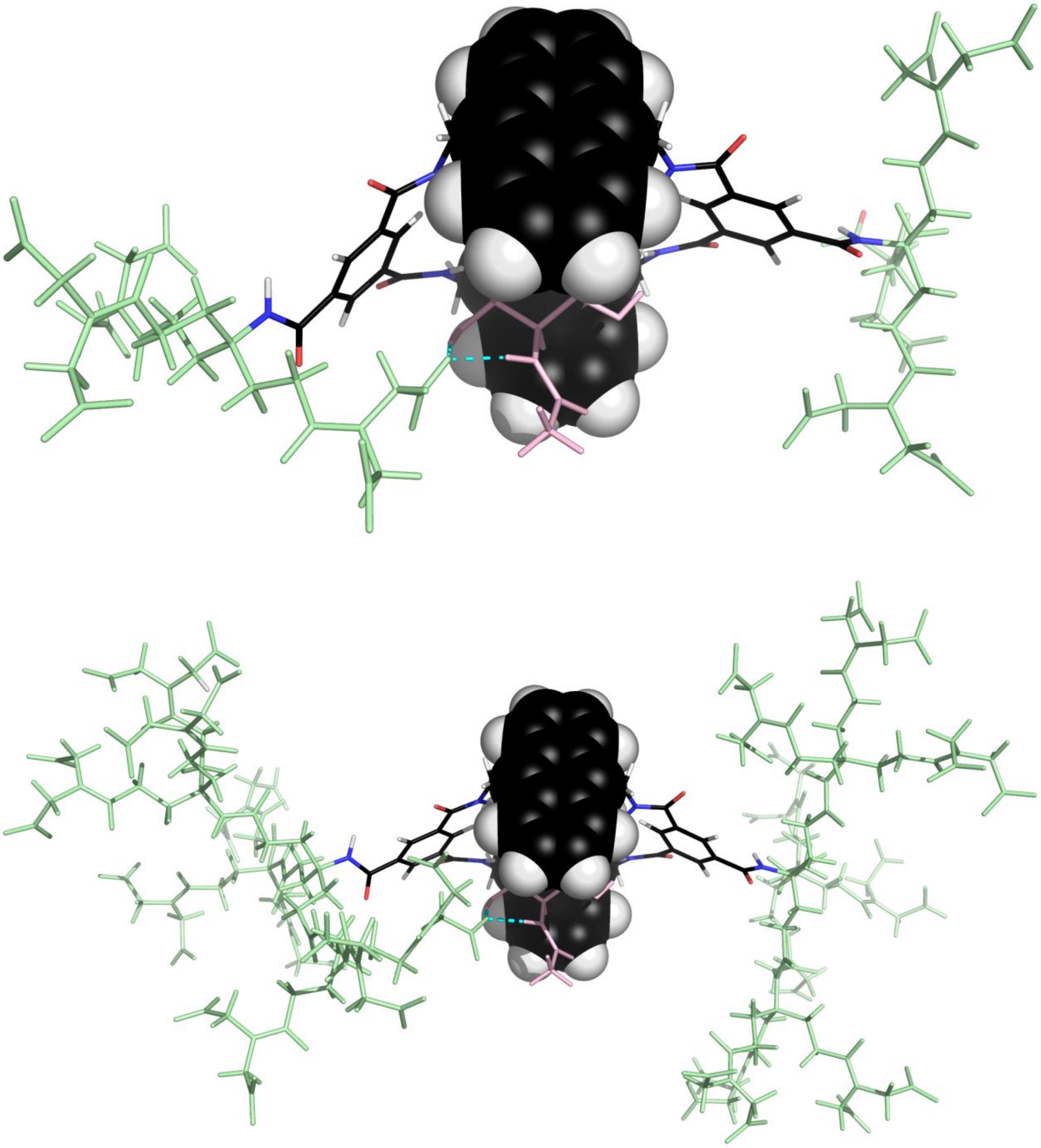

Figure S185. Models of **4** (above) and **8** (below) binding GlcNAc **15**, both featuring hydrogen bonds (cyan) from a side-chain carboxylate to NH and OH. In **4**·GlcNAc the hydrogen bonds survive minimisation but, as a consequence, an isophthaloyl spacer is pulled out of position. In **8**·GlcNAc there are no indications that the complex is strained.

References

[1] Commercially available from Frontier Scientific.

[2] Iorio, E. J.; Still, W. C. *Bioorg. Med. Chem. Lett.* **1999**, *9*, 2145-2150. Leung, D. K.; Atkins, J. H.; Breslow, R. *Tetrahedron Lett.* **2001**, *42*, 6255-6258.

[3] Ke, C.; Destecroix, H.; Crump, M. P.; Davis, A. P. *Nature Chem.* **2012**, *4*, 718-723.

[4] Klein, E.; Crump, M. P.; Davis, A. P. *Angew. Chem. Int. Ed.* **2005**, 44, 298.

[5] Cardona, C. M.; Gawley, R. E. *J. Org. Chem.* **2002**, 67, 1411.

[6] Brettreich, M.; Hirsch, A. *Synlett.* **1998**, 1998, 1396.

[7] Newkome, G. R.; Kotta, K. K.; Moorefield, C. N. *J. Org. Chem.* **2005**, *70*, 4893.
